# Fragment-Sized and Bidentate (Immuno)Proteasome Inhibitors Derived from Cysteine and Threonine Targeting Warheads

**DOI:** 10.3390/cells10123431

**Published:** 2021-12-06

**Authors:** Levente Kollár, Martina Gobec, Matic Proj, Lara Smrdel, Damijan Knez, Tímea Imre, Ágnes Gömöry, László Petri, Péter Ábrányi-Balogh, Dorottya Csányi, György G. Ferenczy, Stanislav Gobec, Izidor Sosič, György M. Keserű

**Affiliations:** 1 Medicinal Chemistry Research Group, Research Centre for Natural Sciences, Magyar Tudósok Krt. 2, H-1117 Budapest, Hungary; kollar.levente@ttk.hu (L.K.); petri.laszlo@ttk.mta.hu (L.P.); abranyi-balogh.peter@ttk.hu (P.Á.-B.); csanyi.dorottya@ttk.hu (D.C.); ferenczy.gyorgy@ttk.hu (G.G.F.); 2 Faculty of Pharmacy, University of Ljubljana, Aškerčeva cesta 7, SI-1000 Ljubljana, Slovenia; martina.gobec@ffa.uni-lj.si (M.G.); matic.proj@ffa.uni-lj.si (M.P.); lara.smrdel@ffa.uni-lj.si (L.S.); damijan.knez@ffa.uni-lj.si (D.K.); stanislav.gobec@ffa.uni-lj.si (S.G.); 3 MS Metabolomics Research Group, Research Centre for Natural Sciences, Magyar Tudósok Krt. 2, H-1117 Budapest, Hungary; imre.timea@ttk.hu; 4 MS Proteomics Research Group, Research Centre for Natural Sciences, Magyar Tudósok Krt. 2, H-1117 Budapest, Hungary; gomory.agnes@ttk.mta.hu

**Keywords:** immunoproteasome, benzoxazole-2-carbonitriles, bidentate covalent inhibitors, fragments, non-covalent recognition

## Abstract

Constitutive- and immunoproteasomes are part of the ubiquitin–proteasome system (UPS), which is responsible for the protein homeostasis. Selective inhibition of the immunoproteasome offers opportunities for the treatment of numerous diseases, including inflammation, autoimmune diseases, and hematologic malignancies. Although several inhibitors have been reported, selective nonpeptidic inhibitors are sparse. Here, we describe two series of compounds that target both proteasomes. First, benzoxazole-2-carbonitriles as fragment-sized covalent immunoproteasome inhibitors are reported. Systematic substituent scans around the fragment core of benzoxazole-2-carbonitrile led to compounds with single digit micromolar inhibition of the β5i subunit. Experimental and computational reactivity studies revealed that the substituents do not affect the covalent reactivity of the carbonitrile warhead, but mainly influence the non-covalent recognition. Considering the small size of the inhibitors, this finding emphasizes the importance of the non-covalent recognition step in the covalent mechanism of action. As a follow-up series, bidentate inhibitors are disclosed, in which electrophilic heterocyclic fragments, i.e., 2-vinylthiazole, benzoxazole-2-carbonitrile, and benzimidazole-2-carbonitrile were linked to threonine-targeting (*R*)-boroleucine moieties. These compounds were designed to bind both the Thr1 and β5i-subunit-specific residue Cys48. However, inhibitory activities against (immuno)proteasome subunits showed that bidentate compounds inhibit the β5, β5i, β1, and β1i subunits with submicromolar to low-micromolar IC_50_ values. Inhibitory assays against unrelated enzymes showed that compounds from both series are selective for proteasomes. The presented nonpeptidic and covalent derivatives are suitable hit compounds for the development of either β5i-selective immunoproteasome inhibitors or compounds targeting multiple subunits of both proteasomes.

## 1. Introduction

Protein degradation was considered a neglected field before the 1980s, but the discovery of ubiquitin pathways has put the topic in a different perspective [1]. The ubiquitin–proteasome system (UPS) [2] and, in particular, the multicatalytic activity of the 26S proteasome [3,4,5,6,7] play a fundamental role in important cellular processes such as apoptosis [8], immune response [9], cell cycle progression [10], and regulation of transcription.

The complex structure of the proteasome can be divided into smaller units. The 20S core particle (CP), which is responsible for the catalytic activity, consists of four stacked rings, each containing seven subunits. Three subunits of the inner β-rings are proteolytically active and are defined as the β1 subunit (caspase-like), the β2 subunit (trypsin-like), and the β5 subunit (chymotrypsin-like). The differential substrate preference of these subunits enables the degradation of diverse ubiquitinated proteins [11,12].

The immunoproteasome (iCP) represents an isoform of the constitutive proteasome (cCP), which is expressed primarily in cells of hematopoietic origin. When exposed to inflammatory stimuli, such as tumor necrosis factor-α or interferon-γ, iCP expression is induced in non-hematopoietic cells. The β-rings of iCP contain unique β subunits (β1i, β2i, and β5i), which replace their constitutive counterparts [13,14,15,16]. Increased expression of proteasomes has been associated with inflammatory and autoimmune diseases, certain cancers, and neurodegenerative disorders [3,17,18,19]. Both cCP and iCP are highly valuable pharmacological targets and selective inhibition of their subunits represents a viable option for therapy of these diseases. Several iCP inhibitors have been reported; however, they are predominantly peptides or peptidomimetics that have low oral bioavailability and limited metabolic stability. The selectivity of inhibitors is also crucial, as most iCP inhibitors have significant activity against multiple proteasomal subunits, leading to undesirable side-effects in clinical settings [5,20,21]. Recently, we discovered benzo[*d*]oxazole-2-carbonitriles, benzo[*d*]thiazole-2-carbonitriles, benzo[*d*]imidazole-2-carbonitriles, and 1-methylbenzo[*d*]imidazole-2-carbonitriles (using a general term: benzoXazole-2-carbonitriles) as selective fragment-sized iCP inhibitors [22]. Out of this set of benzoXazole-2-carbonitrile cores, the benzoxazole-2-carbonitrile scaffold showed the most promising inhibitory activities. In the present paper, we prepared and evaluated two series of compounds. The first focuses on the preliminary optimization of benzoxazole-2-carbonitriles as covalent fragment-like inhibitors of the β5i subunit of iCP. The second series, representing a continuation of the first one, is based on both benzoxazole- and benzimidazole-2-carbonitriles, as well as on 2-vinylthiazole, a hit compound obtained in the screening of a collection of fragment-sized electrophilic heterocyclic compounds [23] against iCP. We linked these molecules to a threonine-targeting (*R*)-boroleucine moiety to obtain bidentate compounds that could bind to both Thr1, present in all iCP and cCP subunits, and Cys48 found in the β5i subunit of iCP. The synthetic pathways towards these compounds are presented, followed by their biochemical characterization. The latter included the evaluation of inhibitory activities on all catalytic cCP and iCP subunits, together with biophysical and computational studies to analyze the effects of warhead and substituents on the inhibition mechanism and potency.

## 2. Materials and Methods

### 2.1. General Chemistry Methods

Reagents and solvents were obtained from commercial sources (Sigma Aldrich, St. Louis, MO, USA; TCI Europe, Zwijndrecht, Belgium; Merck KGaA, Darmstadt, Germany; Alfa Aesar, Haverhill, MA, USA; Combi-Blocks, San Diego, CA, USA; Fluorochem, Ltd., Hadfield, UK) and were used as received. Bortezomib, carfilzomib, and PR-957 were purchased from MedChemExpress. For reactions with air- or moisture-sensitive reagents, solvents were distilled before use and these reactions were performed under nitrogen or argon atmosphere. Flash column chromatography was performed using a CombiFlash Rf 200 instrument (Teledyne ISCO, Lincoln, NE, USA). In the case of reversed-phase chromatography, RediSep Rf reversed-phase C18 columns (4.3 g, 26 g, 43 g, 86 g) were used. Melting points were determined using a Reichelt hot stage apparatus and are uncorrected. ^1^H and ^13^C NMR spectra were recorded at 295 K on a Varian System 500 NMR spectrometer (Varian, Palo Alto, CA, USA) or Varian System 300 NMR spectrometer operating at frequencies for ^1^H NMR at 500 MHz or 300 MHz, and for ^13^C NMR at 126 MHz or 75 MHz, respectively. The chemical shifts (δ) are reported in parts per million (ppm) and are referenced to the deuterated solvent used. The coupling constants (*J*) are given in Hz, and the splitting patterns are designated as follows: s, singlet; br s, broad singlet; d, doublet; app d, apparent doublet; dd, doublet of doublets; ddd, doublet of doublets of doublets; t, triplet; dt, doublet of triplets; td, triplet of doublets; m, multiplet. All ^13^C NMR spectra were proton decoupled. HPLC-MS measurements were performed using a Shimadzu LC-MS-2020 instrument (Shimadzu Corporation, Kyoto, Japan) equipped with a Reprospher 100 C18 (5 mm, 100 × 3 mm) column and a positive–negative double ion source (DUIS±) with a quadrupole mass spectrometer in a range of 50–1000 m/z. Samples were eluted by gradient elution using eluent A (0.1% HCOOH in H_2_O) and eluent B (0.1% HCOOH in MeCN). The flow rate was set to 1.5 mL/min. The initial condition was 0% B eluent, followed by a linear gradient to 100% B eluent by 2 min, from 2 to 3.75 min 100% B eluent was maintained, and from 3.75 to 4.5 min back to the initial condition and maintained to 5 min. The column temperature was kept at 30 °C and the injection volume was 1 µL. HRMS measurements (ESI^+^, ESI^–^, APCI^+^ and APCI^–^) were performed for all new compounds, except for **1**, **2**, **3**, **10,** and **11,** which were not ionizable. This phenomenon was also observed for some other benzoxazole-2-carbonitrile derivatives [22].

#### 2.1.1. General Methods for the Synthesis of Benzoxazole-2-Carbonitriles Using Appel’s Salt

Method A. To a solution of an appropriate 2-aminophenol derivative (1.0 equiv.) in pyridine (6 mL for 1.0 mmol of the 2-aminophenol derivative), Appel’s salt (1.1 equiv.) was added portion-wise. The reaction mixtures were stirred at different temperatures and the reaction time was also varied (see Supplementary Materials for details). After completion of the reaction, the volatiles were removed under reduced pressure and the crude residue was purified by reversed-phase flash column chromatography using eluents A (0.1% HCOOH in MeCN) and B (0.1% HCOOH in H_2_O) (gradient from 1:9 to 10:0).
Method B. To a solution of an appropriate 2-aminophenol derivative (1.0 equiv.) in anhydrous THF (6 mL for 1.0 mmol of the 2-aminophenol derivative), Appel’s salt (1.0 equiv.) was added portion-wise. The reaction mixture was stirred at RT for 1 h and then the volatiles were removed under reduced pressure. The residue was re-dissolved in DMSO (4 mL for 1.0 mmol of the 2-aminophenol derivative), the reaction mixture was stirred at 100 °C for 1 h. The volatiles were removed under reduced pressure and the crude residue was purified by reversed-phase flash column chromatography using eluents A (0.1% HCOOH in MeCN) and B (0.1% HCOOH in H_2_O) (gradient from 1:9 to 10:0).
Method C. To a solution of an appropriate 2-aminophenol derivative (1.0 equiv.) in anhydrous THF (6 mL for 1.0 mmol of the 2-aminophenol derivative), Appel’s salt (1.0 equiv.) was added portion-wise. The reaction mixture was stirred at 140 °C for 20 min in a microwave reactor. After completion of the reaction, the volatiles were removed under reduced pressure and the crude residue was purified by reversed-phase flash column chromatography using eluents A (0.1% HCOOH in MeCN) and B (0.1% HCOOH in H_2_O) (gradient from 1:9 to 10:0).

#### 2.1.2. Alternative Method for the Synthesis of Benzoxazole-2-Carbonitriles

Step 1: To a solution of an appropriate 2-aminophenol derivative (1.0 equiv.) in EtOH:H_2_O (5:1, 6 mL for 1.0 mmol of the 2-aminophenol derivative), CS_2_ (1.0 equiv.) and solid KOH (1.0 equiv.) were added. The reaction mixtures were stirred at different temperatures and the reaction time was also varied (see Supplementary Materials for details). After the reaction was complete, it was cooled to room temperature, diluted with H_2_O (20 mL for 1.0 mmol of the 2-aminophenol derivative), and the pH was adjusted to 1 with 10% HCl. The precipitate formed was filtered off and washed with H_2_O (2 × 30 mL).
Step 2: To the obtained 2,3-dihydro-1,3-benzoxazole-2-thiol derivative, SOCl_2_ (6 mL for 1.0 mmol of the 2,3-dihydro-1,3-benzoxazole-2-thiol derivative) was added drop-wise, followed by the addition of DMF (one drop). The reaction mixture was refluxed for 1 h, the volatiles were then removed under reduced pressure and the crude 2-chloro-2,3-dihydro-1,3-benzoxazole derivative was used in the next step without further purification.
Step 3: To a solution of the obtained 2-chloro-2,3-dihydro-1,3-benzoxazole derivative (1.0 equiv.) in DMF (8 mL for 1.0 mmol of the 2-chloro-2,3-dihydro-1,3-benzoxazole derivative), KCN (1.4 equiv.) was added. The reaction mixtures were stirred at different temperatures and the reaction time was also varied (see Supplementary Materials for details). After the reaction was complete, it was diluted with H_2_O (30 mL) and extracted with DCM (3 × 30 mL). The volatiles were removed under reduced pressure and the crude residue was purified by reversed-phase flash column chromatography using eluents A (0.1% HCOOH in MeCN) and B (0.1% HCOOH in H_2_O) (gradient from 1:9 to 10:0).

#### 2.1.3. General O-Acylation Procedure

To a solution of the corresponding phenol (1.0 equiv.) in MeCN (2 mL), acetic anhydride (3 equiv.) and concentrated H_2_SO_4_ (one drop) were added, and the mixture was stirred at 60 °C for 1 h. After completion of the reaction, the crude product was purified by reversed-phase flash column chromatography using eluents A (0.1% HCOOH in MeCN) and B (0.1% HCOOH in H_2_O) (gradient from 1:9 to 10:0).

#### 2.1.4. General Nitro Reduction Procedure

To a solution of the appropriate nitro-containing compound (1.0 equiv.) in EtOAc (10 mL for 1.0 mmol of nitro-containing compound), SnCl_2_ × 2H_2_O (6.0 equiv.) was added. The reaction was stirred at 70 °C for 2 h. After the reaction was completed, it was diluted with EtOAc (80 mL), washed with saturated aqueous NaHCO_3_ (2 × 80 mL) and brine (20 mL), dried with MgSO_4_, and evaporated. It was purified by reversed-phase flash column chromatography using eluents A (0.1% HCOOH in MeCN) and B (0.1% HCOOH in H_2_O) (gradient from 1:9 to 10:0).

#### 2.1.5. General *N*-Acylation Procedure

To a solution of the corresponding amine (1.0 equiv.) in DMF (2 mL), Et_3_N (3 equiv.), 4-dimethylaminopyridine (0.1 equiv.) and benzoic anhydride (3 equiv.) were added. The reaction mixtures were stirred at different temperatures and the reaction time was also varied (see below). After completion of the reaction, the crude product was purified by reversed-phase flash column chromatography using eluents A (0.1% HCOOH in MeCN) and B (0.1% HCOOH in H_2_O) (gradient from 1:9 to 10:0).

#### 2.1.6. General Synthetic Strategy for the Preparation of Boronic Acid Derivatives

The discovered β5i-inhibiting fragments (benzimidazole-2-carbonitrile, benzoxazole-2-carbonitrile, and 2-vinylthiazole) or their intermediates were expanded with different linkers, which contained terminal carboxylic acid functional groups (compounds **XXVI**, **XXX**, **XXXI**, **XXXII**, **XXXIII**, **XXXV**, **XXXVI**, **XLIII**, **XLVI**—see structures in the Supplementary Materials). These were used to acylate (*R*)-BoroLeu-(+)-pinanediol trifluoroacetate to obtain compounds **28–32**. After the introduction of the boronic acid ester, deprotection reactions were carried out by various methods leading to the free boronic acids (compounds **33**–**36** and **40**). Alternatively, the boronic ester-containing intermediates were converted into the corresponding fragment-containing molecules (compounds **37**–**39**). Further synthetic details and all spectroscopic analyses can be found in the Supplementary Materials.

### 2.2. Residual Activity Measurements

Residual activity measurements were performed at 10 μM final concentrations in the assay buffer (0.01% SDS, 50 mM Tris-HCl, 0.5 mM EDTA, pH 7.4). Stock solutions of the compounds were prepared in DMSO. To 50 μL of each compound in buffer, 25 μL of 0.8 nM human iCP or human cCP (both from Boston Biochem, Inc., Cambridge, MA, USA) were added. After incubation at 37 °C for 30 min, the reaction was started by adding of 25 μL of 100 μM relevant fluorogenic substrate: acetyl-Nle-Pro-Nle-Asp-AMC (Ac-nLPnLD-AMC, (Bachem, Bubendorf, Switzerland)) for β1, acetyl-Pro-Ala-Leu-7-amino-4-methylcoumarin (Ac-PAL-AMC, (Boston Biochem, Inc., Cambridge, MA, USA)) for β1i, *t*-butyloxycarbonyl-Leu-Arg-Arg-7-amino-4-methylcoumarin (Boc-LRR-AMC, (Bachem, Bubendorf, Switzerland)) for β2 and β2i, succinyl-Leu-Leu-Val-Tyr-7-amino-4-methylcoumarin (Suc-LLVY-AMC) (Bachem, Bubendorf, Switzerland) for β5 and β5i. Reaction progress was recorded on BioTek Synergy HT microplate reader by monitoring fluorescence at 460 nm (λ_ex_ = 360 nm) for 90 min at 37 °C. The initial linear ranges were used to calculate velocity and determine residual activity. In the case of evaluation of β1, βi, β2, and β2i activities, the assay buffer was modified by replacing SDS with the proteasomal activator PA28α (Boston Biochem, Inc., Cambridge, MA, USA).

### 2.3. Determination of IC_50_ Values

Final assay mixtures contained 0.2 nM human iCP or 0.2 nM human cCP in assay buffer (0.01% SDS (or PA28α in cases of β1, β1i subunits), 50 mM Tris-HCl, 0.5 mM EDTA, pH 7.4). Inhibitors were dissolved in DMSO and added to the black 96-well plates for at least eight different concentrations (the final concentration of DMSO was not more than 1%). After 30 min of incubation at 37 °C, the reaction was started by adding the substrate Suc-LLVY-AMC (for β5 and β5i) or Ac-nLPnLD-AMC (for β1) or Ac-PAL-AMC (for β1i). Fluorescence was monitored at 460 nm (λ_ex_ = 360 nm) for 90 min at 37 °C. The progress of the reactions was recorded and the initial linear ranges were used to calculate the velocity. IC_50_ values were calculated using GraphPadPrism (GraphPad Software, San Diego, CA, USA) and are means from at least three independent determinations.

To determine the IC_50_ shift for the selected compound on the β5i subunit, the same protocol was used, except for the 0 min (or 60 min) incubation time before addition of the substrate Suc-LLVY-AMC.

### 2.4. Screening of the Heterocyclic Electrophilic Compounds

Screening of heterocyclic electrophilic compounds was performed at concentration of 500 µM in assay buffer (0.01% SDS, 50 mM Tris-HCl, 0.5 mM EDTA, pH 7.4). To 50 µL of each compound in buffer, 25 µL of 0.8 nM human iCP (Boston Biochem, Inc., Cambridge, MA, USA) was added. After 30 min incubation at 37 °C, the reaction was started by adding of 25 µL of 100 mM Suc-LLVY-AMC (Bachem, Bubendorf, Switzerland), final concentration 25 µM). The reaction progress was recorded on BioTek Synergy HT microplate reader by monitoring fluorescence at 460 nm (λ_ex_ = 360 nm) for 90 min at 37 °C. The initial linear ranges were used to calculate the velocity and determine the residual activity.

### 2.5. Reactivity Assays

#### 2.5.1. UV-Vis-Based Stability and Reactivity Assay

The aqueous stability of all compounds was determined spectrophotometrically by following the changes in the absorption spectra of the compounds. The buffer solution (50 mM Tris-HCl, 0.5 mM EDTA, pH 7.4) was pre-incubated at 37 °C. Aliquots of the compounds studied (1 mM in DMSO) were transferred to a 96-well flat-bottom UV-transparent microplate (Corning, CLS3635, Corning Inc, Corning, NY, USA) containing the buffer solution to obtain a 50 μM solution containing 5% DMSO (final volume, 300 μL). The plate was incubated without lid at 37 °C in a plate reader (Synergy H4, BioTek Instruments, Inc., Santa Clara, CA, USA) and the absorbance spectrum (244–400 nm) was acquired in sweep mode after 0, 15, 30, 60, 120, 180, and 240 min using a discontinuous kinetic procedure in Gen5 software (BioTek Instruments, Inc., Santa Clara, CA, USA). The time required to read the entire plate was 3 min. To determine the baseline, the compound solution was replaced with pure DMSO and subtracted from each reading. Compounds with an absorbance maximum of less than 0.20 AU were not evaluated using these assays due to the high experimental error. For other compounds, the relative absorbance difference between the first time point and 60 min at the most responsive wavelength was calculated. If the relative absorbance difference for the compound in the buffer was below 0.1, between 0.1–0.2, and above 0.2, the compound was classified as stable, intermediate, and unstable, respectively.

The same method was used to determine the reactivity of stable and intermediate stable compounds with *N*-acetyl cysteine. In this experiment, the final concentrations of the reaction mixture were 50 µM compound, 0.5 mM *N*-acetyl cysteine and 5% DMSO in the buffer solution. The baseline for *N*-acetyl cysteine in the buffer solution was subtracted from each measurement. To detect hyperreactive compounds where the reaction with *N*-acetyl cysteine is complete before the first time point can be acquired, i.e., up to 3 min, the spectra of the compound in the buffer solution with or without 0.5 mM *N*-acetyl cysteine at the first time point (t = 0) were compared. Significant changes (i.e., relative absorbance difference at the most responsive wavelength above 0.1) indicate hyperreactivity. Figures were generated using Matplotlib v3.3.4 (https://github.com/matplotlib/matplotlib, accessed on 10 November 2021) for Python v3.7 (https://www.python.org/, accessed on 10 November 2021).

#### 2.5.2. HPLC-Based Stability and Reactivity Assay

GSH reactivity assay was performed as described by Petri et al. [24]; 500 μM solution of compound (PBS buffer pH 7.4, 10% MeCN, 250 μL) with 200 μM solution of indoprofen as internal standard were added to 10 mM GSH solution (dissolved in PBS buffer, 250 μL) in a 1:1 ratio. The final concentration was 250 μM compound, 100 μM indoprofen, 5 mM GSH and 5% MeCN (500 μL). The final mixture was analyzed by HPLC after 0, 1, 2, 4, 8, 12, 24, 48, and 72 h. The degradation kinetics were also studied using the method described in [24], except that pure PBS buffer was used instead of GSH solution. In this experiment, the final concentration of the mixture was 250 μM fragment, 100 μM indoprofen and 5% MeCN. The area under the curve (AUC) values were determined by integrating the HPLC spectra and then corrected with the internal standard. The AUC values of the fragments were used for linear least squares regression and a programmed Excel (Visual Basic for Applications) was used to calculate the important parameters (kinetic rate constant, half-life. Data are expressed as means of duplicate determinations and standard deviations are within 10% of the reported values. Structures and data were manipulated and visualized with JChem for Office (ChemAxon, Budapest, Hungary) [25].

### 2.6. LC-MS Measurements

Molecular weights of β5i conjugates were identified using a Triple TOF 5600ţ hybrid Quadrupole-TOF LC/MS/MS system (AB Sciex LLC, Framingham, MA, USA) equipped with a Duo-Spray IonSource coupled to a Shimadzu Prominence LC20 UFLC (Shimadzu, Kyoto, Japan) system consisting of a binary pump, autosampler, and thermostatted column compartment. Data acquisition and processing were performed using Analyst TF software version 1.7.1 (AB Sciex LLC, Framingham, MA, USA). Chromatographic separation was performed on a Thermo Beta Basic C8 (50 mm × 2.1 mm, 3 µm, 150 Å) HPLC column. The sample was eluted in gradient elution mode using solvent A (0.1% HCOOH in H_2_O) and solvent B (0.1% HCOOH in MeCN). The initial condition was 20% B for 1 min, followed by a linear gradient to 90% B by 4 min, from 5 to 6 min 90% B was retained; and from 6 to 6.5 min back to initial condition with 20% eluent B and retained from 6.5 to 9.0 min. The flow rate was set to 0.4 mL/min. The column temperature was 40 °C and the injection volume was 5 μL. Nitrogen was used as the nebulizer gas (GS1), heater gas (GS2), and curtain gas with optimal values set at 30, 30 and 35 (arbitrary units), respectively. Data were recorded in positive electrospray mode in the mass range of *m*/*z* = 300 to 2500, with 1 s accumulation time. The source temperature was 350 °C and the spray voltage was set to 5500 V. The declustering potential value was set to 80 V. Peak View Software™ V.2.2 (AB Sciex LLC, Framingham, MA, USA) was used to deconvolute the raw electrospray data to obtain the neutral molecular masses.

### 2.7. Reactivity Calculations

The energy barrier of the reaction between substituted benzoxazole-2-carbonitriles and cysteamine was calculated based on an analogous study for the reaction between aromatic nitriles and cysteamine [26]. The following reaction was studied:

DFT calculations with B3LYP functional and 6-311++G** basis were used with conductor-like polarizable continuum water model [27,28]. First, the transition state was identified and validated by frequency calculations. Intrinsic reaction coordinate (IRC) [29] calculations were performed to track the reaction from the transition state to the reactants. The reactant endpoint of the IRC pathway was optimized by fixing the distance between the sulfur atom of the cysteamine and the carbon atom of the nitrile group to maintain the non-covalent complex geometry.

### 2.8. Computational Docking

Preparation of the compounds for docking included the generation of tautomeric and ionization states at pH 6–8 and the creation of 3D structures using LigPrep (Schrödinger Suite 2020-4: LigPrep, Schrödinger, LLC, New York, NY, USA, 2020). The X-ray structure deposited as PDB entry 6E5B [30] was used for docking. The binding site is defined by the K and L chains; therefore, all other chains as well as the covalently bound ligand were removed. Protein Preparation Wizard [31,32] was used to add hydrogen atoms, protonate residues at pH 7, refine the H-bond network, and to perform a restrained minimization. Cys48 was mutated to Ala to avoid steric clash in constraint docking. The receptor’s grid box required for docking calculations was centered on the C_β_ of Ala48. Docking with Glide (Schrödinger Suite 2020-4: Glide, Schrödinger, LLC, New York, NY, USA, 2020) was performed with the constraint that the carbon of the CN group should be within 3.5 Å of the C_β_ of Ala48.

### 2.9. Cholinesterase Assay

The activity of the compounds against ChEs, namely hAChE and hBChE, was determined by the Ellman method [33]. 5,5′-Dithiobis-2-nitrobenzoic acid (Ellman’s reagent; DTNB), butyrylthiocholine iodide, and acetylthiocholine iodides were from Sigma-Aldrich. Recombinant hAChE and hBChE were kindly provided by Xavier Brazzolotto, Florian Nachon, and José Dias (IRBA, Brétigny-sur-Orge, France). Reactions were performed in a final volume of 300 µL of 0.1 M phosphate-buffered solution, pH = 8.0, containing 370 µM DTNB, 500 μM butyrylthiocholine/acetylthiocholine iodide, and approximately 1 nM or 50–100 pM hBChE or hAChE, respectively. The reactions were started by the addition of the substrate after 30-min pre-incubation at room temperature. The final organic solvent (DMSO) content was always 1%. The formation of the yellow 5-thio-2-nitrobenzoate anion was monitored for 2 min at 412 nm, using a 96-well microplate reader (Synergy HT, BioTek Instruments, Inc., Santa Clara, CA, USA). The initial velocities in the presence (v_i_) and absence (v_o_) of the test compounds were calculated. Inhibitory potencies were expressed as residual activities (RA), according to the equation RA = (v_i_ − *b*)/(v_o_ − *b*), where *b* is the blank value.

### 2.10. Monoamine Oxidase Assay

The effects of the test compounds on hMAO were investigated using fluorimetric assay [34,35]. Recombinant hMAO-A and hMAO-B, expressed in BTI-TN-5B1-4 insect cells, horseradish peroxidase type II and *p*-tyramine hydrochloride were purchased from Sigma Aldrich. Amplex Red was synthesized as previously described [36].

Briefly, 50 mM sodium phosphate buffer (pH = 7.4, 0.05 vol.% Triton X-114) containing the compounds or the reference inhibitors and hMAO were incubated at 37 °C for 30 min. The reaction was started by adding Amplex Red (final concentration, 250 µM), horseradish peroxidase (final activity, 1 U/mL), and *p*-tyramine (final concentration, 1 mM). The increase in fluorescence (λ_ex_ = 530 nm, λ_em_ = 590 nm) was monitored for 30 min at 37 °C. DMSO at a concentration of 1.5% (*v*/*v*) was used for the control experiments. For the determination of blank (*b*), the enzyme was replaced by phosphate-buffered solution. Each measurement was performed in duplicate. Inhibitory potencies were expressed as residual activities (RA), as described under the Cholinesterase assay section.

### 2.11. Caspase-1 Assay

The inhibitory activity of the compounds against caspase-1 was investigated by fluorimetric assay using Ac-YVAD-AMC (SCP0069-5 mg, Sigma Aldrich, St. Louis, MO, USA) as a substrate. Recombinant human caspase-1 (expressed in *E. coli*, C5482, batch: SLBX3303, Sigma Aldrich, St. Louis, MO, USA) was diluted with 1 mL of assay buffer (50 mM HEPES pH 7.4, 100 mM NaCl, 1 mM EDTA, 10% (*v*/*v*) glycerol, 0.1% CHAPS, 1 mM DTT), divided into 50 µL aliquots and stored at −80 °C.

Briefly, compounds were incubated at a concentration of 100 µM (or 1% (*v*/*v*) DMSO as a control) with caspase-1 (1:5 dilution of caspase-1 stock solution) in assay buffer without DTT (to exclude reaction of compounds with DTT) in a 384-well microplate at room temperature for 30 min. The reaction was started by adding the substrate Ac-YVAD-AMC (final concentration: 20 µM) and monitored at λex = 360/40 and λem = 460/40 (Synergy HT, BioTek Instruments, Inc., Santa Clara, CA, USA) for 1 h. The initial linear ranges were used to calculate the velocity and determine RAs. *Z*-Val-Ala-DL-Asp-fluoromethylketone (N-1510, Bachem, Bubendorf, Switzerland) was used as a positive control.

## 3. Results and Discussion

**Figure 1:**
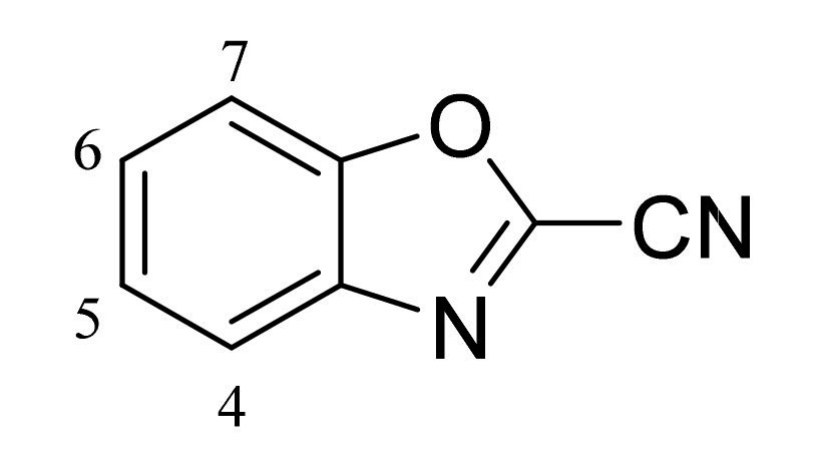
Structure of benzoxazole-2-carbonitrile with numbering of possible positions of substituents shown.

In the absence of structural information, the identification of growing vectors for the optimization of benzoxazole-2-carbonitriles (Figure 1) is challenging. Since, a priori, all positions around the fragment core have the same chance for growing, we therefore used substituent scans to collect structure-activity relationship data (SAR) for directional prioritization. One of the possible strategies for using SAR information is based on the concept of group efficiency (GE) [37]. Assuming similar binding modes for the compared molecules, GE evaluates the relative contribution of substituents to the free energy of binding. By synthesizing and testing a series of close analogues around the fragment core, the contributions of each substituent can be evaluated and positions prioritized according to their GE value [38]. To monitor a range of possible interactions around the benzoxazole-2-carbonitrile core, we used multiple substituents in the fragment scan. Full synthetic details (Schemes S1–S4) and spectroscopic characterization of the compounds can be found in the Supplementary Materials. First, we selected a chloro atom that could form halogen bonds in a polar environment and could also fit into apolar pockets (see Table 1 for IC_50_ values) [22].

Next, hydroxyl substituents were introduced to different positions, because this substituent can participate in H-bond interactions as both a donor and an acceptor. H-bond donor character was also monitored by the synthesis of amino derivatives and their synthetic precursors, nitro compounds. Hydrophobic interactions were monitored by the methyl scan (see Table 2).

All benzoxazole-2-carbonitriles, **5**–**27**, were first evaluated for their stability in the assay buffer using a UV-Vis-based high-throughput assay. Stability was assessed after 1 h to mimic conditions used in biochemical assays. Depending on the relative difference in absorbance at the most responsive wavelength, compounds were classified as stable, intermediate, and unstable (Table 2, Figure S1). The results were confirmed using a medium-throughput HPLC-based assay in phosphate-buffered saline (PBS), pH 7.4. Inhibitory potencies against the β5i subunit of human iCP were then determined. Data were calculated as residual β5i activities (RAs) in the presence of 10 µM of each compound. Only compounds that showed notable inhibition (>50%) were subjected to a dose-dependent measurement of inhibitory activity. The results are summarized in Table 2.

It is imperative to note that despite their purity and chemical stability, we observed limited buffer stability for chloro and nitro-substituted benzoxazoles. With the exception of derivatives **2**–**4**, **16**–**18**, and **26**, all other novel benzoxazole-2-carbonitriles were stable in the assay buffer (Table 2, Figure S1) and were therefore evaluated in the iCP inhibition assay.

**Figure 2:**
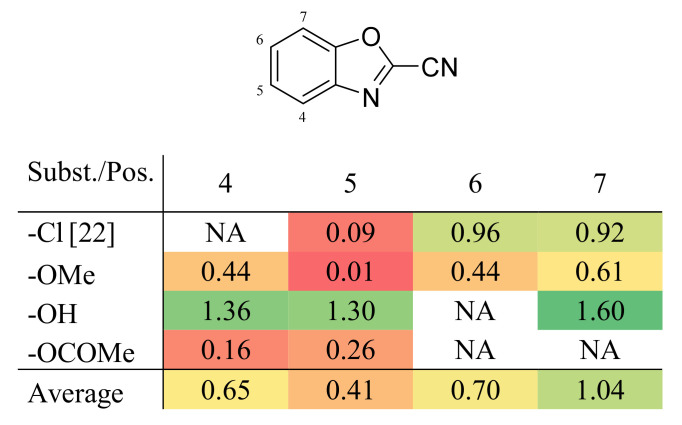
Group efficiency analysis of benzoxazoles. GE values are color-coded row-wise (green to red for large to small) according to the relative contribution to the binding free energy. GE was calculated as ΔpIC_50_/ΔN_heavy_ with compound **1** as a reference. IC_50_ = 80 µM and 5 µM was used for 5-OMe (RA = 84% at 10 µM) and 7-OMe (RA = 20% at 10 µM), respectively.

The substitution pattern had an important influence on the observed inhibitory potencies (Table 2). The most potent compounds, namely the OH-substituted (**10**, **11**, and **13**) and the 5-OCOMe derivative **14** showed single digit micromolar IC_50_ values against the β5i subunit. 4-OMe- and 6-OMe-substituted compounds (**6** and **8**, respectively) also inhibited β5i with IC_50_ values just above 10 µM. In contrast, several compounds, such as NH_2_-substituted compounds **20**, **21**, and **23** as well as the 4- and 7-NHCOPh derivatives **24** and **27**, respectively, did not inhibit β5i at 10 µM concentration (Table 2). Using the GE concept, we evaluated the effect of positions around the benzoxazole-2-carbonitrile core (Figure 2). The largest increase in GE compared to the unsubstituted benzoxazole-2-carbonitrile was observed in position 7, which can be considered the preferred vector for fragment growing, followed by positions 6 and 4. This analysis revealed that position 5 is the least preferred for most substituents.

In our previous work, we used MS/MS experiments and Ellman’s assay to investigate the mechanism of action of benzoxazole-2-carbonitriles. Our results indicated reversible covalent binding to a cysteine residue and the labeling of Cys48 of the β5i subunit was proposed based on solvent accessibilities of the cysteine residues as computed using the protein X-ray structure [22]. Therefore, we investigated the effect of substituents on the reactivity of carbonitrile warhead-containing fragment-sized inhibitors. Testing the compounds against *N*-acetyl cysteine (see protocol in the Materials and Methods section and in Figure S1) showed that they were hyperreactive, which prevented the determination of half-lives (Table S1). The reactivity of seven compounds (**5**–**7**, **9**, **22**, **25**, and **26**) was also tested against glutathione (GSH) (see protocol in the Materials and Methods section), since in an earlier study GSH had a different reactivity profile compared with *N*-acetyl cysteine [24]. The methoxy-substituted compounds (**6**, **7**, and **9**) were also hyperreactive in this assay. The reactions of the benzamides (**25** and **26**) were more traceable, but their half-life was less than 120 min. It was hypothesized that the reactivity of compounds towards *N*-acetyl cysteine or GSH depends on the charge distribution of the carbon atom of carbonitrile moiety with a more electron-deficient carbon atom having higher reactivity. Therefore, the ^13^C NMR chemical shifts of the nitrile carbon were compared for different benzoxazole-2-carbonitriles dissolved in DMSO-*d_6_* (Table S1). The chemical shifts vary in a narrow range between 109.0 and 112.4 ppm, suggesting similar electron distributions in agreement with the observed uniform reactivity of these compounds.

**Scheme 1:**
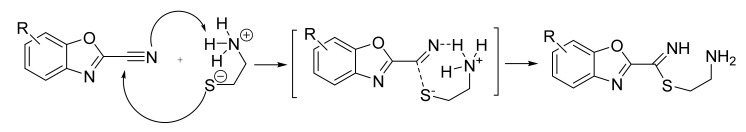
Computationally studied reaction between aromatic nitriles and cysteamine.

The reactivity of compounds was also investigated by quantum chemical calculations. The energy barrier of the reaction between benzene-substituted benzoxazole-2-carbonitriles and cysteamine (Scheme 1) was calculated with density functional theory (DFT) with the B3LYP functional using 6-311++G** basis set and an implicit water model (Table S1). The barriers of 4-substituted OMe (**6**), OH (**10**), and NH_2_ (**20**) derivatives ranged from 6.8 to 7.5 kcal/mol, whereas 7.2 kcal/mol was calculated for the unsubstituted benzoxazole-2-carbonitrile. The barriers of compounds with OMe substitution at all four available positions of the benzene ring (compounds **6**–**9**) were evaluated and ranged from 6.8 kcal/mol (4-OMe) to 8.1 kcal/mol (6-OMe). These results also show that the substitution pattern on the benzene moiety of the benzoxazole-2-carbonitrile has little effect on the carbonitrile reactivity towards thiolate. We note that these calculations are analogous to those of Berteotti and co-workers, who found a good correlation between calculated and experimental reactivities for a set of aromatic nitrile compounds [26].

Our results show that benzene ring substituents affect the β5i inhibitory potencies of benzoxazole-2-carbonitriles. However, the variations in potencies cannot be attributed to the reactivity variations measured against the model nucleophiles *N*-acetyl cysteine and GSH. As discussed above, the ^13^C NMR chemical shifts and quantum chemical barrier calculations indicate that neither substituent type nor substituent position have significant effect on reactivity. Therefore, the differences in inhibitory potencies are most likely related to the non-covalent recognition step of the binding. To gain further insight into the non-covalent recognition of benzoxazole-2-carbonitriles, we performed computational docking. Non-covalent docking was performed on the Cys48Ala mutant structure using a constraint to ensure that the carbon atom of the carbonitrile moiety is positioned near to C_β_ of Ala48. An examination of the binding poses of OH- and OMe-substituted compounds revealed that the best scoring poses formed two clusters, both occupying the binding channel, and are separated by 8–9 Å from the oxygen atom of the Thr1 side chain (Figure S2). The loose SAR, observed for benzoxazole-2-carbonitriles with small benzene substituents, is probably related to the large space available for the bound compound and to the different H-bond acceptors and donors on the surface of the binding channel. Larger benzene substituents are able to extend toward the catalytic Thr1 residue, as shown by the 4-OCOMe derivative (**14**) (Figure S3).

To assess the selectivity of benzoxazole-2-carbonitrile-based inhibitors of β5i, seven compounds were selected that demonstrated reasonable potency against the β5i subunit: **6**, **8**, **10**, **11**, and **13**–**15**. These compounds showed preferential inhibition of β5i over the β2i and β1i subunits of human iCP, as well as over the β1 and β2 subunits of human cCP (Table 3). The compounds also inhibited β5 subunit, albeit to a lesser extent in comparison to β5i. These results are in accordance with covalent binding to the Cys48 residue present in the β5i subunit, but not in all other catalytically active subunits of iCP and cCP.

**Figure 3:**
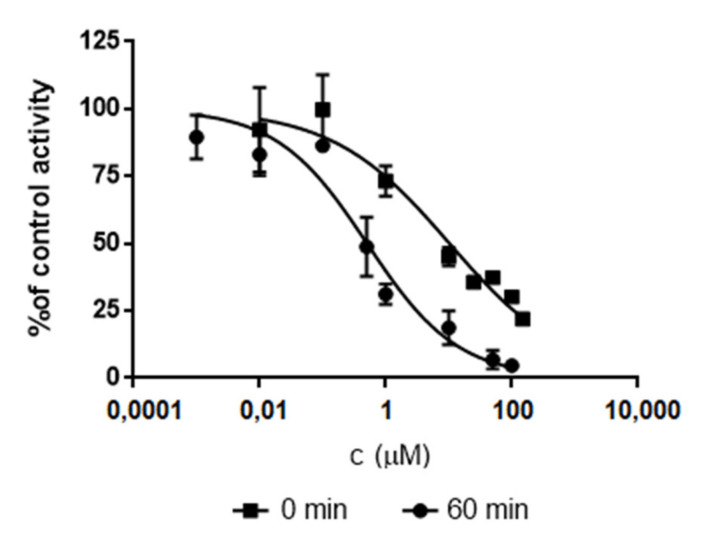
Inhibition curves (for β5i activity) without and with 60-min pre-incubation of compound **10** demonstrate IC_50_ shift.

Next, the time-dependence of β5i inhibition was demonstrated using the IC_50_ shift assay. For the 4-OH derivative **10**, a characteristic time-dependent left shift of the inhibition curve was observed. More precisely, the IC_50_ values of 10 ± 1 µM without pre-incubation and 0.50 ± 0.07 µM after 60-min pre-incubation were determined (Figure 3).

**Figure 4:**
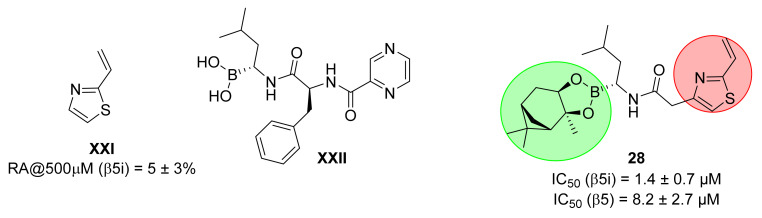
Structure of 2-vinylthiazole (**XXI**), bortezomib (**XXII**), and the designed compound **28**. The boron-bearing warhead is marked with green circle and the 2-vinylthiazole is marked with red circle.

In parallel with the synthesis and evaluation of benzoxazole-2-carbonitrile derivatives, we pursued a follow-up approach to identify iCP inhibitors. A library of heterocyclic electrophilic fragments [23] was screened in the β5i inhibition assay and 12 compounds showed RAs less than 50% at screening concentration of 500 µM (Table S2). After visual inspection, availability of derivatives, and synthetic feasibility, we selected 2-vinylthiazole (**XXI**, RA (β5i) = 5 ± 3%, Figure 4) to be incorporated into a potential covalent inhibitor containing the additional boronic acid warhead moiety similar to the (*R*)-boroleucine moiety of bortezomib (**XXII**, Figure 4). The compound was designed based on the X-ray structure of bortezomib in complex with humanized yeast proteasome [39,40], with the vinylthiazole group added at a position that could allow its access to the β5i-specific Cys48 residue. Details of the synthesis can be found in the Supplementary Materials (Scheme S5). For synthetic reasons, compound **28** contained a pinanediol ester rather than free boronic acid; however, it was demonstrated previously [30,41,42,43] that pinanediol esters show similar inhibitory potencies as boronic acids. Compound **28** was found to be unreactive with GSH in the HPLC-based reactivity assay. However, it inhibited the β5i and β5 catalytic activities with IC_50_ values of 1.4 µM and 8.2 µM, respectively 

To extend this approach to benzoxazole- and benzimidazole-2-carbonitriles, we synthesized next a set of compounds, in which the carbonitrile-bearing heterocycle was attached to the (*R*)-boroleucine via different linkers. First, we turned our attention to benzimidazole-2-carbonitriles and prepared compounds **29**–**36**, in which the two electrophilic moieties were connected with linkers of varying length (Table 4, Scheme S6). These compounds were initially evaluated for their stability in buffer solutions (pH 7.4, PBS, and in the assay buffer), followed by determination of their inhibitory potencies against the β5i subunit of human iCP and the β5 subunit of human cCP (Table 4). With the exception of **29** and **33**, all of the investigated compounds showed reasonable stability in buffer solutions. In general, boronic acids and their related pinanediol esters showed similar inhibitory potencies. Compound **30,** containing an ethylene linker, was found to be the most potent β5i inhibitor with an IC_50_ value of 0.6 µM. In a previous study [22], we found that the introduction of a chloro substituent into the benzene ring of the benzimidazole-2-carbonitrile structure resulted in a significant improvement in the inhibitory potencies. Therefore, we equipped the submicromolar inhibitor **30** (see Table 4 and Scheme S7) with a chloro substituent at positions 6 and 7. Although chloro-substituted derivatives **37** and **38** showed no improvement over their parent compound **30** in terms of β5i inhibition, **37** exhibited slight selectivity over β5 (Table 4). Compounds with longer linkers (propylene, *n*-butylene) showed a slightly reduced β5i inhibitory potencies. Interestingly, compounds with *n*-butylene linkers (**32** and **36**) were better inhibitors of the β5 subunit in comparison to the β5i subunit.

Of note, the reactivities of these compounds were also investigated against two small-molecule thiol surrogates, *N*-acetyl cysteine and GSH, and none of the benzimidazole-2-carbonitrile-containing compounds proved to be reactive in these assays (Table S3).

In the benzoxazole-2-carbonitrile subseries, two compounds (**39** and **40**) were prepared (see Scheme S8 in the Supplementary Materials for details of the synthesis). The benzoxazole-based bidentates were stable in the assay buffers and these compounds also showed similar inhibitory characteristics to benzimidazole-based bidentates (Table 5). Reactivity evaluations against *N*-acetyl cysteine using the UV-Vis-based assay (see Section 2.5.1. for details) showed that compounds **39** and **40** were hyperreactive (Table S4).

Inhibition assays of selected bidentates **28**, **30**, **31**, **34**–**36**, and **38**–**40** against the remaining subunits of both iCP and cCP showed that all compounds inhibited β1i and β1 equipotently, while β2 activities were not inhibited (Table 6). The fact that bidentates inhibited the β1 and β5 subunits of both proteasomes is not surprising since bortezomib, which contains the threonine-directed (*R*)-boroleucine moiety, is also a potent inhibitor of the same subunits [44].

In an attempt to investigate the mechanism of inhibition, the Ellman’s assay to determine the fraction of available cysteines was performed using β5i with and without incubation of the compound. However, we found that the Ellman’s assay was not feasible with bidentate compounds (**28–40**) due to significant assay interference. In contrast, intact MS/MS experiments showed single labelling of β5i by **39** (Figure S4). This confirms the covalent binding mechanism. Although the site of labelling was not investigated by MS/MS experiment of the digested labelled protein, the similar inhibitory activities toward β5 and β5i strongly suggest that **39** binds to the Thr1 residue common in the two isoforms and does not directly interact with the β5i-specific Cys48.

IC_50_ shift experiments to demonstrate time-dependent inhibition of the β5i subunit were performed for bidentates as well. Surprisingly, a very marginal time-dependence was observed for these compounds (Table S5). This could indicate either a reversible, low barrier covalent interaction or a very rapid and irreversible inactivation of enzyme that occurs almost immediately after the inhibitors are mixed with the proteasomes during the inhibition assays.

Nitriles and boronic acids can react with other proteins containing catalytic or noncatalytic nucleophilic residues amenable for covalent modification [45,46,47,48]. Inhibition of unrelated serine esterases, i.e., human acetylcholinesterase (hAChE) and butyrylcholinesterase (hBChE) with catalytic serine, human caspase-1 with catalytic cysteine, and human monoamine oxidases A and B (hMAO-A/B) with tractable cysteines at the active site [49] was investigated with selected compounds (Table S6). At a screening concentration of 100 µM, which is 10-fold higher compared to (immuno)proteasome assays, benzoxazole-2-carbonitriles show moderate inhibition of hMAO-A/B and hAChE (RAs: 11–48%) and are inactive towards hBChE. These results are expected due to the small size of the compounds and their relatively lipophilic character, which allows them reach hydrophobic active sites of the studied enzymes. On the contrary, the majority of benzoxazole-2-carbonitriles inhibited cysteine protease caspase-1. Nonetheless, the selectivity of bidentate compounds towards the (immuno)proteasome is enhanced when compared to benzoxazole-2-carbonitriles. Only marginal inhibition for bidentate compounds was determined towards both MAOs and AChE/BChE enzymes with RAs above 40%, and no inhibition of caspase-1 was observed. This implies that fragment growing can not only increase the on-target potency, but also improve the selectivity.

## 4. Conclusions

As a next step of our fragment-based approach to discover (immuno)proteasome inhibitors, we further investigated benzoxazole-2-carbonitriles as fragment-like inhibitors of the β5i subunit of iCP. A library of analogs was synthesized by systematically introducing substituents at all positions of the benzene ring. Most compounds were found to be stable in the buffer solutions and were highly reactive towards both *N*-acetyl cysteine and GSH. ^13^C NMR chemical shifts and quantum chemical reaction barrier calculations confirmed that the high reactivity is not dependent on the nature of the substituent on the benzene ring. Considering the variation in inhibitory potencies (from no inhibition at 10 µM to up to 2.1 ± 1.2 µM IC_50_) and the uniform reactivity of the compounds studied, we conclude that non-covalent recognition of binding is responsible for the variations in inhibitory potency. This emphasizes the importance of non-covalent recognition patterns in the binding of fragment-sized covalent inhibitors and has relevance for drug discovery programs using a fragment-based covalent approach. Computational docking of the molecules suggested that multiple binding poses and several H-bonding interactions are plausible for the substituted benzoxazole-2-carbonitriles binding to Cys48. The time-dependent inhibition of the β5i subunit by the 4-OH derivative **10** and the selectivity of this class of compounds for the β5i in comparison to other catalytically active subunits of iCP and cCP that do not contain the Cys48 residue suggest the covalent inhibition mechanism. The most potent benzoxazole-2-carbonitriles identified, i.e., compounds **10** and **13**, could be further transferred to a hit optimization campaign to develop non-peptide, selective, covalent β5i inhibitors. Selective inhibition of the β5i subunit might have great potential for the treatment of both non-cancer diseases related to the iCP [50] and malignant diseases [14].

As a continuation of the first approach, we developed bidentate compounds in which we combined two different electrophilic moieties into a single molecule. All derivatives were evaluated in stability and reactivity assays, and against all catalytically active subunits of human iCP and cCP. No discrimination between proteasomes was observed, as most bidentate compounds inhibited the β1 and β5 subunits of both cCP and iCP in the sub-micromolar to low micromolar range. We realize that further medicinal chemistry endeavours are needed to increase noncovalent recognition, which would lead to an increase in (immuno)proteasome inhibition. However, the fact that most bidentates do not inhibit unrelated enzymes makes this novel class of compounds a valuable starting point for hit-to-lead optimization of inhibitors targeting multiple subunits of both proteasomes. If successfully optimized, such pan-proteasome inhibitors could prove useful as anticancer drugs [51].

## Figures and Tables

**Table 1:** Inhibitory potencies of benzoxazole-2-carbonitrile and chloro derivatives against the β5i subunit of human iCP.

Substituent	Position	Label	IC_50_ (µM) ^a^
-	-	**1**	83 ± 6.0
-Cl	5	**2**	67 ± 11
	6	**3**	9.1 ± 4.5
	7	**4**	10 ± 4.6

^a^ IC_50_ values from ref [22]; compounds showed limited stability after 1 h in the assay buffer.

**Table 2:** Inhibitory potencies of benzoxazole-2-carbonitriles against the β5i subunit of human iCP. The RA values were determined at 10 µM of compounds and 30 min pre-incubation. PR-957 was used as a positive control and its IC_50_ value for inhibition of β5i was 0.022 ± 0.009 µM.

Substituent	Position	Cpd	Stability in Buffer	RA (%) at 10 µM or IC_50_ (µM) ^a^
-Me ^b^	6	**5**	low absorbance ^c^/stable ^d^	81 ± 4%
-OMe	4	**6**	stable ^c,d^	11 ± 7 µM
	5	**7**	stable ^c,d^	84 ± 11%
	6	**8**	intermediate ^c^/stable ^d^	11 ± 6 µM ^e^
	7	**9**	stable ^c,d^	20 ± 6% ^f^
-OH	4	**10**	stable ^c^	3.6 ± 2.8 µM
	5	**11**	stable ^c^	4.2 ± 2.5 µM
	6	**12**	intermediate ^c^	ND ^g^
	7	**13**	stable ^c^	2.1 ± 1.2 µM
-OCOMe	4	**14**	stable ^c^	19 ± 9 µM
	5	**15**	stable ^c^	7.4 ± 1.7 µM
-NO_2_	4	**16**	intermediate^c^/unstable ^d^	NA ^h^
	5	**17**	NA ^i^	NA ^i^
	6	**18**	unstable ^c^	NA ^h^
	7	**19**	stable ^c^	82 ± 2%
-NH_2_	4	**20**	stable ^c^	94 ± 7%
	5	**21**	stable ^c^	81 ± 3%
	6	**22**	stable ^c,d^	ND ^g^
	7	**23**	stable ^c^	98 ± 4%
-NHCOPh	4	**24**	stable ^c^	74 ± 17%
	5	**25**	stable ^c,d^	ND ^g^
	6	**26**	unstable ^c^/stable ^d^	NA ^h^
	7	**27**	stable ^c^	103 ± 8%

^a^ IC_50_ and RA are means from at least three independent determinations.^b^ 4-, 5-, and 7-Me substituted compounds were unstable in solid form. ^c^ Stability assessment after 1 h using UV-Vis-based assay. Stability was not determined for compounds with low absorbance. ^d^ Stability assessment with HPLC-based assay. ^e^ This value should be interpreted with caution due to the intermediate stability of the compound. ^f^ The lack of concentration dependence prevented proper IC_50_ determination. ^g^ High fluorescence background precluded reliable measurements (interference with assay readout). ^h^ Not determined due to instability in the assay buffer. ^i^ Not determined due to insolubility in the assay buffer.

**Table 3:** Inhibitory potencies of selected compounds against other subunits (β2i and β1i) of the human iCP and against all subunits (β5, β2, β1) of the human cCP. For β1i, β1, β2i, β2, and β5, we measured RAs in the presence of 10 µM of each compound. IC_50_ values were determined only for compounds that showed notable inhibition at 10 µM (RA < 60%). For β5, carfilzomib was used as a control and exhibited RAs of 25 ± 1% at 10 nM. Bortezomib was used as a control for β2i, β1i, β2, and β1, and exhibited RAs (all at 10 µM) of 16 ± 5%, 0%, 62 ± 9%, and 0%, respectively. Inhibitory activities of compounds against subunits β5 and β5i were measured using SDS as the proteasome activator, whereas for β1, βi, β2, and β2i activities, PA28α was used.

Cpd	Residual Activity or IC_50_ (µM) According to CP, Subunit, and Substrate					
	iCP			cCP		
	β5i (IC_50_ [μM]) ^a^	β2i (RA [%]) ^a^	β1i (IC_50_ [μM]) ^a^	β5 (RA [%] or IC_50_ [μM]) ^a^	β2 (RA [%]) ^a^	β1 (RA [%]) ^a^
	Suc-LLVY-AMC	Boc-LRR-AMC	Ac-PAL-AMC	Suc-LLVY-AMC	Boc-LRR-AMC	Ac-nLPnLD-AMC
**6**	11 ± 7	NA	78	63% ^c^	NA	82%
**8**	11 ± 6 ^b^	NA	69	74% ^b^	NA	NA
**10**	3.6 ± 2.8	NA	NA	30 ± 12	NA	NA
**11**	4.2 ± 2.5	NA	NA	53% ^c^	NA	NA
**13**	2.1 ± 1.2	83%	NA	11 ± 9	71%	76%
**14**	19 ± 9	NA	NA	56% ^c^	NA	NA
**15**	7.4 ± 1.7	NA	NA	55% ^c^	NA	NA

^a^ IC_50_ and RA are means from at least three independent determinations. Standard errors for RAs were <15%. ^b^ This value should be interpreted with caution due to intermediate stability of compound. ^c^ IC_50_ was not determined due to limited solubility that precluded the preparation of solution with higher than 150 µM concentration. NA, Not active (RA above 90%). Suc-LLVY-AMC, succinyl-Leu-Leu-Val-Tyr-7-amino-4-methylcoumarin; Boc-LRR-AMC, *t*-butyloxycarbonyl-Leu-Arg-Arg-7-amino-4-methylcoumarin; Ac-PAL-AMC, acetyl-Pro-Ala-Leu-7-amino-4-methylcoumarin; Ac-nLPnLD-AMC, acetyl-Nle-Pro-Nle-Asp-AMC.

**Table 4:** Inhibitory potencies of benzimidazole-2-carbonitrile-containing boronic acid derivatives against the β5i subunit of human iCP and the β5 subunit of human cCP. The RA (for β5i and β5) values were determined at 10 µM compound concentration and 30 min pre-incubation.

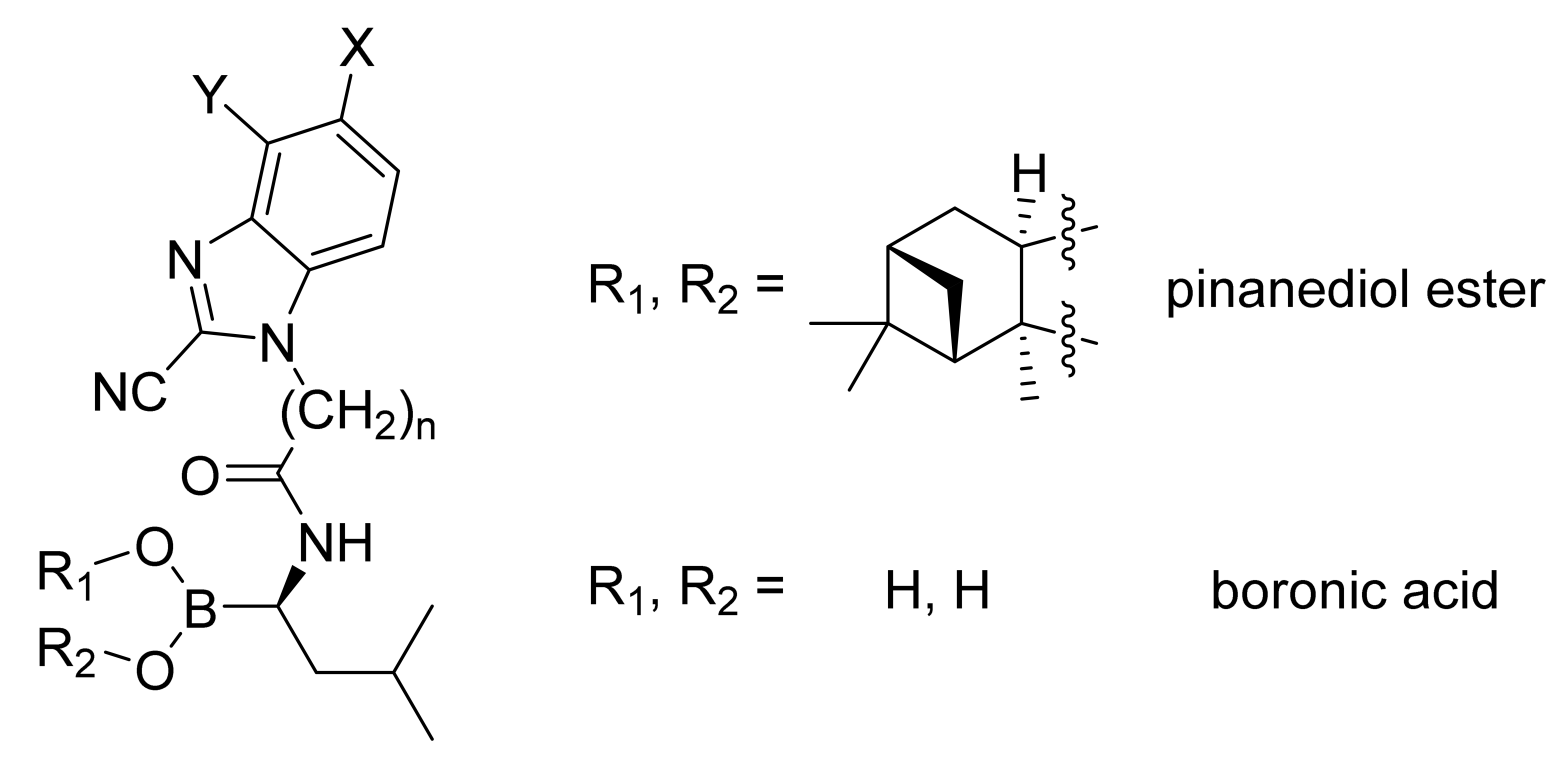							
n	X	Y	R_1_, R_2_	Cpd	Stability in Buffer	RA (%) at 10 µM or IC_50_ (µM) ^a^	
						β5i	β5
1	-	-	pinanediol ester	**29**	unstable ^b,c^	ND ^d^	ND ^d^
2	-	-	pinanediol ester	**30**	stable ^b,c^	0.6 ± 0.0 µM	0.7 ± 0.1 µM
3	-	-	pinanediol ester	**31**	stable ^b,c^	5.7 ± 0.9 µM	13 ± 4 µM
4	-	-	pinanediol ester	**32**	intermediate ^b^/stable ^c^	13 ± 2 µM ^e^	1.6 ± 0.8 µM ^e^
1	-	-	boronic acid	**33**	unstable ^b,c^	ND ^d^	ND ^d^
2	-	-	boronic acid	**34**	low absorbance ^b^	2.2 ± 0.6 µM	1.8 ± 0.4 µM
3	-	-	boronic acid	**35**	stable ^b^	7.9 ± 0.5 µM	16 ± 8 µM
4	-	-	boronic acid	**36**	stable ^b^	59 ± 7 µM	6.4 ± 2.6 µM
2	-Cl	-	pinanediol ester	**37**	intermediate ^b^/stable ^c^	1.6 ± 1.3 µM ^e^	14 ± 1% ^e^
2	-	-Cl	pinanediol ester	**38**	stable ^b,c^	0.6 ± 0.1 µM	2.0 ± 0.7 µM

^a^ IC_50_ and RA are means from at least three independent determinations. ^b^ Stability assessment after 1 h with UV-Vis-based assay. Stability was not determined for compounds with low absorbance. ^c^ Stability assessment with HPLC-based assay. ^d^ Not determined due to instability in the assay buffer. ^e^ This value should be interpreted with caution due to intermediate stability of compound.

**Table 5:** Inhibitory potencies of benzoxazole-2-carbonitriles combined with boronic acid derivatives against the β5i subunit of human iCP and the β5 subunit of human cCP. The RA (for β5i and β5) values were determined at 10 µM of the compounds and 30 min pre-incubation.

Structure	Cpd	Stability in Buffer	IC_50_ (µM) ^a^	
			β5i	β5
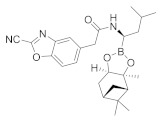	**39**	stable ^b,c^	3.1 ± 0.3 µM	3.6 ± 1.1 µM
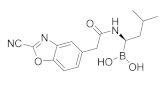	**40**	stable ^b^	2.4 ± 1.0 µM	2.0 ± 0.7 µM

^a^ IC_50_ values are means from at least three independent determinations. ^b^ Stability assessment with UV-Vis-based assay. Stability was not determined for compounds with low absorbance. ^c^ Stability assessment with HPLC-based assay.

**Table 6:** Inhibitory potencies of selected bidentate compounds against β2i and β1i subunits of human iCP and against β2 and β1 subunits of the human cCP. The IC_50_ values were determined for compounds that showed notable inhibition at 10 µM (RA < 60%). For β2i and β2, we measured RAs in the presence of 10 µM of each compound. The same control compound, i.e., bortezomib, was used as indicated in Table 3. Inhibitory activities of compounds against subunits β1, βi, β2, and β2i were measured using PA28α as the proteasome activator.

Cpd	Residual Activity or IC_50_ (µM) According to CP, Subunit, and Substrate			
	iCP		cCP	
	β2i (RA [%]) ^a^	β1i (IC_50_ [μM]) ^a^	β2 (RA [%]) ^a^	β1 (IC_50_ [μM]) ^a^
	Boc-LRR-AMC	Ac-PAL-AMC	Boc-LRR-AMC	Ac-nLPnLD-AMC
**28**	NA	2.2 ± 0.1	NA	4.8 ± 0.2
**30**	NA	1.2 ± 0.1	NA	0.4 ± 0.0
**31**	NA	6.0 ± 3.2	NA	8.4 ± 1.6
**34**	NA	2.8 ± 0.5	NA	3.2 ± 0.2
**35**	NA	9.4 ± 1.9	NA	13 ± 2
**36**	NA	4.9 ± 0.4	NA	23 ± 6
**38**	NA	1.9 ± 0.0	NA	5.0 ± 0.1
**39**	NA	1.3 ± 0.0	NA	2.9 ± 0.4
**40**	NA	2.1 ± 0.1	NA	3.7 ± 0.9

^a^ IC_50_ and RA are means from at least three independent determinations. Standard errors for RAs were <15%. NA, Not active (RA above 95%).

## Data Availability

Data are available upon request.

## Supplementary Materials

The following are available online at https://www.mdpi.com/article/10.3390/cells10123431/s1; Scheme S1: Preparation of 2-aminobenzene-1,4-diol (**II**) and 3-aminobenzene-1,2-diol (**VII**), Scheme S2: General methods for the synthesis of benzoxazole-2-carbonitriles using Appel’s salt, Scheme S3: Alternative method of the synthesis of benzoxazole-2-carbonitriles, Scheme S4: Synthesis of further benzoxazole-2-carbonitrile derivatives, Scheme S5: Synthesis of compound **28**, Scheme S6: Synthesis of benzimidazole-2-carbonitrile containing boronic acid derivatives, Scheme S7: Synthesis of chloro-substituted derivatives of **30**, Scheme S8: Synthesis of benzoxazole-2-carbonitrile containing boronic acid derivative, Table S1: Reactivity of benzoxazole-2-carbonitrile derivatives, Table S2: Results of electrophilic heterocyclic library screening, Table S3: Reactivity of benzimidazole-2-carbonitrile-containing boronic acid derivatives, Table S4: Reactivity of other boronic acid derivatives, Table S5: Determination of the IC_50_ shift for selected bidentate compounds for the inhibition of β5i activity, Table S6: Inhibitory potencies of selected benzoxazole-2-carbonitriles and bidentate ligands against hAChE, hBChE and MAO-A/B, Figure S1: Absorption spectra from the UV-Vis-based stability and reactivity assays, Figure S2: Binding poses of OH- and OMe-substituted benoxazole-2-carbonitriles form two clusters both separated by 8–9 Å from the catalytic Thr1, Figure S3: Putative binding pose of 4-OCOMe-substituted benzoxazole-2-carbonitrile **14**. The substituent at position 4 extends toward the catalytic Thr1 residue, Figure S4: Deconvoluted mass spectrum of reference β5i (PSMB8 Human) (marked with blue) and β5i modified by **39** (449 Da) (marked with magenta). Supplementary Material Section 1: Details of chemical syntheses, Supplementary Material Section 2: Detailed synthetic procedures for the preparation of all compounds and their spectroscopic and analytical data, Supplementary Material Section 3: LC-MS chromatograms and spectra, ^1^H NMR and ^13^C NMR spectra of the synthesized compounds.

## Abbreviations

AChE: acetylcholinesterase; AUC: area under the curve; BChE: butyrylcholinesterase; cCP: constitutive proteasome; CP: core particle; DFT: density functional theory; GE: group efficiency; GSH: glutathione; HRMS: high-resolution mass spectrometry; iCP: immunoproteasome; IRC: intrinsic reaction coordinate; MAO: monoamine oxidase; PBS: phosphate buffered saline; SAR: structure–activity relationship; UPS: ubiquitin–proteasome system.
