# Supplementary material for: Fragment-Sized and Bidentate (Immuno)Proteasome Inhibitors Derived from Cysteine and Threonine Targeting Warheads
Source: Cells. 2021 Dec 6;10(12):3431. doi: 10.3390/cells10123431 (PMC8700061; doi:10.3390/cells10123431)
Supplement: Supplementary file 1 [file cells-10-03431-s001.zip › cells-1461843-supplementary.pdf]

SUPPORTING INFORMATION

Fragment-sized and bidentate (immuno)proteasome inhibitors derived from cysteine and threonine targeting warheads

Contents of Supporting Information:

1. Details of chemical syntheses 1

1.1. Preparation of benzoxazole-2-carbonitriles 1

1.2. Preparation of vinylthiazole-linked boronic acid derivative 4

1.3. Preparation of benzimidazole-2-carbonitrile-linked boronic acids 5

1.4. Preparation of chloro-substituted benzimidazole-2-carbonitrile-linked boronic acids 6

1.5. Preparation of benzoxazole-2-carbonitrile-linked boronic acids 7

2. Synthesized compounds 8

2.1. Synthesis of intermediates for the benzoxazole-2-carbonitrile library 8

2.2. Synthesis of benzoxazole-2-carbonitriles 10

2.3. Synthesis of boronic acid derivatives 17

3. LC-MS chromatograms, ^{1}H NMR and ^{13}C NMR spectra of the synthesized compounds 34

4. Supplementary Tables and Figures 121

References 134

1. Details of chemical syntheses

1.1. Preparation of benzoxazole-2-carbonitriles

Appropriately substituted 2-aminophenol derivatives were used as synthetic starting materials. Most of them were commercially available, while 2-aminobenzene-1,4-diol (**II**) and 3-aminobenzene-1,2-diol (**VI**) were prepared in-house (Scheme S1). 2-Nitrobenzene-1,4-diol (**I**) was hydrogenated using Selcat-Q6 (10% Pd/C) as catalyst at room temperature and 8 bar hydrogen pressure to achieve complete conversion in 2 hours. 2-Aminobenzene-1,4-diol (**II**) was isolated after filtration and evaporation of the volatiles. 3-Aminobenzene-1,2-diol (**VI**) was prepared in two steps. Catechol (**III**) was first nitrated with fuming HNO_3 at 0 °C according to Cozza et al. [1]. The desired compound **IV** was formed as the minor product since the formation of 4-nitrobenzene-1,2-diol (**V**) prevailed. Interestingly, the formation of a small amount of dinitro-catechol (**VI**) could also be observed. After crystallization workup, 3-nitrobenzene-1,2-diol (**IV**) was obtained, which was hydrogenated as for the preparation of **II**. Complete conversion was achieved in 4 hours, whereby atmospheric hydrogen pressure was sufficient to give the desired 3-aminobenzene-1,2-diol (**VII**).

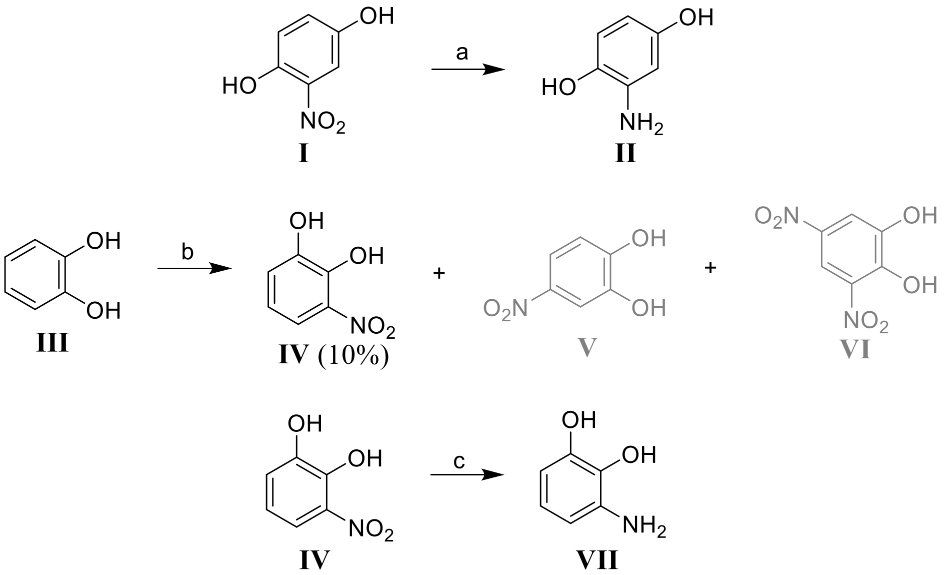

Scheme S1. Preparation of 2-aminobenzene-1,4-diol (**II**) and 3-aminobenzene-1,2-diol (**VII**).[a] [a]Reagents and conditions: (a) H_2/Pd/C, EtOH, RT, 8 bar, 2 h, 99%; (b) 90% HNO_3, Et_2O, 0 °C, 2 h to RT, 16 h, 10%; (c) H_2/Pd/C, MeOH, RT, 1 bar, 4 h, 98%

For the synthesis of benzoxazole-2-carbonitriles, the general synthetic method was very similar to that reported previously [2], *i.e.*, ring closure and incorporation of the nitrile warhead was achieved by 4,5-dichloro-1,2,3-dithiazolium chloride (Appel's salt) (Scheme S2).

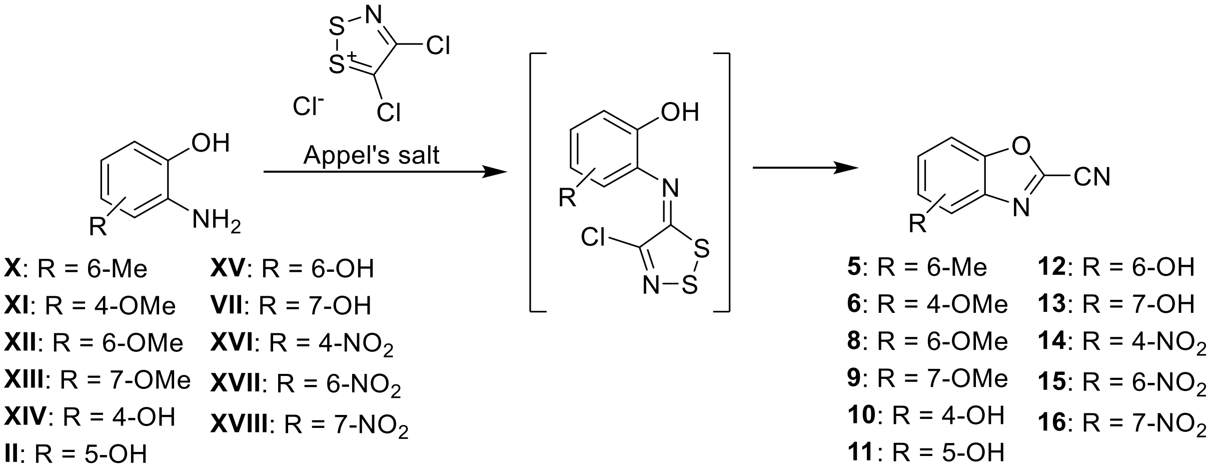

Scheme S2. General methods for the synthesis of benzoxazole-2-carbonitriles using Appel's salt.[a]
[a]Reagents and conditions: Method A: 1. pyridine, RT, 1 h; 2. 100 °C, 1–3 h, 21% for **1**, 59% for **2**, 14% for **8**, 53% for **9**, 34% for **16**, 56% for **18**, 30% for **19**; Method B: 1. THF, RT, 1 h; 2. DMSO, 100 °C, 1 h, 16% for **10**, 5% for **11**, 19% for **13**; Method C: THF, 140 °C, 20 min (MW), 5% for **12**.

The corresponding 2-aminophenol derivative and Appel's salt were stirred in pyridine at room temperature for 1 h to form the intermediate and then the mixture was heated to 100 °C for 1–3 h, depending on the starting compound (Method A, see main text).

This general method A was used in the majority of cases. Nevertheless, in some cases, these conditions resulted in a significant amount of by-products and inability to adequately purify the final products. Alternatively, the intermediate was formed in THF, and then evaporated, dissolved in DMSO and stirred at 100 °C for 1 h (Method B, see main text). Despite rather poor yields, this method was useful for the synthesis of hydroxy-substituted benzoxazole-2-carbonitriles (**10**, **11**, and **13**). 6-Hydroxy-substituted benzoxazole-2-carbonitrile (**12**) was synthesized according to Woodrofe et al. [3]. The reaction was carried-out in THF under microwave irradiation at 140 °C for 20 min. (Method C, see main text).

The above methods were effective to produce most of the 2-carbonitrile-substituted benzoxazoles. However, we could not synthesize the 5-methoxy derivative (**7**) and the 5-nitro derivative (**17**) using Appel's salt, so an alternative synthetic procedure was required. This synthetic route was inspired by Möller [4]. The corresponding 2-aminophenol derivatives were converted to 1,3-benzoxazole-2-thiols using CS_2 and KOH in an EtOH-H_2O mixture. Thiols (**VIII**, **IX**) were refluxed in thionyl chloride in the presence of DMF as catalyst to afford 2-chloro-substituted benzoxazoles in 1 h. The latter were subjected to an aromatic nucleophilic substitution reaction with KCN, requiring different temperatures and reaction times were needed, depending on the chloro-derivative (Scheme S3).

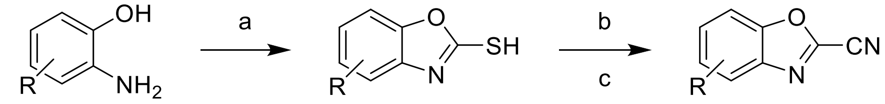

XIX: R = 5-OMe
XX: R = 5-NO_2

VIII: R = 5-OMe
IX: R = 5-NO_2

7: R = 5-OMe
17: R = 5-NO_2

Scheme S3. Alternative method of the synthesis of benzoxazole-2-carbonitriles[a]

[a]Reagents and conditions: (a) CS_2, KOH, EtOH/H_2O, reflux, 4–7 h, 83% for **VIII**, 63% for **IX**; (b) $SOCl_2$, cat. DMF, reflux, 1 h; (c) KCN, DMF, various temperatures and reaction times, 41% for **7**, 63% for **17** (yields after two consecutive steps).

The preparation of methyl-substituted benzoxazole-2-carbonitriles led to unexpected problems. While the 6-methyl derivative **5** was synthesized according to Method A without a problem, the 4-methyl- and 5-methyl-substituted benzoxazoles could not be isolated. Several methods were used for these syntheses, and the formation of these compounds was observed in reasonable amounts in the reaction mixtures. However, a considerable part of the products was lost during the workup and, moreover, we observed their significant decomposition within one hour, even at –78 °C. In the case of the 7-methyl derivative, the situation was similar, with an even higher decomposition rate that started during workup process.

Once the hydroxy- (**10–13**) and the nitro-substituted products (**16–19**) were available, further analogues were prepared from them to expand our library (Scheme S4). Compounds **10** and **11** were converted to *O*-acetyl derivatives **14** and **15**, respectively, using acetic anhydride in MeCN and concentrated H_2SO_4 served as catalyst.

The nitro-compounds were reduced with tin(II)chloride dihydrate in EtOAc with reactions proceeding to completion at 70 °C for 2 h. The obtained amines (**20**, **21**, **22**, and **23**) were acylated with benzoic anhydride in Et_3N-DMF solution mixture and a catalytic amount of DMAP to yield compounds **24**-**27**.

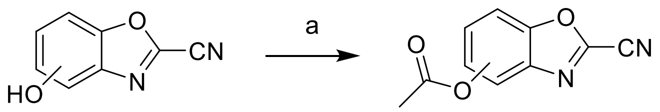

10: 4-OH
11: 5-OH

14: 4-OCOMe
15: 5-OCOMe

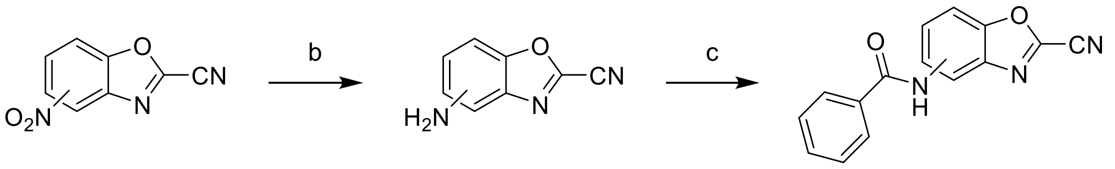

16: 4-NO_2
17: 5-NO_2
18: 6-NO_2
19: 7-NO_2

20: 4-NH_2
21: 5-NH_2
22: 6-NH_2
23: 7-NH_2

24: 4-NHCOPh
25: 5-NHCOPh
26: 6-NHCOPh
27: 7-NHCOPh

Scheme S4. Synthesis of further benzoxazole-2-carbonitrile derivatives[a]

[a]Reagents and conditions: (a) Ac_2O, conc. H_2SO_4, MeCN, 60 °C, 1 h, 24% for **14**, 25% for **15**; (b) $SnCl_2$ × $2H_2O$, EtOAc, 70 °C, 2 h, 52% for **20**, 63% for **21**, 66% for **22**, 68% for **23**; (c) $(PhCO)_2O$, DMAP, Et_3N, DMF, various temperatures and reaction times, 41% for **24**, 68% for **25**, 55% for **26**, 60% for **27**.

1.2. Preparation of vinylthiazole-linked boronic acid derivative

First, 2-(2-bromothiazol-4-yl)acetic acid (**XXIII**) was reacted with thionyl chloride in MeOH to give the methyl ester **XXIV** in excellent yield. The bromine of the ester was converted to vinyl in a reaction with tributyl(vinyl)tin to give the compound **XXV** in moderate yield. Subsequently, the ester group was hydrolyzed in the presence of NaOH to afford the carboxylic acid **XXVI**, which could acylate (*R*)-BoroLeu-(+)-pinanediol trifluoroacetate in the presence of HATU, leading to compound **28** (Scheme S5).

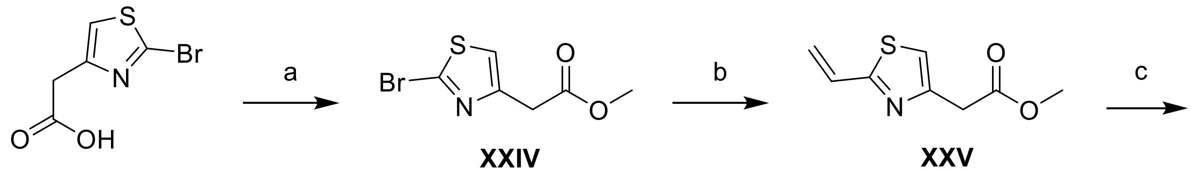

XXIII **XXIV** **XXV**

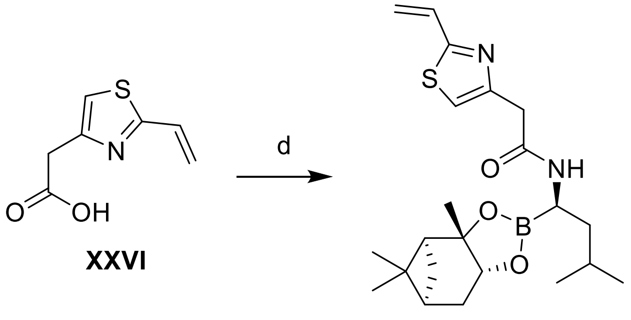

XXVI **28**

Scheme S5. Synthesis of compound **28**[a]

[a]Reagents and conditions: (a) $SOCl_2$, MeOH, RT, overnight, 95%, (b) Bu_3(vinyl)Sn, $Pd(PPh_3)_2Cl_2$, THF, 100 °C, 2 h, 40%, (c) NaOH, MeOH-H_2O, RT, 5 h, 99%, (d) (*R*)-BoroLeu-(+)-pinanediol trifluoroacetate, HATU, DIPEA, DMF, RT, overnight, 9%.

1.3. Preparation of benzimidazole-2-carbonitrile-linked boronic acids

The synthetic strategy was to attach the boronic acid moiety to the nitrogen atom of benzimidazole-2-carbonitrile via an alkyl linker (Scheme S6). The molecules were synthesized with methylene, ethylene, propylene and *n*-butylene linkers. First, benzimidazole-2-carbonitrile had to be synthesized as a key intermediate. Therefore, *o*-phenylenediamine (**XXVII**) was reacted with methyl 2,2,2-trichloroacetimidate to give the 2-trichlomethyl-benzimidazole (**XXVIII**), which was stirred in 7 M NH_3 solution in MeOH to obtain the carbonitrile derivative **XXIX**. This compound was *N*-alkylated with four different ω-bromoalkyl esters to introduce the linker. In all cases DMF was used as a solvent and DIPEA served as base. However, different temperatures and reaction times were required to achieve complete conversions (see data alongside the characterization of the intermediates below). The resulting *N*-alkylated compounds were not isolated but immediately subjected to ester hydrolysis in a one-pot reaction. Depending on the substrate, LiOH × H_2O or K_2CO_3 was used for the hydrolysis. In general, two competing reactions took place: the desired hydrolysis of the ester functional group and the undesired hydrolysis of the carbonitrile functional group. The latter was the main reason for the low yields. The resulting free carboxylic acids (**XXX**, **XXXI**, **XXXII** and **XXXIII**) were activated with HATU and coupled to (*R*)-BoroLeu-(+)-pinanediol trifluoroacetate with good yields. Three different methods were used to deprotect the boronic acids. Compound **33** was obtained by an oxidative cleavage using $NaIO_4$ and NH_4OAc in an acetone-H_2O solvent mixture, compounds **34** and **36** were obtained by a biphasic transesterification reaction using isobutylboronic acid in a MeOH-HCl-*n*-hexane solvent mixture, while **35** was prepared by a different transesterification method. Namely, it was reacted with boric acid in a $(iPr)_2O$-HCl solvent mixture (Scheme S6).

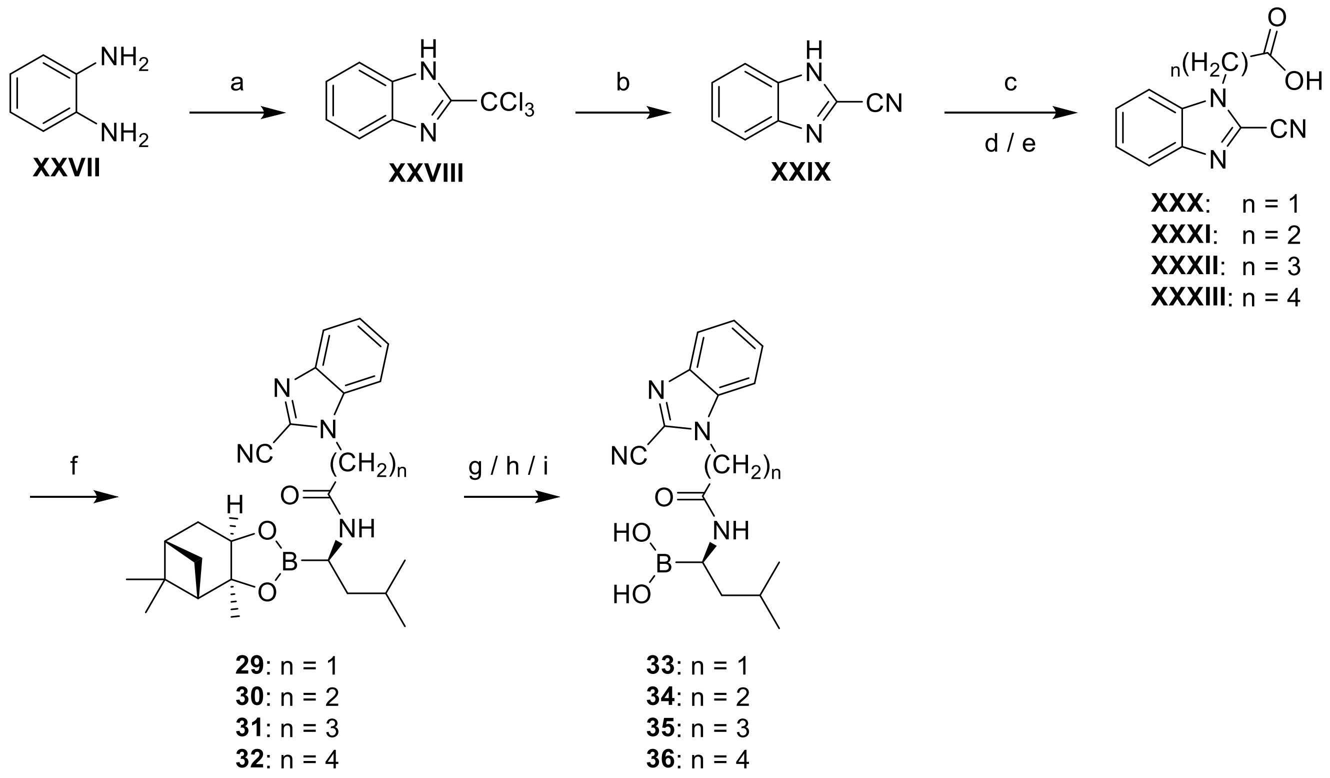

Scheme S6. Synthesis of benzimidazole-2-carbonitrile containing boronic acid derivatives[a]

[a]Reagents and conditions: (a) methyl 2,2,2-trichloroacetimidate, AcOH, RT, 1 h, 88%; (b) NH_3, MeOH, RT, 4 h, 60%; (c) various ω-bromoalkyl esters, DIPEA, DMF, various temperatures and reaction times; (d) LiOH × H_2O, DMF/THF/H_2O, RT, 4 h, 49% for **XXX**; (e) K_2CO_3, DMF/THF/H_2O, RT, 16 h, 24% for **XXXI**, 25% for **XXXII**, 28% for **XXXIII** (yields of two consecutive steps); (f) (*R*)-BoroLeu-(+)-pinanediol trifluoroacetate, HATU, DIPEA, DMF, RT, 2 h, 80% for **29**, 72% for **30**, 82% for **31**, 83% for **32**; (g) $NaIO_4$, NH_4OAc, acetone/H_2O, RT, 16 h, 49% for **33**; (h) isobutylboronic acid, MeOH/HCl/*n*-hexane, RT, 16 h, 51% for **34**, 63% for **36**; (i) $B(OH)_3$, $(iPr)_2O$/HCl, RT, 16 h, 43% for **35**.

1.4. Preparation of chloro-substituted benzimidazole-2-carbonitrile-linked boronic acids

As starting materials, appropriately chloro-substituted *o*-fluoronitrobenzenes [4-chloro-2-fluoronitrobenzene (**XXXIV**) and 1-chloro-2-fluoro-3-nitrobenzene (**XXXV**)] were used (Scheme S7). Aromatic nucleophilic substitution reactions were carried-out with ethyl 3-aminopropanoate hydrochloride and DIPEA was used to release the amine and neutralize the HF from the reaction. Hydrolysis of the ethyl esters was carried out in a one-pot reaction with NaOH in a DMF/THF/H_2O solvent mixture at 70 °C. Acids **XXXVI** and **XXXVII** were coupled to (*R*)-BoroLeu-(+)-pinanediol trifluoroacetate using the same method as mentioned previously. Then, the reduction of the nitro group was carried out with $SnCl_2$ × $2H_2O$ in EtOAc, and the reaction proceeded overnight at 70 °C to achieve complete conversion. To obtain the designed carbonitriles **37** and **38**, diamines **XL** and **XLI** were reacted with Appel's salt in pyridine (Scheme S7). Interestingly, the formation of **37** was significantly faster than that of **38**.

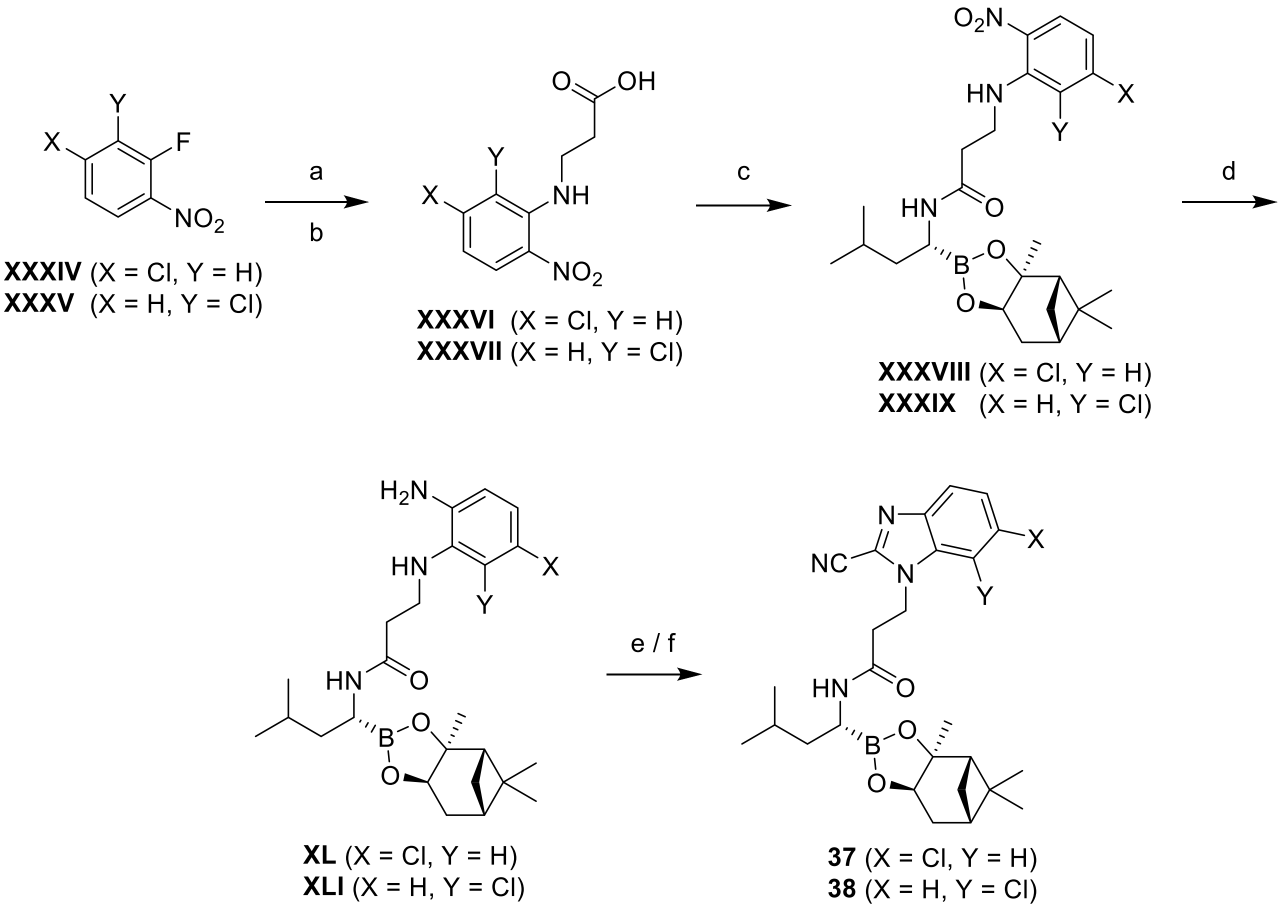

Scheme S7. Synthesis of chloro-substituted derivatives of **30**[a]

[a]Reagents and conditions: (a) ethyl 3-aminopropanoate hydrochloride, DIPEA, DMF, RT, 16 h; (b) NaOH, DMF/THF/H_2O, 70 °C, 2 h, 88% for **XXXVI**, 74% for **XXXVII** (yields of two consecutive steps); (c) (*R*)-BoroLeu-(+)-pinanediol trifluoroacetate, HATU, DIPEA, DMF, RT, 2 h, 92% for **XXXVIII**, 84% for **XXXIX**; (d) $SnCl_2$ × $2H_2O$, EtOAc, 70 °C, 16 h, 84% for **XL**, 88% for **XLI**; (e) Appel's salt, pyridine, RT, 1 h, then 50 °C, 3 h, 42% for **37**; (f) Appel's salt, pyridine, RT, 1 h, then 70 °C, 6 h, 34% for **38**.

1.5. Preparation of benzoxazole-2-carbonitrile-linked boronic acids

Two compounds were synthesized with linkers of different lengths connecting the Thr and Cys targeting units. The synthetic routes are shown in Schemes S8 and S9. Alkaline hydrolysis of methyl 2-(3-amino-4-hydroxyphenyl)acetate (**XLII**) led to a large number of side products, whereas acid-catalyzed hydrolysis gave much better results. The hydrochloride salt of 2-(3-amino-4-hydroxyphenyl)acetic acid (**XLIII**) was obtained almost in quantitative yield. It was then coupled with (*R*)-BoroLeu-(+)-pinanediol trifluoroacetate by the usual method. The amide **XLIV** was converted to the benzoxazole-2-carbonitrile derivative **39** in a reaction with Appel's salt (Scheme S8). Hydrolysis of the pinanediol ester was carried out with the biphasic transesterification reaction to give the boronic acid **40**.

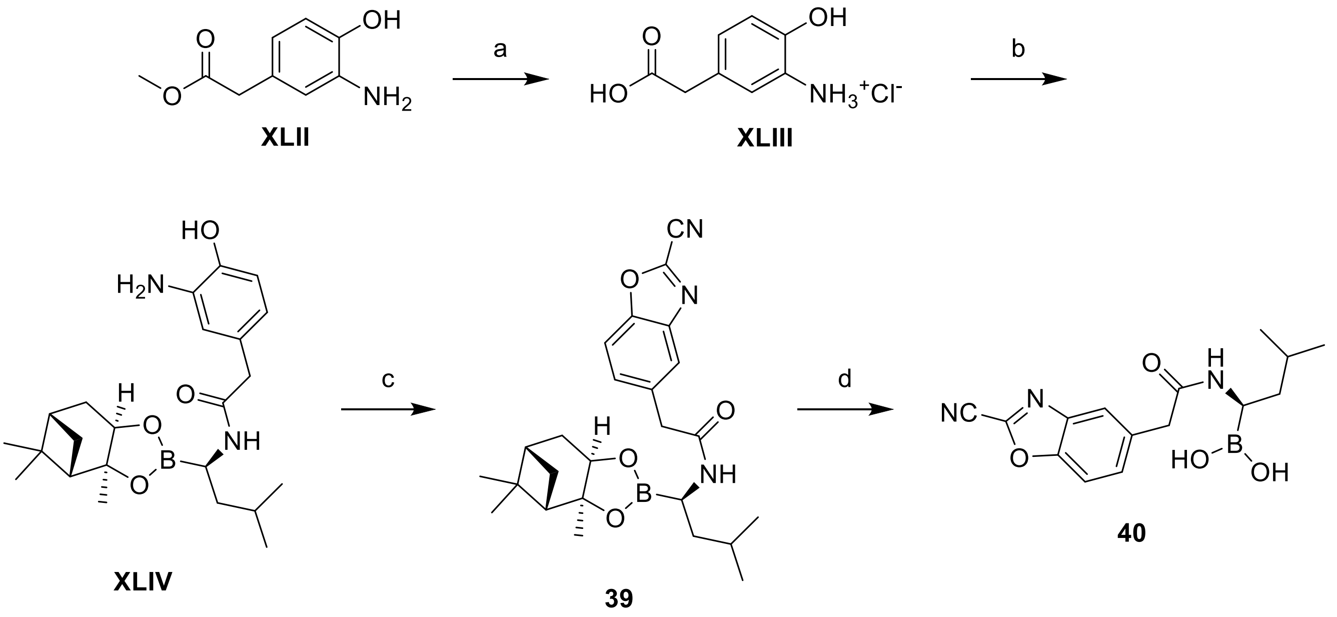

Scheme S8. Synthesis of benzoxazole-2-carbonitrile containing boronic acid derivative[a]

[a]Reagents and conditions: (a) 20% HCl, reflux, 3 h, 98%; (b) (*R*)-BoroLeu-(+)-pinanediol trifluoroacetate, HATU, DIPEA, DMF, RT, 2 h, 51%; (c) Appel's salt, pyridine, RT, 1 h, then 90 °C, 1 h, 23%; (d) isobutylboronic acid, MeOH/HCl/*n*-hexane, RT, 16 h, 80%.

2. Synthesized compounds

2.1. Synthesis of intermediates for the benzoxazole-2-carbonitrile library

2-Aminobenzene-1,4-diol (II)

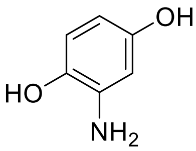

To a solution of 2-nitrobenzene-1,4-diol (**I**, 264 mg, 1.70 mmol) in EtOH (20 mL), 10% Pd/C (Selcat Q6, 26 mg) was added. The reactor was filled with H_2 (8 bar) and the mixture was allowed to stir at room temperature for 2 h. It was then filtered through a pad of Celite, washed with EtOH (2 × 20 mL) and the volatiles evaporated. The product 2-aminobenzene-1,4-diol (**II**) was obtained as a brown solid (210 mg, 99% yield). Physical and spectroscopic data were identical to those reported previously [5].

3-Nitrobenzene-1,2-diol (IV)

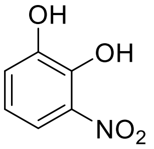

Catechol (**III**, 10.0 g, 90.9 mmol) was dissolved in Et_2O (500 mL) and the solution was cooled to 0 °C in an ice bath. Fuming HNO_3 (4.0 mL) was added dropwise in 2 h, then the reaction mixture was allowed to warm to room temperature where it was stirred for 16 h. The mixture was extracted with H_2O (3 ×

150 mL), followed by evaporation of the volatiles. The residue was triturated with *n*-hexane (300 mL), heated to boiling and then filtered. The mother liquor was concentrated and the trituration process was repeated once more. The product 3-nitrobenzene-1,2-diol (**IV**) was obtained as a yellow solid (1343 mg, 10% yield). Physical and spectroscopic data were identical to those reported previously [1].

3-Aminobenzene-1,2-diol (VII)

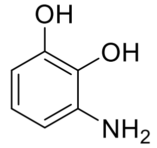

To a solution of 3-nitrobenzene-1,2-diol (**IV**, 151 mg, 0.97 mmol) in MeOH (10 mL), 10% Pd/C (Selcat Q6, 15 mg) was added. The mixture was hydrogenated at atmospheric pressure at room temperature for 4 h. It was then filtered through a pad of Celite, washed with MeOH (2 × 20 mL) and the volatiles evaporated. The product 3-aminobenzene-1,2-diol (**VII**) was obtained as a brown solid (119 mg, 98% yield). Physical and spectroscopic data were identical to those reported previously [6].

5-Methoxy-1,3-benzoxazole-2-thiol (VIII)

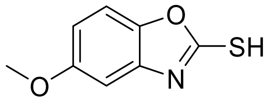

To a solution of 2-amino-4-methoxyphenol (**XIX**, 415 mg, 2.98 mmol, 1.0 equiv.) in EtOH (15 mL) and H_2O (3 mL), CS_2 (180 μL, 2.98 mmol, 1.0 equiv.) and KOH (167 mg, 2.98 mmol, 1.0 equiv.) were added. The reaction mixture was allowed to reflux for 4 h, then it was cooled to room temperature, diluted with H_2O (60 mL), and the pH was adjusted to 1 with 10% HCl. The precipitate formed was filtered off and washed with H_2O (2 × 30 mL). The product 5-methoxy-1,3-benzoxazole-2-thiol (**VIII**) was obtained as a brown solid (449 mg, 83% yield). Mp.: 196–200 °C; ^{1}H NMR (500 MHz, DMSO-d_6) 13.75 (s, 1H), 7.39 (d, *J* = 8.9 Hz, 1H), 6.80 (dd, *J* = 8.9, 2.5 Hz, 1H), 6.75 (d, *J* = 2.3 Hz, 1H), 3.77 (s, 3H); ^{13}C NMR (126 MHz, DMSO-d_6) 180.59, 157.16, 142.39, 131.93, 110.37, 109.98, 95.60, 55.86; HRMS (APCI$^-$) *m/z* [M–H]$^-$, calcd. for $C_8H_6NO_2S$: 180.0124, found: 180.0107; Purity by HPLC: 99%

5-Nitro-1,3-benzoxazole-2-thiol (IX)

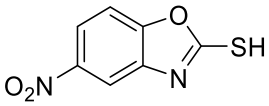

To a solution of 2-amino-4-nitrophenol (**XX**, 1064 mg, 6.90 mmol, 1.0 equiv.) in EtOH (31 mL) and H_2O (7 mL) CS_2 (417 μL, 6.90 mmol, 1.0 equiv.) and KOH (387 mg, 6.90 mmol, 1.0 equiv.) were added. The reaction mixture was allowed to reflux for 7 h, then it was cooled to room temperature, diluted with H_2O (90 mL), and the pH was adjusted to 1 with 10% HCl. The precipitate formed was filtered off and washed with H_2O (2 × 30 mL). The 5-nitro-1,3-benzoxazole-2-thiol (**IX**) was obtained as a yellow solid (891 mg, 66% yield). Mp.: 231–235 °C; ^{1}H NMR (500 MHz, DMSO-d_6) 8.17 (dd, *J* = 8.9, 2.3 Hz, 1H), 7.92 (d, *J* = 2.3 Hz, 1H), 7.71 (d, *J* = 8.9 Hz, 1H); ^{13}C NMR (126 MHz, DMSO-d_6) 181.42, 151.96, 144.66, 132.22,

120.01, 110.29, 105.67; HRMS (APCI$^-$) *m/z* [M–H]$^-$, calcd. for $C_7H_3N_2O_3S$: 194.9869, found: 194.9855; Purity by HPLC: 99%

2.2. Synthesis of benzoxazole-2-carbonitriles

6-Methyl-1,3-benzoxazole-2-carbonitrile (5)

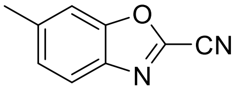

The reaction was carried out according to Method A, starting from 6-amino-*m*-cresol (**X**, 74 mg, 0.60 mmol) and Appel's salt (138 mg, 0.66 mmol). Reagents were stirred in pyridine (3.6 mL) at room temperature for 1 h, then the mixture was heated to 100 °C for 1 h. After workup, compound **5** (20 mg, 21% yield) was isolated as a dark brown solid. Mp.: 54–55 °C; ^{1}H NMR (500 MHz, DMSO-*d*$_6$) 7.85 (d, *J* = 8.3 Hz, 1H), 7.74 (s, 1H), 7.42 (d, *J* = 8.2 Hz, 1H), 2.51 (s, 3H); ^{13}C NMR (126 MHz, DMSO-*d*$_6$) 150.22, 140.12, 136.68, 136.51, 128.08, 120.82, 111.56, 109.78, 21.37; Purity by HPLC: 99%. HRMS measurements (ESI$^+$, ESI$^-$, APCI$^+$ and APCI$^-$) were done, but the compound was not ionizable.

4-Methoxy-1,3-benzoxazole-2-carbonitrile (6)

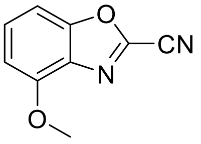

The reaction was carried out according to Method A, starting from 2-amino-3-methoxyphenol hydrochloride (**XI**, 176 mg, 1.0 mmol) and Appel's salt (229 mg, 1.1 mmol). Reagents were stirred in pyridine (6 mL) at room temperature for 1 h, then the mixture was heated to 100 °C for 2 h. After workup, compound **6** (103 mg, 59% yield) was isolated as a brown solid. Mp.: 54–57 °C; ^{1}H NMR (500 MHz, DMSO-*d*$_6$) 7.62 (t, *J* = 8.3 Hz, 1H), 7.44 (d, *J* = 8.3 Hz, 1H), 7.11 (d, *J* = 8.2 Hz, 1H), 4.01 (s, 3H); ^{13}C NMR (126 MHz, DMSO-*d*$_6$) 151.97, 151.25, 135.65, 130.35, 128.78, 109.68, 108.04, 103.74, 56.46; Purity by HPLC: 98%. HRMS measurements (ESI$^+$, ESI$^-$, APCI$^+$ and APCI$^-$) were done, but the compound was not ionizable.

5-Methoxy-1,3-benzoxazole-2-carbonitrile (7)

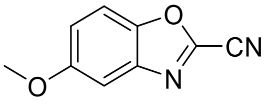

5-Methoxy-1,3-benzoxazole-2-thiol (**VIII**, 181 mg, 1.0 mmol**)** was charged in a flask, followed by a dropwise addition of $SOCl_2$ (6 mL) and DMF (one drop). The reaction mixture was allowed to reflux for 1 h, the volatiles were then removed under reduced pressure and the crude 2-chloro-5-methoxy-1,3-benzoxazole was used in the next step without further purification.

To a solution of the obtained 2-chloro-5-methoxy-1,3-benzoxazole in DMF (8 mL), KCN (91 mg, 1.4 mmol, 1.4 equiv.) was added. The reaction mixture was allowed to stir at 80 °C for 1 h. After the reaction was complete, it was diluted with H_2O (30 mL) and extracted with DCM (3 × 30 mL). The volatiles were removed under reduced pressure and the crude residue was purified by reversed-phase

flash column chromatography using eluents A (0.1% HCOOH in MeCN) and B (0.1% HCOOH in H_2O) (gradient from 1:9 to 10:0). The product 5-methoxy-1,3-benzoxazole-2-carbonitrile (**7**) was obtained as a brown solid (71 mg, 41% yield). Mp.: 66–70 °C; ^1H NMR (500 MHz, DMSO-d_6) 7.81 (d, *J* = 9.1 Hz, 1H), 7.48 (d, *J* = 2.5 Hz, 1H), 7.27 (dd, *J* = 9.1, 2.6 Hz, 1H), 3.84 (s, 3H); ^{13}C NMR (126 MHz, DMSO-d_6) 158.31, 144.58, 139.83, 137.44, 118.77, 112.22, 109.67, 103.17, 56.02; Purity by HPLC: 99%. HRMS measurements (ESI$^+$, ESI$^-$, APCI$^+$ and APCI$^-$) were done, but the compound was not ionizable.

6-Methoxy-1,3-benzoxazole-2-carbonitrile (8)

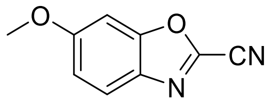

The reaction was carried out according to Method A, starting from 2-amino-5-methoxyphenol (**XII**, 139 mg, 1.0 mmol) and Appel's salt (229 mg, 1.1 mmol). Reagents were stirred in pyridine (6 mL) at room temperature for 1 h, then the mixture was heated to 100 °C for 2 h. After workup, compound **8** (25 mg, 14% yield) was isolated as a brown solid. Mp.: 68—72 °C; ^1H NMR (500 MHz, DMSO-d_6) 7.84 (d, *J* = 8.6 Hz, 1H), 7.53 – 7.50 (m, 1H), 7.20 – 7.15 (m, 1H), 3.87 (s, 3H); ^{13}C NMR (126 MHz, DMSO-d_6) 161.04, 151.47, 135.84, 132.43, 121.73, 116.34, 109.85, 95.63, 56.30; HRMS (APCI$^+$) *m/z* $[M+H]^+$, calcd. for $C_9H_7N_2O_2$: 175.0502, found: 175.0504; Purity by HPLC: 99%

7-Methoxy-1,3-benzoxazole-2-carbonitrile (9)

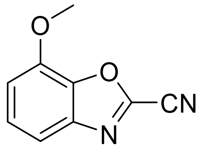

The reaction was carried out according to Method A, starting from 2-amino-6-methoxyphenol (**XIII**, 139 mg, 1.0 mmol) and Appel's salt (229 mg, 1.1 mmol). Reagents were stirred in pyridine (6 mL) at room temperature for 1 h, then the mixture was heated to 100 °C for 2 h. After workup, compound **9** (93 mg, 53% yield) was isolated as a light brown solid. Mp.: 86–90 °C; ^1H NMR (500 MHz, DMSO-d_6) 7.52 – 7.49 (m, 2H), 7.32 – 7.27 (m, 1H), 4.00 (s, 3H); ^{13}C NMR (126 MHz, DMSO-d_6) 144.74, 140.38, 139.33, 136.91, 127.35, 112.98, 111.33, 109.52, 56.50; HRMS (APCI$^+$) *m/z* $[M+H]^+$, calcd. for $C_9H_7N_2O_2$: 175.0502, found: 175.0501; Purity by HPLC: 100%

4-Hydroxy-1,3-benzoxazole-2-carbonitrile (10)

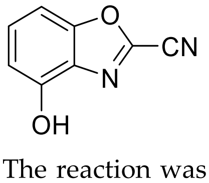

The reaction was carried out according to Method B, starting from 2-aminobenzene-1,3-diol (**XIV**, 75 mg, 0.6 mmol) and Appel's salt (125 mg, 0.6 mmol). After workup, compound **10** (15 mg, 16% yield) was isolated as a brown solid. Mp.: 107–111 °C; ^1H NMR (500 MHz, DMSO-d_6) 10.99 (s, 1H), 7.47 (t, *J* = 8.2 Hz, 1H), 7.27 (d, *J* = 8.4 Hz, 1H), 6.92 (d, *J* = 7.9 Hz, 1H); ^{13}C NMR (126 MHz, DMSO-d_6) 151.55, 150.77, 135.17, 130.17, 128.39, 111.46, 109.83, 101.79; HRMS (APCI$^-$) *m/z* $[M–H]^-$, calcd. for $C_8H_3N_2O_2$: 159.0189, found: 159.0194; Purity by HPLC: 97%

5-Hydroxy-1,3-benzoxazole-2-carbonitrile (11)

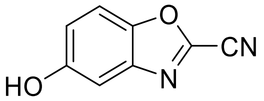

The reaction was carried out according to Method B, starting from 2-aminobenzene-1,4-diol (**II**, 63 mg, 0.5 mmol) and Appel's salt (104 mg, 0.5 mmol). After workup, compound **11** (4 mg, 5% yield) was isolated as a brown solid. Mp.: 140–145 °C; ^{1}H NMR (300 MHz, DMSO-d_6) 10.01 (s, 1H), 7.73 (d, J = 9.1 Hz, 1H), 7.20 (s, 1H), 7.14 (d, J = 8.9 Hz, 1H). ^{13}C NMR (75 MHz, DMSO-d_6) 156.42, 143.82, 139.88, 137.32, 118.57, 112.09, 109.83, 105.12; HRMS (APCI$^-$) m/z [M–H]$^-$, calcd. for $C_8H_3N_2O_2$: 159.0189, found: 159.0198; Purity by HPLC: 100%

6-Hydroxy-1,3-benzoxazole-2-carbonitrile (12)

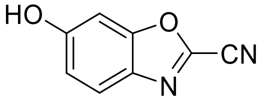

The reaction was carried out according to Method C, starting from 4-aminobenzene-1,3-diol (**XV**, 125 mg, 1.0 mmol) and Appel's salt (209 mg, 1.0 mmol). After workup, compound **12** (8 mg, 5% yield) was isolated as a brown solid. Mp.: 135–140 °C; ^{1}H NMR (300 MHz, DMSO-d_6) 10.52 (s, 1H), 7.77 (d, J = 8.8 Hz, 1H), 7.17 (s, 1H), 7.04 (d, J = 7.2 Hz, 1H); ^{13}C NMR (75 MHz, DMSO-d_6) 159.52, 151.45, 135.21, 131.43, 121.85, 116.39, 109.98, 97.23; HRMS (APCI$^-$) m/z [M–H]$^-$, calcd. for $C_8H_3N_2O_2$: 159.0189, found: 159.0196; Purity by HPLC: 100%

7-Hydroxy-1,3-benzoxazole-2-carbonitrile (13)

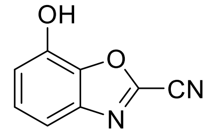

The reaction was carried out according to Method B, starting from 3-aminobenzene-1,2-diol (**VII**, 63 mg, 0.5 mmol) and Appel's salt (104 mg, 0.5 mmol). After workup, compound **13** (15 mg, 19% yield) was isolated as a brown solid. Mp.: 115–116 °C; ^{1}H NMR (300 MHz, DMSO-d_6) 10.51 (s, 1H), 7.37 (d, J = 4.6 Hz, 4H), 7.13 – 7.06 (m, 2H); ^{13}C NMR (75 MHz, DMSO-d_6) 142.93, 140.72, 139.22, 136.77, 127.29, 114.85, 111.35, 109.78; HRMS (APCI$^-$) m/z [M–H]$^-$, calcd. for $C_8H_3N_2O_2$: 159.0189, found: 159.0196; Purity by HPLC: 96%

2-Cyano-1,3-benzoxazol-4-yl acetate (14)

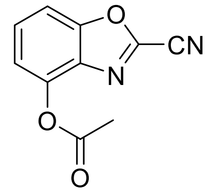

The reaction was carried out according to general *O*-acylation procedure, starting from 4-hydroxy-1,3-benzoxazole-2-carbonitrile (**6**, 17 mg, 0.10 mmol) and acetic anhydride (30 μL, 0.31 mmol). After

workup, compound **14** (5 mg, 24% yield) was isolated as a brown solid. Mp.: 65 °C; ^{1}H NMR (500 MHz, DMSO-d_6) 7.85 (dd, *J* = 7.5, 6.9 Hz, 1H), 7.73 (t, *J* = 8.2 Hz, 1H), 7.44 – 7.40 (m, 1H), 2.41 (s, 3H); ^{13}C NMR (75 MHz, DMSO-d_6) 168.36, 150.99, 142.17, 132.13, 129.73, 129.63, 119.54, 109.81, 109.46, 20.46; Purity by HPLC: 98%. HRMS measurements (ESI$^+$, ESI$^-$, APCI$^+$ and APCI$^-$) were done, but the compound was not ionizable.

2-Cyano-1,3-benzoxazol-5-yl acetate (15)

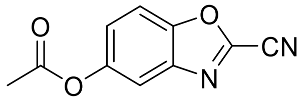

The reaction was carried out according to general *O*-acylation procedure, starting from 5-hydroxy-1,3-benzoxazole-2-carbonitrile (**11**, 13 mg, 0.08 mmol) and acetic anhydride (23 µL, 0.24 mmol). After workup, compound **15** (4 mg, 25% yield) was isolated as a brown solid. Mp.: 58 °C; ^{1}H NMR (300 MHz, DMSO-d_6) 7.99 (d, *J* = 9.0 Hz, 1H), 7.83 (d, *J* = 2.0 Hz, 1H), 7.49 (dd, *J* = 9.0, 2.1 Hz, 1H), 2.31 (s, 3H); ^{13}C NMR (75 MHz, DMSO-d_6) 169.31, 148.69, 147.58, 143.17, 139.34, 138.39, 123.84, 114.61, 112.39, 20.81; Purity by HPLC: 100%. HRMS measurements (ESI$^+$, ESI$^-$, APCI$^+$ and APCI$^-$) were done, but the compound was not ionizable.

4-Nitro-1,3-benzoxazole-2-carbonitrile (16)

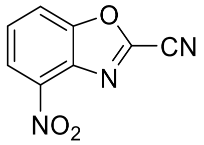

The reaction was carried out according to Method A, starting from 2-amino-3-nitrophenol (**XVI**, 339 mg, 2.2 mmol) and Appel's salt (505 mg, 2.4 mmol). Reagents were stirred in pyridine (13.2 mL) at room temperature for 1 h, then the mixture was heated to 100 °C for 3 h. After workup, compound **16** (140 mg, 34% yield) was isolated as a brown solid. Mp.: 92–95 °C; ^{1}H NMR (500 MHz, DMSO-d_6) 8.47 – 8.40 (m, 2H), 7.94 (t, *J* = 8.3 Hz, 1H); ^{13}C NMR (126 MHz, DMSO-d_6) 151.20, 140.00, 132.81, 129.22, 122.84, 118.93, 109.21; HRMS (APCI$^-$) *m*/*z* [M–H]$^-$, calcd. for $C_8H_3N_3O_3$: 189.0179, found: 189.0151; Purity by HPLC: 99%

5-Nitro-1,3-benzoxazole-2-carbonitrile (17)

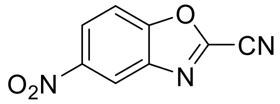

5-Nitro-1,3-benzoxazole-2-thiol (**IX**, 196 mg, 1.0 mmol, 1.0 equiv.) was charged in a flask, followed by a dropwise addition of $SOCl_2$ (6 mL) and DMF (one drop). The reaction mixture was allowed to reflux for 1 h, the volatiles were then removed under reduced pressure and the crude 2-chloro-5-nitro-1,3-benzoxazole was used in the next step without further purification.

To a solution of the obtained 2-chloro-5-nitro-1,3-benzoxazole in DMF (8 mL), KCN (91 mg, 1.4 mmol, 1.4 equiv.) was added. The reaction mixture was allowed to stir at room temperature for 3 h. After the reaction was complete, it was diluted with H_2O (30 mL) and extracted with DCM (3 × 30 mL). The

volatiles were removed under reduced pressure and the crude residue was purified by reversed-phase flash column chromatography using eluents A (0.1% HCOOH in MeCN) and B (0.1% HCOOH in H_2O) (gradient from 1:9 to 10:0). The product 5-nitro-1,3-benzoxazole-2-carbonitrile (**17**) was obtained as a brown solid (118 mg, 63% yield). Mp.: 110–112 °C; ^{1}H NMR (300 MHz, DMSO-d_6) 8.93 (s, 1H), 8.56 (d, *J* = 8.2 Hz, 1H), 8.22 (d, *J* = 9.1 Hz, 1H); ^{13}C NMR (75 MHz, DMSO-d_6) 153.14, 146.16, 140.14, 139.13, 124.59, 117.89, 113.05, 109.24; HRMS (APCI$^-$) *m/z* [M–H]$^-$, calcd. for $C_8H_3N_3O_3$: 189.0179, found: 189.0175; Purity by HPLC: 100%

6-Nitro-1,3-benzoxazole-2-carbonitrile (18)

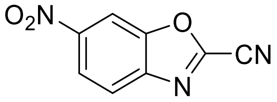

The reaction was carried out according to Method A, starting from 5-nitro-2-amino-fenol (**XVII**, 170 mg, 1.1 mmol) and Appel's salt (252 mg, 1.2 mmol). Reagents were stirred in pyridine (6.6 mL) at room temperature for 1 h, then the mixture was heated to 100 °C for 1 h. After workup, compound **18** (117 mg, 56% yield) was isolated as a brown solid. Mp.: 56 °C; ^{1}H NMR (500 MHz, DMSO-d_6) 8.95 (d, *J* = 2.1 Hz, 1H), 8.43 (dd, *J* = 8.9, 2.1 Hz, 1H), 8.24 (d, *J* = 8.9 Hz, 1H); ^{13}C NMR (126 MHz, DMSO-d_6) 149.65, 147.85, 143.95, 141.66, 122.69, 122.40, 109.66, 109.35; HRMS (APCI$^-$) *m/z* [M–H]$^-$, calcd. for $C_8H_3N_3O_3$: 189.0179, found: 189.0154; Purity by HPLC: 99%

7-Nitro-1,3-benzoxazole-2-carbonitrile (19)

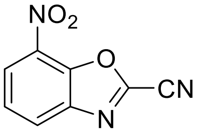

The reaction was carried out according to Method A, starting from 2-amino-6-nitrophenol (**XVIII**, 123 mg, 0.8 mmol) and Appel's salt (184 mg, 0.88 mmol). Reagents were stirred in pyridine (4.8 mL) at room temperature for 1 h, then the mixture was heated to 100 °C for 3 h. After workup, compound **19** (46 mg, 30% yield) was isolated as a brown solid. Mp.: 111–114 °C; ^{1}H NMR (500 MHz, DMSO-d_6) 8.53 (d, *J* = 8.2 Hz, 1H), 8.49 (d, *J* = 8.1 Hz, 1H), 7.84 (t, *J* = 8.2 Hz, 1H); ^{13}C NMR (75 MHz, DMSO-d_6) 142.73, 141.82, 139.04, 133.32, 129.08, 126.99, 124.98, 109.16; HRMS (APCI$^-$) *m/z* [M–H]$^-$, calcd. for $C_8H_3N_3O_3$: 189.0179, found: 189.0155; Purity by HPLC: 100%

4-Amino-1,3-benzoxazole-2-carbonitrile (20)

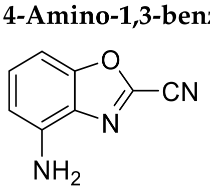

The reaction was carried out according to general nitro reduction procedure, starting from 4-nitro-1,3-benzoxazole-2-carbonitrile (**16**, 227 mg, 1.20 mmol) and $SnCl_2 \times 2H_2O$ (1.62 g, 7.2 mmol). After workup, compound **20** (99 mg, 52% yield) was isolated as a yellow solid. Mp.: 80–84 °C; ^{1}H NMR (500 MHz, DMSO-d_6) 7.31 (t, *J* = 8.1 Hz, 1H), 6.87 (d, *J* = 8.1 Hz, 1H), 6.63 (d, *J* = 8.1 Hz, 1H), 6.19 (s, 2H); ^{13}C

NMR (126 MHz, DMSO-d_6) 151.84, 143.01, 134.25, 130.82, 126.92, 110.53, 109.01, 97.54; HRMS (APCI$^-$) *m/z* [M–H]$^-$, calcd. for $C_8H_4N_3O$: 158.0359, found: 158.0336; Purity by HPLC: 98%

5-Amino-1,3-benzoxazole-2-carbonitrile (21)

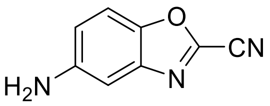

The reaction was carried out according to general nitro reduction procedure, starting from 5-nitro-1,3-benzoxazole-2-carbonitrile (**17**, 102 mg, 0.54 mmol) and $SnCl_2$ × $2H_2O$ (731 mg, 3.24 mmol). After workup, compound **21** (54 mg, 63% yield) was isolated as a yellow solid. Mp.: 169–173 °C; ^{1}H NMR (500 MHz, DMSO-d_6) 7.54 (d, *J* = 8.9 Hz, 1H), 6.94 (dd, *J* = 6.4, 2.5 Hz, 1H), 6.92 (d, *J* = 2.0 Hz, 1H), 5.43 (s, 2H); ^{13}C NMR (126 MHz, DMSO-d_6) 148.31, 142.42, 140.06, 136.39, 117.73, 111.51, 109.95, 101.90. HRMS (APCI$^-$) *m/z* [M–H]$^-$, calcd. for $C_8H_4N_3O$: 158.0359, found: 158.0340; Purity by HPLC: 100%

6-Amino-1,3-benzoxazole-2-carbonitrile (22)

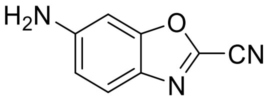

The reaction was carried out according to general nitro reduction procedure, starting from 6-nitro-1,3-benzoxazole-2-carbonitrile (**18**, 76 mg, 0.40 mmol) and $SnCl_2$ × $2H_2O$ (542 mg, 2.4 mmol). After workup, compound **22** (42 mg, 66% yield) was isolated as a yellow solid. Mp.:157 °C; ^{1}H NMR (500 MHz, DMSO-d_6) 7.55 (d, *J* = 8.7 Hz, 1H), 6.83 (dd, *J* = 8.8, 1.9 Hz, 1H), 6.79 (d, *J* = 1.8 Hz, 1H), 6.02 (s, 2H); ^{13}C NMR (126 MHz, DMSO-d_6) 152.37, 151.58, 132.96, 128.77, 121.48, 115.27, 110.38, 92.88; HRMS (APCI$^-$) *m/z* [M–H]$^-$, calcd. for $C_8H_4N_3O$: 158.0359, found: 158.0339; Purity by HPLC: 95%

7-Amino-1,3-benzoxazole-2-carbonitrile (23)

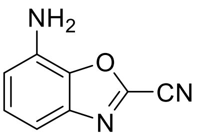

The reaction was carried out according to general nitro reduction procedure, starting from 7-nitro-1,3-benzoxazole-2-carbonitrile (**19**, 31 mg, 0.18 mmol) and $SnCl_2$ × $2H_2O$ (237 mg, 1.05 mmol). After workup, compound **23** (19 mg, 68% yield) was isolated as a yellow solid. Mp.: 96–99 °C; ^{1}H NMR (300 MHz, DMSO-d_6) 7.25 (t, *J* = 6.8 Hz, 1H), 7.05 (d, *J* = 8.0 Hz, 1H), 6.83 (d, *J* = 7.0 Hz, 1H), 5.96 (s, 2H); ^{13}C NMR (75 MHz, DMSO-d_6) 139.72, 138.72, 136.17, 134.25, 127.32, 112.46, 109.96, 107.26; HRMS (APCI$^-$) *m/z* [M–H]$^-$, calcd. for $C_8H_4N_3O$: 158.0359, found: 158.0342; Purity by HPLC: 98%

***N*-(2-cyano-1,3-benzoxazol-4-yl)benzamide (24)**

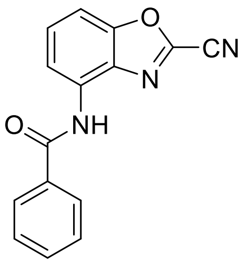

The reaction was carried out according to general *N*-acylation procedure, starting from 4-amino-1,3-benzoxazole-2-carbonitrile (**20**, 18 mg, 0.11 mmol), benzoic anhydride (75 mg, 0.33 mmol), DMAP (1.3 mg, 0.011 mmol) and Et_3N (46 µL, 0.33 mmol). Reagents were stirred in DMF (2 mL) at 120 °C for 48 h and after workup, compound **24** (12 mg, 41% yield) was isolated as a white solid. Mp.: 148–151 °C; ^{1}H NMR (500 MHz, DMSO-d_6) 10.63 (s, 1H), 8.05 – 8.01 (m, 2H), 7.97 (dd, *J* = 6.7, 2.1 Hz, 1H), 7.74 – 7.69 (m, 2H), 7.64 (t, *J* = 7.3 Hz, 1H), 7.57 (t, *J* = 7.5 Hz, 2H); ^{13}C NMR (126 MHz, DMSO-d_6) 166.25, 150.95, 136.64, 134.33, 132.90, 132.52, 132.06, 130.03, 128.92, 128.42, 120.27, 110.13, 108.29; HRMS (APCI$^-$) *m/z* [M–H]$^-$, calcd. for $C_{15}H_8N_3O_2$: 262.0622, found: 262.0588; Purity by HPLC: 100%

N-(2-cyano-1,3-benzoxazol-5-yl)benzamide (25)

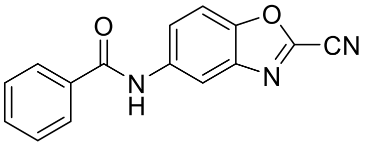

The reaction was carried out according to general *N*-acylation procedure, starting from 5-amino-1,3-benzoxazole-2-carbonitrile (**21**, 40 mg, 0.25 mmol), benzoic anhydride (170 mg, 0.75 mmol), DMAP (3.1 mg, 0.025 mmol) and Et_3N (105 µL, 0.75 mmol). Reagents were stirred in DMF (2 mL) at room temperature for 5 h and after workup, compound **25** (45 mg, 68% yield) was isolated as a white solid. Mp.: 187–188 °C; ^{1}H NMR (500 MHz, DMSO-d_6) 10.55 (s, 1H), 8.46 (d, *J* = 1.8 Hz, 1H), 8.02 – 7.96 (m, 3H), 7.90 (d, *J* = 9.0 Hz, 1H), 7.62 (t, *J* = 7.3 Hz, 1H), 7.55 (t, *J* = 7.4 Hz, 2H); ^{13}C NMR (126 MHz, DMSO-d_6) 165.80, 146.21, 138.99, 137.90, 137.61, 134.52, 131.76, 128.41, 127.67, 122.64, 111.73, 111.68, 109.66; HRMS (APCI$^-$) *m/z* [M–H]$^-$, calcd. for $C_{15}H_8N_3O_2$: 262.0622, found: 262.0588; Purity by HPLC: 100%

N-(2-cyano-1,3-benzoxazol-6-yl)benzamide (26)

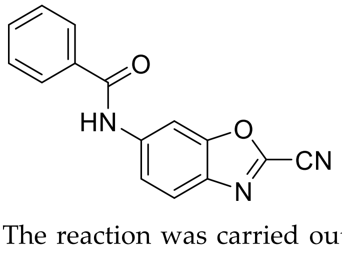

The reaction was carried out according to general *N*-acylation procedure, starting from 6-amino-1,3-benzoxazole-2-carbonitrile (**22**, 20 mg, 0.13 mmol), benzoic anhydride (88 mg, 0.39 mmol), DMAP (1.5 mg, 0.013 mmol) and Et_3N (52 µL, 0.39 mmol). Reagents were stirred in DMF (2 mL) at room temperature for 16 h and after workup, compound **26** (18 mg, 55% yield) was isolated as a light-yellow solid. Mp.: 201–202 °C; ^{1}H NMR (500 MHz, DMSO-d_6) 10.71 (s, 1H), 8.51 (d, *J* = 1.6 Hz, 1H), 8.03 – 7.94 (m, 3H), 7.88 (dd, *J* = 8.9, 1.8 Hz, 1H), 7.63 (t, *J* = 7.3 Hz, 1H), 7.56 (t, *J* = 7.5 Hz, 2H); ^{13}C NMR (126 MHz,

DMSO-d_6) 166.04, 150.31, 140.52, 136.60, 134.48, 134.36, 131.93, 128.44, 127.75, 121.29, 119.74, 109.79, 102.21; HRMS (APCI$^-$) *m/z* [M–H]$^-$, calcd. for $C_{15}H_8N_3O_2$: 262.0622, found: 262.0589; Purity by HPLC: 100%

N-(2-cyano-1,3-benzoxazol-7-yl)benzamide (27)

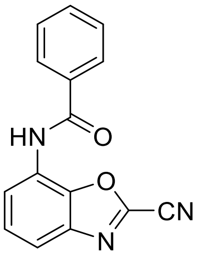

The reaction was carried out according to general *N*-acylation procedure, starting from 7-amino-1,3-benzoxazole-2-carbonitrile (**23**, 15 mg, 0.095 mmol), benzoic anhydride (64 mg, 0.29 mmol), DMAP (1.2 mg, 0.0095 mmol) and Et_3N (40 µL, 0.29 mmol). Reagents were stirred in DMF (2 mL) at 100 °C for 5 h and after workup, compound **27** (15 mg, 60% yield) was isolated as a white solid. Mp.: 157–160 °C; ^{1}H NMR (500 MHz, DMSO-d_6) 10.82 (s, 1H), 8.06 – 8.01 (m, 2H), 7.86 – 7.79 (m, 2H), 7.67 – 7.55 (m, 4H); ^{13}C NMR (126 MHz, DMSO-d_6) 165.39, 143.41, 139.92, 136.87, 133.43, 132.15, 128.50, 127.96, 126.79, 123.70, 123.60, 117.96, 109.57; HRMS (APCI$^-$) *m/z* [M–H]$^-$, calcd. for $C_{15}H_8N_3O_2$: 262.0622, found: 262.0590; Purity by HPLC: 98%

2.3. Synthesis of boronic acid derivatives

Methyl 2-(2-bromo-1,3-thiazol-4-yl)acetate (XXIV)

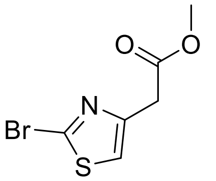

In a round bottom flask, the commercially available 2-(2-bromothiazol-4-yl)acetic acid (**XXIII**, 244 mg, 1.1 mmol) was dissolved in MeOH (20 mL) at 0 °C. Thionyl chloride (235 µL, 3.3 mmol, 3.0 equiv) was added dropwise and the reaction mixture was stirred at room temperature overnight. The product was isolated as an orange solid after evaporation of the solvent (250 mg, 95% yield). Mp.: 91–93 °C; ^{1}H NMR (500 MHz, DMSO-d_6) δ 7.52 (s, 1H), 7.48 (s, 1H) [duplication of signals], 3.72 (s, 3H), 3.69 (s, 2H); ^{13}C NMR (126 MHz, DMSO-d_6) δ 171.52, 150.52, 122.12, 122.60, 36.96, 36.81; HRMS (ESI$^+$) *m/z* [M+H]$^+$, calcd. for $C_6H_7NO_2SBr$: 235.9380, found: 235.9379; Purity by HPLC: >95%

Methyl 2-(2-ethenyl-1,3-thiazol-4-yl)acetate (XXV)

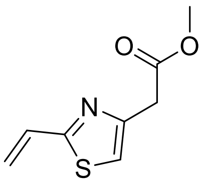

In a sealed tube, **XXIV** (222 mg, 0.94 mmol) was dissolved in dry THF (10 mL), bis(triphenylphosphine)palladium(II) dichloride (66 mg, 0.094 mmol, 10%) was added, followed by tributyl(vinyl)tin (440 µL, 1.5 mmol, 1.6 equiv.). The reaction mixture was heated to 100 °C and stirred for 2 h. The mixture was cooled and purified directly by flash column chromatography (*n*-hexane-EtOAc) to afford the product as a colourless oil (70 mg, 40% yield). ^{1}H NMR (500 MHz, $CDCl_3$) δ 7.11 (s, 1H), 7.10 (s, 1H) [duplication of signals], 6.89 (dd, *J* = 17.5, 10.9 Hz, 1H), 6.01 (d, *J* = 17.5 Hz, 1H), 5.53 (d, *J* = 10.9 Hz, 1H), 3.83 (s, 2H), 3.77 (s, 2H) [duplication of signals], 3.74 (s, 3H), 3.73 (s, 3H) [duplication of signals]; ^{13}C NMR (126 MHz, $CDCl_3$) δ 170.63, 170.10 [duplication of signals], 149.14, 147.66 [duplication of signals], 151.48, 130.37, 119.92, 118.29, 115.70, 52.23, 52.13 [duplication of signals], 36.73, 36.71 [duplication of signals]; Purity by HPLC: >95%

2-(2-Ethenyl-1,3-thiazol-4-yl)acetic acid (XXVI)

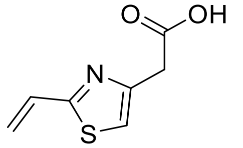

In a round bottom flask, **XXV** (44 mg, 0.24 mmol) was dissolved in MeOH (10 mL) and 1 M NaOH (1.0 mL) solution was added. The mixture was stirred at room temperature for 5 h until full conversion. After evaporation of the solvent, the crude was suspended in H_2O (15 mL) and the pH was set to 1 by 10% HCl. EtOAc (3 × 10 mL) was used to extract the product that was isolated after drying ($MgSO_4$) and evaporating the organic solvent to obtain a yellowish oil (40 mg, 99% yield). ^{1}H NMR (500 MHz, DMSO-d_6) δ 7.44 (s, 1H), 6.91 (dd, *J* = 17.4, 10.9 Hz, 1H), 6.01 (d, *J* = 17.5 Hz, 1H), 5.56 (d, *J* = 10.9 Hz, 1H), 3.82 (s, 2H); ^{13}C NMR (126 MHz, DMSO-d_6) δ 187.4, 144.5, 133.4, 122.6, 116.1, 108.2, 31.8; HRMS (ESI$^-$) *m/z* $[M–H]^-$, calcd. for $C_7H_6NO_2S$: 168.0119, found: 168.0125; Purity by HPLC: >95%

2-(2-Ethenyl-1,3-thiazol-4-yl)-*N*-[(1*R*)-3-methyl-1-[(1*S*,2*S*,6*R*,8*S*)-2,9,9-trimethyl-3,5-dioxa-4-boratricyclo[6.1.1.0^{2,6}]decan-4-yl]butyl]acetamide (28)

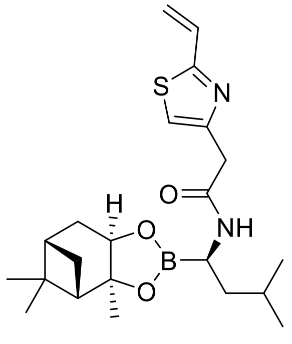

In a round bottom flask, **XXVI** (34 mg, 0.2 mmol) was dissolved in DMF (3.0 mL) under Ar atmosphere at room temperature. (*R*)-BoroLeu-(+)-pinanediol trifluoroacetate (76 mg, 0.2 mmol, 1.0 equiv), HATU (84 mg, 0.22 mmol, 1.1 equiv), and DIPEA (77 µL, 0.44 mmol, 2.2 equiv.) were added subsequently, followed by stirring the reaction mixture overnight at room temperature. The mixture was then purified directly by reversed-phase flash column chromatography using eluents A (0.1% HCOOH in MeCN) and B (0.1% HCOOH in H_2O) (gradient from 1:9 to 10:0) to give the product as a brownish oil (8 mg, 9% yield). ^{1}H NMR (500 MHz, DMSO-d_6) δ 7.07 (d, *J* = 9.5 Hz, 1H), 6.87 (dd, *J* =

17.4, 10.9 Hz, 1H), 6.10 (d, *J* = 17.5 Hz, 1H), 5.59 (d, *J* = 10.9 Hz, 1H), 4.31 – 4.23 (m, 1H), 3.77 (s, 2H), 3.10 – 2.91 (m, 2H), 2.37 – 2.29 (m, 1H), 2.19 – 2.12 (m, 1H), 2.06 (d, *J* = 11.1 Hz, 1H), 2.02 (dd, *J* = 10.8, 5.4 Hz, 1H), 1.87 – 1.81 (m, 1H), 1.67 – 1.59 (m, 1H), 1.45 (dd, *J* = 14.1, 7.7 Hz, 2H), 1.41 (s, 6H), 0.91 (dd, *J* = 10.6, 6.2 Hz, 6H), 0.86 (s, 3H) ppm; ^{13}C NMR (126 MHz, DMSO-d_6) δ 176.22, 129.63, 120.48, 118.19, 115.96, 84.57, 51.85, 39.84, 38.17, 37.30, 36.17, 36.02, 35.81, 28.78, 27.24, 26.39, 25.98, 24.12, 23.12, 21.97 ppm; HRMS (ESI$^+$) *m/z* $[M+H]^+$, calcd. for $C_{22}H_{34}N_2O_3SBr$: 417.2383, found: 417.2393; Purity by HPLC: >95%

2-(Trichloromethyl)-1*H*-1,3-benzodiazole (XXVIII)

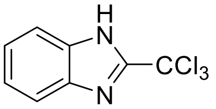

To a stirred solution of benzene-1,2-diamine (**XXVII**, 3785 mg, 35.00 mmol) in acetic acid (100 mL), methyl 2,2,2-trichloroacetimidate (4.33 mL, 35.00 mmol, 1.0 equiv.) was added dropwise. The mixture was allowed to stir at room temperature for 1 h. The white precipitate was filtered and washed with H_2O (3 × 40 mL). The product 2-(trichloromethyl)-1*H*-1,3-benzodiazole (**XXVIII)** was obtained as a white powder (7254 mg, 88% yield). Physical and spectroscopic data were identical to those reported previously [7].

1*H*-1,3-Benzodiazole-2-carbonitrile (XXIX)

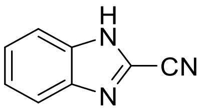

2-(Trichloromethyl)-1*H*-1,3-benzodiazole (**XXVIII**, 7154 mg, 30.38 mmol) was dissolved in ammonia solution (7 M in MeOH, 350 mL) and the reaction mixture was allowed to stir at room temperature for 4 h. White precipitation was then observed and the resulting mixture was concentrated, re-dissolved in EtOAc (500 mL), and washed with H_2O (300 mL). The aqueous layer was washed with EtOAc (2 × 100 mL) and the combined organic layers were concentrated. The concentrate was suspended in Et_2O (300 mL), sonicated for 15 min, and the mixture filtered. The product, 1*H*-1,3-benzodiazole-2-carbonitrile (**XXIX**), was obtained as a white powder (2880 mg, 60% yield). Physical and spectroscopic data were identical to those reported previously [7].

2-(2-Cyano-1*H*-1,3-benzodiazol-1-yl)acetic acid (XXX)

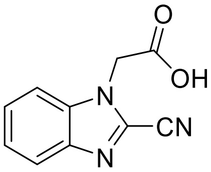

1*H*-1,3-Benzodiazole-2-carbonitrile (**XXIX**, 501 mg, 3.50 mmol, 1.0 equiv.) was dissolved in DMF (20 mL) and DIPEA (1219 µL, 7.00 mmol, 2.0 equiv.). Ethyl bromoacetate (426 µL, 4.20 mmol, 1.2 equiv.) was then added dropwise and the reaction mixture was stirred at room temperature for 1 h. After the reaction was complete, it was diluted with THF (30 mL) and H_2O (60 mL). LiOH × H_2O (2.0 equiv.) was then added and the reaction mixture was allowed to stir at room temperature for 4 h. The mixture

was concentrated, the residue re-dissolved in H_2O (80 mL,) and washed with EtOAc (100 mL). The aqueous layer was acidified to pH 1 with 1 M HCl and extracted with EtOAc (3 × 80 mL). The combined organic layers were dried ($MgSO_4$), filtered, and the volatiles were removed under reduced pressure. The crude residue was purified by reversed-phase flash column chromatography using eluents A (0.1% HCOOH in MeCN) and B (0.1% HCOOH in H_2O) (gradient from 1:9 to 10:0). The product 2-(2-cyano-1*H*-1,3-benzodiazol-1-yl)acetic acid (**XXX**) was obtained as a light yellow powder (344 mg, 49% yield). Mp.: 140–143 °C; ^{1}H NMR (500 MHz, DMSO-*d*$_6$) 7.83 (t, *J* = 7.5 Hz, 2H), 7.52 (t, *J* = 7.8 Hz, 1H), 7.43 (t, *J* = 8.0 Hz, 1H), 5.39 (s, 2H), resonance for COOH missing; ^{13}C NMR (126 MHz, DMSO-*d*$_6$) 168.55, 141.53, 134.55, 127.03, 126.39, 124.32, 120.74, 111.67, 111.28, 45.83; HRMS (ESI$^+$) *m*/*z* [M+H]$^+$, calcd. for $C_{10}H_8N_3O_2$: 202.0617, found: 202,0617; Purity by HPLC: 99%.

2-(2-Cyano-1*H*-1,3-benzodiazol-1-yl)-*N*-[(1*R*)-3-methyl-1-[(1*S*,2*S*,6*R*,8*S*)-2,9,9-trimethyl-3,5-dioxa-4-boratricyclo[6.1.1.0^{2,6}]decan-4-yl]butyl]acetamide (29)

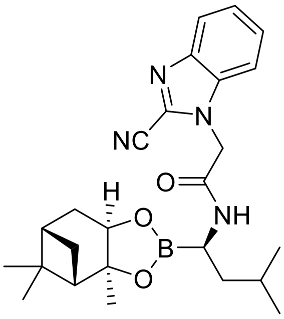

To a stirred solution of 2-(2-cyano-1*H*-1,3-benzodiazol-1-yl)acetic acid (**XXX**, 81 mg, 0.40 mmol, 1.0 equiv.), HATU (167 mg, 0.44 mmol, 1.1 equiv.) and DIPEA (153 µL, 0.88 mmol, 2.2 equiv.) in DMF (2.5 mL), (*R*)-BoroLeu-(+)-pinanediol trifluoroacetate (152 mg, 0.40 mmol, 1.0 equiv.) was added and the mixture was allowed to stir at room temperature for 2 h. The mixture was purified by reversed-phase flash column chromatography using eluents A (0.1% HCOOH in MeCN) and B (0.1% HCOOH in H_2O) (gradient from 1:9 to 10:0). The obtained viscous oil was triturated with Et_2O (20 mL) and concentrated. The product 2-(2-cyano-1*H*-1,3-benzodiazol-1-yl)-*N*-[(1*R*)-3-methyl-1-[(1*S*,2*S*,6*R*,8*S*)-2,9,9-trimethyl-3,5-dioxa-4-boratricyclo[6.1.1.0^{2,6}]decan-4-yl]butyl]acetamide (**29**) was obtained as a white powder (144 mg, 80% yield). Mp.: 71–72 °C; ^{1}H NMR (500 MHz, DMSO-*d*$_6$) 8.64 (d, *J* = 5.7 Hz, 1H), 7.82 (d, *J* = 8.2 Hz, 1H), 7.66 (d, *J* = 8.3 Hz, 1H), 7.50 (t, *J* = 7.7 Hz, 1H), 7.41 (t, *J* = 7.7 Hz, 1H), 5.14 (s, *J* = 6.2 Hz, 2H), 4.22 (dd, *J* = 8.7, 1.8 Hz, 1H), 3.00 (dt, *J* = 9.9, 5.9 Hz, 1H), 2.21 (dt, *J* = 21.8, 9.6 Hz, 1H), 1.98 (ddd, *J* = 10.6, 6.1, 4.5 Hz, 1H), 1.86 (t, *J* = 5.5 Hz, 1H), 1.77 (dd, *J* = 6.6, 4.2 Hz, 1H), 1.69 – 1.59 (m, 2H), 1.45 (ddd, *J* = 15.3, 9.6, 5.8 Hz, 1H), 1.31 (ddd, *J* = 7.8, 5.1, 1.8 Hz, 1H), 1.21 (s, *J* = 5.9 Hz, 6H), 1.09 (t, *J* = 8.1 Hz, 1H), 0.85 (dt, *J* = 10.7, 5.4 Hz, 6H), 0.77 (s, 3H); ^{13}C NMR (126 MHz, DMSO-*d*$_6$) 165.30, 141.62, 134.50, 127.26, 126.04, 124.11, 120.64, 111.52, 111.28, 84.93, 76.73, 50.88, 46.52, 38.88, 37.67, 35.01, 28.23, 26.83, 25.66, 24.70, 23.60, 22.91, 21.79; HRMS (ESI$^+$) *m*/*z* [M+H]$^+$, calcd. for $C_{25}H_{34}N_4O_3B$: 449.2724, found: 449.2725; Purity by HPLC: 98%.

[(1*R*)-1-[2-(2-Cyano-1*H*-1,3-benzodiazol-1-yl)acetamido]-3-methylbutyl]boronic acid (33)

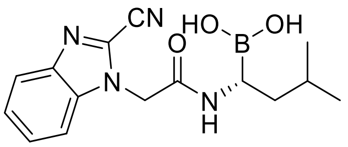

To a stirred solution of 2-(2-cyano-1*H*-1,3-benzodiazol-1-yl)-*N*-[(1*R*)-3-methyl-1-[(1*S*,2*S*,6*R*,8*S*)-2,9,9-trimethyl-3,5-dioxa-4-boratricyclo[6.1.1.0^{2,6}]decan-4-yl]butyl]acetamide (**29**, 58 mg, 0.13 mmol, 1.0 equiv.) in acetone (6 mL) and H_2O (6 mL), $NaIO_4$ (83 mg, 0.39 mmol, 3.0 equiv.) and NH_4OAc (24 mg, 0.31 mmol, 2.4 equiv.) were added and the mixture was allowed to stir at room temperature for 16 h. The mixture was then diluted with H_2O (10 mL), pH was adjusted to 3 with 1 M HCl, and it was extracted with EtOAc (3 × 20 mL). The combined organic layers were removed under reduced pressure and the crude residue was purified by reversed-phase flash column chromatography using eluents A (0.1% HCOOH in MeCN) and B (0.1% HCOOH in H_2O) (gradient from 1:9 to 10:0 The obtained viscous oil was triturated with Et_2O (10 mL) and concentrated. The product [(1*R*)-1-[2-(2-cyano-1*H*-1,3-benzodiazol-1-yl)acetamido]-3-methylbutyl]boronic acid (**33**) was obtained as a white powder (20 mg, 49% yield). Mp.: 120–121 °C; ^{1}H NMR (500 MHz, DMSO-d_6) 8.82 (d, *J* = 8.5 Hz, 1H), 7.83 (d, *J* = 8.2 Hz, 1H), 7.66 (d, *J* = 8.3 Hz, 1H), 7.51 (t, *J* = 7.7 Hz, 1H), 7.42 (t, *J* = 7.7 Hz, 1H), 5.14 – 5.11 (m, 2H), 1.67 (dp, *J* = 13.5, 6.8 Hz, 1H), 1.47 (dt, *J* = 13.7, 6.9 Hz, 1H), 1.38 (dt, *J* = 13.4, 6.7 Hz, 1H), 1.27 – 1.21 (m, 1H), 0.90 – 0.82 (m, 6H); ^{13}C NMR (126 MHz, DMSO-d_6) 164.46, 141.61, 134.59, 127.37, 126.17, 124.17, 120.72, 111.44, 111.40, 71.56, 47.03, 45.12, 24.02, 22.49, 22.30; HRMS (ESI$^-$) *m*/*z* [M–H]$^-$, calcd. for $C_{15}H_{18}N_4O_3B$: 313.1472, found: 313.1478; Purity by HPLC: 97%.

3-(2-Cyano-1*H*-1,3-benzodiazol-1-yl)propanoic acid (XXXI)

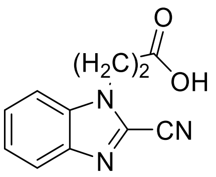

1*H*-1,3-Benzodiazole-2-carbonitrile (**XXIX**, 408 mg, 2.85 mmol, 1.0 equiv.) was dissolved in DMF (15 mL) and DIPEA (993 μL, 5.70 mmol, 2.0 equiv.). Ethyl 3-bromopropionate (534 μL, 4.28 mmol, 1.5 equiv.) was then added dropwise and the reaction mixture was stirred at 140 °C for 16 h. After the reaction was complete, it was diluted with THF (20 mL) and H_2O (30 mL). K_2CO_3 (788 mg, 5.70 mmol, 2.0 equiv.) was then added and the reaction mixture was allowed to stir at room temperature for 16 h. The mixture was concentrated, the residue re-dissolved in H_2O (80 mL), and washed with EtOAc (100 mL). The aqueous layer was acidified to pH 1 with 1 M HCl and extracted with EtOAc (3 × 80 mL). The combined organic layers were dried ($MgSO_4$), filtered, and the volatiles were removed under reduced pressure. The crude residue was purified by reversed-phase flash column chromatography using eluents A (0.1% HCOOH in MeCN) and B (0.1% HCOOH in H_2O) (gradient from 1:9 to 10:0). The product 3-(2-cyano-1*H*-1,3-benzodiazol-1-yl)propanoic acid (**XXXI**) was obtained as a yellow powder (150 mg, 24% yield). Mp.: 163–166 °C; ^{1}H NMR (500 MHz, DMSO-d_6) 7.81 (dd, *J* = 13.1, 8.3 Hz, 2H), 7.50 (dd, *J* = 11.4, 4.0 Hz, 1H), 7.40 (t, *J* = 7.4 Hz, 1H), 4.64 (t, *J* = 6.6 Hz, 2H), 2.91 (t, *J* = 6.6 Hz, 2H); ^{13}C NMR (126 MHz, DMSO-d_6) 171.72, 141.98, 133.68, 126.97, 126.07, 124.15, 120.78, 112.01, 111.57, 41.33, 33.24; HRMS (ESI$^+$) *m*/*z* [M+H]$^+$, calcd. for $C_{11}H_{10}N_3O_2$: 216.0773, found: 216.0768; Purity by HPLC: 99%.

3-(2-Cyano-1*H*-1,3-benzodiazol-1-yl)-*N*-[(1*R*)-3-methyl-1-[(1*S*,2*S*,6*R*,8*S*)-2,9,9-trimethyl-3,5-dioxa-4-boratricyclo[6.1.1.0^{2,6}]decan-4-yl]butyl]propanamide (30)

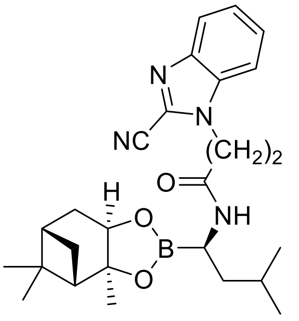

To a stirred solution of 3-(2-cyano-1*H*-1,3-benzodiazol-1-yl)propanoic acid (**XXXI**, 44 mg, 0.21 mmol, 1.0 equiv.), HATU (86 mg, 0.23 mmol, 1.1 equiv.) and DIPEA (79 µL, 0.45 mmol, 2.2 equiv.) in DMF (2.5 mL), (*R*)-BoroLeu-(+)-pinanediol trifluoroacetate (78 mg, 0.20 mmol, 1.0 equiv.) was added and the mixture was allowed to stir at room temperature for 2 h. The mixture was purified by reversed-phase flash column chromatography using eluents A (0.1% HCOOH in MeCN) and B (0.1% HCOOH in H_2O) (gradient from 1:9 to 10:0). The obtained viscous oil was triturated with Et_2O (20 mL) and concentrated. The product 3-(2-cyano-1*H*-1,3-benzodiazol-1-yl)-*N*-[(1*R*)-3-methyl-1-[(1*S*,2*S*,6*R*,8*S*)-2,9,9-trimethyl-3,5-dioxa-4-boratricyclo[6.1.1.0^{2,6}]decan-4-yl]butyl]propanamide (**30**) was obtained as a white powder (68 mg, 72% yield). Mp.: 52–55 °C; ^{1}H NMR (500 MHz, DMSO-*d*$_6$) 8.71 (s, 1H), 7.78 (t, *J* = 9.0 Hz, 2H), 7.48 (dd, *J* = 11.3, 4.1 Hz, 1H), 7.43 – 7.36 (m, 1H), 4.66 (t, *J* = 6.4 Hz, 2H), 4.08 (dd, *J* = 8.5, 1.8 Hz, 1H), 2.87 (t, *J* = 6.4 Hz, 2H), 2.46 (t, *J* = 6.0 Hz, 1H), 2.24 – 2.14 (m, 1H), 2.04 – 1.96 (m, 1H), 1.83 (t, *J* = 5.5 Hz, 1H), 1.80 – 1.74 (m, 1H), 1.59 (d, *J* = 13.9 Hz, 1H), 1.43 – 1.32 (m, 1H), 1.27 – 1.20 (m, 7H), 1.12 – 1.02 (m, 2H), 0.80 (s, *J* = 12.2 Hz, 3H), 0.72 (d, *J* = 6.4 Hz, 6H); ^{13}C NMR (126 MHz, DMSO-*d*$_6$) 171.26, 141.87, 133.76, 126.66, 125.95, 124.01, 120.60, 112.08, 111.46, 83.10, 75.74, 51.54, 41.43, 39.26, 37.61, 35.88, 32.24, 28.75, 27.06, 25.81, 24.66, 23.81, 22.99, 21.64; HRMS (ESI$^+$) *m/z* [M+H]$^+$, calcd. for $C_{26}H_{36}N_4O_3B$: 463.2880, found: 463.2888; Purity by HPLC: 100%.

[(1*R*)-1-[3-(2-Cyano-1*H*-1,3-benzodiazol-1-yl)propanamido]-3-methylbutyl]boronic acid (34)

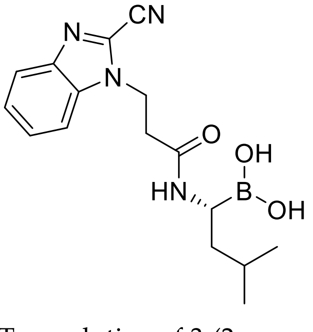

To a solution of 3-(2-cyano-1*H*-1,3-benzodiazol-1-yl)-*N*-[(1*R*)-3-methyl-1-[(1*S*,2*S*,6*R*,8*S*)-2,9,9-trimethyl-3,5-dioxa-4-boratricyclo[6.1.1.0^{2,6}]decan-4-yl]butyl]propanamide (**30**, 14 mg, 0.03 mmol, 1.0 equiv.) and 2-methyl-1-propylboronic acid (18 mg, 0.18 mmol, 6.0 equiv.) in MeOH (1.0 mL), 1 M HCl (100 µL) and *n*-hexane (1.0 mL) were added, and the biphasic reaction mixture was allowed to stir vigorously at room temperature for 16 h. The *n*-hexane layer was discarded, the MeOH layer washed again with *n*-hexane (3 × 1 mL), and finally concentrated. The residue was re-dissolved in diisopropyl ether (2.0

mL), sonicated for 15 min, followed by decanting and concentrating the ether. The residue was re-dissolved in MeCN (1.8 mL), sonicated for 15 min, decanted, and the MeCN solution was concentrated. The product [(1*R*)-1-[3-(2-cyano-1*H*-1,3-benzodiazol-1-yl)propanamido]-3-methylbutyl]boronic acid (**34**) was obtained as a yellow solid (5 mg, 51% yield). Mp.: 185–189 °C; ^{1}H NMR (500 MHz, DMSO-*d*$_6$) 8.14 (s, 1H), 7.77 (t, *J* = 8.2 Hz, 2H), 7.41 (dt, *J* = 14.1, 7.6 Hz, 2H), 4.80 – 4.56 (m, 2H), 3.17 (s, 1H), 2.82 (ddd, *J* = 43.4, 10.9, 5.0 Hz, 2H), 1.26 (dd, *J* = 12.9, 6.2 Hz, 1H), 1.03 (ddd, *J* = 48.3, 26.5, 20.1 Hz, 2H), 0.84 – 0.56 (m, 6H); ^{13}C NMR (126 MHz, DMSO-*d*$_6$) 163.00, 141.83, 133.82, 126.56, 126.03, 124.08, 120.60, 112.18, 111.44, 42.02, 41.51, 32.24, 24.94, 23.03, 22.23; HRMS (ESI$^-$) *m*/*z* [M–H]$^-$, calcd. for $C_{16}H_{20}N_4O_3B$: 327.1628, found: 327.1628; Purity by ^{1}H NMR: 97%.

4-(2-Cyano-1*H*-1,3-benzodiazol-1-yl)butanoic acid (XXXII)

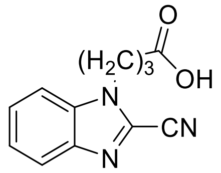

1*H*-1,3-Benzodiazole-2-carbonitrile (**XXIX**, 495 mg, 3.46 mmol, 1.0 equiv.) was dissolved in DMF (15 mL) and DIPEA (1205 µL, 6.92 mmol, 2.0 equiv.). Ethyl 4-bromobutyrate (834 µL, 6.23 mmol, 1.8 equiv.) was then added dropwise and the reaction mixture was stirred at 120 °C for 16 h. After the reaction was complete, it was diluted THF (20 mL) and H_2O (30 mL). K_2CO_3 (956 mg, 6.92 mmol, 2.0 equiv.) was then added and the reaction mixture was allowed to stir at room temperature for 16 h. The mixture was concentrated, the residue re-dissolved in H_2O (80 mL) and washed with EtOAc (100 mL). The aqueous layer was acidified to pH 1 with 1 M HCl and extracted with EtOAc (3 × 80 mL). The combined organic layers were dried ($MgSO_4$), filtered, and the volatiles were removed under reduced pressure. The crude residue was purified by reversed-phase flash column chromatography using eluents A (0.1% HCOOH in MeCN) and B (0.1% HCOOH in H_2O) (gradient from 1:9 to 10:0). The product 4-(2-cyano-1*H*-1,3-benzodiazol-1-yl)butanoic acid (**XXXII**) was obtained as a white powder (195 mg, 25% yield). Mp.: 132–134 °C; ^{1}H NMR (500 MHz, DMSO-*d*$_6$) 12.18 (s, 1H), 7.81 (d, *J* = 7.8 Hz, 2H), 7.51 (dd, *J* = 10.5, 4.9 Hz, 1H), 7.41 (dd, *J* = 10.2, 5.1 Hz, 1H), 4.47 (dd, *J* = 9.3, 4.5 Hz, 2H), 2.36 – 2.28 (m, 2H), 2.13 – 2.02 (m, 2H); ^{13}C NMR (126 MHz, DMSO-*d*$_6$) 173.40, 141.95, 134.11, 126.15, 124.17, 120.83, 111.68, 111.45, 44.41, 30.42, 24.78; HRMS (ESI$^+$) *m*/*z* [M+H]$^+$, calcd. for $C_{12}H_{12}N_3O_2$: 230.0930, found: 230.0928; Purity by HPLC: 98%.

4-(2-Cyano-1*H*-1,3-benzodiazol-1-yl)-*N*-[(1*R*)-3-methyl-1-[(1*S*,2*S*,6*R*,8*S*)-2,9,9-trimethyl-3,5-dioxa-4-boratricyclo[6.1.1.0^{2,6}]decan-4-yl]butyl]butanamide (31)

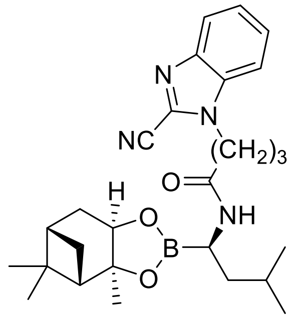

To a stirred solution of 4-(2-cyano-1*H*-1,3-benzodiazol-1-yl)butanoic acid (**XXXII**, 92 mg, 0.40 mmol, 1.0 equiv.), HATU (167 mg, 0.44 mmol, 1.1 equiv.) and DIPEA (153 μL, 0.88 mmol, 2.2 equiv.) in DMF (2.5 mL), (*R*)-BoroLeu-(+)-pinanediol trifluoroacetate (152 mg, 0.40 mmol, 1.0 equiv.) was added and the mixture was allowed to stir at room temperature for 2 h. The mixture was purified by reversed-phase flash column chromatography using eluents A (0.1% HCOOH in MeCN) and B (0.1% HCOOH in H_2O) (gradient from 1:9 to 10:0). The obtained viscous oil was triturated with Et_2O (20 mL) and concentrated. The product 4-(2-cyano-1*H*-1,3-benzodiazol-1-yl)-*N*-[(1*R*)-3-methyl-1-[(1*S*,2*S*,6*R*,8*S*)-2,9,9-trimethyl-3,5-dioxa-4-boratricyclo[6.1.1.0^{2,6}]decan-4-yl]butyl]butanamide (**31**) was obtained as a white powder (156 mg, 82% yield). Mp.: 72–74 °C; ^{1}H NMR (500 MHz, DMSO-d_6) 9.14 (s, 1H), 7.83 (d, *J* = 8.9 Hz, 2H), 7.51 (t, *J* = 8.1 Hz, 1H), 7.43 (t, *J* = 7.9 Hz, 1H), 4.47 (t, *J* = 7.2 Hz, 2H), 4.04 (dd, *J* = 8.5, 1.9 Hz, 1H), 2.45 (t, *J* = 7.1 Hz, 1H), 2.37 – 2.31 (m, 2H), 2.23 – 2.15 (m, 1H), 2.13 – 2.06 (m, 2H), 1.99 (dt, *J* = 6.3, 5.3 Hz, 1H), 1.82 (t, *J* = 5.6 Hz, 1H), 1.80 – 1.75 (m, 1H), 1.70 – 1.59 (m, 2H), 1.37 (d, *J* = 9.9 Hz, 1H), 1.27 – 1.19 (m, 8H), 0.82 (dt, *J* = 11.2, 5.5 Hz, 9H); ^{13}C NMR (126 MHz, DMSO-d_6) 174.69, 141.93, 134.11, 126.13, 124.23, 120.85, 111.70, 111.47, 82.13, 75.36, 51.96, 44.28, 40.53, 37.62, 36.50, 29.19, 28.01, 27.19, 25.95, 25.17, 24.98, 23.94, 23.16, 21.85; HRMS (ESI$^+$) *m/z* [M+H]$^+$, calcd. for $C_{27}H_{38}N_4O_3B$: 477.3037, found: 477.3030; Purity by HPLC: 99%.

[(1*R*)-1-[4-(2-Cyano-1*H*-1,3-benzodiazol-1-yl)butanamido]-3-methylbutyl]boronic acid (35)

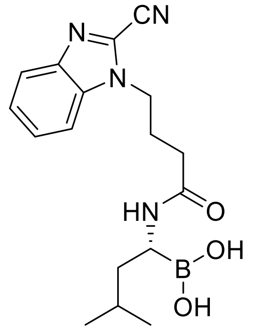

To a solution of 4-(2-cyano-1*H*-1,3-benzodiazol-1-yl)-*N*-[(1*R*)-3-methyl-1-[(1*S*,2*S*,6*R*,8*S*)-2,9,9-trimethyl-3,5-dioxa-4-boratricyclo[6.1.1.0^{2,6}]decan-4-yl]butyl]butanamide (**31**, 26 mg, 0.055 mmol, 1.0 equiv.) in diisopropyl ether (2.0 mL), boric acid (10 mg, 0.17 mmol, 3.0 equiv.) and concentrated HCl (120 μL) were added, and the reaction mixture was allowed to stir vigorously at room temperature for 16 h. It was then diluted with diisopropyl ether (1.5 mL), sonicated, and the ether layer decanted. The residue was washed with diisopropyl ether (2 × 2 mL), re-dissolved in MeCN (2.0 mL), sonicated for 15 min, decanted, and the MeCN solution was concentrated. The product [(1*R*)-1-[4-(2-cyano-1*H*-1,3-benzodiazol-1-yl)butanamido]-3-methylbutyl]boronic acid (**35**) was obtained as a white solid (8 mg, 43% yield). Mp.: 215–220 °C; ^{1}H NMR (500 MHz, DMSO-d_6) 10.15 (s, *J* = 5.2 Hz, 1H), 7.83 (d, *J* = 8.5 Hz, 2H), 7.53 (t, *J* = 7.8 Hz, 1H), 7.48 – 7.38 (m, 1H), 4.66 (t, *J* = 6.8 Hz, 1H), 4.48 (t, *J* = 6.9 Hz, 2H), 2.48 – 2.44 (m, 1H), 2.36 (t, *J* = 7.5 Hz, 1H), 2.12 (dt, *J* = 11.7, 7.6 Hz, 2H), 1.54 (dt, *J* = 13.0, 6.5 Hz, 1H), 1.25 – 1.13 (m, 2H), 0.87 – 0.61 (m, 6H); ^{13}C NMR (126 MHz, DMSO-d_6) 176.85, 142.01, 134.14, 126.40, 126.26, 124.38, 120.94, 111.83, 111.59, 46.64, 44.18, 26.47, 25.65, 24.97, 23.24, 21.96; HRMS (ESI$^-$) *m/z* [M–H]$^-$, calcd. for $C_{17}H_{22}N_4O_3B$: 341.1785, found: 341.1792; Purity by ^{1}H NMR: 97%.

5-(2-Cyano-1*H*-1,3-benzodiazol-1-yl)pentanoic acid (XXXIII)

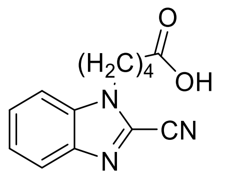

1*H*-1,3-Benzodiazole-2-carbonitrile (**XXIX**, 429 mg, 3.00 mmol, 1.0 equiv.) was dissolved in DMF (15 mL) and DIPEA (1045 µL, 6.00 mmol, 2.0 equiv.). Ethyl 5-bromovalerate (855 µL, 5.40 mmol, 1.8 equiv.) was then added dropwise and the reaction mixture was stirred reflux for 3 h. After the reaction was complete, it was diluted with THF (20 mL) and H_2O (60 mL). K_2CO_3 (1659 mg, 12.00 mmol, 4.0 equiv.) was then added and the reaction mixture was allowed to stir at room temperature for 16 h. The mixture was concentrated, the residue was re-dissolved in H_2O (80 mL) and washed with EtOAc (100 mL). The aqueous layer was acidified to pH 1 with 1 M HCl and extracted with EtOAc (3 × 80 mL). The combined organic layers were dried ($MgSO_4$), filtered, and the volatiles were removed under reduced pressure. The crude residue was purified by reversed-phase flash column chromatography using eluents A (0.1% HCOOH in MeCN) and B (0.1% HCOOH in H_2O) (gradient from 1:9 to 10:0). The product 5-(2-cyano-1*H*-1,3-benzodiazol-1-yl)pentanoic acid (**XXXIII**) was obtained as a white powder (206 mg, 28% yield). Mp.: 104–105 °C; ^{1}H NMR (500 MHz, DMSO-*d*$_6$) 12.02 (s, 1H), 7.81 (d, *J* = 8.5 Hz, 2H), 7.51 (t, *J* = 7.7 Hz, 1H), 7.41 (t, *J* = 7.8 Hz, 1H), 4.47 (t, *J* = 7.0 Hz, 2H), 2.29 – 2.22 (m, 2H), 1.91 – 1.82 (m, 2H), 1.49 (dt, *J* = 10.5, 7.5 Hz, 2H); ^{13}C NMR (126 MHz, DMSO-*d*$_6$) 173.98, 141.93, 134.12, 126.15, 126.10, 124.18, 120.82, 111.80, 111.56, 44.87, 32.92, 28.87, 21.59; HRMS (ESI$^+$) *m/z* [M+H]$^+$, calcd. for $C_{13}H_{14}N_3O_2$: 244.1086, found: 244.1083; Purity by HPLC: 98%.

5-(2-Cyano-1*H*-1,3-benzodiazol-1-yl)-*N*-[(1*R*)-3-methyl-1-[(1*S*,2*S*,6*R*,8*S*)-2,9,9-trimethyl-3,5-dioxa-4-boratricyclo[6.1.1.0^{2,6}]decan-4-yl]butyl]pentanamide (32)

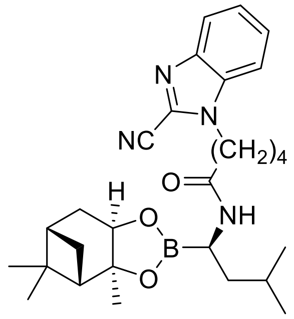

To a stirred solution of 5-(2-cyano-1*H*-1,3-benzodiazol-1-yl)pentanoic acid (**XXXIII**, 110 mg, 0.45 mmol, 1.0 equiv.), HATU (188 mg, 0.50 mmol, 1.1 equiv.) and DIPEA (172 µL, 0.99 mmol, 2.2 equiv.) in DMF (2.5 mL), (*R*)-BoroLeu-(+)-pinanediol trifluoroacetate (171 mg, 0.45 mmol, 1.0 equiv.) was added and the mixture was allowed to stir at room temperature for 2 h. The mixture was purified by reversed-phase flash column chromatography using eluents A (0.1% HCOOH in MeCN) and B (0.1% HCOOH in H_2O) (gradient from 1:9 to 10:0). The obtained viscous oil was triturated with Et_2O (20 mL) and concentrated. The product 5-(2-cyano-1*H*-1,3-benzodiazol-1-yl)-*N*-[(1*R*)-3-methyl-1-[(*SR*,2*S*,6*R*,8*S*)-2,9,9-trimethyl-3,5-dioxa-4-boratricyclo[6.1.1.0^{2,6}]decan-4-yl]butyl]pentanamide (**32**) was obtained as a light brown powder (183 mg, 83% yield). Mp.: 147–149 °C; ^{1}H NMR (500 MHz, DMSO-*d*$_6$) 9.24 (s, 1H), 7.82 (dd, *J* = 8.3, 2.5 Hz, 2H), 7.50 (t, *J* = 7.8 Hz, 1H), 7.42 (t, *J* = 7.6 Hz, 1H), 4.47 (t, *J* = 7.1 Hz, 2H), 3.94 (dd, *J* = 8.5, 1.9 Hz, 1H), 2.42 – 2.36 (m, 1H), 2.31 (t, *J* = 7.3 Hz, 2H), 2.19 – 2.11

(m, 1H), 1.98 – 1.90 (m, 1H), 1.89 – 1.81 (m, 2H), 1.80 – 1.72 (m, 2H), 1.65 (td, *J* = 13.4, 6.7 Hz, 1H), 1.60 – 1.51 (m, 3H), 1.34 (t, *J* = 8.3 Hz, 1H), 1.23 – 1.16 (m, 8H), 0.83 (dd, *J* = 6.6, 3.0 Hz, 6H), 0.79 (s, 3H); ^{13}C NMR (126 MHz, DMSO-d_6) 175.71, 141.92, 134.07, 126.08, 126.04, 124.15, 120.78, 111.80, 111.49, 81.73, 75.16, 52.04, 44.76, 40.70, 37.57, 36.61, 30.14, 29.20, 28.79, 27.22, 25.92, 24.98, 23.94, 23.11, 22.06, 21.95; HRMS (ESI$^+$) *m/z* [M+H]$^+$, calcd. for $C_{28}H_{40}N_4O_3B$: 491.3193, found: 491.3194; Purity by HPLC: 98%.

[(1*R*)-1-[5-(2-Cyano-1*H*-1,3-benzodiazol-1-yl)pentanamido]-3-methylbutyl]boronic acid (36)

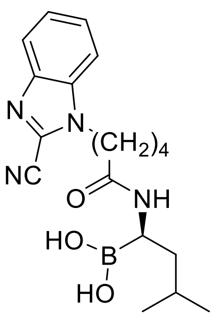

To a solution of 5-(2-cyano-1*H*-1,3-benzodiazol-1-yl)-*N*-[(1*R*)-3-methyl-1-[(1*S*,2*S*,6*R*,8*S*)-2,9,9-trimethyl-3,5-dioxa-4-boratricyclo[6.1.1.0^{2,6}]decan-4-yl]butyl]pentanamide (**32**, 39 mg, 0.08 mmol, 1.0 equiv.) and 2-methyl-1-propylboronic acid (25 mg, 0.24 mmol, 3.0 equiv.) in MeOH (1.0 mL), 1 M HCl (240 μL) and *n*-hexane (1.0 mL) were added, and the biphasic reaction mixture was allowed to stir vigorously at room temperature for 16 h. The *n*-hexane layer was discarded, the MeOH layer washed again with *n*-hexane (3 × 1 mL), and finally concentrated. The residue was re-dissolved in diisopropyl ether (2.0 mL), sonicated for 15 min, followed by decanting and concentrating the ether. The residue was re-dissolved in MeCN (1.8 mL), sonicated for 15 min, decanted, and the MeCN solution was concentrated. The product [(1*R*)-1-[5-(2-cyano-1*H*-1,3-benzodiazol-1-yl)pentanamido]-3-methylbutyl]boronic acid (**36**) was obtained as a white solid (18 mg, 63%). Mp.: 95–98 °C; ^{1}H NMR (500 MHz, DMSO-d_6) 7.82 (dt, *J* = 12.6, 8.1 Hz, 2H), 7.51 (dt, *J* = 15.4, 7.7 Hz, 1H), 7.41 (ddd, *J* = 14.0, 7.9, 5.1 Hz, 1H), 4.46 (dt, *J* = 17.8, 7.1 Hz, 2H), 2.56 – 2.51 (m, 1H), 2.43 (s, 1H), 2.35 (d, *J* = 15.3 Hz, 1H), 1.92 – 1.79 (m, 2H), 1.53 (ddd, *J* = 15.6, 12.5, 7.5 Hz, 3H), 1.27 – 1.11 (m, 2H), 0.85 – 0.79 (m, 3H), 0.76 (dd, *J* = 6.5, 3.9 Hz, 3H); ^{13}C NMR (126 MHz, DMSO-d_6) 141.94, 134.11, 134.05, 126.18, 126.13, 124.18, 120.81, 111.81, 111.52, 44.73, 28.74, 25.24, 25.02, 23.17, 22.97, 22.29, 21.86; HRMS (ESI$^-$) *m/z* [M–H]$^-$, calcd. for $C_{18}H_{24}N_4O_3B$: 355.1941, found: 355.1936; Purity by ^{1}H NMR: 99%.

3-[(5-Chloro-2-nitrophenyl)amino]propanoic acid (XXXVI)

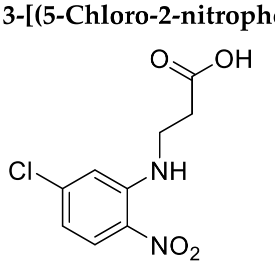

To a stirred solution of 4-chloro-2-fluoronitrobenzene (**XXXIV**, 488 mg, 2.78 mmol, 1.0 equiv.) in DMF (4.0 mL) and DIPEA (1453 μL, 8.34 mmol, 3.0 equiv.), ethyl 3-aminopropanoate hydrochloride (512 mg, 3.34 mmol, 1.2 equiv.) was added and the mixture was allowed to stir at room temperature for 16 h. The mixture was diluted with DMF (4 mL), THF (10 mL) and H_2O (20 mL), followed by the addition

of 10% NaOH (8.0 mL). The reaction mixture was stirred at 70 °C for 2 h. After the reaction was complete, THF was evaporated, the remaining suspension diluted with H_2O (60 mL), the pH adjusted to 1 with 10% HCl, and finally extracted with EtOAc (3 × 60 mL). The combined organic layers were dried ($MgSO_4$), volatiles evaporated and the product was purified by reversed-phase flash column chromatography using eluents A (0.1% HCOOH in MeCN) and B (0.1% HCOOH in H_2O) (gradient from 1:9 to 10:0). The product 3-[(5-chloro-2-nitrophenyl)amino]propanoic acid (**XXXVI**) was obtained as a yellow solid (597 mg, 88% yield). Mp.: 150–152 °C; ^{1}H NMR (500 MHz, DMSO-d_6) 12.40 (s, 1H), 8.28 (t, *J* = 5.5 Hz, 1H), 8.06 (d, *J* = 9.1 Hz, 1H), 7.16 (d, *J* = 1.5 Hz, 1H), 6.70 (dd, *J* = 9.1, 1.9 Hz, 1H), 3.58 (q, *J* = 6.3 Hz, 2H), 2.61 (t, *J* = 6.6 Hz, 2H); ^{13}C NMR (126 MHz, DMSO-d_6) 172.89, 145.36, 141.70, 130.08, 128.23, 115.42, 113.44, 38.38, 33.16; HRMS (ESI$^-$) *m/z* [M–H]$^-$, calcd. for $C_9H_8N_2O_4Cl$: 243.0173, found: 243.0174; Purity by HPLC: 99%.

3-[(5-Chloro-2-nitrophenyl)amino]-*N*-[(1*R*)-3-methyl-1-[(1*S*,2*S*,6*R*,8*S*)-2,9,9-trimethyl-3,5-dioxa-4-boratricyclo[6.1.1.0^{2,6}]decan-4-yl]butyl]propanamide (XXXVIII)

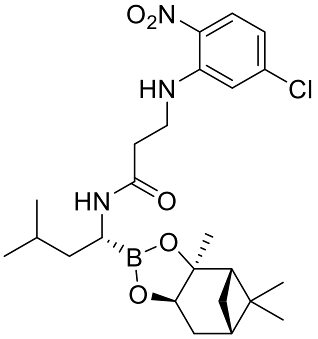

To a stirred solution of 3-[(5-chloro-2-nitrophenyl)amino]propanoic acid (**XXXVI**, 196 mg, 0.80 mmol, 1.0 equiv.), HATU (335 mg, 0.88 mmol, 1.1 equiv.) and DIPEA (307 μL, 1.76 mmol, 2.2 equiv.) in DMF (2.5 mL), (*R*)-BoroLeu-(+)-pinanediol trifluoroacetate (303 mg, 0.80 mmol, 1.0 equiv.) was added and the mixture was allowed to stir at room temperature for 2 h. The mixture was purified by reversed-phase flash column chromatography using eluents A (0.1% HCOOH in MeCN) and B (0.1% HCOOH in H_2O) (gradient from 1:9 to 10:0). The product 3-[(5-chloro-2-nitrophenyl)amino]-*N*-[(1*R*)-3-methyl-1-[(1*S*,2*S*,6*R*,8*S*)-2,9,9-trimethyl-3,5-dioxa-4-boratricyclo[6.1.1.0^{2,6}]decan-4-yl]butyl]propanamide (**XXXVIII**) was obtained as a yellow solid (362 mg, 92% yield). Mp.: 58–60 °C; ^{1}H NMR (500 MHz, DMSO-d_6) 9.11 (s, 1H), 8.27 (t, *J* = 5.8 Hz, 1H), 8.08 (d, *J* = 9.1 Hz, 1H), 7.16 (d, *J* = 2.0 Hz, 1H), 6.72 (dd, *J* = 9.1, 2.0 Hz, 1H), 4.06 – 4.00 (m, 1H), 3.61 (q, *J* = 6.4 Hz, 2H), 2.64 – 2.57 (m, 2H), 2.21 – 2.13 (m, 1H), 2.02 – 1.93 (m, 1H), 1.80 (t, *J* = 5.6 Hz, 1H), 1.78 – 1.74 (m, 1H), 1.62 (td, *J* = 14.2, 9.4 Hz, 2H), 1.33 (d, *J* = 10.0 Hz, 1H), 1.30 – 1.16 (m, 9H), 0.83 (dd, *J* = 6.6, 2.1 Hz, 6H), 0.79 (s, 3H); ^{13}C NMR (126 MHz, DMSO-d_6) 173.37, 145.24, 141.62, 130.13, 128.17, 115.48, 113.50, 82.45, 75.49, 51.80, 40.44, 38.70, 37.60, 36.24, 31.06, 28.95, 27.13, 25.89, 24.92, 23.88, 23.09, 21.86; HRMS (ESI$^+$) *m/z* [M+H]$^+$, calcd. for $C_{24}H_{36}N_3O_5BCl$: 492.2437, found: 492.2437; Purity by HPLC: 99%.

3-[(2-Amino-5-chlorophenyl)amino]-*N*-[(1*R*)-3-methyl-1-[(1*S*,2*S*,6*R*,8*S*)-2,9,9-trimethyl-3,5-dioxa-4-boratricyclo[6.1.1.0^{2,6}]decan-4-yl]butyl]propanamide (XL)

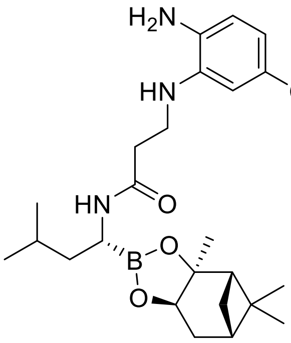

To a solution of 3-[(2-amino-5-chlorophenyl)amino]-*N*-[(1*R*)-3-methyl-1-[(1*S*,2*S*,6*R*,8*S*)-2,9,9-trimethyl-3,5-dioxa-4-boratricyclo[6.1.1.0^{2,6}]decan-4-yl]butyl]propanamide (**XL**, 246 mg, 0.50 mmol, 1.0 equiv.) in EtOAc (25 mL), $SnCl_2 \times 2H_2O$ (677 mg, 3.00 mmol, 6.0 equiv.) was added and the reaction was allowed to proceed at 70 °C for 2 h. Then, another portion of $SnCl_2 \times 2H_2O$ (451 mg, 2.00 mmol, 4.0 equiv.) was added and the reaction was allowed to stir at 70 °C for additional 16 h. The formerly yellow reaction mixture became colourless. It was then diluted with EtOAc (100 mL) and washed with saturated aqueous solution of $NaHCO_3$ (2 × 100 mL) and brine (50 mL). The volatiles were evaporated and the mixture was purified by reversed-phase flash column chromatography using eluents A (0.1% HCOOH in MeCN) and B (0.1% HCOOH in H_2O) (gradient from 1:9 to 10:0). The product 3-[(2-amino-5-chlorophenyl)amino]-*N*-[(1*R*)-3-methyl-1-[(1*S*,2*S*,6*R*,8*S*)-2,9,9-trimethyl-3,5-dioxa-4-boratricyclo[6.1.1.0^{2,6}]decan-4-yl]butyl]propanamide (**XL**) was obtained as a white solid (193 mg, 84% yield). Mp.: 59–61 °C; ^{1}H NMR (500 MHz, DMSO-*d$_6$*) 9.29 (s, 1H), 6.53 (d, *J* = 8.0 Hz, 1H), 6.43 (dt, *J* = 4.9, 2.2 Hz, 2H), 4.77 (s, 2H), 4.02 (dd, *J* = 8.5, 1.9 Hz, 1H), 3.27 (t, *J* = 6.9 Hz, 2H), 2.56 (t, *J* = 7.0 Hz, 2H), 2.45 (t, *J* = 7.3 Hz, 1H), 2.22 – 2.14 (m, 1H), 2.08 – 2.05 (m, 2H), 2.02 – 1.95 (m, 1H), 1.82 (t, *J* = 5.6 Hz, 1H), 1.80 – 1.75 (m, 1H), 1.69 (dq, *J* = 13.0, 6.6 Hz, 1H), 1.63 (d, *J* = 13.8 Hz, 1H), 1.39 (d, *J* = 9.9 Hz, 1H), 1.27 – 1.21 (m, 8H), 0.86 (dd, *J* = 6.4, 4.8 Hz, 6H), 0.81 (s, 3H); ^{13}C NMR (126 MHz, DMSO-*d$_6$*) 74.59, 136.69, 134.46, 121.08, 116.28, 114.65, 109.44, 81.91, 75.25, 52.05, 40.67, 39.27, 37.61, 36.59, 31.08, 29.26, 27.22, 25.98, 25.02, 23.96, 23.14, 22.00; HRMS (ESI$^+$) *m/z* [M+H]$^+$, calcd. for $C_{24}H_{38}N_3O_3BCl$: 462.2695, found: 462.2691; Purity by HPLC: 98%.

3-(6-Chloro-2-cyano-1*H*-1,3-benzodiazol-1-yl)-*N*-[(1*R*)-3-methyl-1-[(1*S*,2*S*,6*R*,8*S*)-2,9,9-trimethyl-3,5-dioxa-4-boratricyclo[6.1.1.0^{2,6}]decan-4-yl]butyl]propanamide (37)

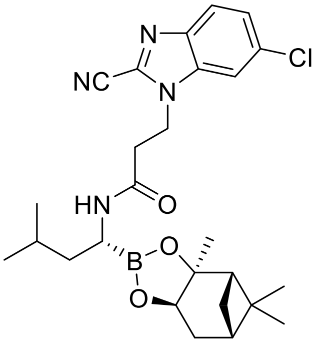

To a solution of 3-[(2-amino-5-chlorophenyl)amino]-*N*-[(1*R*)-3-methyl-1-[(1*S*,2*S*,6*R*,8*S*)-2,9,9-trimethyl-3,5-dioxa-4-boratricyclo[6.1.1.0^{2,6}]decan-4-yl]butyl]propanamide (**XL**, 46 mg, 0.10 mmol, 1.0 equiv.) in pyridine (800 μL), Appel's salt (21 mg, 0.10 mmol, 1.0 equiv.) was added. The reaction mixture was allowed to stir first at room temperature for 1 h and then at 50 °C for 3 h. After the reaction was

complete, the product was purified by reversed-phase flash column chromatography using eluents A (0.1% HCOOH in MeCN) and B (0.1% HCOOH in H_2O) (gradient from 1:9 to 10:0). The product 3-(6-chloro-2-cyano-1*H*-1,3-benzodiazol-1-yl)-*N*-[(1*R*)-3-methyl-1-[(1*S*,2*S*,6*R*,8*S*)-2,9,9-trimethyl-3,5-dioxa-4-boratricyclo[6.1.1.0^{2,6}]decan-4-yl]butyl]propanamide (**37**) was obtained as a brown solid (21 mg, 42% yield).

Mp.: 172–175 °C; ^{1}H NMR (500 MHz, DMSO-*d*$_6$) 8.68 (s, 1H), 7.94 (d, *J* = 1.8 Hz, 1H), 7.80 (d, *J* = 8.8 Hz, 1H), 7.41 (dd, *J* = 8.8, 1.9 Hz, 1H), 4.64 (t, *J* = 6.4 Hz, 2H), 4.06 (d, *J* = 6.8 Hz, 1H), 2.87 (t, *J* = 6.3 Hz, 2H), 2.49 – 2.46 (m, 1H), 2.21 – 2.13 (m, 1H), 2.02 – 1.95 (m, 1H), 1.82 (t, *J* = 5.6 Hz, 1H), 1.77 (s, 1H), 1.57 (d, *J* = 14.1 Hz, 1H), 1.34 (dd, *J* = 13.6, 6.6 Hz, 1H), 1.23 – 1.19 (m, 7H), 1.07 (dt, *J* = 14.8, 7.2 Hz, 2H), 0.79 (s, 3H), 0.72 (d, *J* = 6.6 Hz, 6H); ^{13}C NMR (126 MHz, DMSO-*d*$_6$) 171.10, 140.52, 134.47, 130.81, 127.75, 124.69, 121.99, 112.17, 111.15, 83.19, 75.78, 51.50, 41.65, 39.90, 37.62, 35.83, 32.18, 28.70, 27.06, 25.80, 24.68, 23.81, 22.98, 21.67; HRMS (ESI$^+$) *m*/*z* [M+H]$^+$, calcd. for $C_{26}H_{35}N_4O_3BCl$: 497.2491, found: 497.2497; Purity by HPLC: 99%.

3-[(2-Chloro-6-nitrophenyl)amino]propanoic acid (XXXVII)

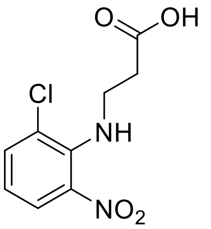

To a stirred solution of 1-chloro-2-fluoro-3-nitrobenzene (**XXXV**, 560 mg, 3.19 mmol, 1.0 equiv.) in DMF (4.0 mL) and DIPEA (1667 μL, 9.57 mmol, 3.0 equiv.), ethyl 3-aminopropanoate hydrochloride (588 mg, 3.83 mmol, 1.2 equiv.) was added and the mixture was allowed to stir at room temperature for 16 h. The mixture was diluted with DMF (4 mL), THF (10 mL), and H_2O (20 mL), followed by the addition of 10% NaOH (8.0 mL). The reaction mixture was allowed to proceed at 70 °C for 2 h. After the reaction was complete, THF was evaporated, the remaining suspension diluted with H_2O (60 mL), pH adjusted to 1 with 10% HCl, and then extracted with EtOAc (3 × 60 mL). The combined organic layers were dried ($MgSO_4$), volatiles evaporated and the product was purified by reversed-phase flash column chromatography using eluents A (0.1% HCOOH in MeCN) and B (0.1% HCOOH in H_2O) (gradient from 1:9 to 10:0). The product 3-[(2-chloro-6-nitrophenyl)amino]propanoic acid (**XXXVII**) was obtained as a yellow solid (580 mg, 74% yield). Mp.: 65–68 °C; ^{1}H NMR (500 MHz, DMSO-*d*$_6$) 12.36 (s, 1H), 7.85 (dd, *J* = 8.4, 1.4 Hz, 1H), 7.67 (dd, *J* = 7.8, 1.4 Hz, 1H), 6.88 (t, *J* = 8.1 Hz, 1H), 6.51 (t, *J* = 5.9 Hz, 1H), 3.37 (q, *J* = 6.3 Hz, 2H), 2.54 (t, *J* = 6.4 Hz, 2H); ^{13}C NMR (126 MHz, DMSO-*d*$_6$) 172.87, 140.99, 138.82, 135.46, 125.15, 123.92, 118.26, 42.54, 34.49; HRMS (ESI$^+$) *m*/*z* [M+H]$^+$, calcd. for $C_9H_{10}N_2O_4Cl$: 245.0329, found: 245.0324; Purity by HPLC: 99%.

3-[(2-Chloro-6-nitrophenyl)amino]-*N*-[(1*R*)-3-methyl-1-[(1*S*,2*S*,6*R*,8*S*)-2,9,9-trimethyl-3,5-dioxa-4-boratricyclo[6.1.1.0^{2,6}]decan-4-yl]butyl]propanamide (XXXIX)

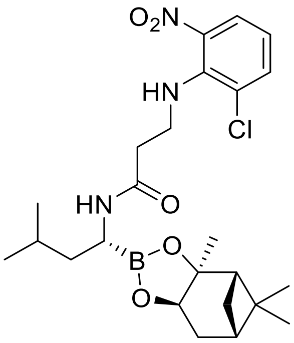

To a stirred solution of 3-[(2-chloro-6-nitrophenyl)amino]propanoic acid (**XXXVII**, 269 mg, 1.10 mmol, 1.0 equiv.), HATU (460 mg, 1.21 mmol, 1.1 equiv.) and DIPEA (422 µL, 2.42 mmol, 2.2 equiv.) in DMF (2.5 mL), (*R*)-BoroLeu-(+)-pinanediol trifluoroacetate (417 mg, 1.10 mmol, 1.0 equiv.) was added and the mixture was allowed to stir at room temperature for 2 h. The mixture was purified by reversed-phase flash column chromatography using eluents A (0.1% HCOOH in MeCN) and B (0.1% HCOOH in H_2O) (gradient from 1:9 to 10:0). The product 3-[(2-chloro-6-nitrophenyl)amino]-*N*-[(1*R*)-3-methyl-1-[(1*S*,2*S*,6*R*,8*S*)-2,9,9-trimethyl-3,5-dioxa-4-boratricyclo[6.1.1.0^{2,6}]decan-4-yl]butyl]propanamide (**XXXIX**) was obtained as a yellow solid (457 mg, 84% yield). Mp.: 145–146 °C; ^{1}H NMR (500 MHz, DMSO-d_6) 9.08 (s, 1H), 7.85 (d, *J* = 8.4 Hz, 1H), 7.67 (d, *J* = 7.8 Hz, 1H), 6.88 (t, *J* = 8.1 Hz, 1H), 6.51 (t, *J* = 5.7 Hz, 1H), 4.00 (d, *J* = 7.4 Hz, 1H), 3.38 (q, *J* = 6.2 Hz, 2H), 2.56 (t, *J* = 6.4 Hz, 2H), 2.46 (d, *J* = 6.9 Hz, 1H), 2.21 – 2.12 (m, 1H), 2.00 – 1.93 (m, 1H), 1.80 (t, *J* = 5.5 Hz, 1H), 1.76 (d, *J* = 5.2 Hz, 1H), 1.64 (ddd, *J* = 23.7, 14.8, 10.2 Hz, 2H), 1.32 (d, *J* = 10.0 Hz, 1H), 1.26 – 1.16 (m, 8H), 0.84 (t, *J* = 6.5 Hz, 6H), 0.79 (s, 3H); ^{13}C NMR (126 MHz, DMSO-d_6) 173.48, 140.69, 138.50, 135.33, 125.17, 123.75, 118.15, 82.35, 75.45, 51.81, 42.79, 40.41, 37.59, 36.27, 32.31, 28.96, 27.14, 25.89, 24.92, 23.89, 23.11, 21.88; HRMS (ESI$^+$) *m/z* [M+H]$^+$, calcd. for $C_{24}H_{36}N_3O_5BCl$: 492.2437, found: 492.2440; Purity by HPLC: 99%.

3-[(2-Amino-6-chlorophenyl)amino]-*N*-[(1*R*)-3-methyl-1-[(1*S*,2*S*,6*R*,8*S*)-2,9,9-trimethyl-3,5-dioxa-4-boratricyclo[6.1.1.0^{2,6}]decan-4-yl]butyl]propanamide (XLI)

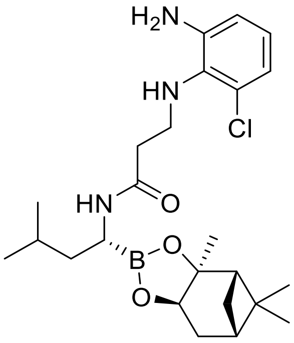

To a solution of 3-[(2-chloro-6-nitrophenyl)amino]-*N*-[(1*R*)-3-methyl-1-[(1*S*,2*S*,6*R*,8*S*)-2,9,9-trimethyl-3,5-dioxa-4-boratricyclo[6.1.1.0^{2,6}]decan-4-yl]butyl]propanamide (**XXXIX**, 271 mg, 0.55 mmol, 1.0 equiv.) in EtOAc (25 mL), $SnCl_2$ × $2H_2O$ (745 mg, 3.30 mmol, 6.0 equiv.) was added and the reaction was allowed to proceed at 70 °C for 2 h. Then, another portion of $SnCl_2$ × $2H_2O$ (496 mg, 2.20 mmol, 4.0 equiv.) was added and the reaction was allowed to stir at 70 °C for additional 16 h. The formerly yellow reaction mixture became colourless. It was then diluted with EtOAc (100 mL), washed with saturated aqueous solution of $NaHCO_3$ (2 × 100 mL) and brine (50 mL). The volatiles were evaporated and the mixture was purified by reversed-phase flash column chromatography using eluents A (0.1%

HCOOH in MeCN) and B (0.1% HCOOH in H_2O) (gradient from 1:9 to 10:0). The product 3-[(2-amino-6-chlorophenyl)amino]-*N*-[(1*R*)-3-methyl-1-[(1*S*,2*S*,6*R*,8*S*)-2,9,9-trimethyl-3,5-dioxa-4-boratricyclo[6.1.1.0^{2,6}]decan-4-yl]butyl]propanamide (**XLI**) was obtained as a white solid (224 mg, 88% yield). Mp.: 57–59 °C; ^{1}H NMR (500 MHz, DMSO-*d*$_6$) 9.29 (s, 1H), 6.73 (t, *J* = 7.9 Hz, 1H), 6.61 (dd, *J* = 8.0, 1.2 Hz, 1H), 6.56 (dd, *J* = 7.9, 1.2 Hz, 1H), 5.13 (s, 1H), 4.02 (dd, *J* = 8.5, 1.8 Hz, 1H), 3.11 – 3.03 (m, 2H), 2.58 – 2.51 (m, 2H), 2.47 – 2.42 (m, 1H), 2.22 – 2.15 (m, 1H), 1.99 (dd, *J* = 13.2, 7.1 Hz, 1H), 1.81 (ddd, *J* = 18.6, 8.2, 3.9 Hz, 2H), 1.73 – 1.66 (m, 1H), 1.62 (d, *J* = 13.7 Hz, 1H), 1.40 – 1.35 (m, 1H), 1.30 – 1.20 (m, 9H), 0.86 (t, *J* = 6.0 Hz, 6H), 0.82 (s, 3H); ^{13}C NMR (126 MHz, DMSO-*d*$_6$) 175.15, 144.25, 129.94, 127.81, 124.12, 116.26, 113.35, 81.84, 75.22, 52.05, 41.51, 40.71, 37.60, 36.64, 31.78, 29.28, 27.23, 26.00, 25.03, 23.97, 23.19, 21.90; HRMS (ESI$^+$) *m*/*z* [M+H]$^+$, calcd. for $C_{24}H_{38}N_3O_3BCl$: 462.2695, found: 462.2685; Purity by HPLC: 98%.

3-(7-Chloro-2-cyano-1*H*-1,3-benzodiazol-1-yl)-*N*-[(1*R*)-3-methyl-1-[(1*S*,2*S*,6*R*,8*S*)-2,9,9-trimethyl-3,5-dioxa-4-boratricyclo[6.1.1.0^{2,6}]decan-4-yl]butyl]propanamide (38)

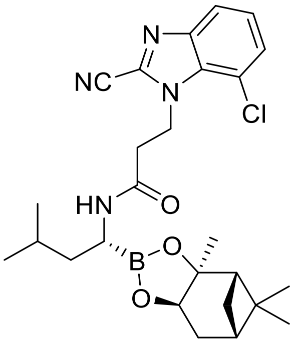

To a solution of 3-[(2-amino-6-chlorophenyl)amino]-*N*-[(1*R*)-3-methyl-1-[(1*S*,2*S*,6*R*,8*S*)-2,9,9-trimethyl-3,5-dioxa-4-boratricyclo[6.1.1.0^{2,6}]decan-4-yl]butyl]propanamide (**XLI**, 97 mg, 0.21 mmol, 1.0 equiv.) in pyridine (1500 µL), Appel's salt (44 mg, 0.21 mmol, 1.0 equiv.) was added. The reaction mixture was allowed to stir first at room temperature for 1 h and then at 70 °C for 6 h. After the reaction was complete, the product was purified by reversed-phase flash column chromatography using eluents A (0.1% HCOOH in MeCN) and B (0.1% HCOOH in H_2O) (gradient from 1:9 to 10:0). The product 3-(7-chloro-2-cyano-1*H*-1,3-benzodiazol-1-yl)-*N*-[(1*R*)-3-methyl-1-[(1*S*,2*S*,6*R*,8*S*)-2,9,9-trimethyl-3,5-dioxa-4-boratricyclo[6.1.1.0^{2,6}]decan-4-yl]butyl]propanamide (**38**) was obtained as a light brown solid (35 mg, 34% yield). Mp.: 68–72 °C; ^{1}H NMR (500 MHz, DMSO-*d*$_6$) 8.74 (d, *J* = 14.2 Hz, 1H), 7.79 (t, *J* = 6.3 Hz, 1H), 7.57 (t, *J* = 6.2 Hz, 1H), 7.40 (dd, *J* = 14.0, 5.9 Hz, 1H), 4.90 – 4.77 (m, 2H), 4.07 (t, *J* = 10.3 Hz, 1H), 2.92 (t, *J* = 6.7 Hz, 2H), 2.58 – 2.52 (m, 1H), 2.23 – 2.14 (m, 1H), 1.99 (dd, *J* = 9.4, 5.3 Hz, 1H), 1.82 (t, *J* = 5.6 Hz, 1H), 1.78 (d, *J* = 5.5 Hz, 1H), 1.58 (d, *J* = 13.9 Hz, 1H), 1.45 (dq, *J* = 13.2, 6.7 Hz, 1H), 1.26 – 1.19 (m, 9H), 0.78 (dd, *J* = 11.2, 5.2 Hz, 9H). ^{13}C NMR (126 MHz, DMSO-*d*$_6$) 170.68, 144.11, 129.67, 129.11, 127.26, 124.96, 120.24, 116.09, 110.90, 83.24, 75.81, 51.50, 43.04, 39.94, 37.64, 35.83, 33.98, 28.68, 27.07, 25.82, 24.75, 23.83, 23.04, 21.74; HRMS (ESI$^-$) *m*/*z* [M–H]$^-$, calcd. for $C_{26}H_{33}N_4O_3BCl$: 495.2334, found: 495.2314; Purity by HPLC: 98%.

2-(3-Amino-4-hydroxyphenyl)acetic acid hydrochloride (XLIII)

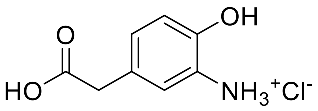

Methyl 2-(3-amino-4-hydroxyphenyl)acetate (**XLII**, 399 mg, 2.20 mmol) was suspended in 20% HCl (25 mL) and the mixture was allowed to reflux for 3 h. The volatiles were evaporated and the residue was triturated with Et_2O (30 mL). After filtration, the solid was washed with Et_2O (2 × 30 mL). The product 2-(3-amino-4-hydroxyphenyl)acetic acid hydrochloride (**XLIII**) was obtained as a brown solid (438 mg, 98% yield). Mp.: 186–189 °C; ^{1}H NMR (500 MHz, DMSO-d_6) 10.65 (s, 1H), 9.92 (s, 1H), 7.23 (d, *J* = 2.0 Hz, 1H), 7.08 (dd, *J* = 8.3, 1.9 Hz, 1H), 7.00 (d, *J* = 8.3 Hz, 1H), 3.48 (s, 2H); ^{13}C NMR (126 MHz, DMSO-d_6) 172.64, 149.41, 129.80, 125.96, 124.59, 119.07, 115.99, 39.58; HRMS (ESI$^+$) *m/z* [M+H]$^+$, calcd. for $C_8H_{10}NO_3$: 168.0661, found: 168.0659; Purity by HPLC: 100%.

2-(3-Amino-4-hydroxyphenyl)-*N*-[(1*R*)-3-methyl-1-[(1*S*,2*S*,6*R*,8*S*)-2,9,9-trimethyl-3,5-dioxa-4-boratricyclo[6.1.1.0^{2,6}]decan-4-yl]butyl]acetamide (XLIV)

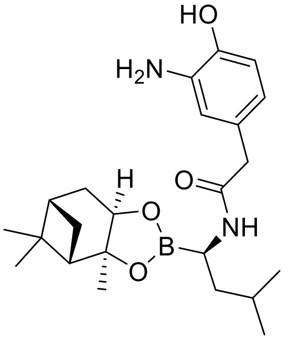

To a stirred solution of 2-(3-amino-4-hydroxyphenyl)acetic acid hydrochloride (**XLIII**, 244 mg, 1.2 mmol, 1.0 equiv.), HATU (546 mg, 1.44 mmol, 1.2 equiv.) and DIPEA (669 µL, 3.84 mmol, 3.2 equiv.) in DMF (4.0 mL), (*R*)-BoroLeu-(+)-pinanediol trifluoroacetate (548 mg, 1.44 mmol, 1.2 equiv.) was added and the mixture was allowed to stir at room temperature for 2 h. The mixture was purified by reversed-phase flash column chromatography using eluents A (0.1% HCOOH in MeCN) and B (0.1% HCOOH in H_2O) (gradient from 1:9 to 10:0). The product 2-(3-amino-4-hydroxyphenyl)-*N*-[(1*R*)-3-methyl-1-[(1*S*,2*S*,6*R*,8*S*)-2,9,9-trimethyl-3,5-dioxa-4-boratricyclo[6.1.1.0^{2,6}]decan-4-yl]butyl]acetamide (**XLIV**) was obtained as a white powder (225 mg, 51% yield). Mp.: 91–93 °C; ^{1}H NMR (500 MHz, DMSO-d_6) 9.36 (s, 1H), 6.56 (d, *J* = 7.9 Hz, 1H), 6.47 (d, *J* = 2.0 Hz, 1H), 6.28 (dd, *J* = 8.0, 2.0 Hz, 1H), 3.95 (dd, *J* = 8.4, 2.0 Hz, 1H), 3.39 – 3.30 (m, 2H), 2.39 (t, *J* = 7.5 Hz, 1H), 2.16 (dt, *J* = 11.0, 10.5 Hz, 1H), 2.00 – 1.91 (m, 1H), 1.81 – 1.65 (m, 3H), 1.59 (d, *J* = 13.8 Hz, 1H), 1.35 (t, *J* = 9.9 Hz, 1H), 1.26 – 1.18 (m, 9H), 0.85 (t, *J* = 6.1 Hz, 6H), 0.80 (s, 3H); ^{13}C NMR (126 MHz, DMSO-d_6) 175.20, 143.00, 136.48, 124.78, 116.69, 114.92, 114.19, 81.61, 75.10, 52.15, 40.82, 37.58, 36.89, 36.71, 29.34, 27.25, 25.98, 25.06, 23.99, 23.14, 22.07; HRMS (ESI$^+$) *m/z* [M+H]$^+$, calcd. for $C_{23}H_{36}N_2O_4B$: 415.2768, found: 415.2766; Purity by HPLC: 99%.

2-(2-Cyano-1,3-benzoxazol-5-yl)-*N*-[(1*R*)-3-methyl-1-[(1*S*,2*S*,6*R*,8*S*)-2,9,9-trimethyl-3,5-dioxa-4-boratricyclo[6.1.1.0^{2,6}]decan-4-yl]butyl]acetamide (39)

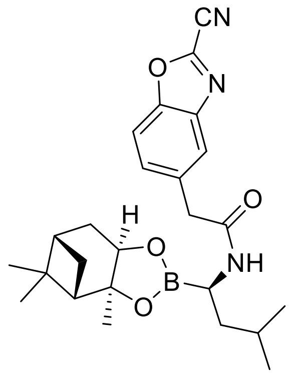

To a solution of 2-(3-amino-4-hydroxyphenyl)-*N*-[(1*R*)-3-methyl-1-[(1*S*,2*S*,6*R*,8*S*)-2,9,9-trimethyl-3,5-dioxa-4-boratricyclo[6.1.1.0^{2,6}]decan-4-yl]butyl]acetamide (**XLIV**, 166 mg, 0.40 mmol, 1.0 equiv.) in pyridine (3000 µL), Appel's salt (100 mg, 0.48 mmol, 1.2 equiv.) was added. The reaction mixture was allowed to stir first at room temperature for 1 h and at 90 °C for 1 h. the product was purified by reversed-phase flash column chromatography using eluents A (0.1% HCOOH in MeCN) and B (0.1% HCOOH in H_2O) (gradient from 1:9 to 10:0). The product 2-(2-cyano-1,3-benzoxazol-5-yl)-*N*-[(1*R*)-3-methyl-1-[(1*S*,2*S*,6*R*,8*S*)-2,9,9-trimethyl-3,5-dioxa-4-boratricyclo[6.1.1.0^{2,6}]decan-4 yl]butyl]acetamide (**39**) was obtained as a brown solid (42 mg, 23% yield). Mp.: 60–65 °C; ^{1}H NMR (500 MHz, DMSO-*d*$_6$) 9.20 (d, *J* = 12.9 Hz, 1H), 7.91 – 7.85 (m, 2H), 7.58 (dd, *J* = 8.6, 1.3 Hz, 1H), 4.02 (d, *J* = 6.9 Hz, 1H), 3.76 (s, 2H), 2.57 – 2.52 (m, 1H), 2.21 – 2.14 (m, 1H), 2.00 – 1.94 (m, 1H), 1.81 (t, *J* = 5.6 Hz, 1H), 1.77 (d, *J* = 5.4 Hz, 1H), 1.69 (dq, *J* = 13.5, 6.8 Hz, 1H), 1.59 (d, *J* = 13.9 Hz, 1H), 1.30 (d, *J* = 7.6 Hz, 2H), 1.20 (d, *J* = 1.4 Hz, 6H), 0.85 (dd, *J* = 6.4, 4.7 Hz, 6H), 0.80 (d, *J* = 2.5 Hz, 1H), 0.79 (s, 3H). ^{13}C NMR (126 MHz, DMSO-*d*$_6$) 172.93, 148.95, 138.95, 137.50, 133.89, 130.35, 121.39, 111.65, 109.70, 82.61, 75.58, 51.76, 40.42, 37.91, 37.64, 36.21, 28.99, 27.14, 25.89, 25.00, 23.89, 13.09, 22.00; HRMS (ESI$^+$) *m*/*z* $[M+H]^+$, calcd. for $C_{25}H_{33}N_3O_4B$: 450.2564, found: 450.2566; Purity by HPLC: 99%.

[(1*R*)-1-[2-(2-Cyano-1,3-benzoxazol-5-yl)acetamido]-3-methylbutyl]boronic acid (40)

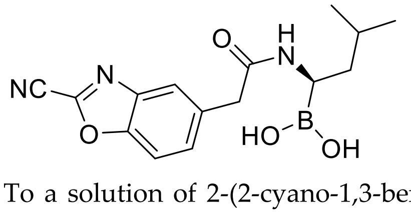

To a solution of 2-(2-cyano-1,3-benzoxazol-5-yl)-*N*-[(1*R*)-3-methyl-1-[(1*S*,2*S*,6*R*,8*S*)-2,9,9-trimethyl-3,5-dioxa-4-boratricyclo[6.1.1.0^{2,6}]decan-4-yl]butyl]acetamide (**39**, 14 mg, 0.031 mmol, 1.0 equiv.) and 2-methyl-1-propylboronic acid (19 mg, 0.19 mmol, 6.0 equiv.) in MeOH (1.2 mL), 1 M HCl (100 µL) and *n*-hexane (1.2 mL) were added, and the biphasic reaction mixture was allowed to stir vigorously at room temperature for 16 h. The *n*-hexane layer was discarded, the MeOH washed again with *n*-hexane (3 × 1 mL), and finally concentrated. The residue was re-dissolved in diisopropyl ether (2.0 mL), sonicated for 15 min, followed by discarding the ether solution. The residue was re-dissolved in MeCN (2.0 mL), sonicated for 15 min, decanted, and the MeCN solution was concentrated. The product [(1*R*)-1-[2-(2-cyano-1,3-benzoxazol-5-yl)acetamido]-3-methylbutyl]boronic acid (**40**) was obtained as a yellow solid (8 mg, 80% yield). Mp.: 125–127 °C; ^{1}H NMR (300 MHz, DMSO-*d*$_6$) 8.13 (s, 1H), 7.85 (t, *J* = 7.8 Hz, 2H), 7.57 (d, *J* = 8.6 Hz, 1H), 3.78 (s, 2H), 1.53 (dd, *J* = 13.2, 6.5 Hz, 1H), 1.19 (dd, *J* = 13.8, 7.1 Hz, 3H), 0.89 – 0.73 (m, 7H). ^{13}C NMR (75 MHz, DMSO-*d*$_6$) 163.00, 148.96, 138.94, 137.42,

133.69, 130.53, 121.62, 111.62, 109.68, 37.71, 25.11, 23.21, 23.01, 22.35; HRMS (ESI$^-$) *m/z* [M–H]$^-$, calcd. for $C_{15}H_{17}N_3O_4B$: 314.1312, found: 314.1310; Purity by ^{1}H NMR: 97%.

3. LC-MS chromatograms, ^{1}H NMR and ^{13}C NMR spectra of the synthesized compounds

5-Methoxy-1,3-benzoxazole-2-thiol (VIII)

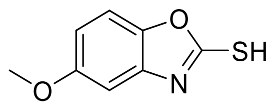

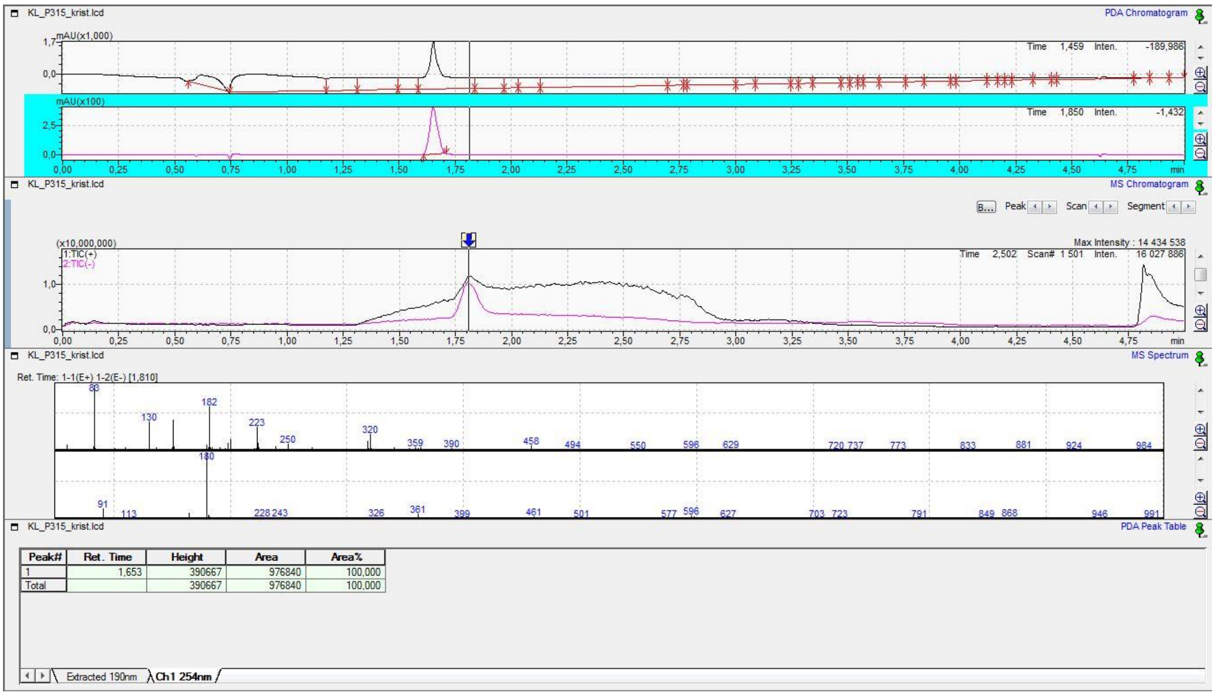
KL_P315_krist.lcd
PDA Chromatogram
mAU(x1,000)
Time 1,459 Inten. -189,986
mAU(x100)
Time 1,850 Inten. -1,432
MS Chromatogram
(x10,000,000)
1:TIC(+)
2:TIC(-)
Max Intensity : 14 434 538
Time 2,502 Scan# 1 501 Inten. 16 027 886
MS Spectrum
Ret. Time: 1-1(E+) 1-2(E-) [1,810]
PDA Peak Table
Peak# Ret. Time Height Area Area%
1 1,653 390667 976840 100,000
Total 390667 976840 100,000
Extracted 190nm
Ch1 254nm

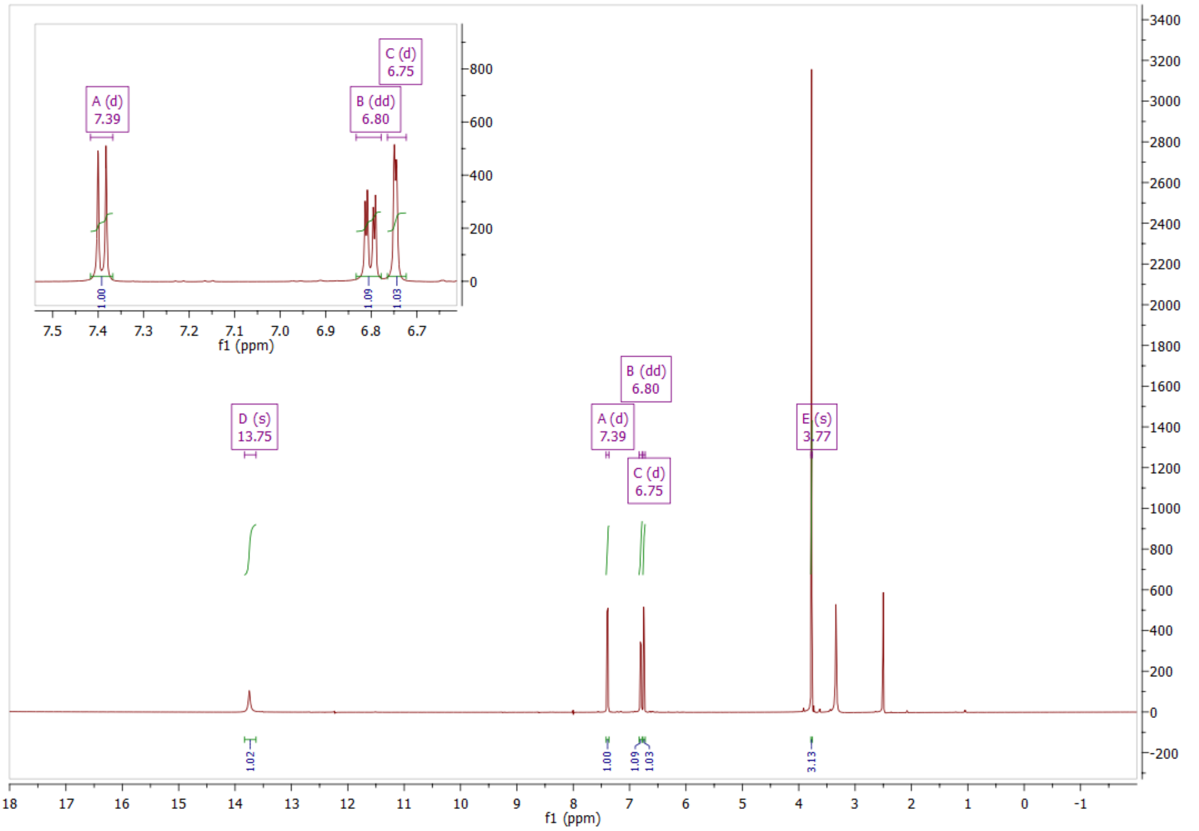
A (d) 7.39
B (dd) 6.80
C (d) 6.75
D (s) 13.75
E (s) 3.77
f1 (ppm)

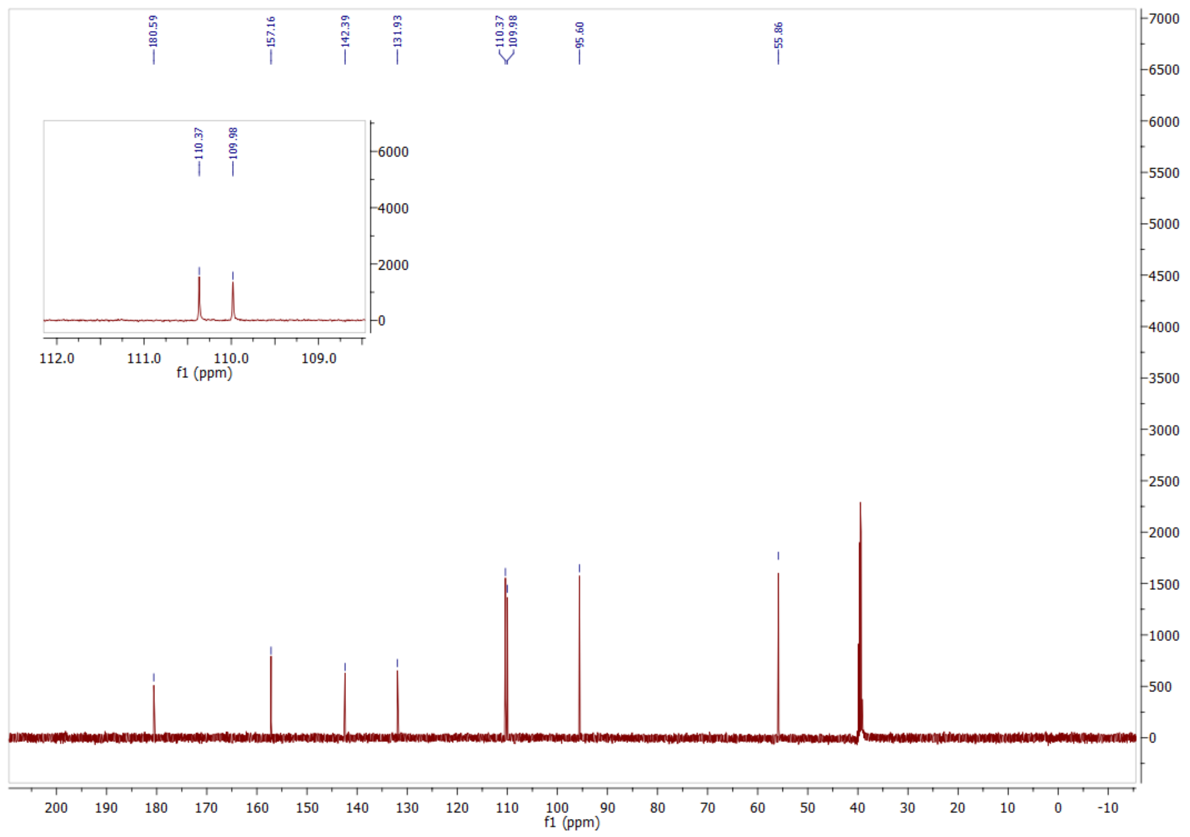

5-Nitro-1,3-benzoxazole-2-thiol (IX)

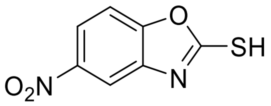

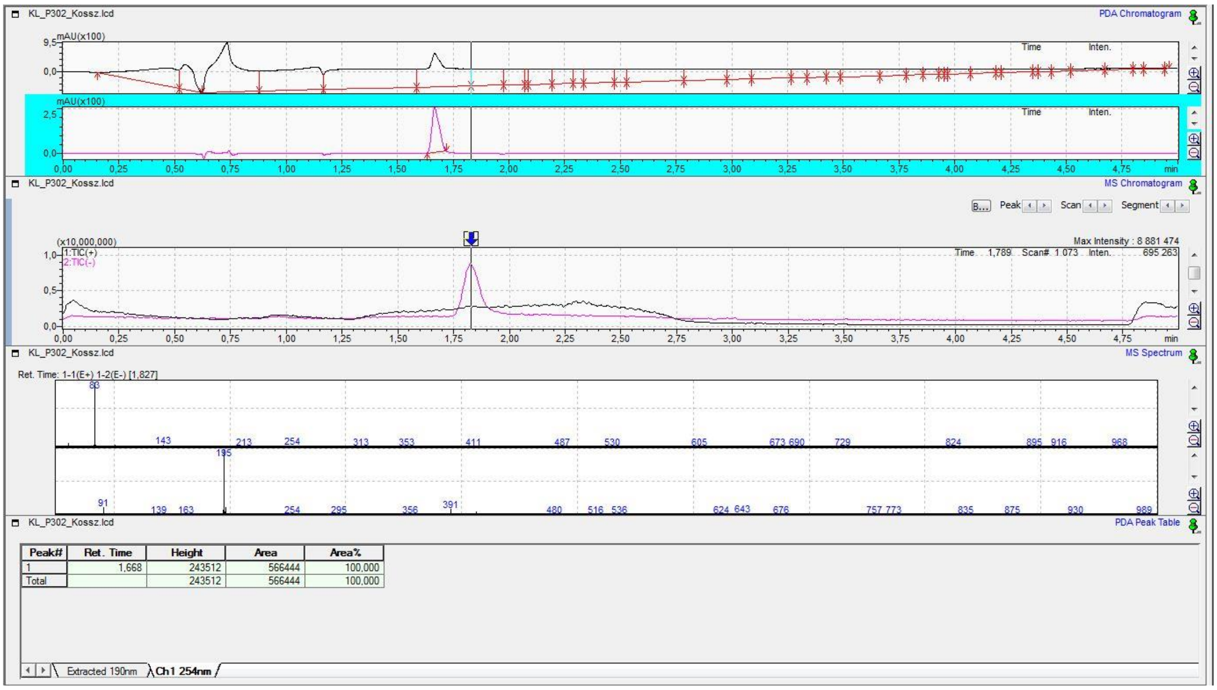

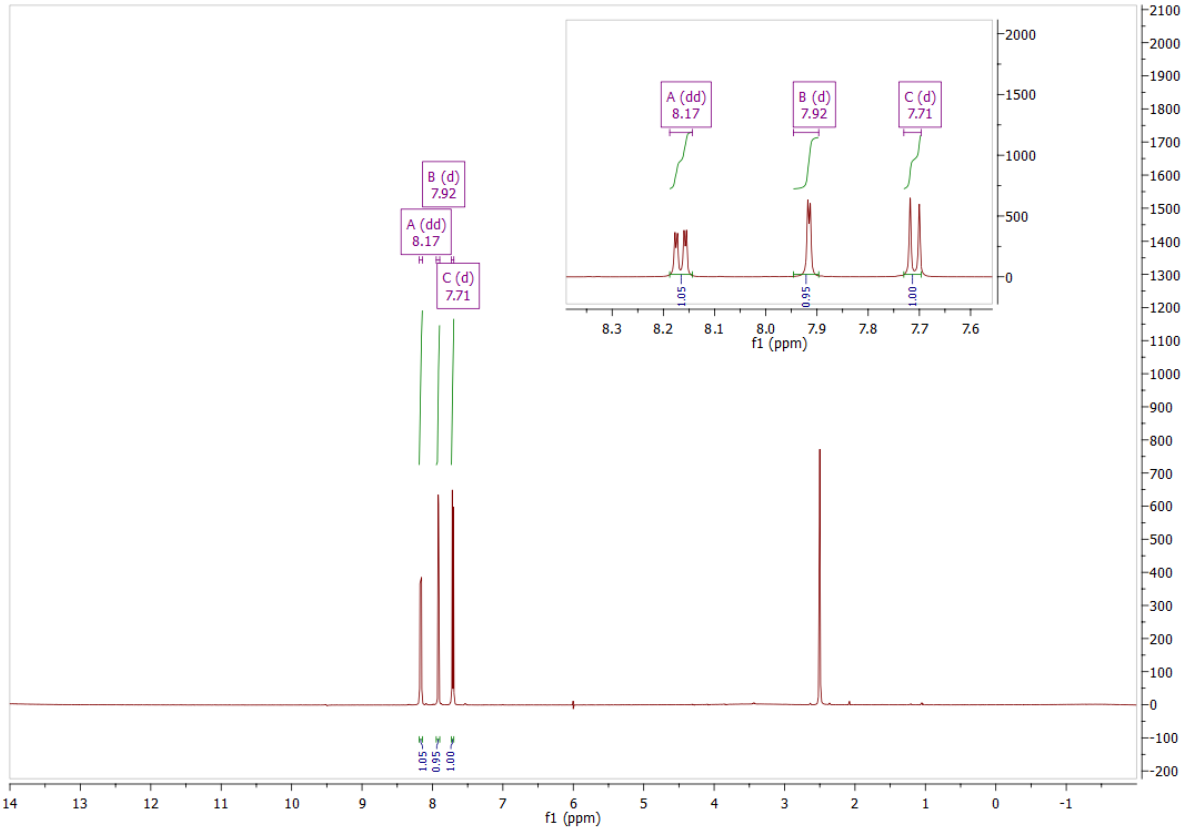
A (dd)
8.17
B (d)
7.92
C (d)
7.71
1.05
0.95
1.00
f1 (ppm)

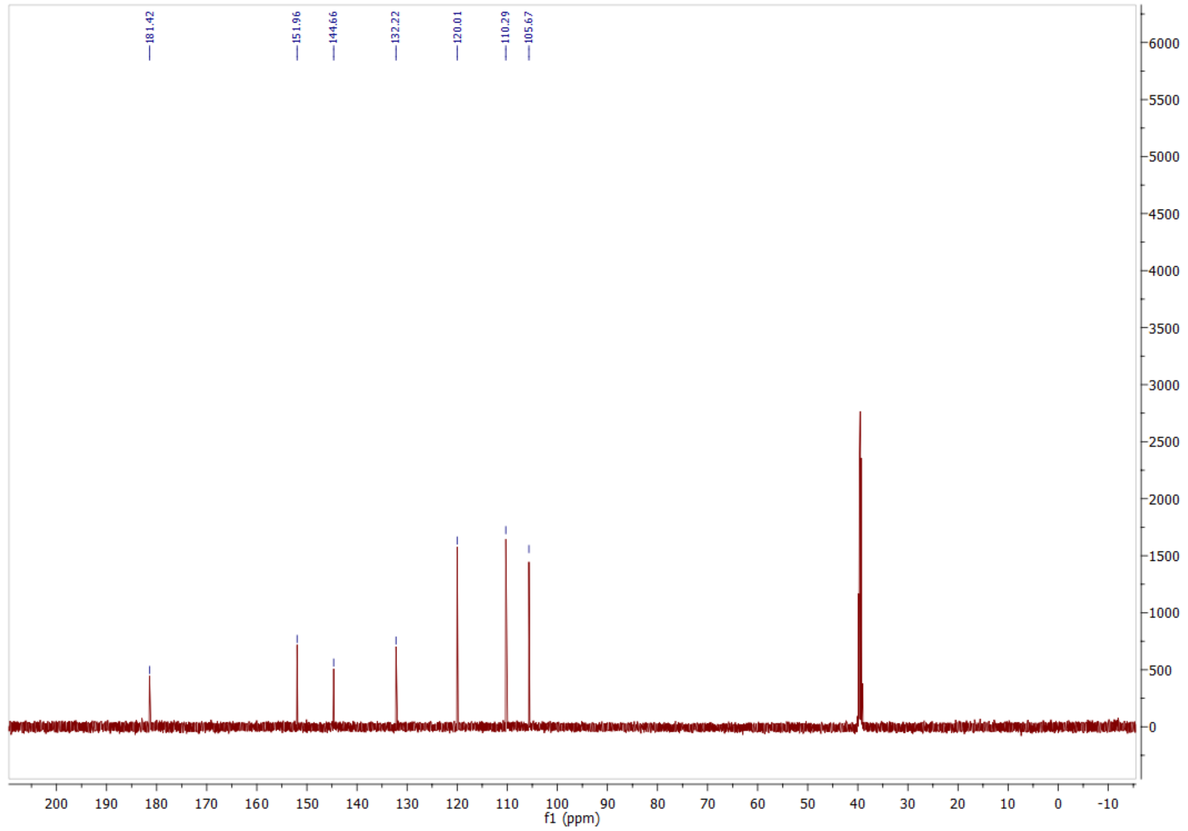
181.42
151.96
144.66
132.22
120.01
110.29
105.67
f1 (ppm)

6-Methyl-1,3-benzoxazole-2-carbonitrile (5)

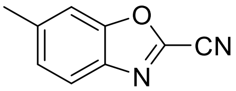

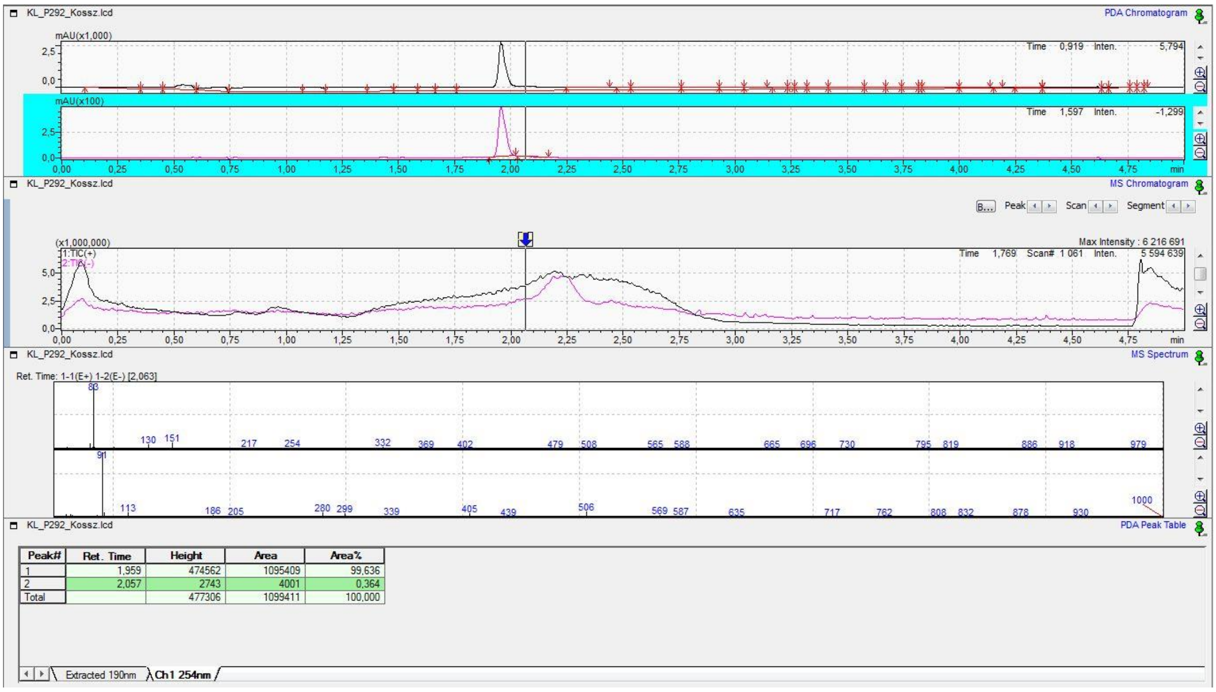

Peak#	Ret. Time	Height	Area	Area%
1	1,959	474562	1095409	99,636
2	2,057	2743	4001	0,364
Total		477306	1099411	100,000

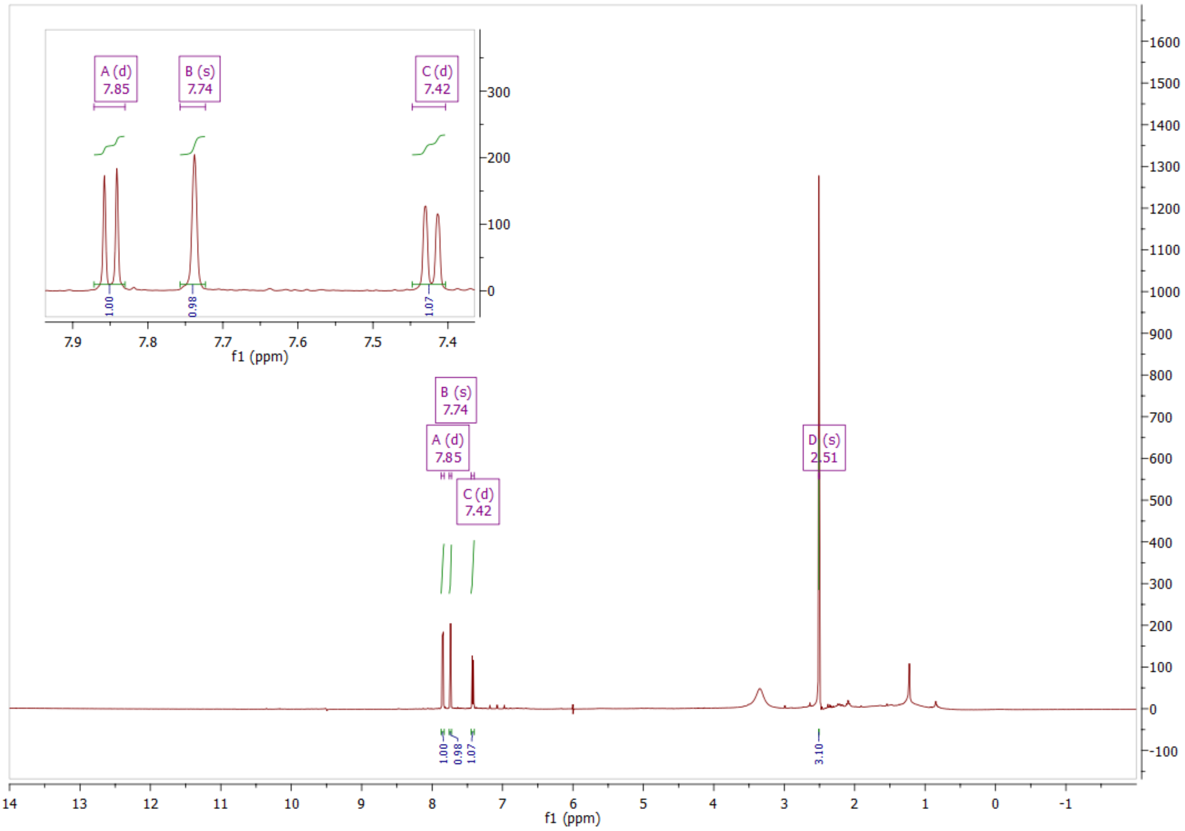

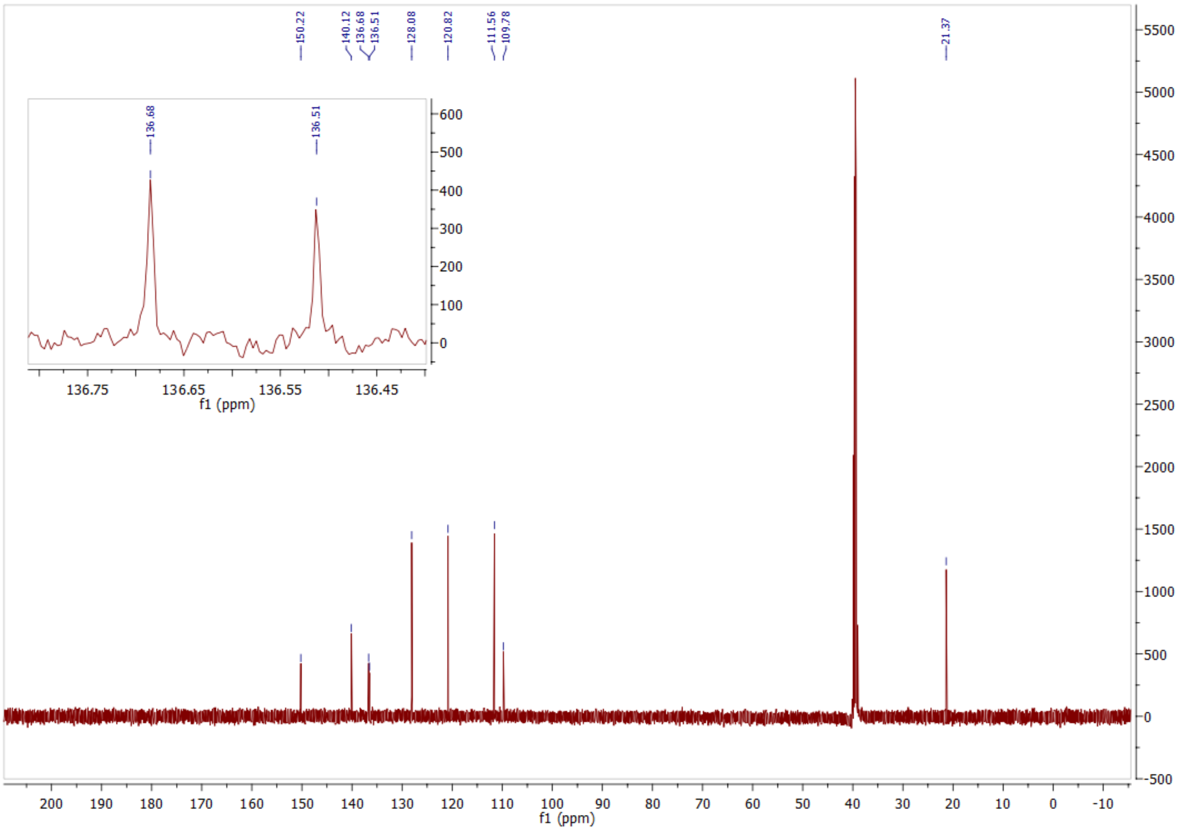

4-Methoxy-1,3-benzoxazole-2-carbonitrile (6)

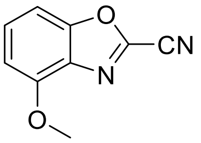

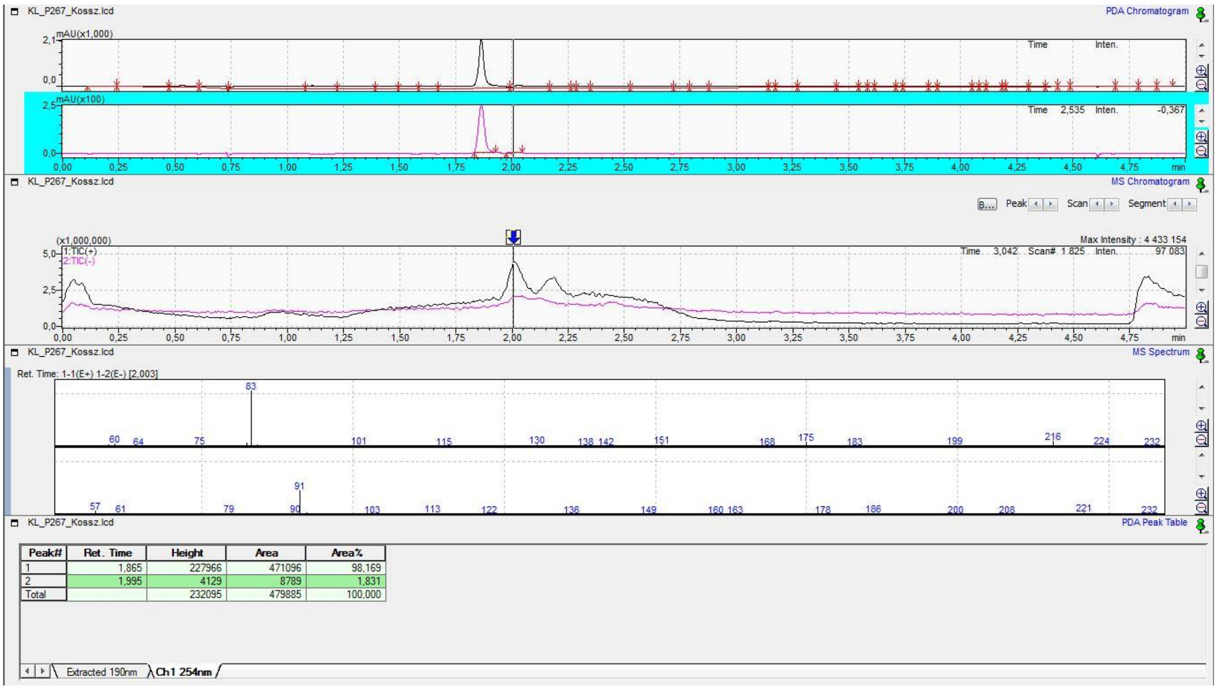

Peak#	Ret. Time	Height	Area	Area%
1	1,865	227966	471096	98,169
2	1,995	4129	8789	1,831
Total		232095	479885	100,000

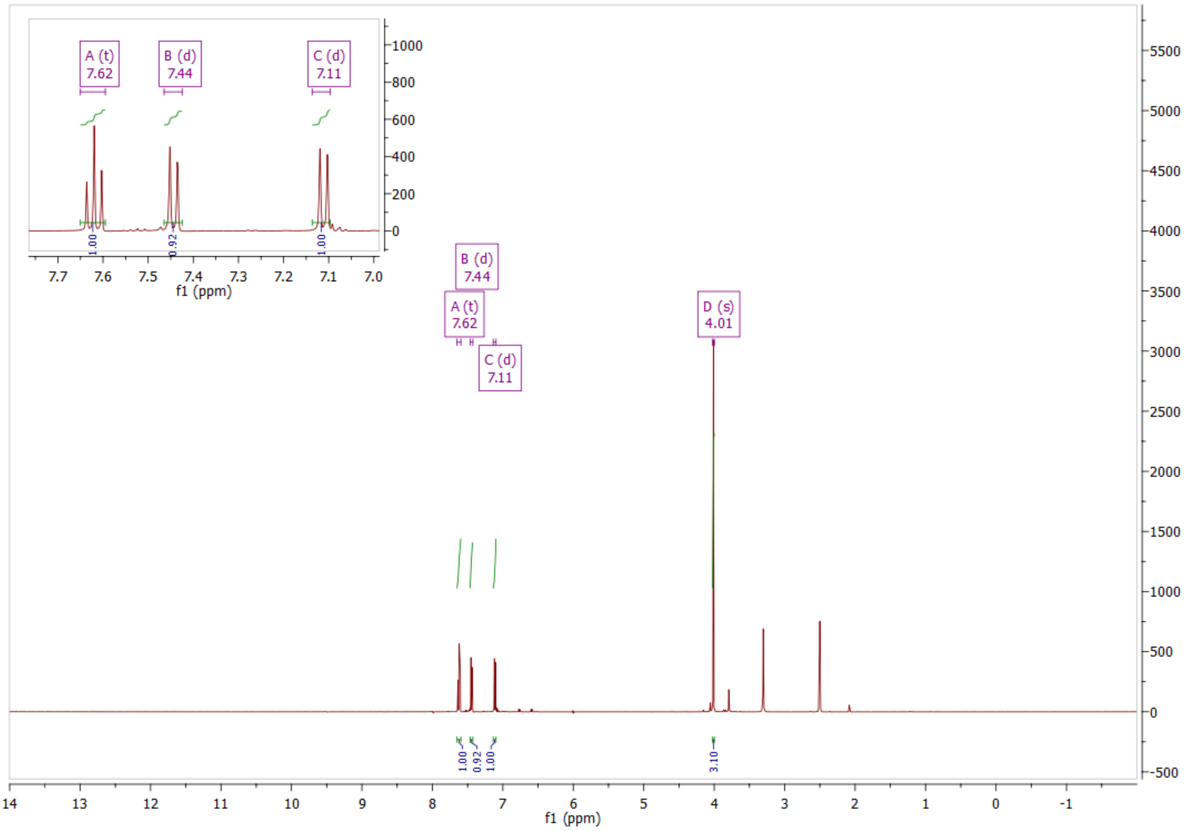
A (t)
7.62
B (d)
7.44
C (d)
7.11
D (s)
4.01
1.00
0.92
1.00
3.10
f1 (ppm)

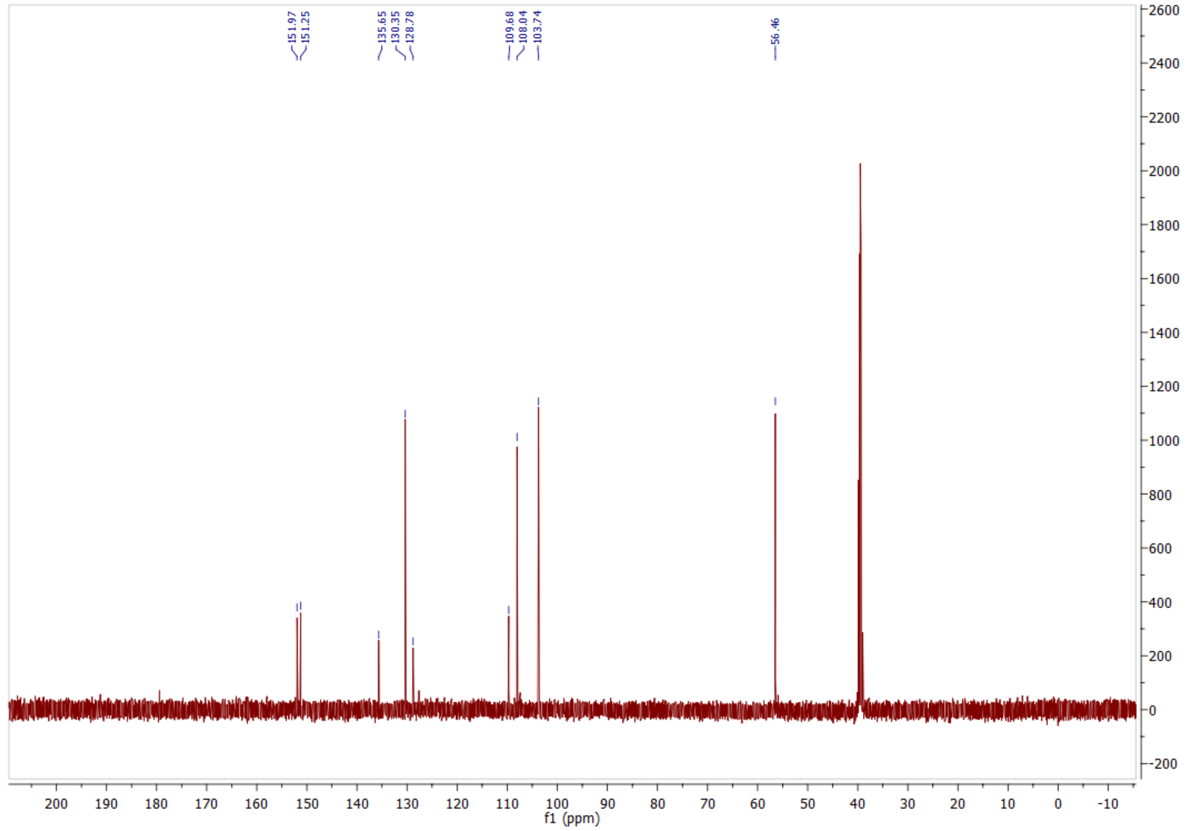
151.97
151.25
135.65
130.35
128.78
109.68
108.04
103.74
56.46
f1 (ppm)

5-Methoxy-1,3-benzoxazole-2-carbonitrile (7)

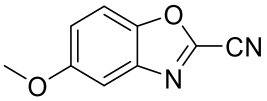

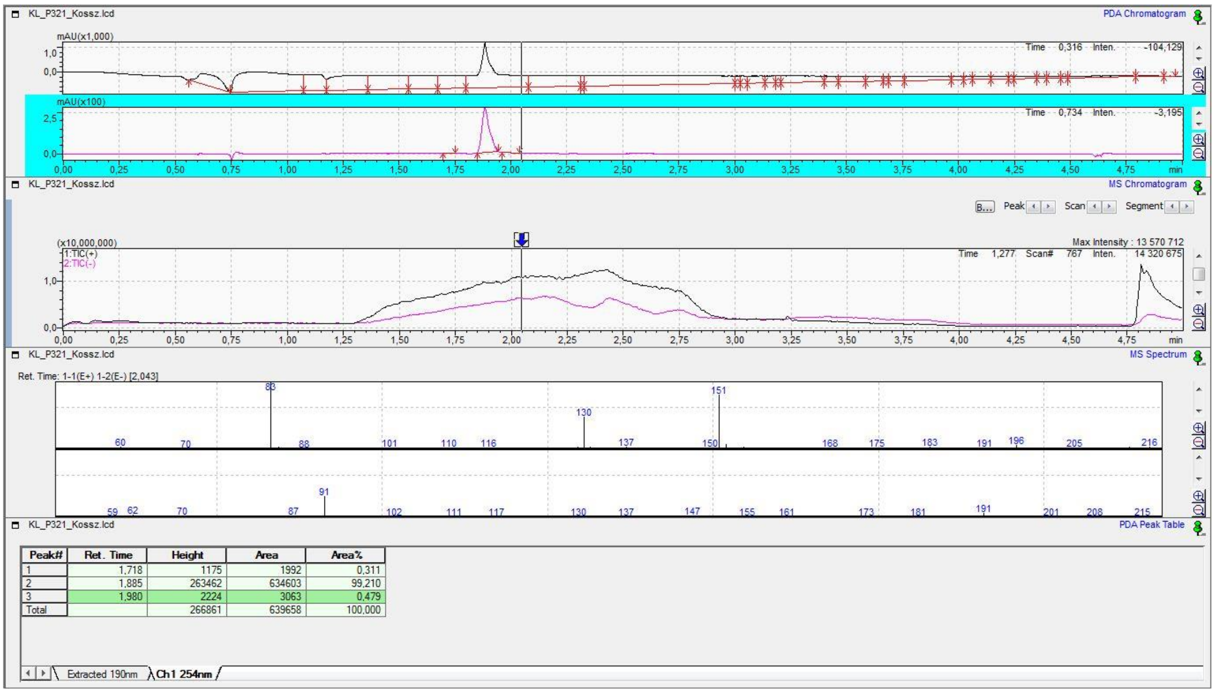

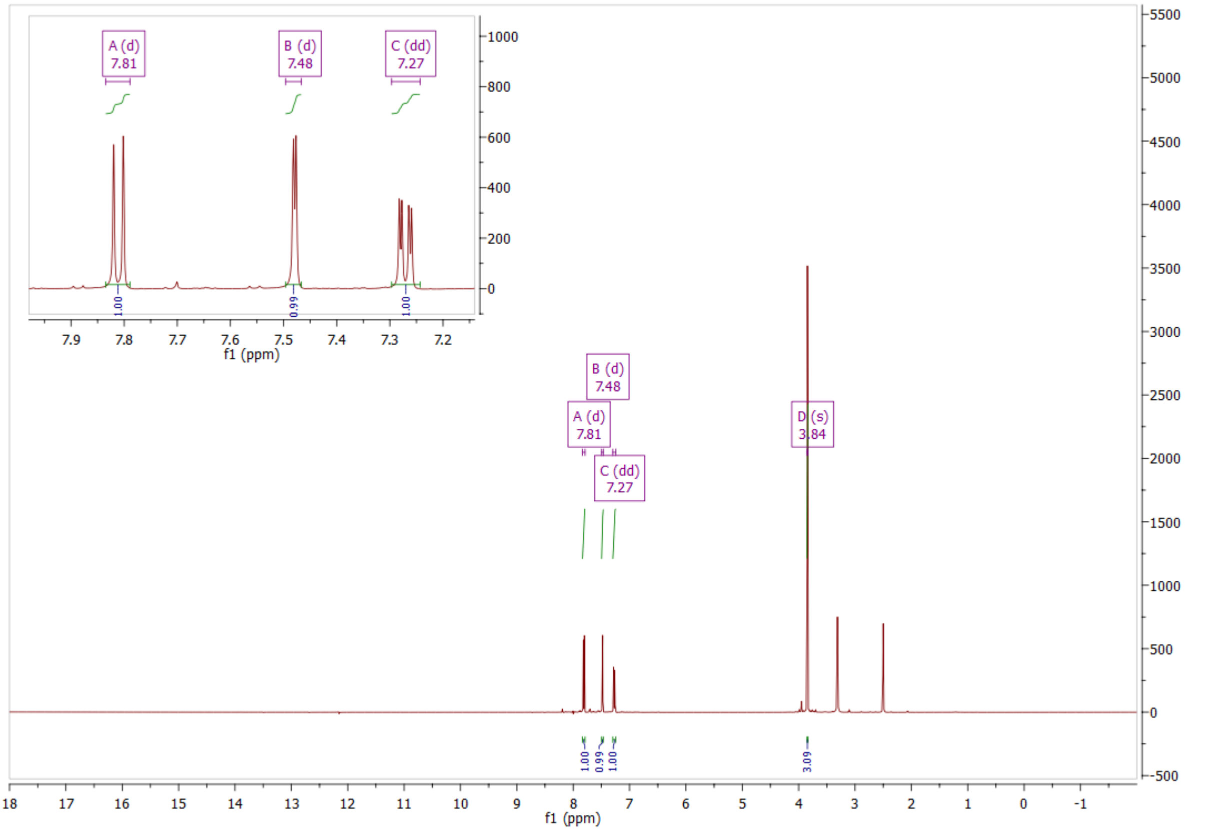

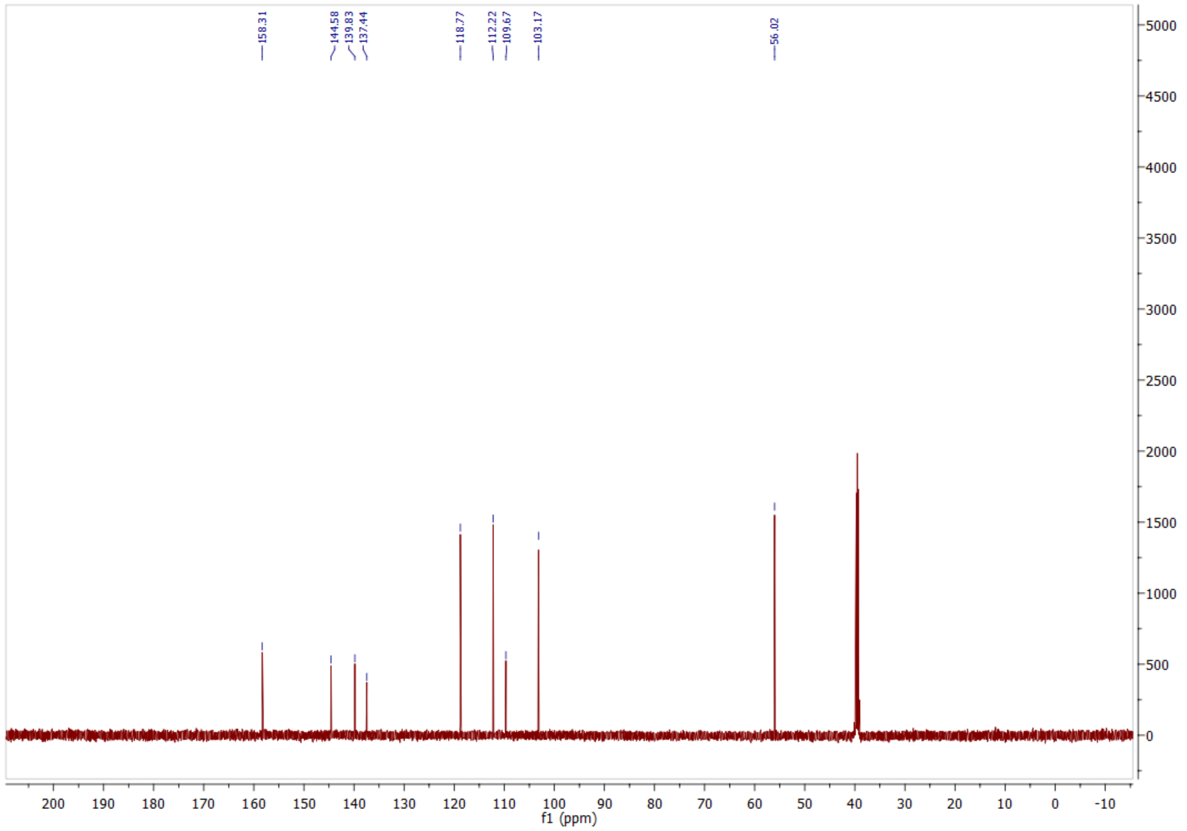

6-Methoxy-1,3-benzoxazole-2-carbonitrile (8)

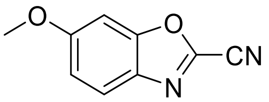

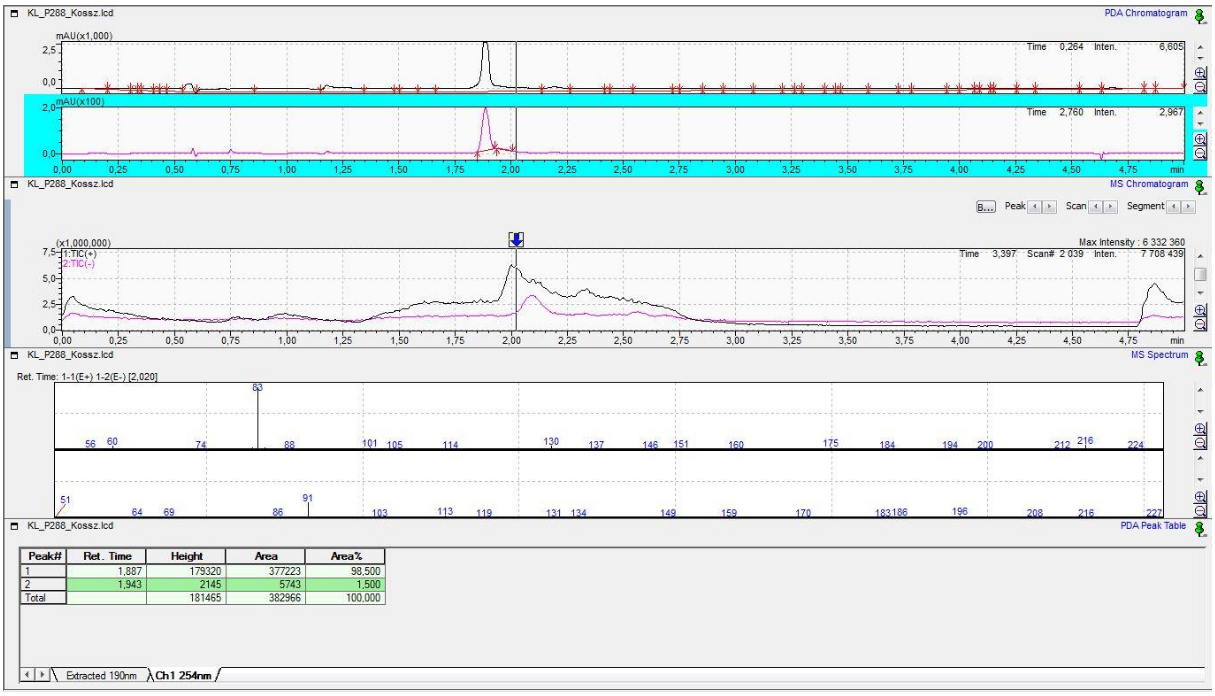

Peak#	Ret. Time	Height	Area	Area%
1	1,887	179320	377223	98,500
2	1,943	2145	5743	1,500
Total		181465	382966	100,000

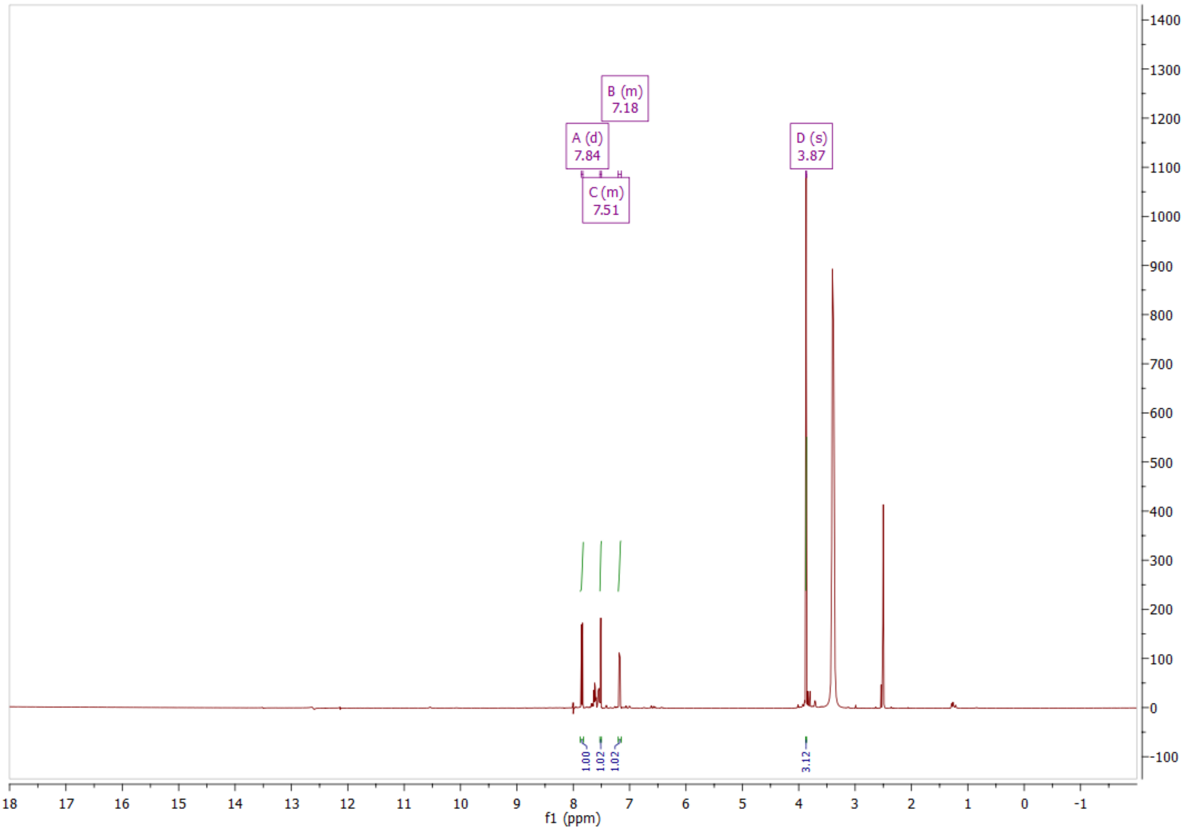
B (m)
7.18
A (d)
7.84
C (m)
7.51
D (s)
3.87
1.00
1.02
1.02
3.12
f1 (ppm)

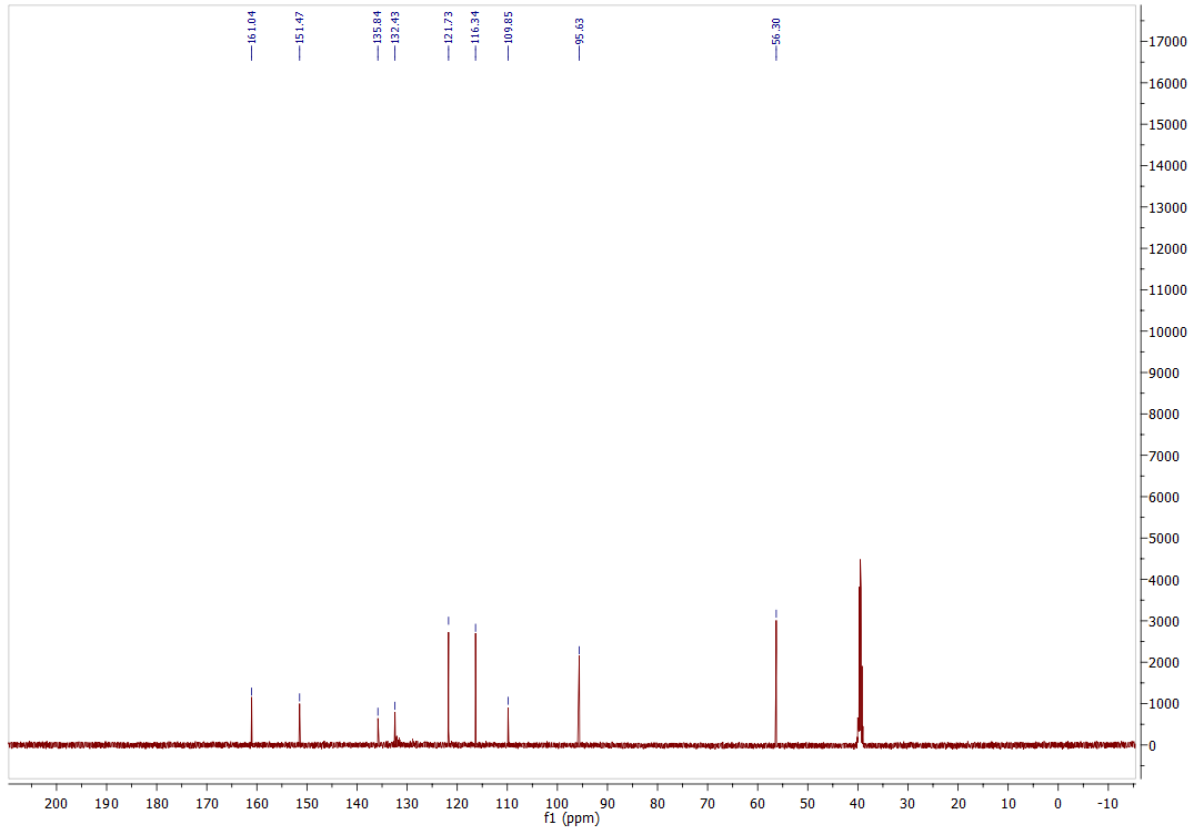
161.04
151.47
135.84
132.43
121.73
116.34
109.85
95.63
56.30
f1 (ppm)

7-Methoxy-1,3-benzoxazole-2-carbonitrile (9)

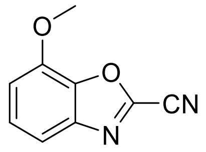

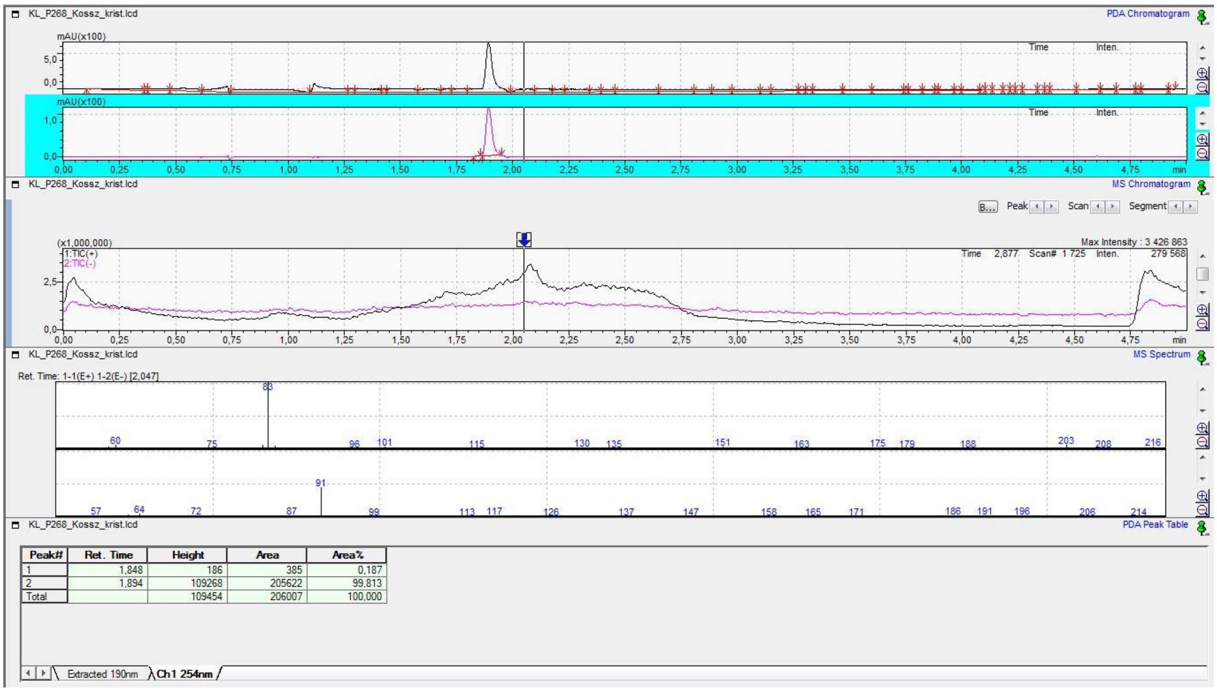

Peak#	Ret. Time	Height	Area	Area%
1	1,848	186	385	0,187
2	1,894	109268	205622	99,813
Total		109454	206007	100,000

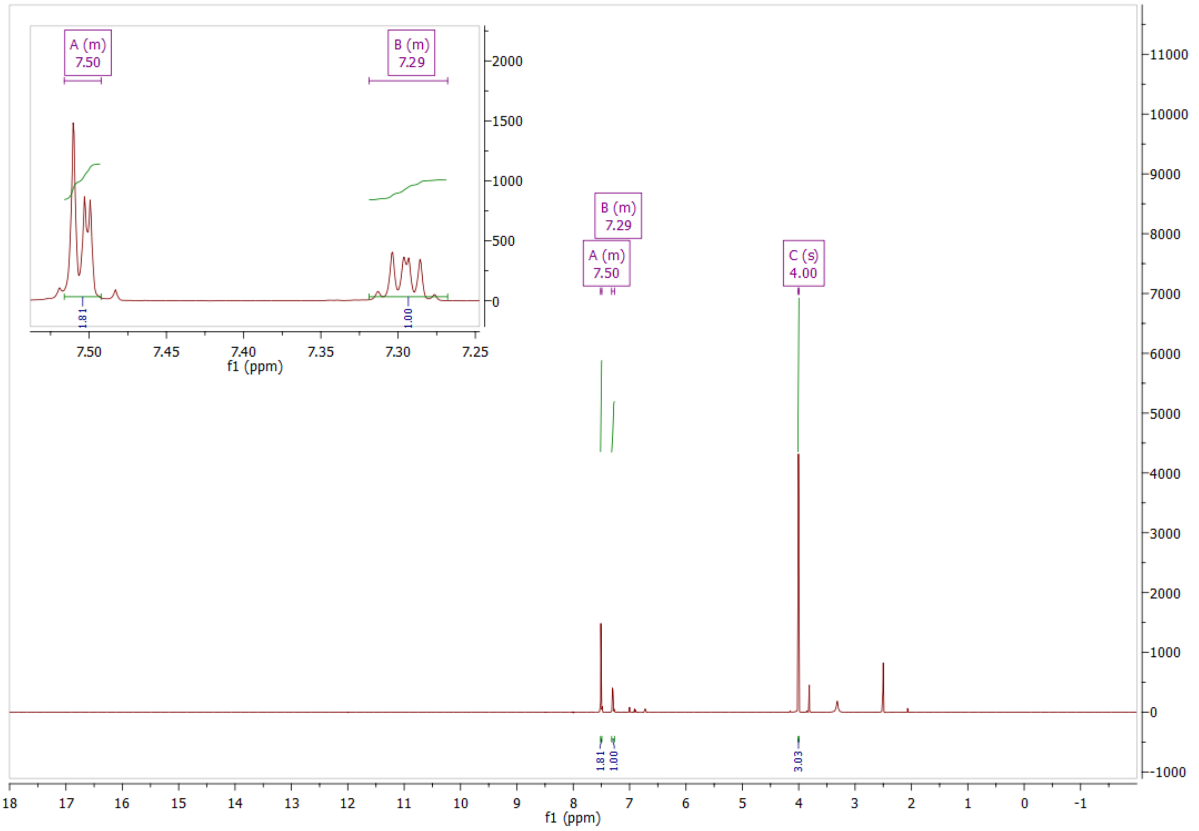

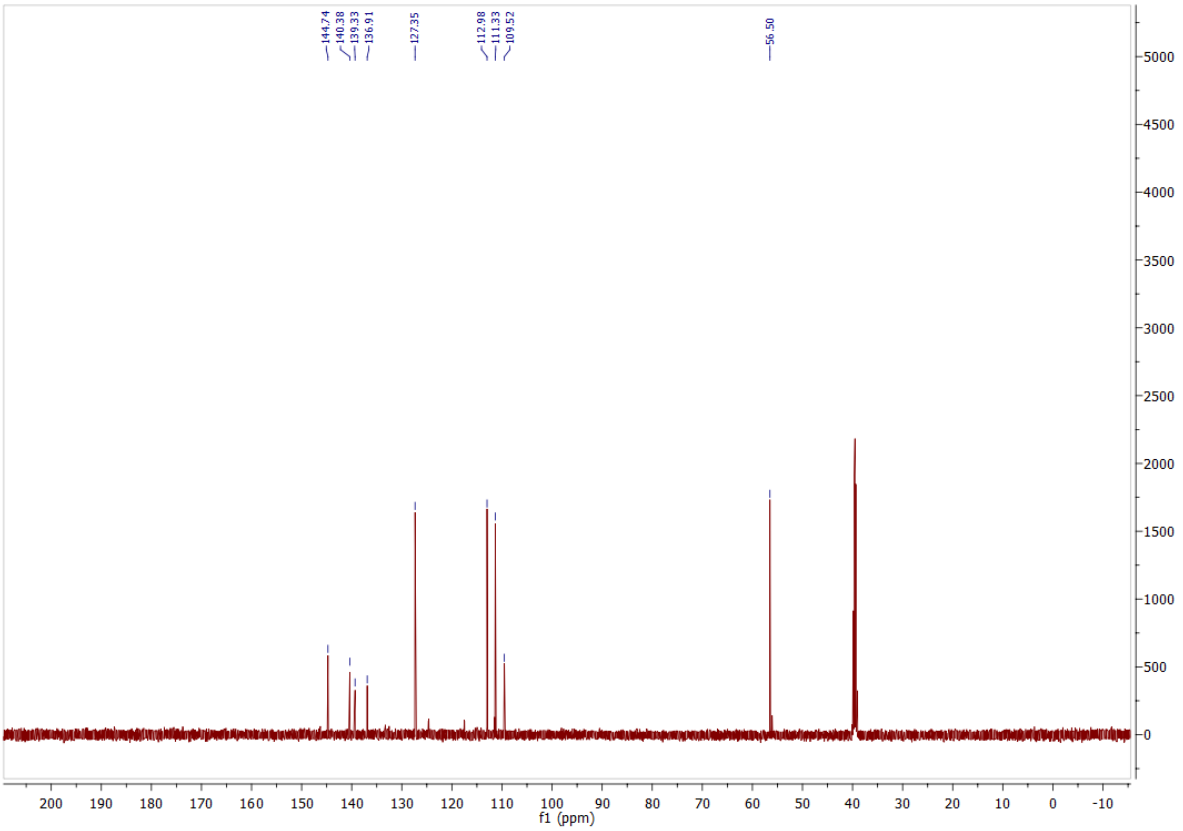

4-Hydroxy-1,3-benzoxazole-2-carbonitrile (10)

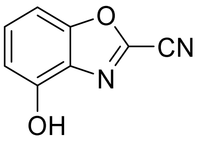

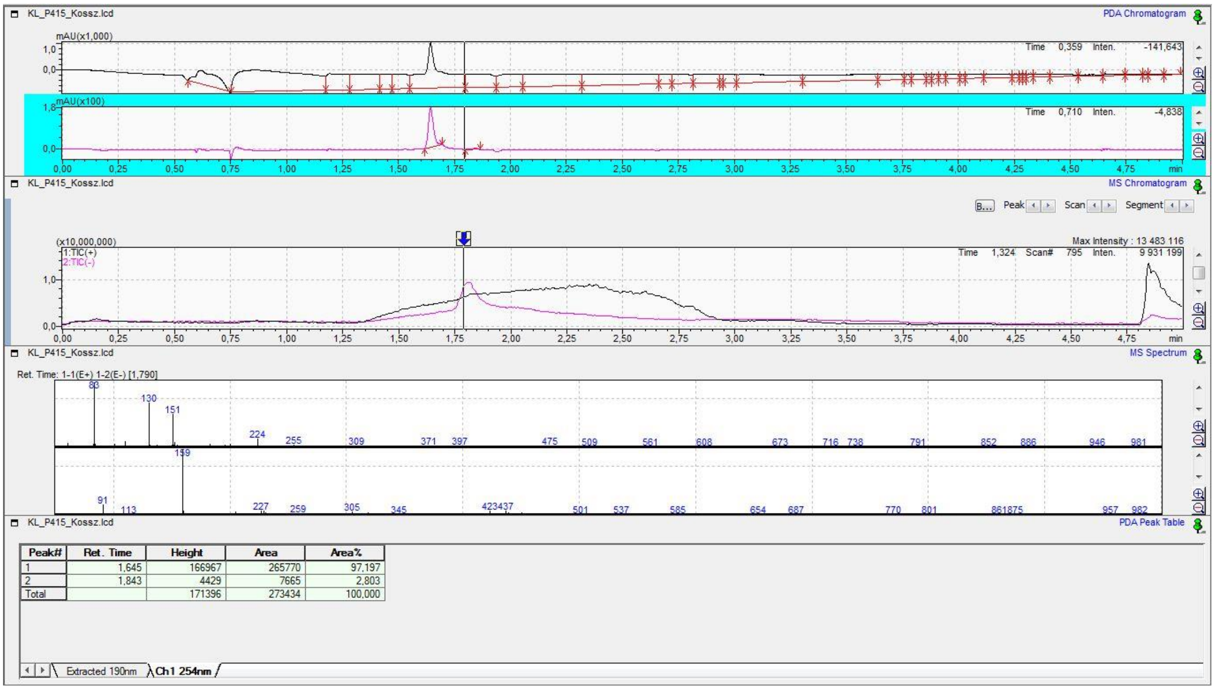

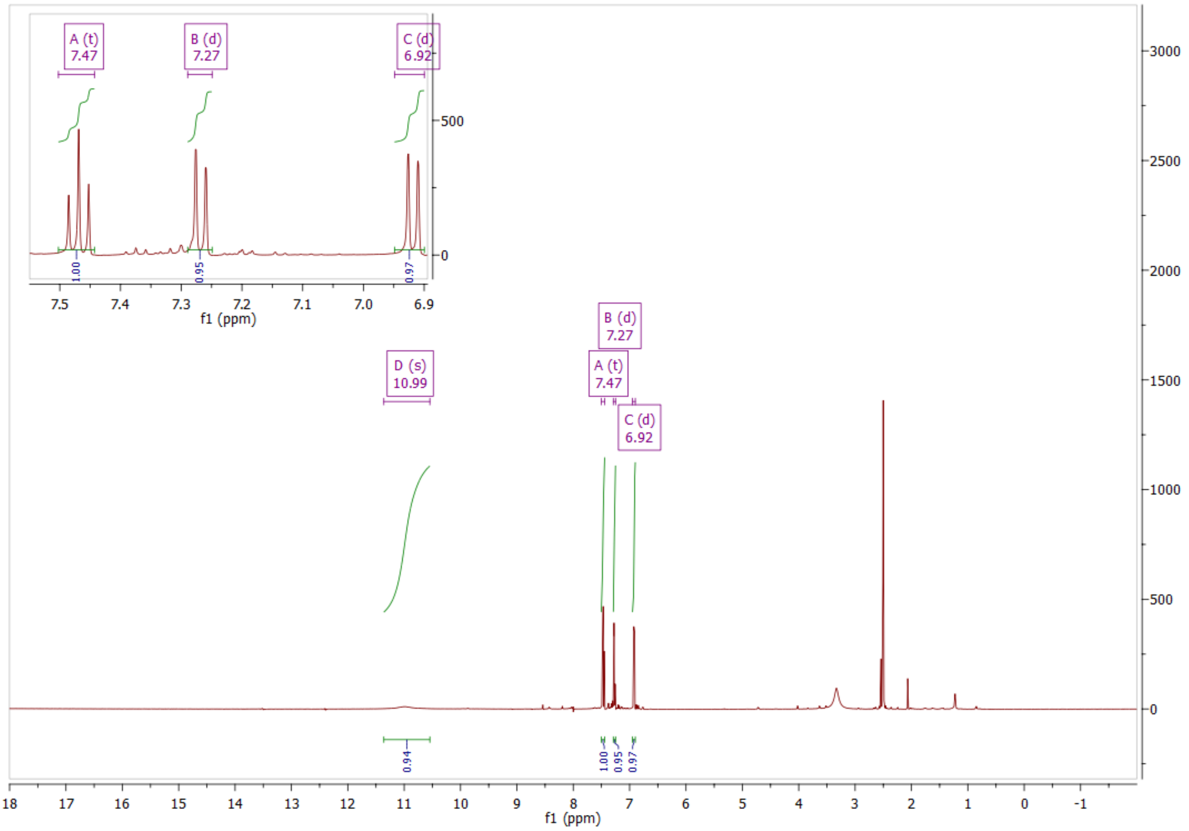

A (t) 7.47
B (d) 7.27
C (d) 6.92
D (s) 10.99
1.00
0.95
0.97
0.94
f1 (ppm)

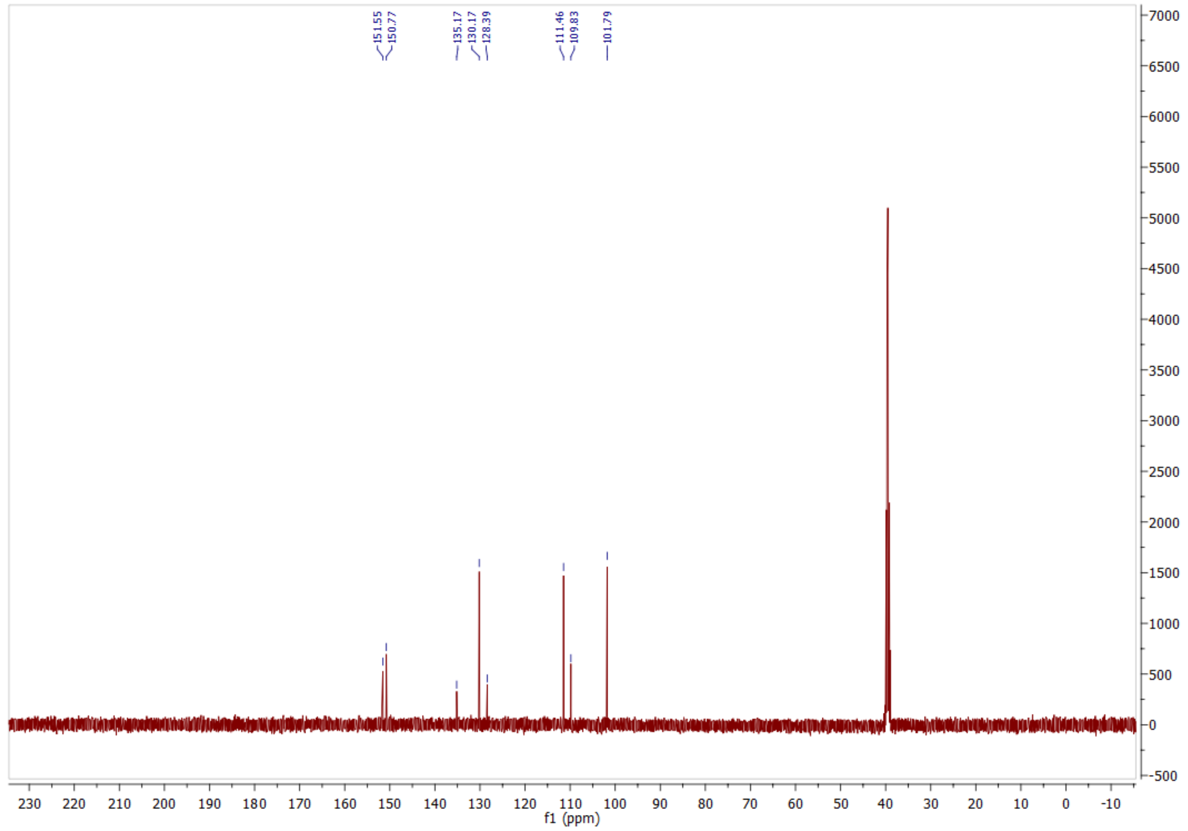

151.55
150.77
135.17
130.17
128.39
111.46
109.83
101.79
f1 (ppm)

5-Hydroxy-1,3-benzoxazole-2-carbonitrile (11)

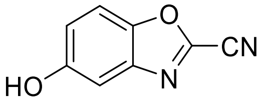

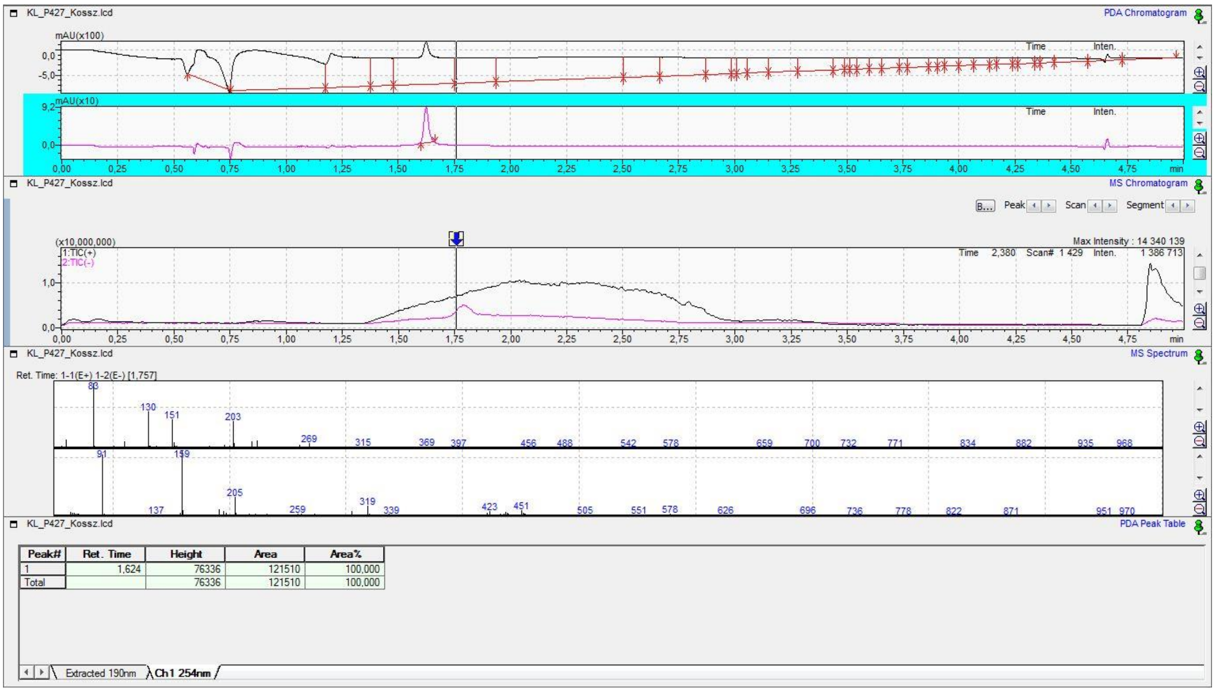

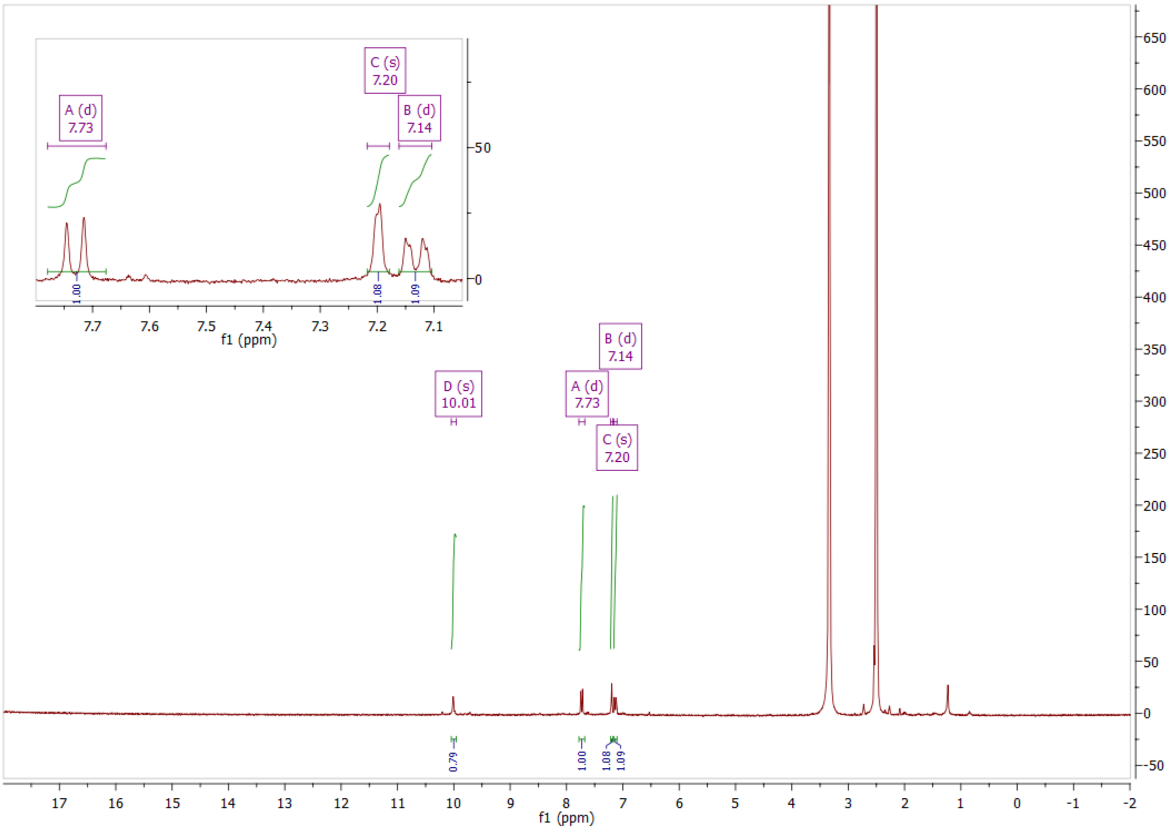

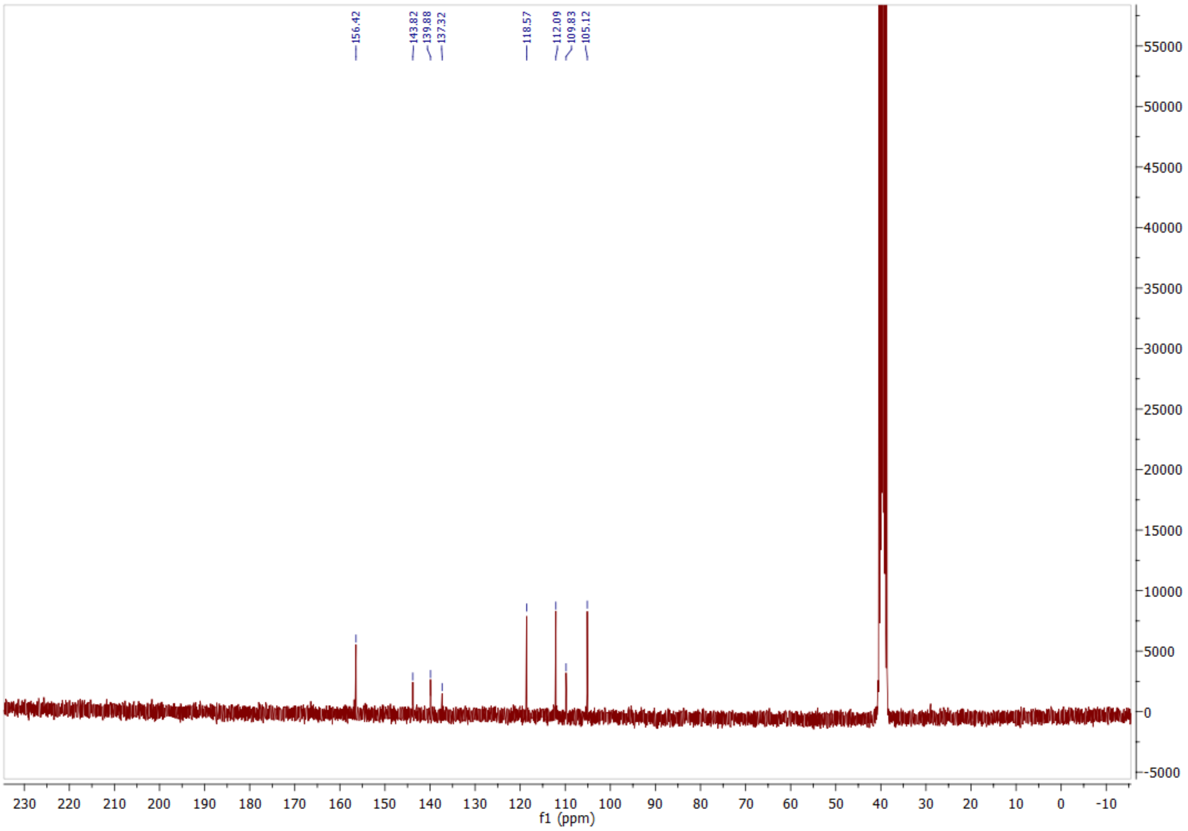

6-Hydroxy-1,3-benzoxazole-2-carbonitrile (12)

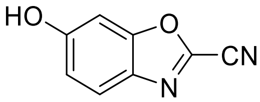

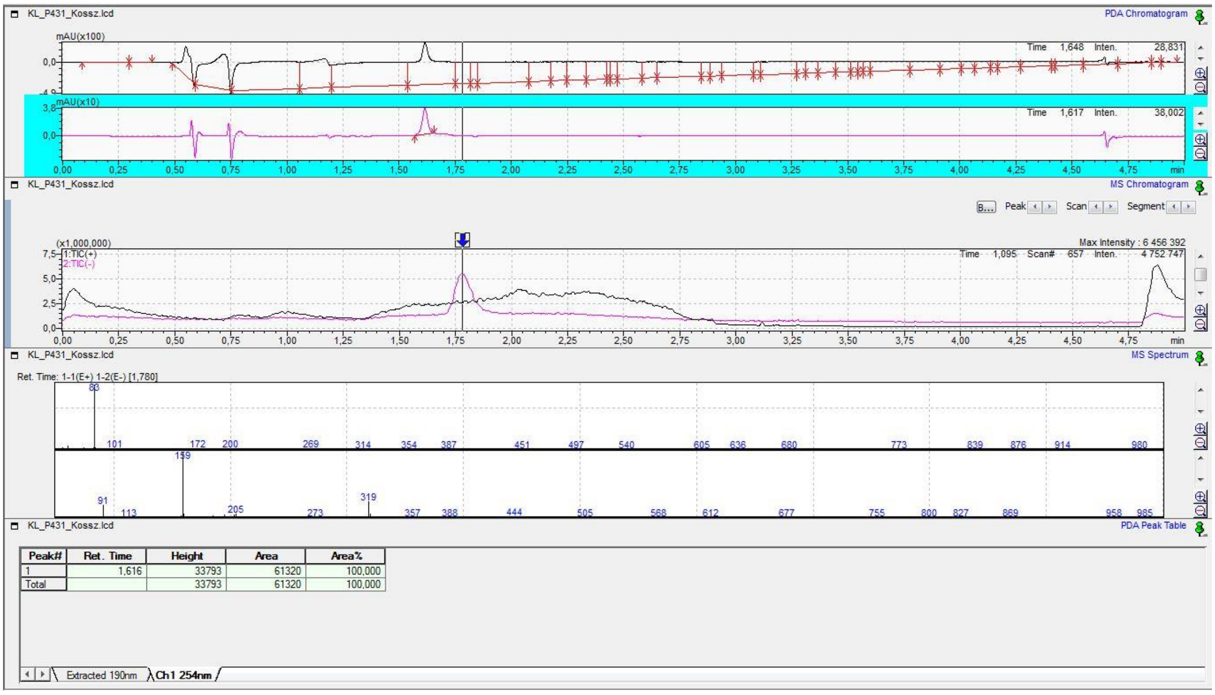

Peak#	Ret. Time	Height	Area	Area%
1	1,616	33793	61320	100,000
Total		33793	61320	100,000

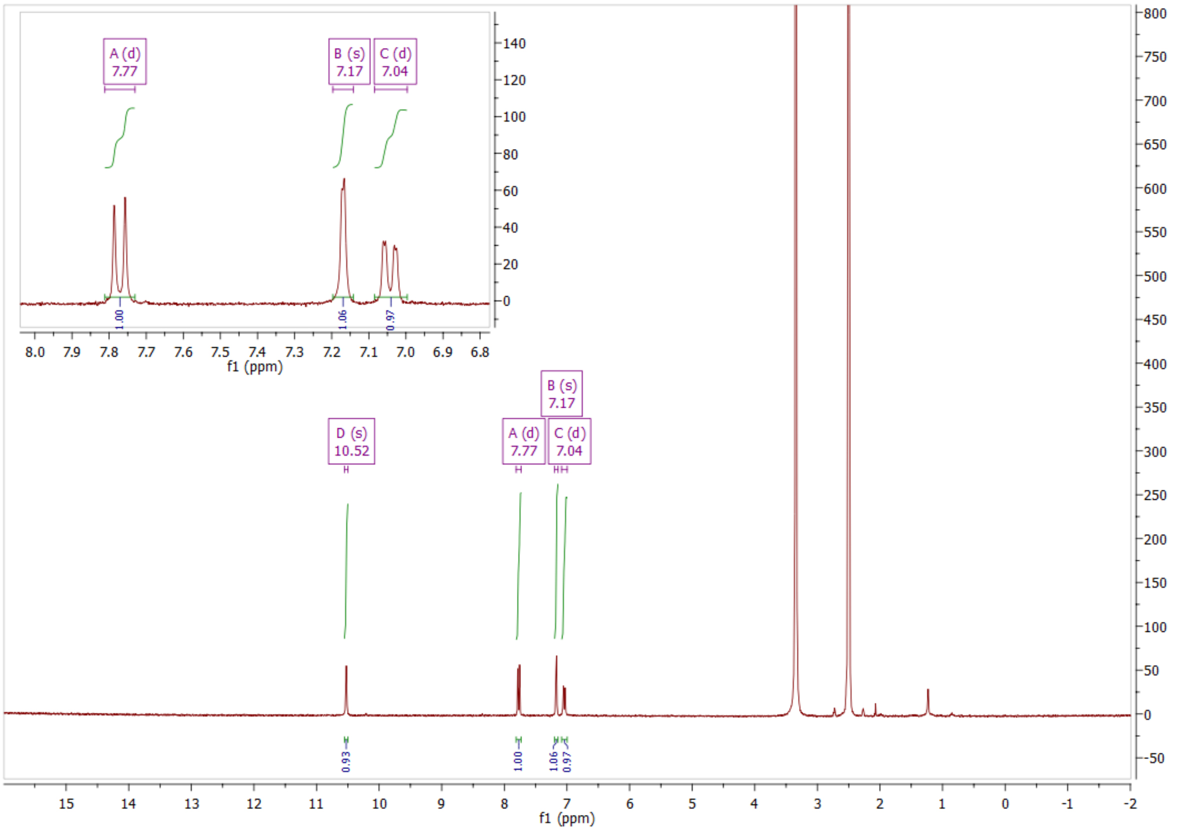
A (d)
7.77
B (s)
7.17
C (d)
7.04
D (s)
10.52
1.00
1.06
0.97
0.93
f1 (ppm)

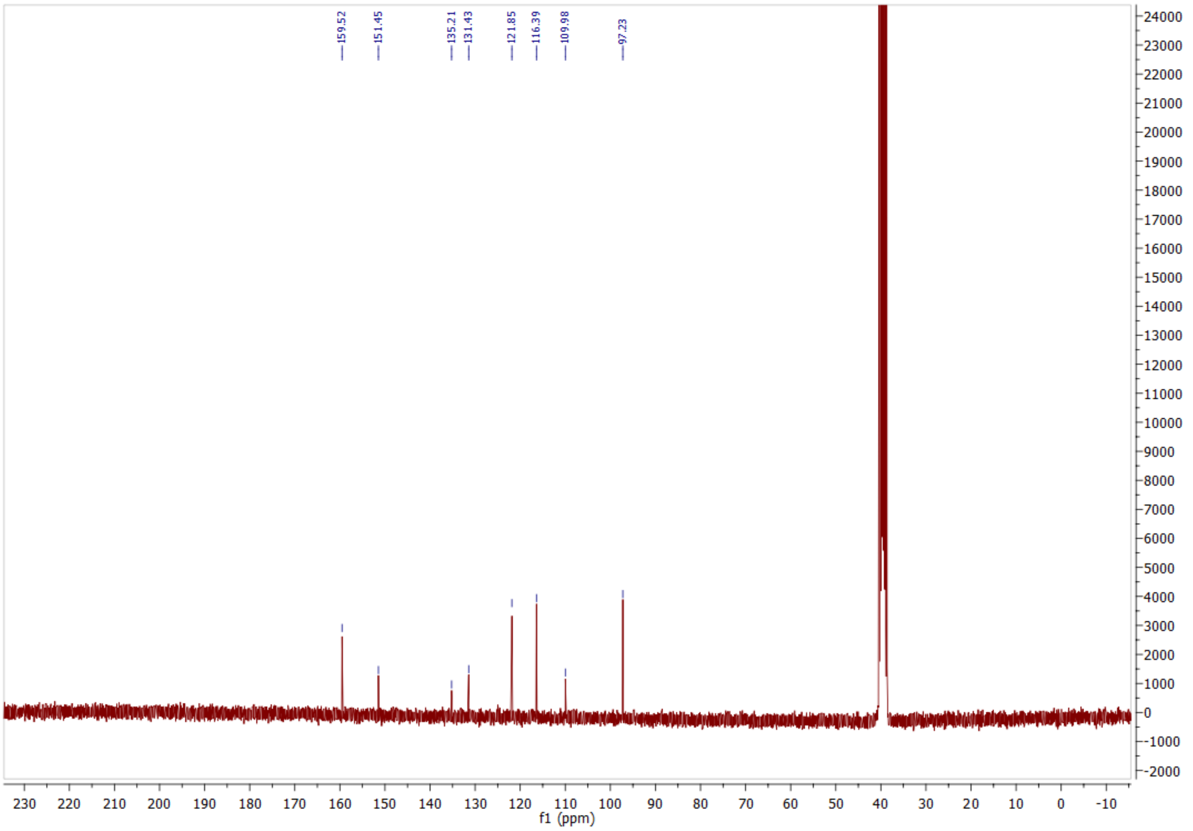
159.52
151.45
135.21
131.43
121.85
116.39
109.98
97.23
f1 (ppm)

7-Hydroxy-1,3-benzoxazole-2-carbonitrile (13)

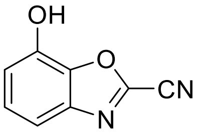

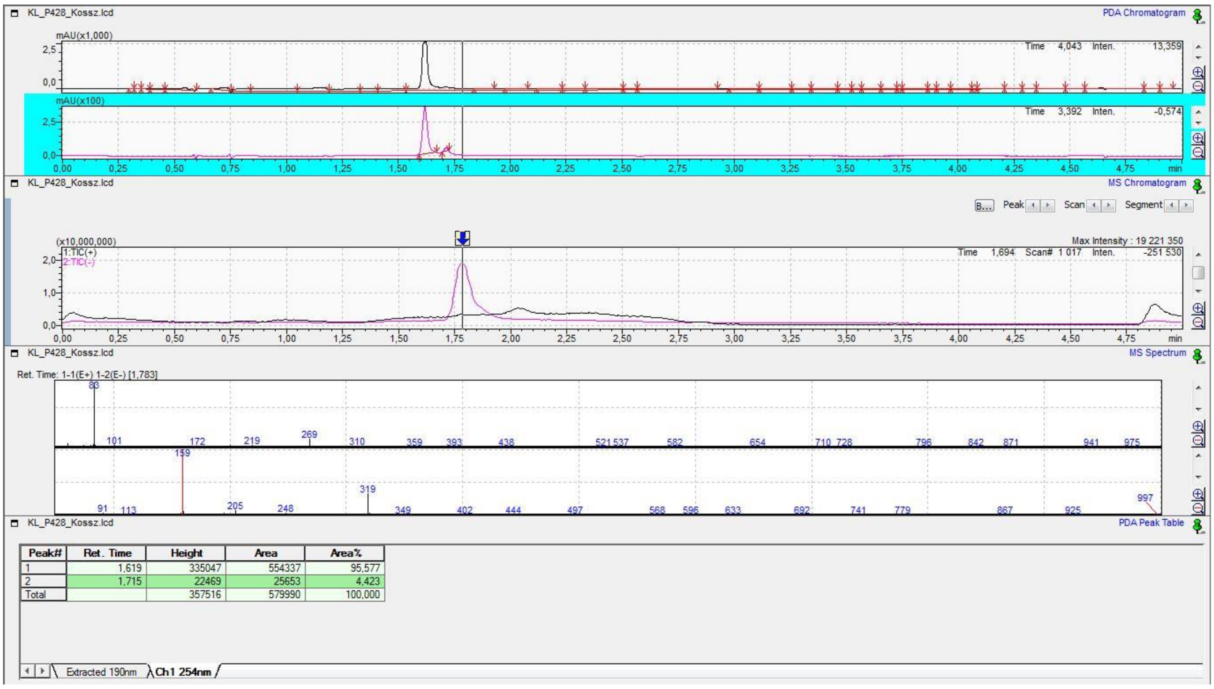

Peak#	Ret. Time	Height	Area	Area%
1	1,619	335047	554337	95,577
2	1,715	22469	25653	4,423
Total		357516	579990	100,000

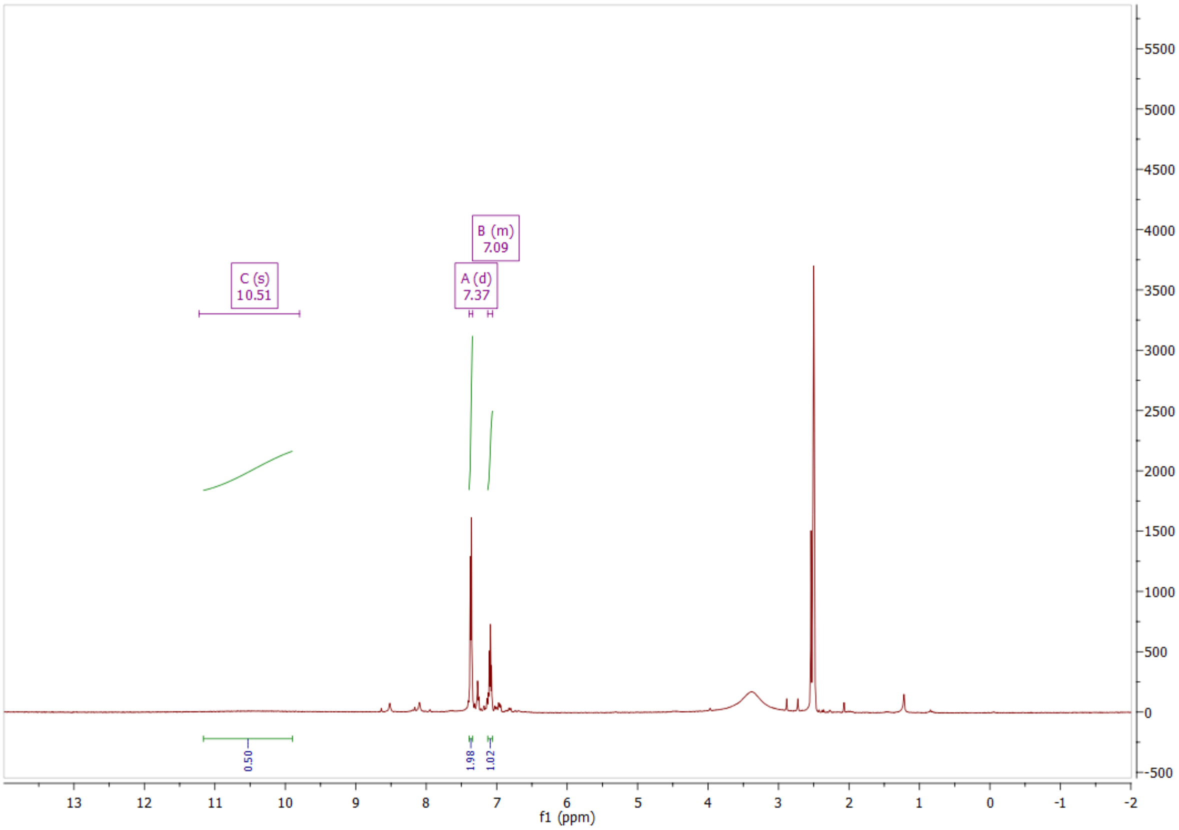

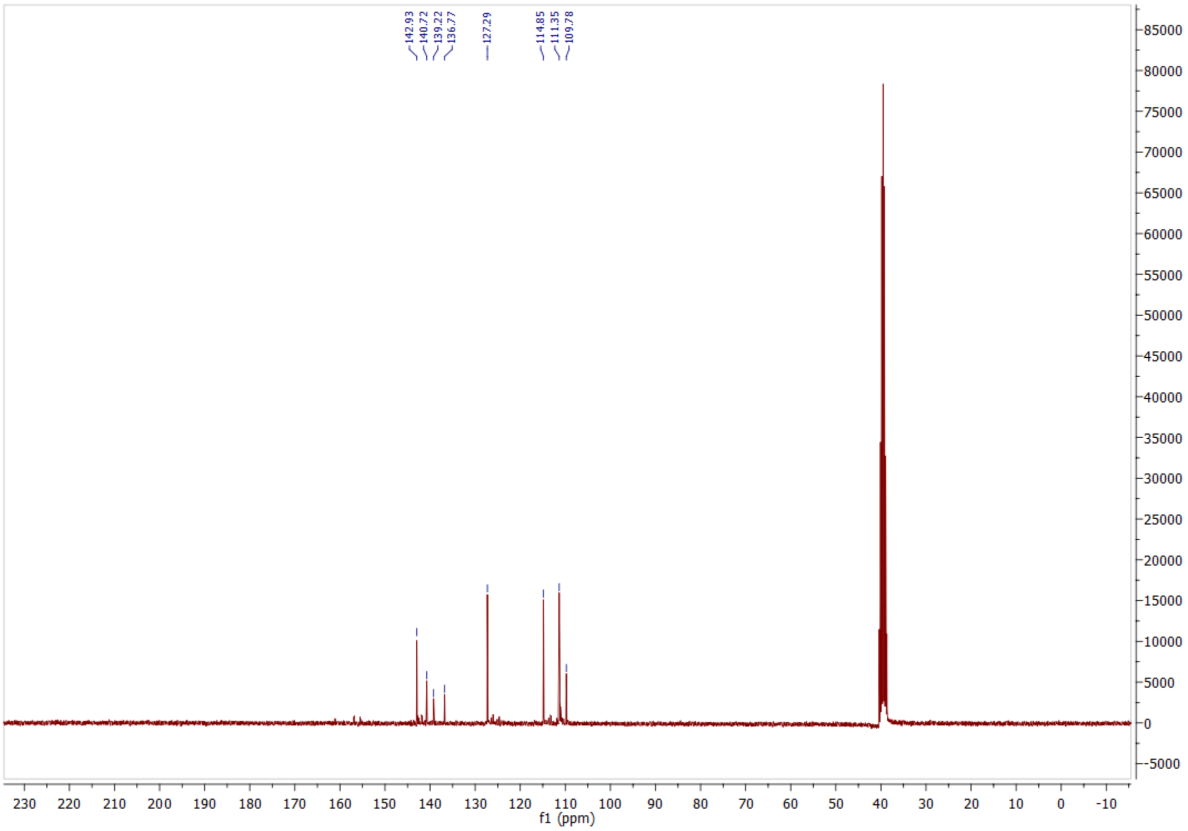

2-Cyano-1,3-benzoxazol-4-yl acetate (14)

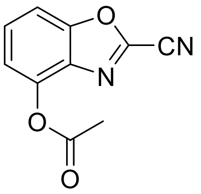

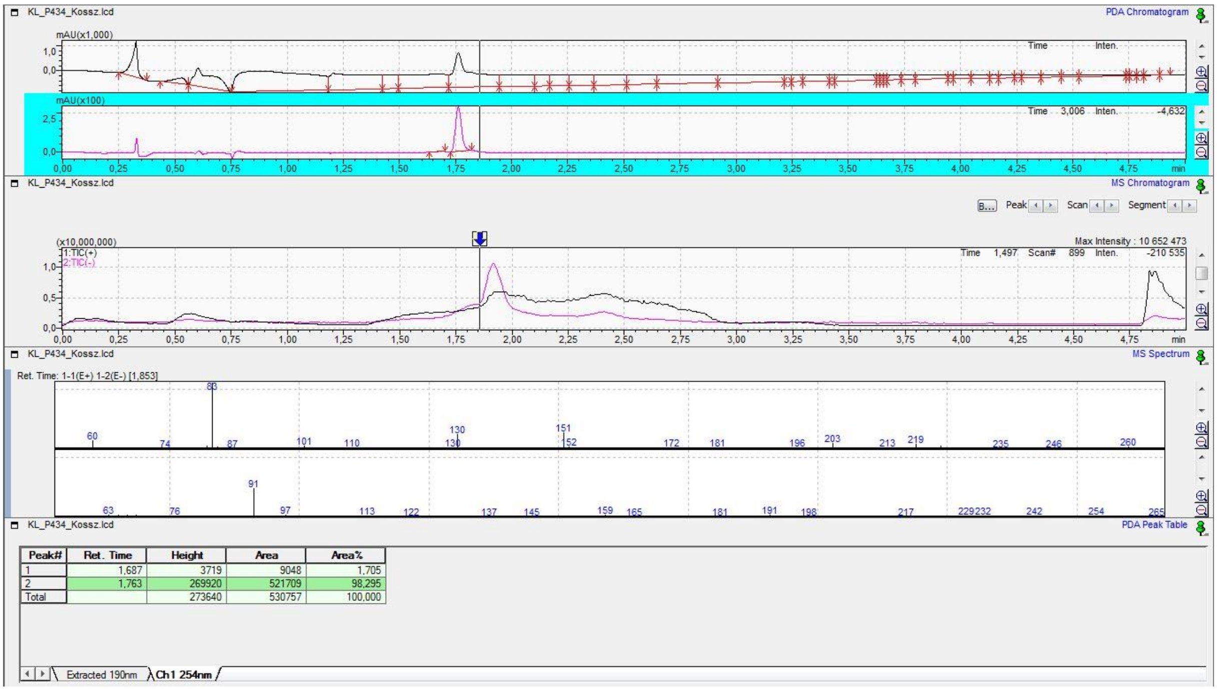

Peak#	Ret. Time	Height	Area	Area%
1	1,687	3719	9048	1,705
2	1,763	269920	521709	98,295
Total		273640	530757	100,000

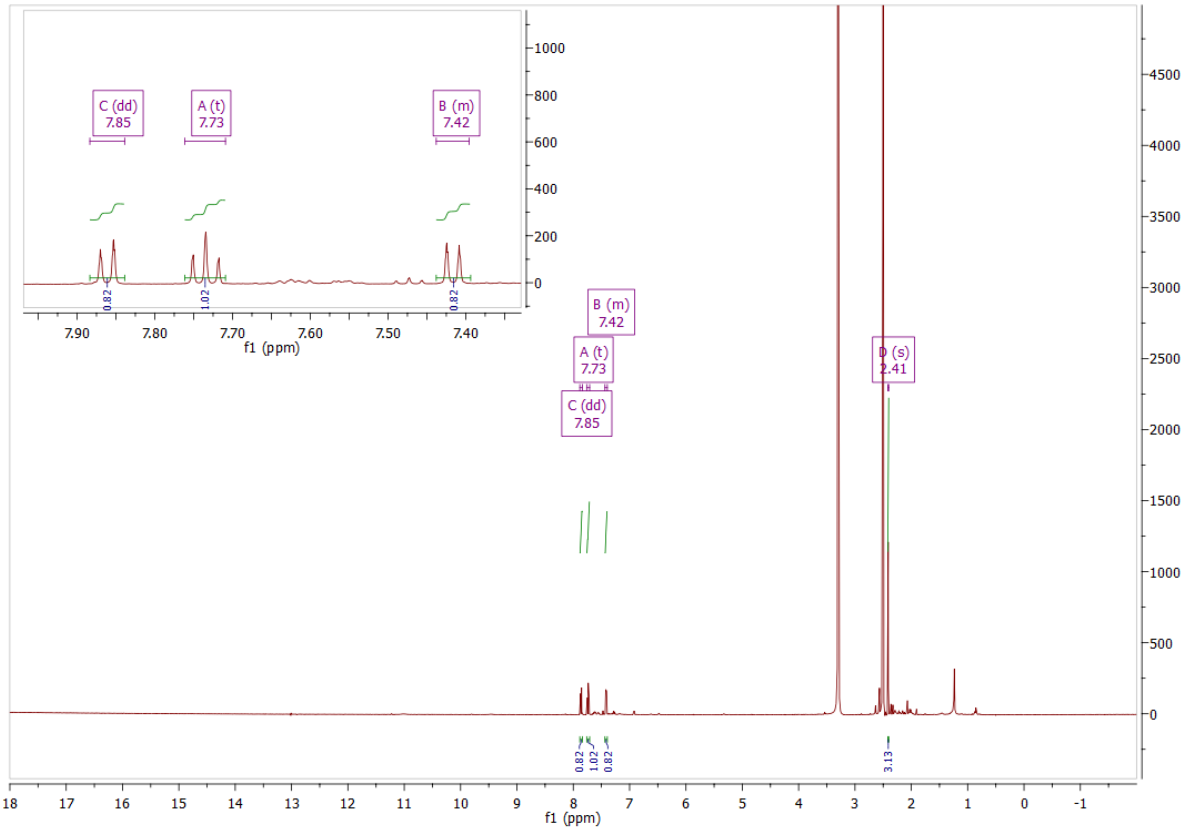
C (dd)
7.85
A (t)
7.73
B (m)
7.42
D (s)
2.41
0.82
1.02
0.82
3.13
f1 (ppm)

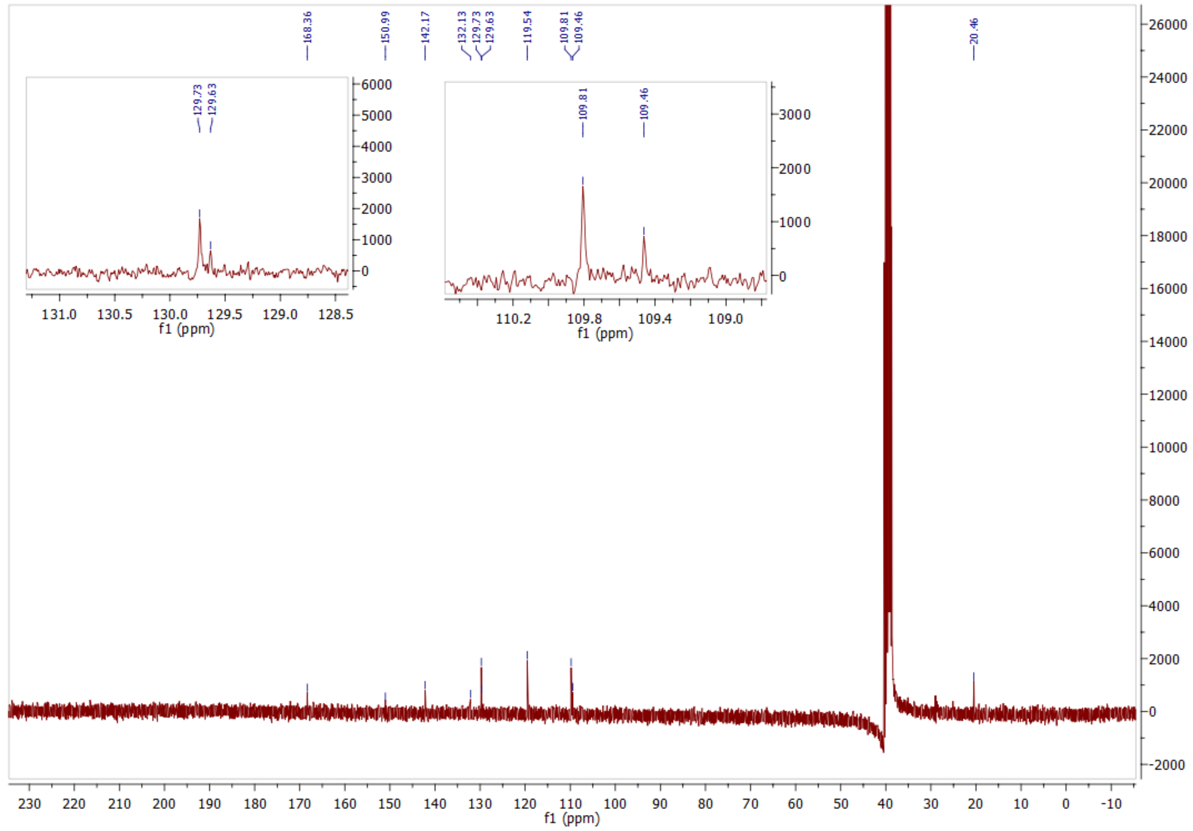
168.36
150.99
142.17
132.13
129.73
129.63
119.54
109.81
109.46
20.46
f1 (ppm)

2-Cyano-1,3-benzoxazol-5-yl acetate (15)

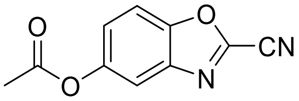

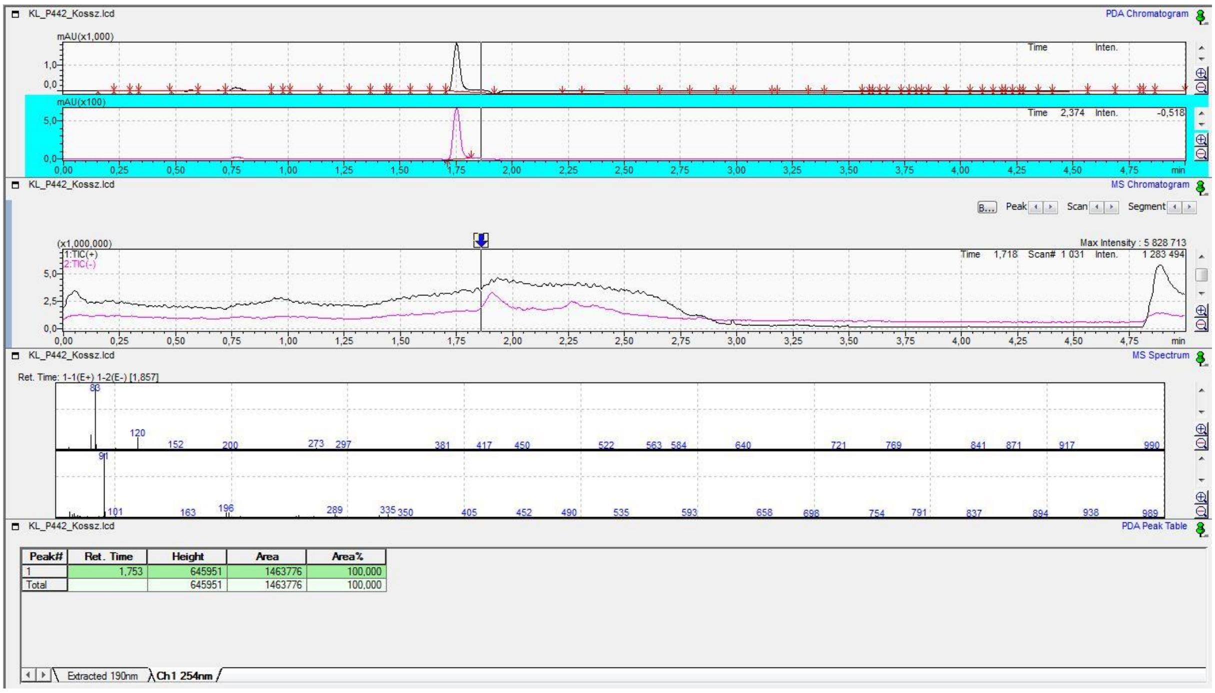

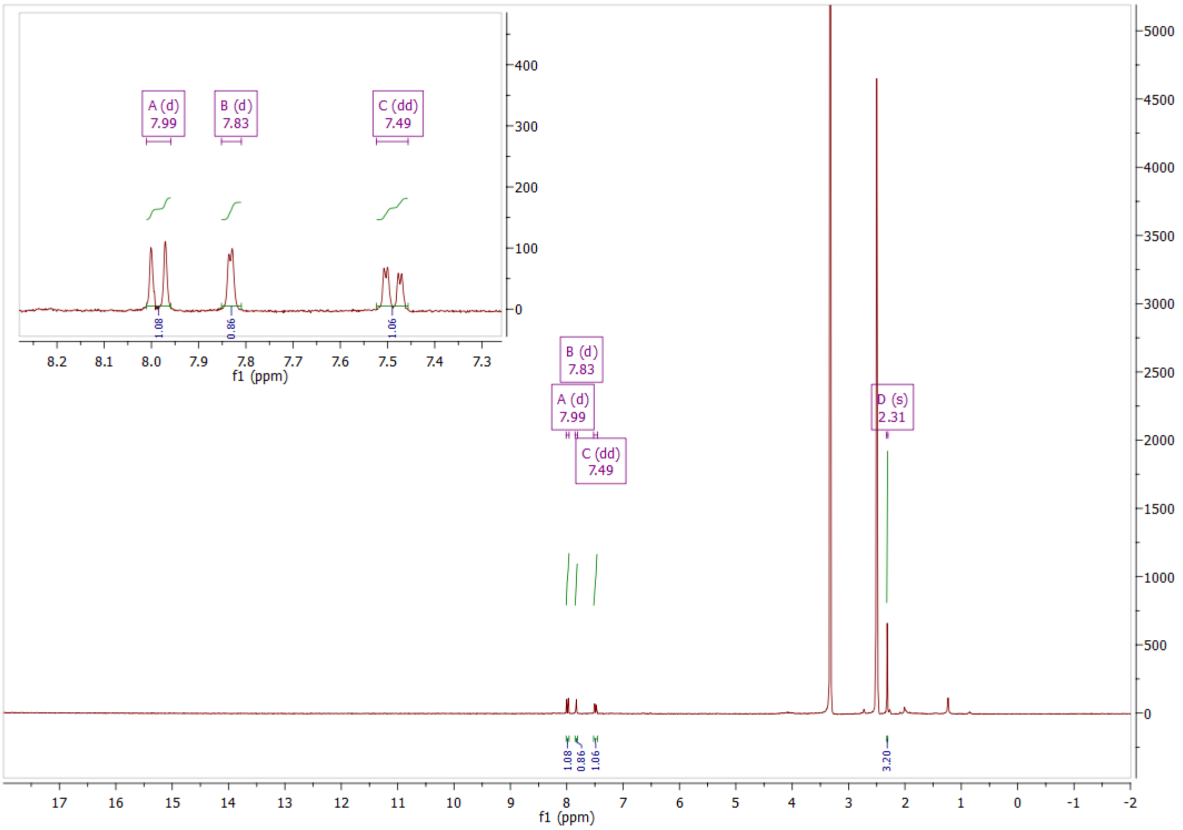

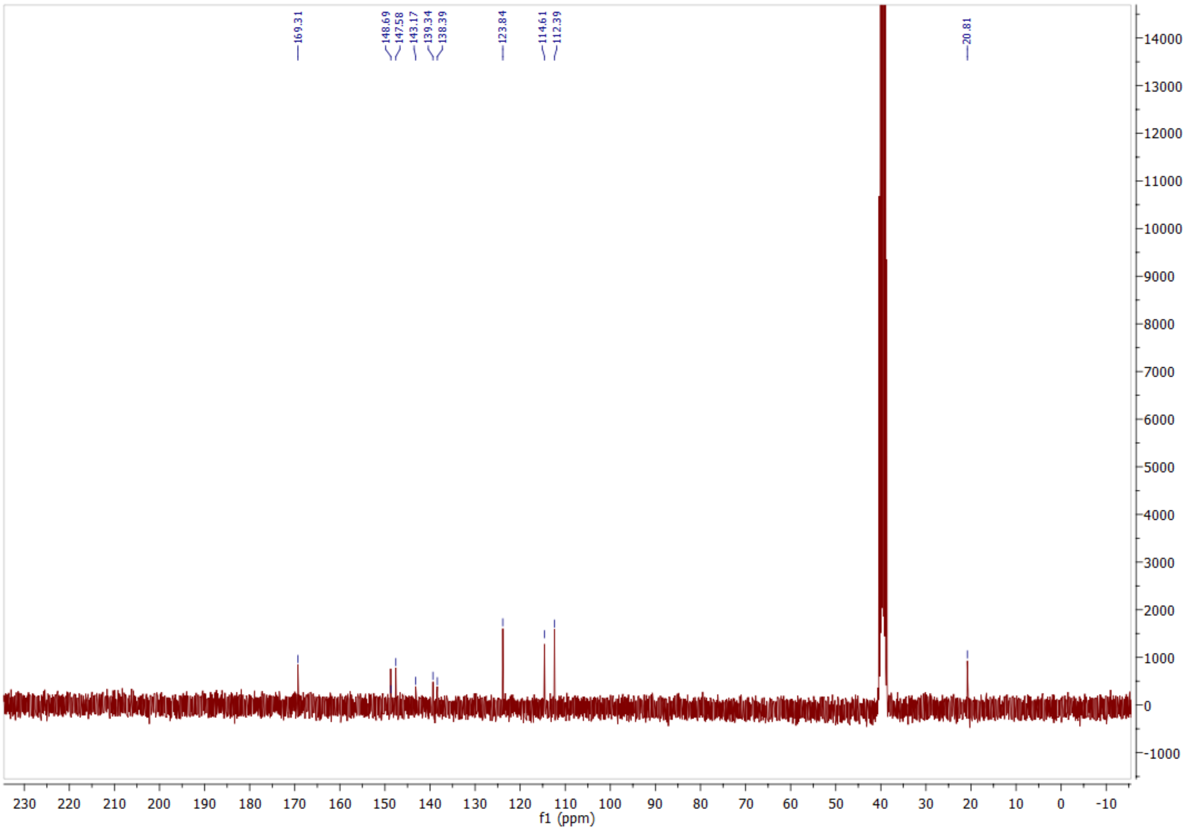

4-Nitro-1,3-benzoxazole-2-carbonitrile (16)

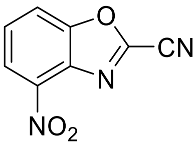

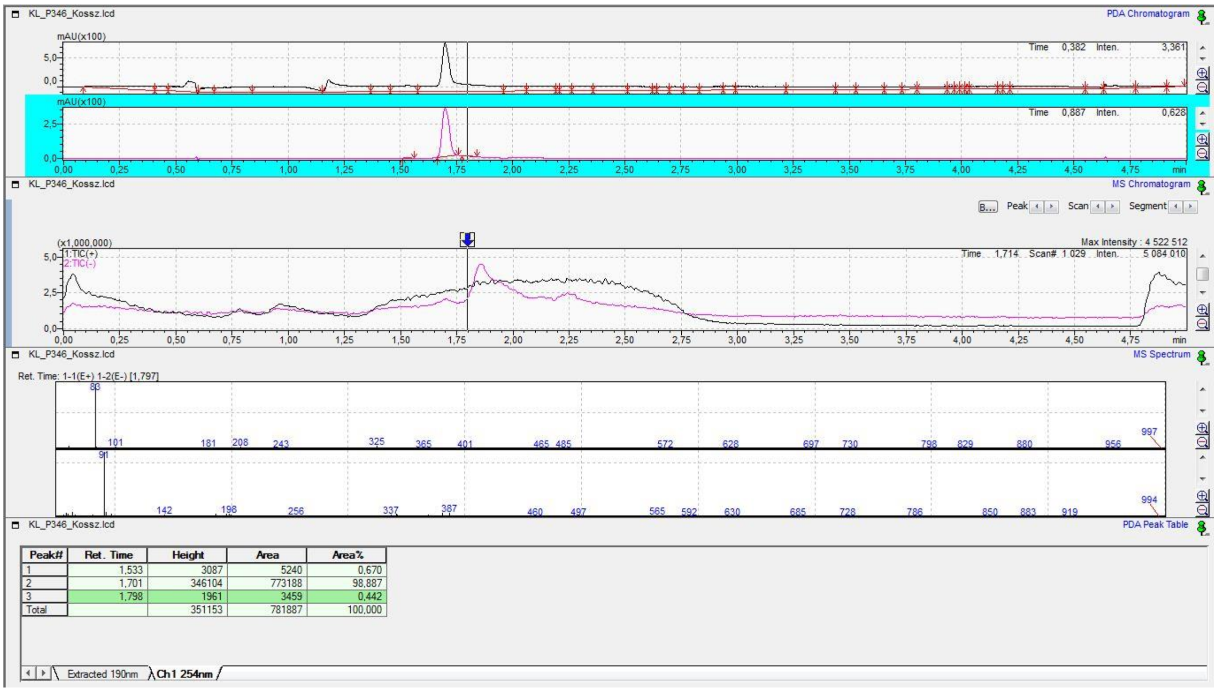

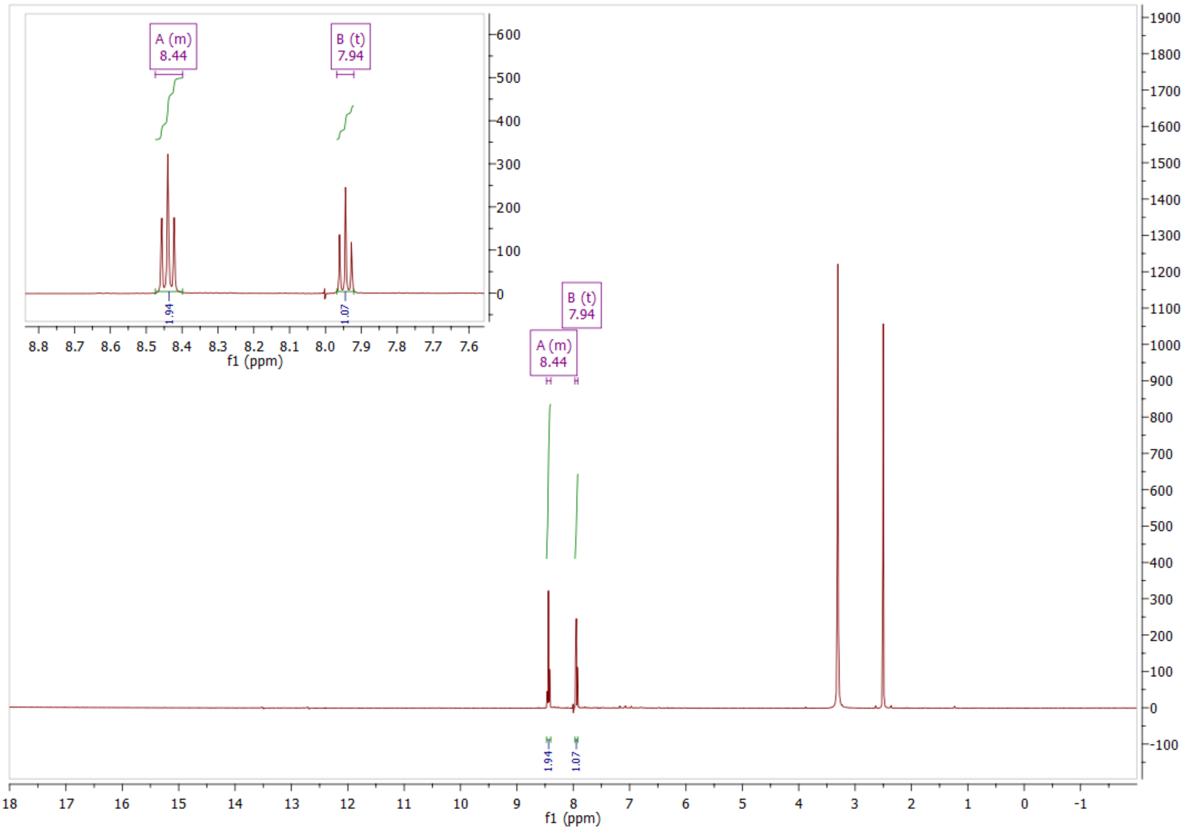
A (m)
8.44
B (t)
7.94
1.94
1.07
f1 (ppm)

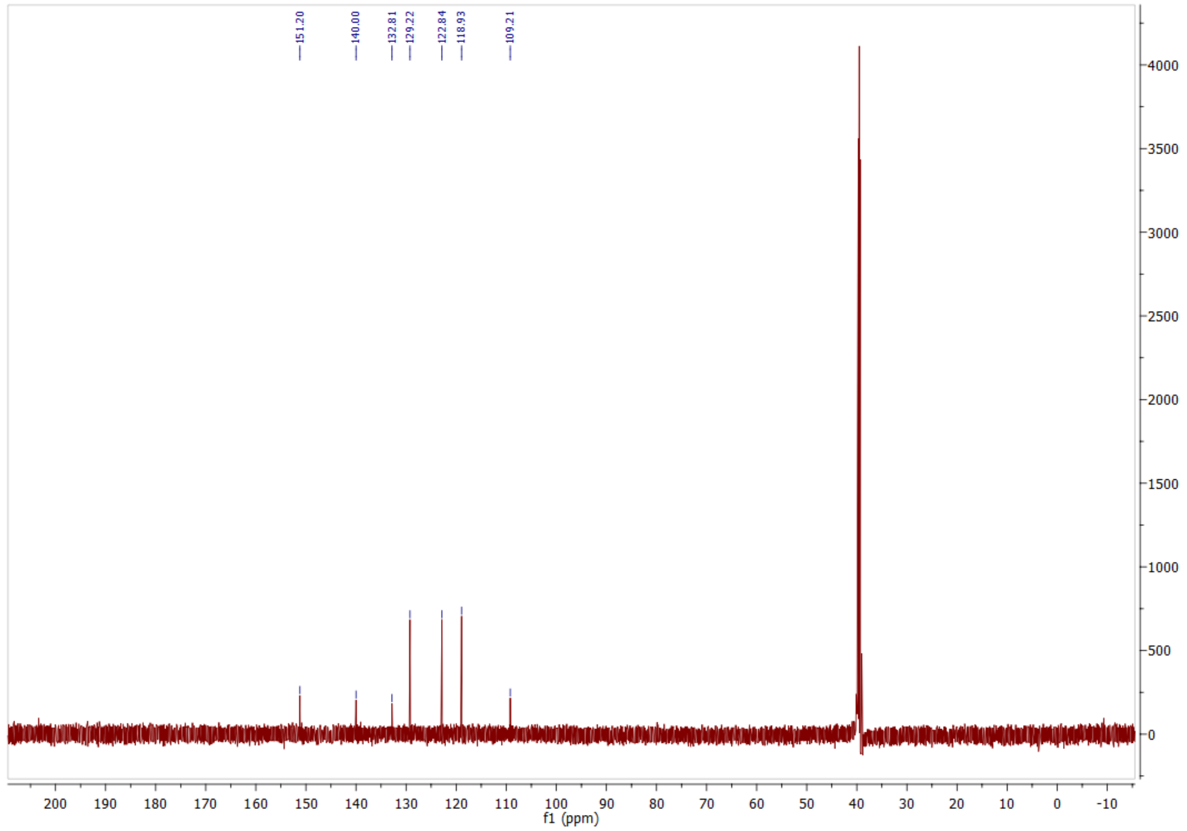
151.20
140.00
132.81
129.22
122.84
118.93
109.21
f1 (ppm)

5-Nitro-1,3-benzoxazole-2-carbonitrile (17)

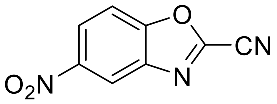

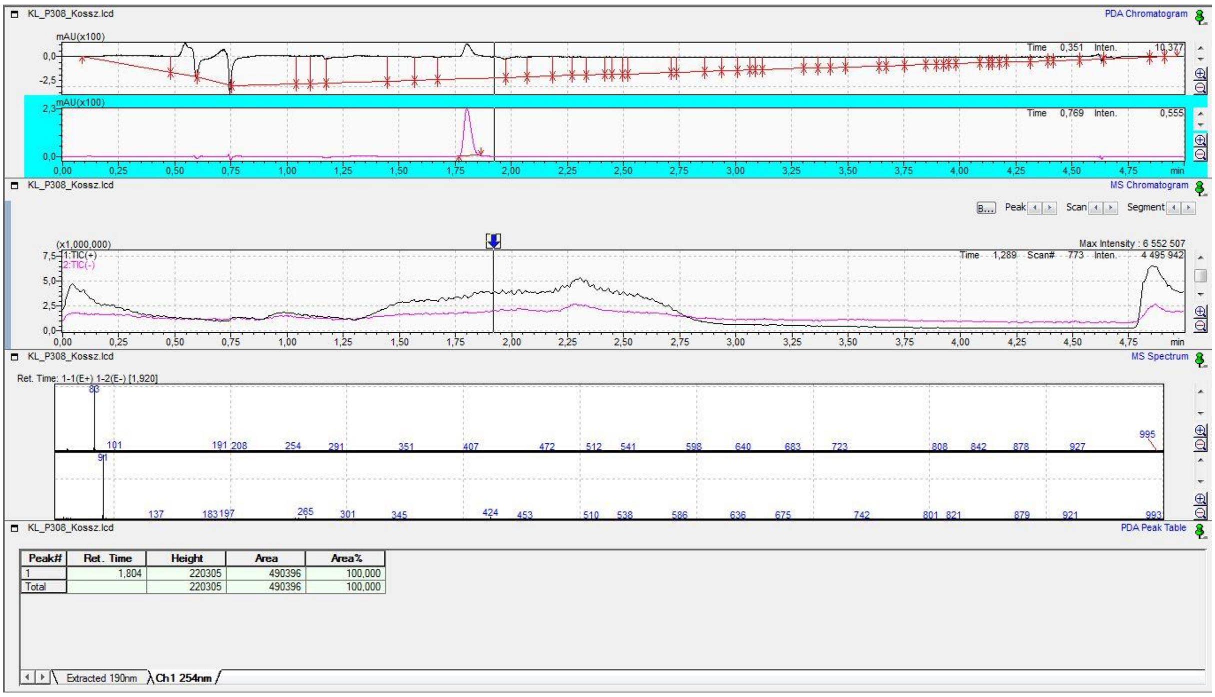

Peak#	Ret. Time	Height	Area	Area%
1	1,804	220305	490396	100,000
Total		220305	490396	100,000

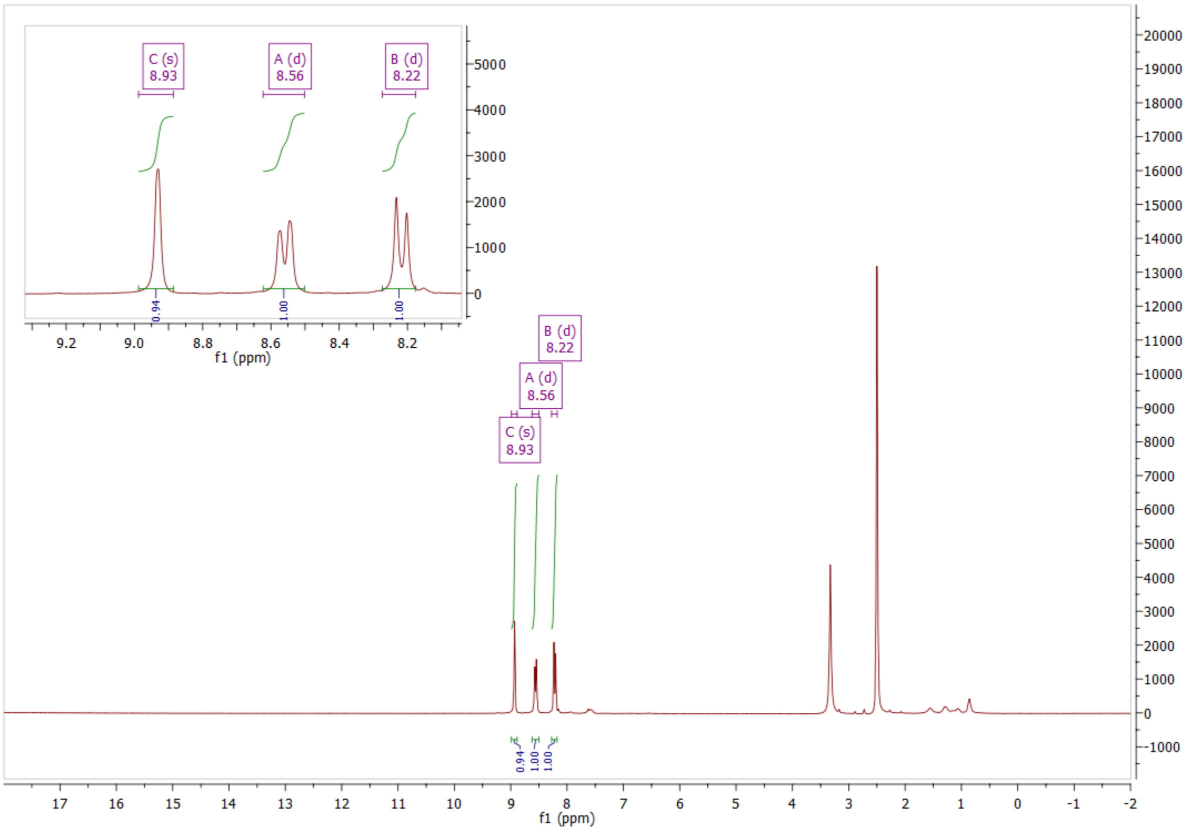

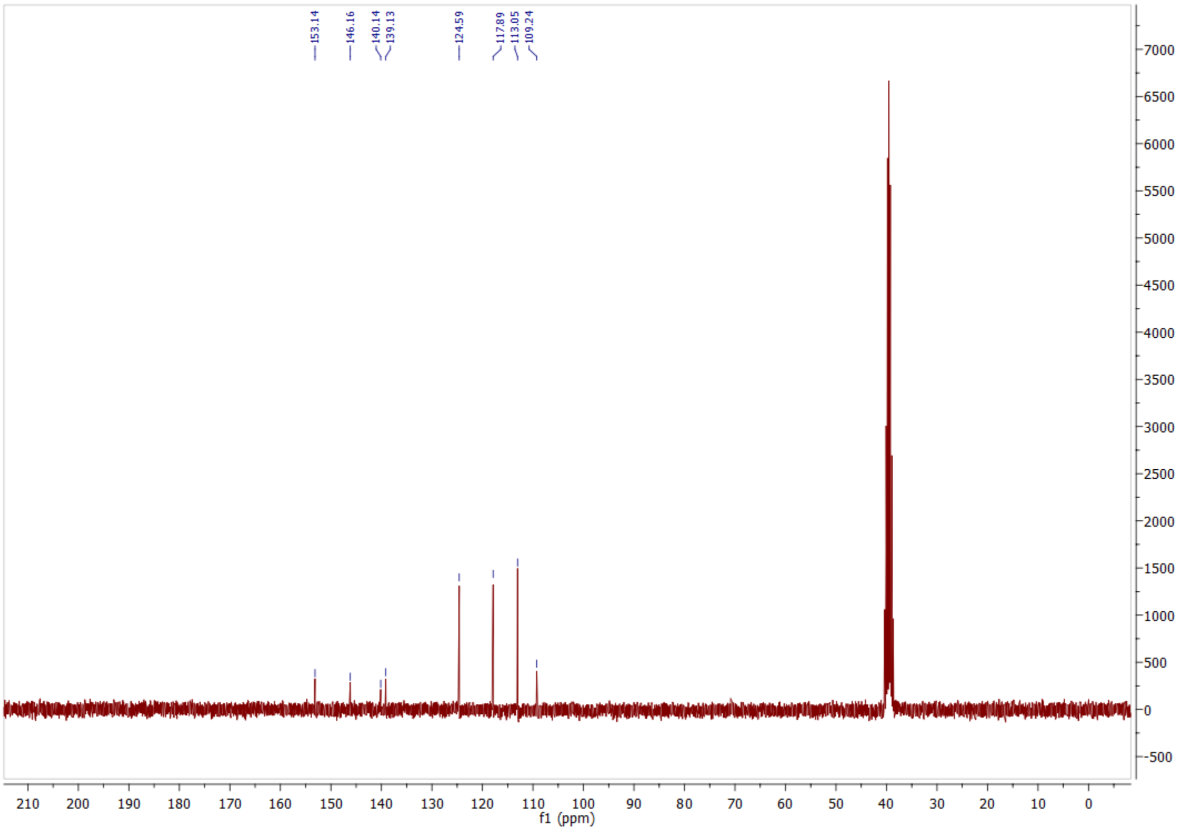

6-Nitro-1,3-benzoxazole-2-carbonitrile (18)

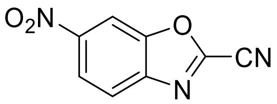

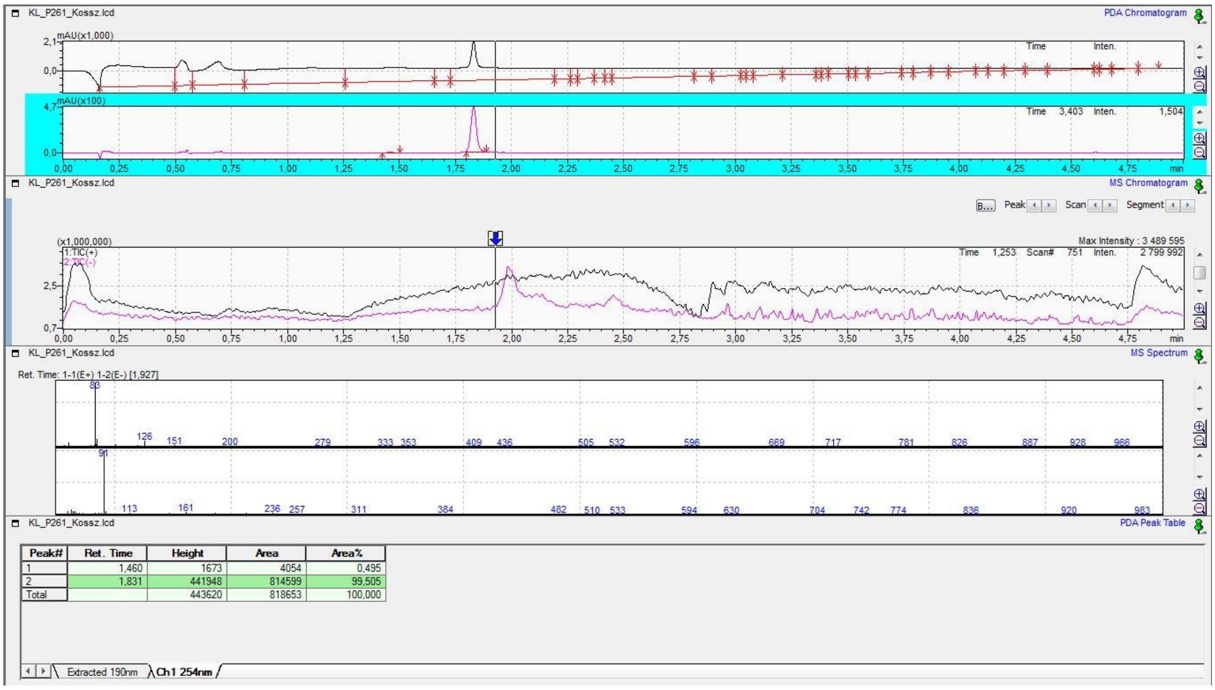

Peak#	Ret. Time	Height	Area	Area%
1	1,460	1673	4054	0,495
2	1,831	441948	814599	99,505
Total		443620	818653	100,000

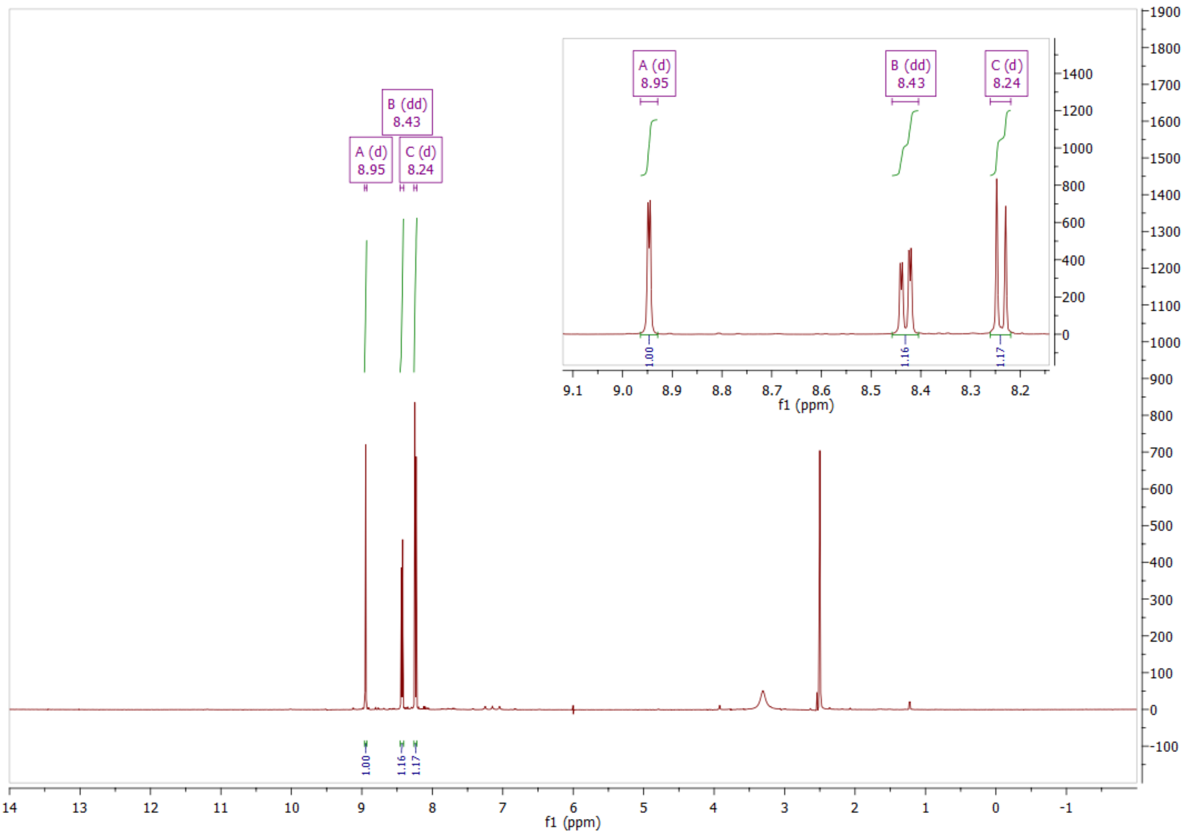
A (d)
8.95
B (dd)
8.43
C (d)
8.24
f1 (ppm)

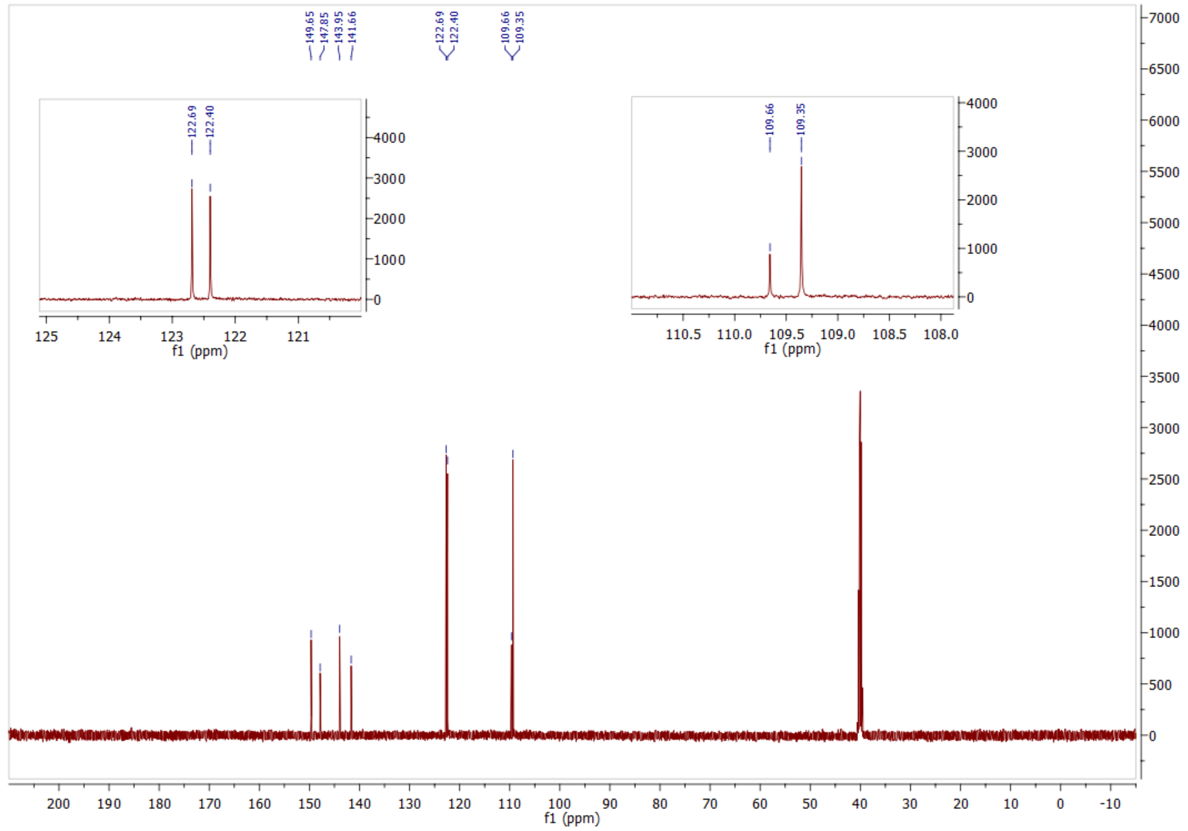
149.65
147.85
143.95
141.66
122.69
122.40
109.66
109.35
f1 (ppm)

7-Nitro-1,3-benzoxazole-2-carbonitrile (19)

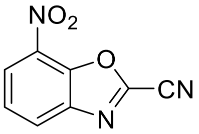

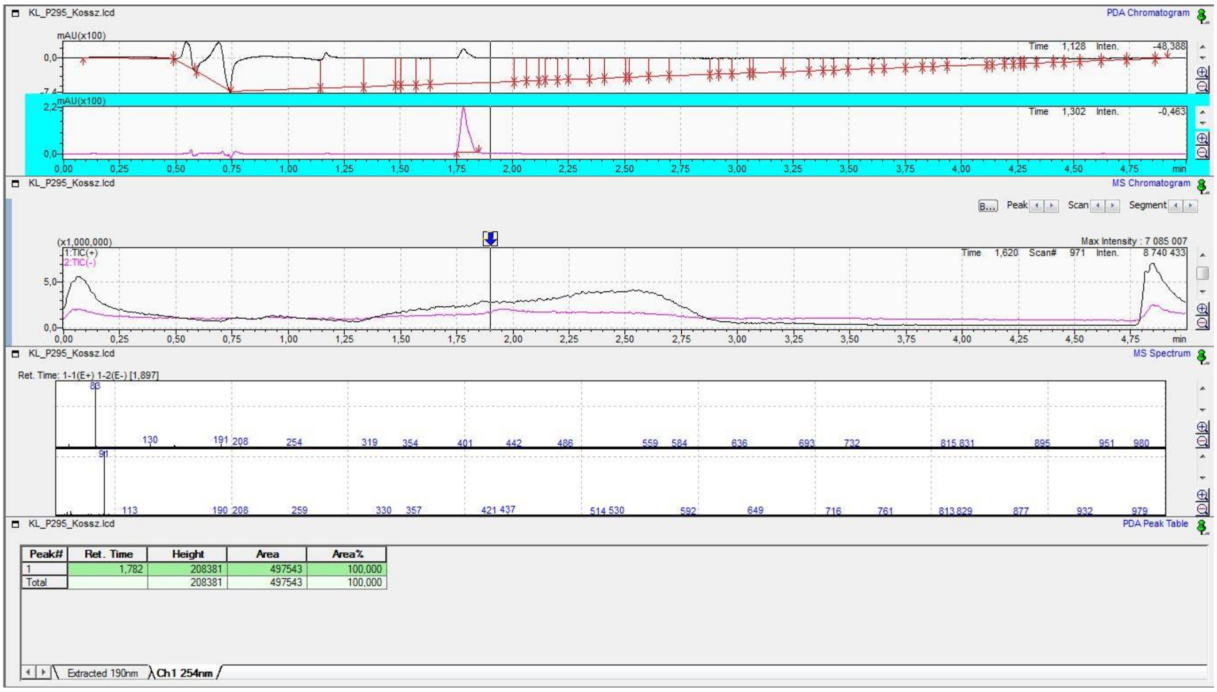

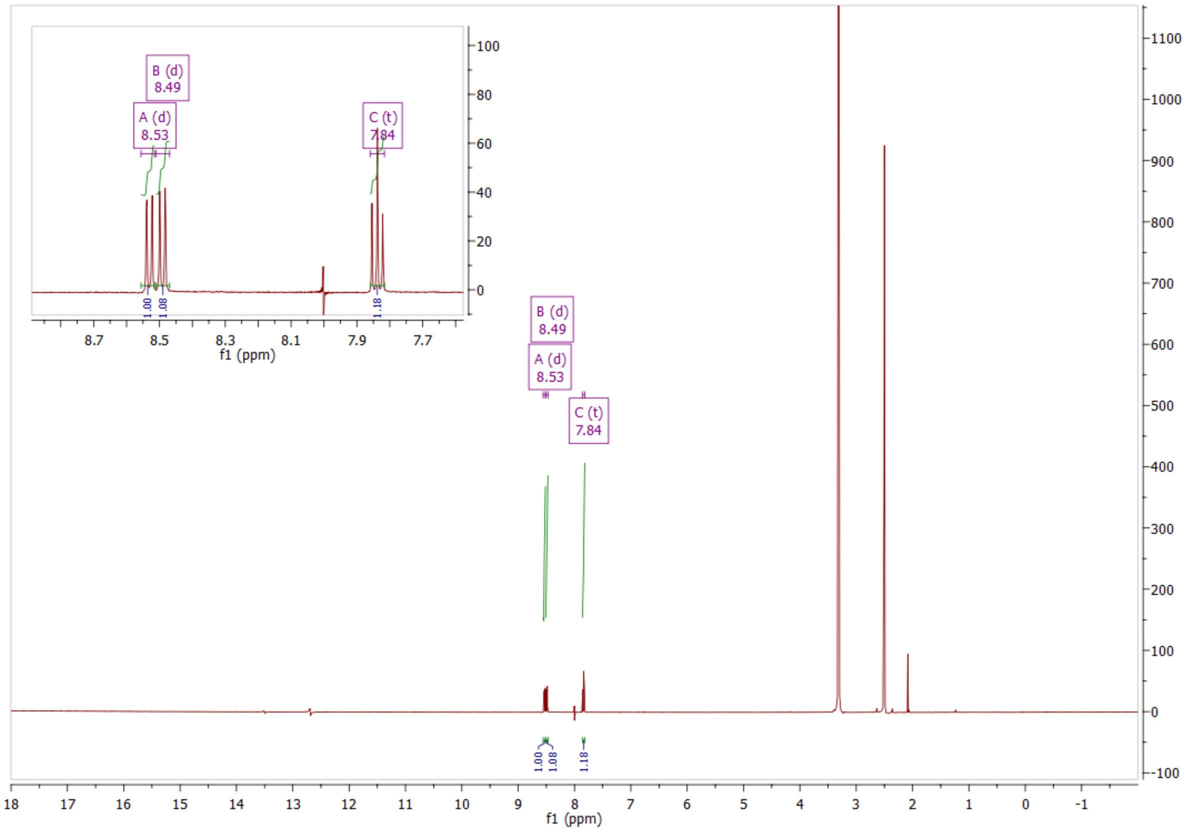

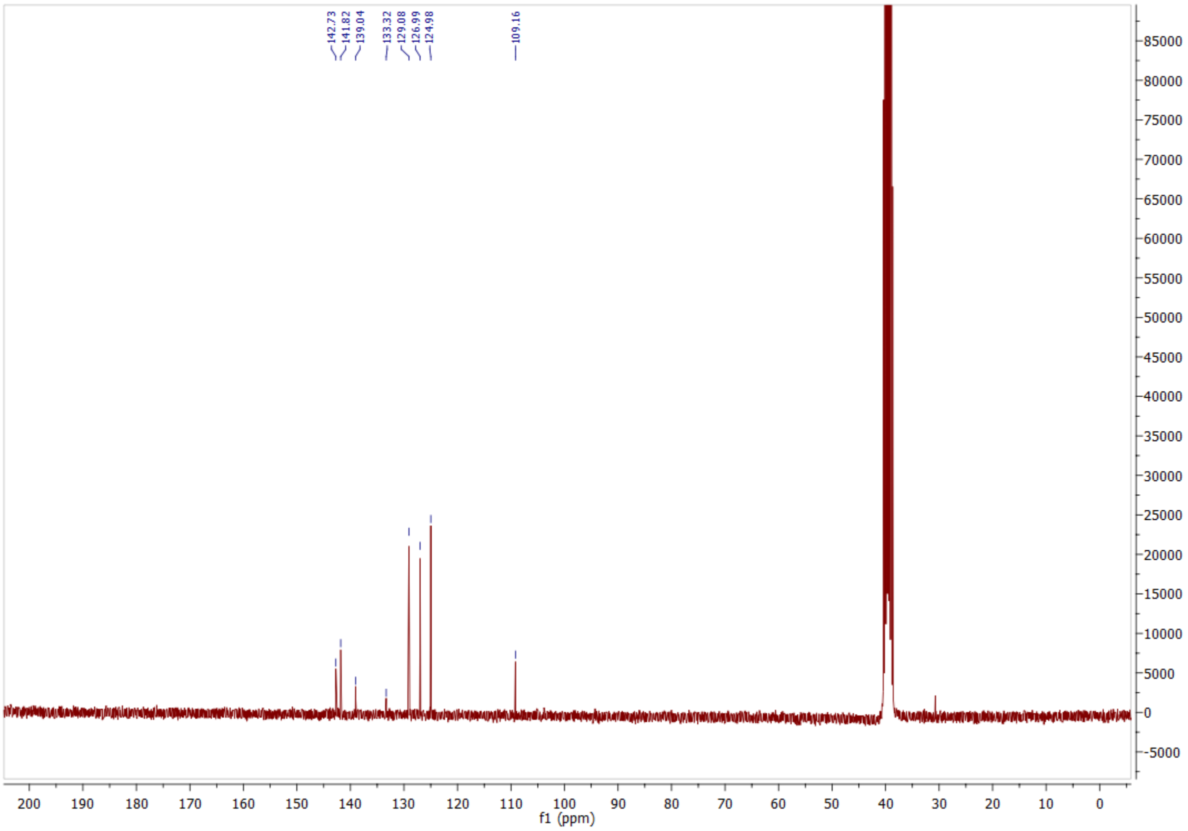

4-Amino-1,3-benzoxazole-2-carbonitrile (20)

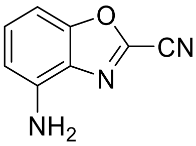

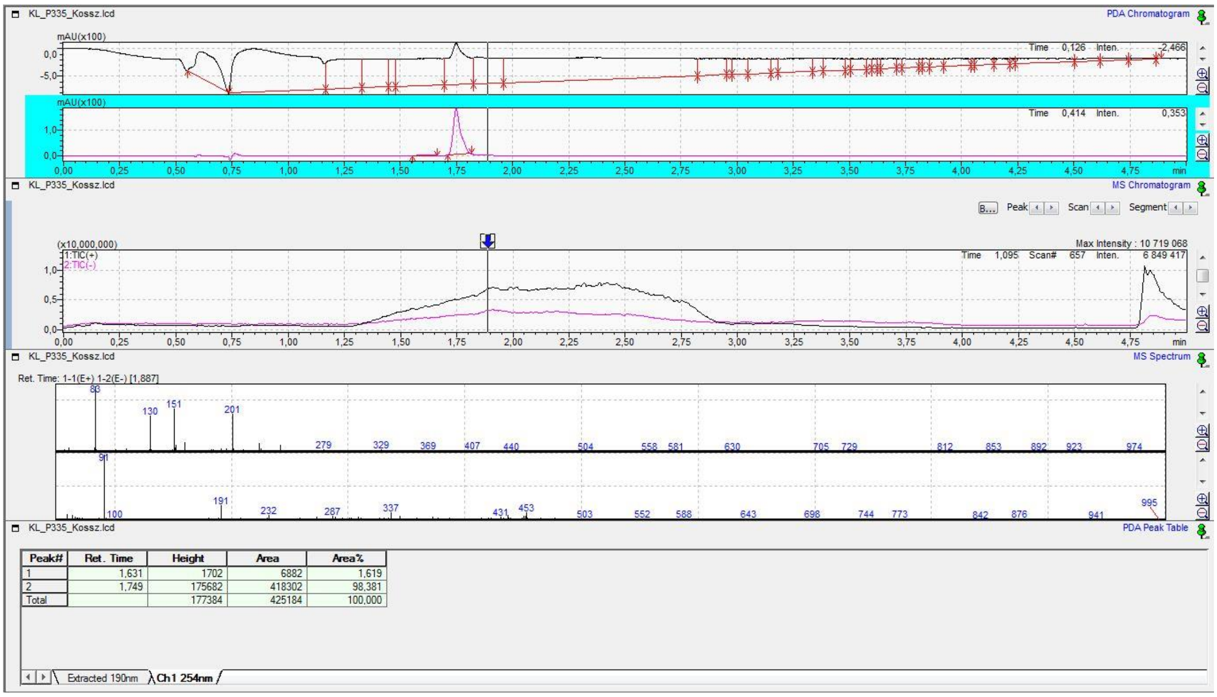

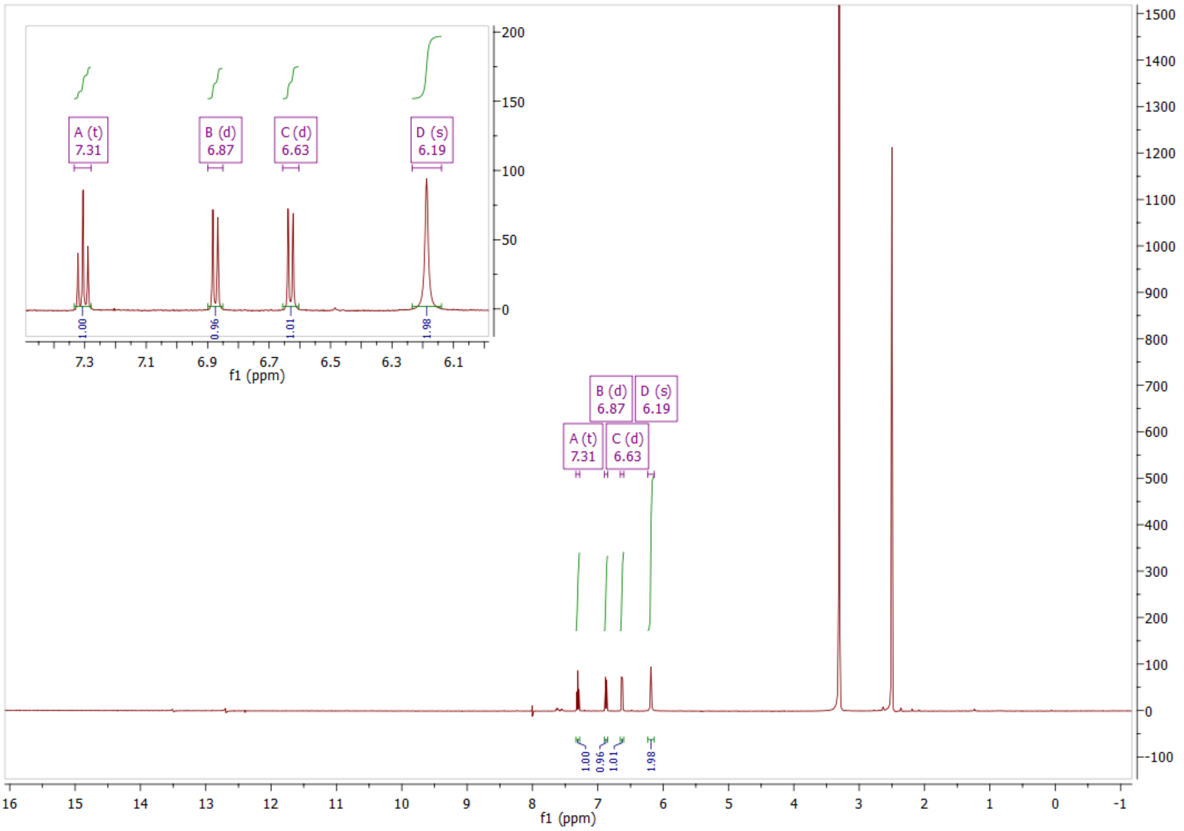
A (t)
7.31
B (d)
6.87
C (d)
6.63
D (s)
6.19
1.00
0.96
1.01
1.98
f1 (ppm)

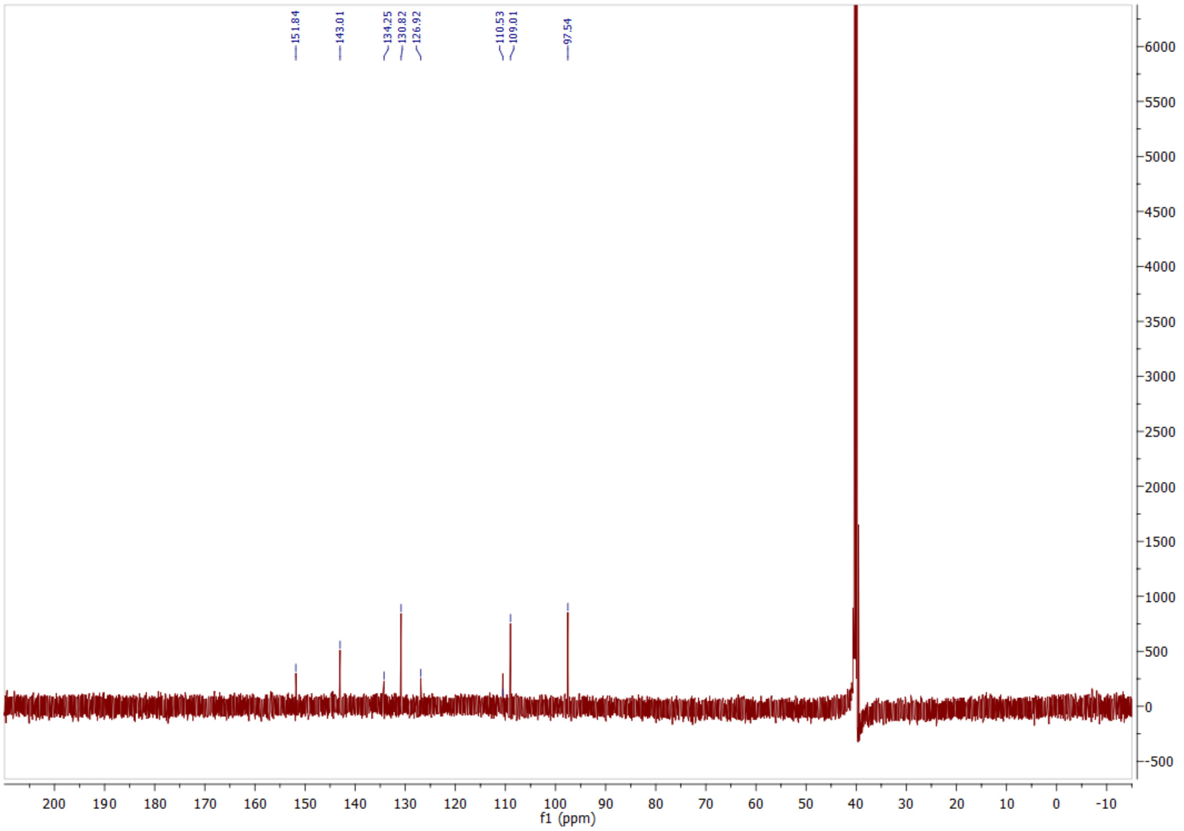
151.84
143.01
134.25
130.82
126.92
110.53
109.01
97.54
f1 (ppm)

5-Amino-1,3-benzoxazole-2-carbonitrile (21)

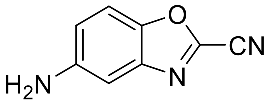

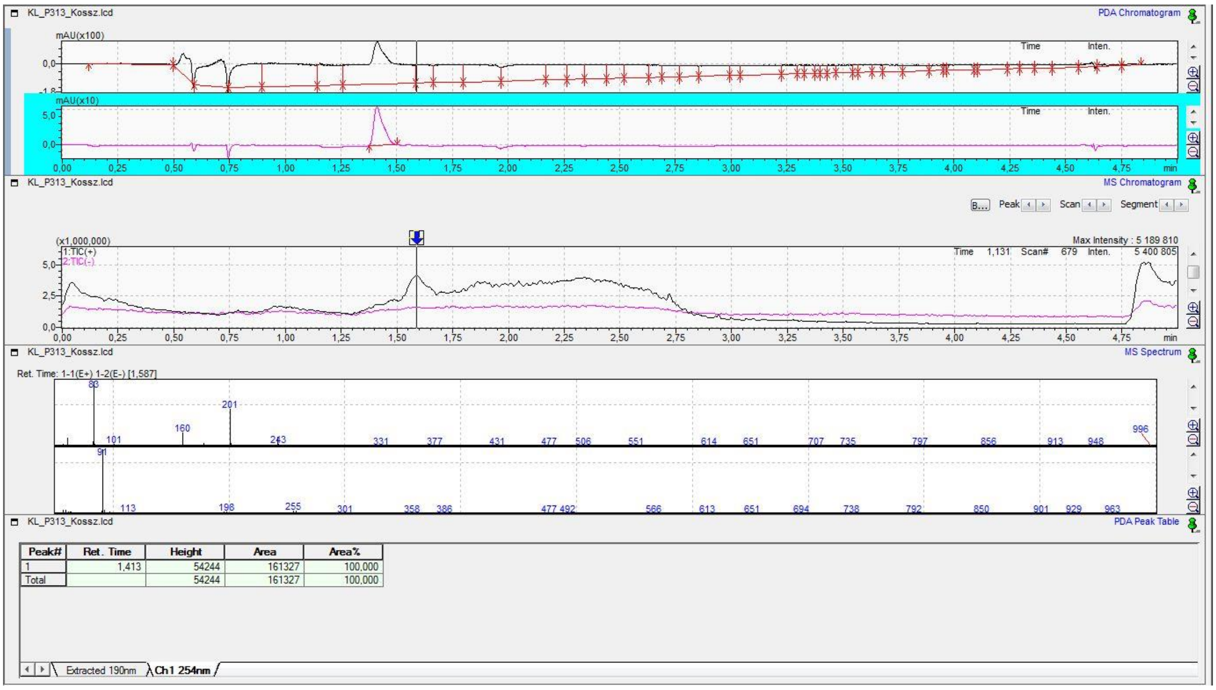

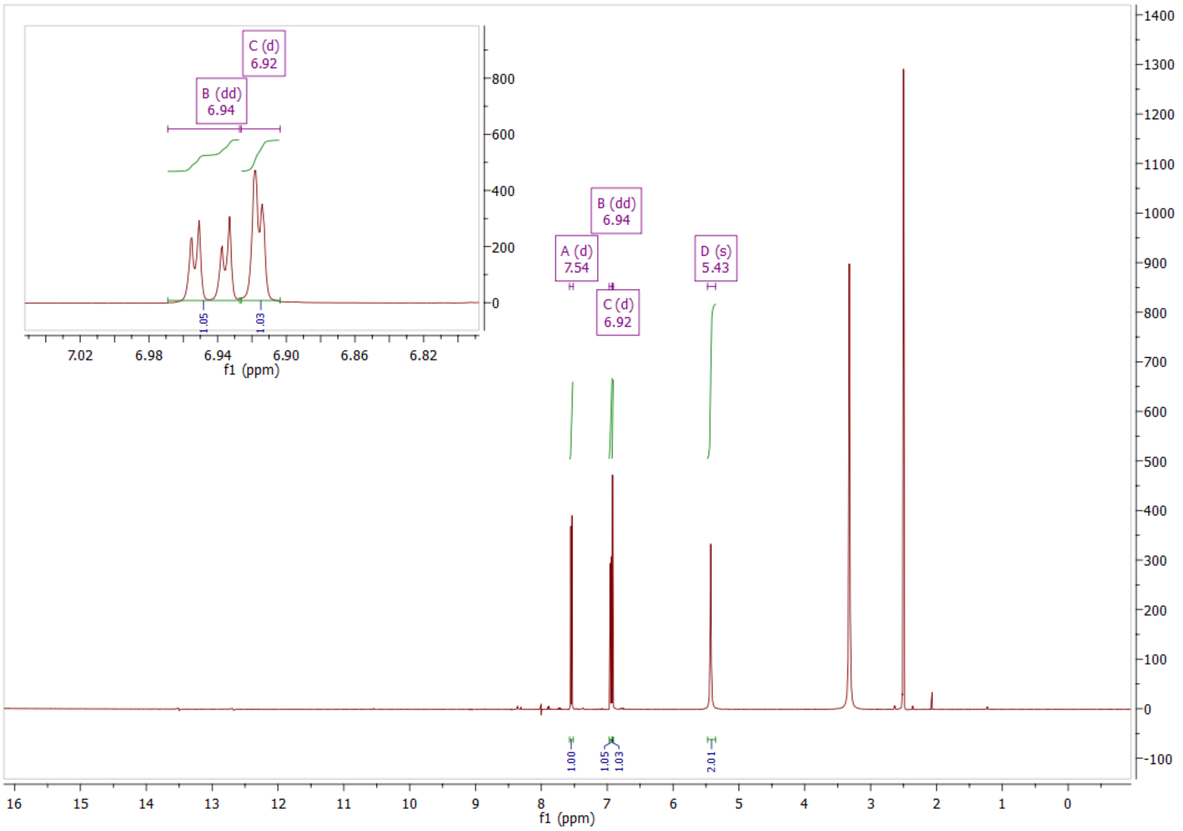

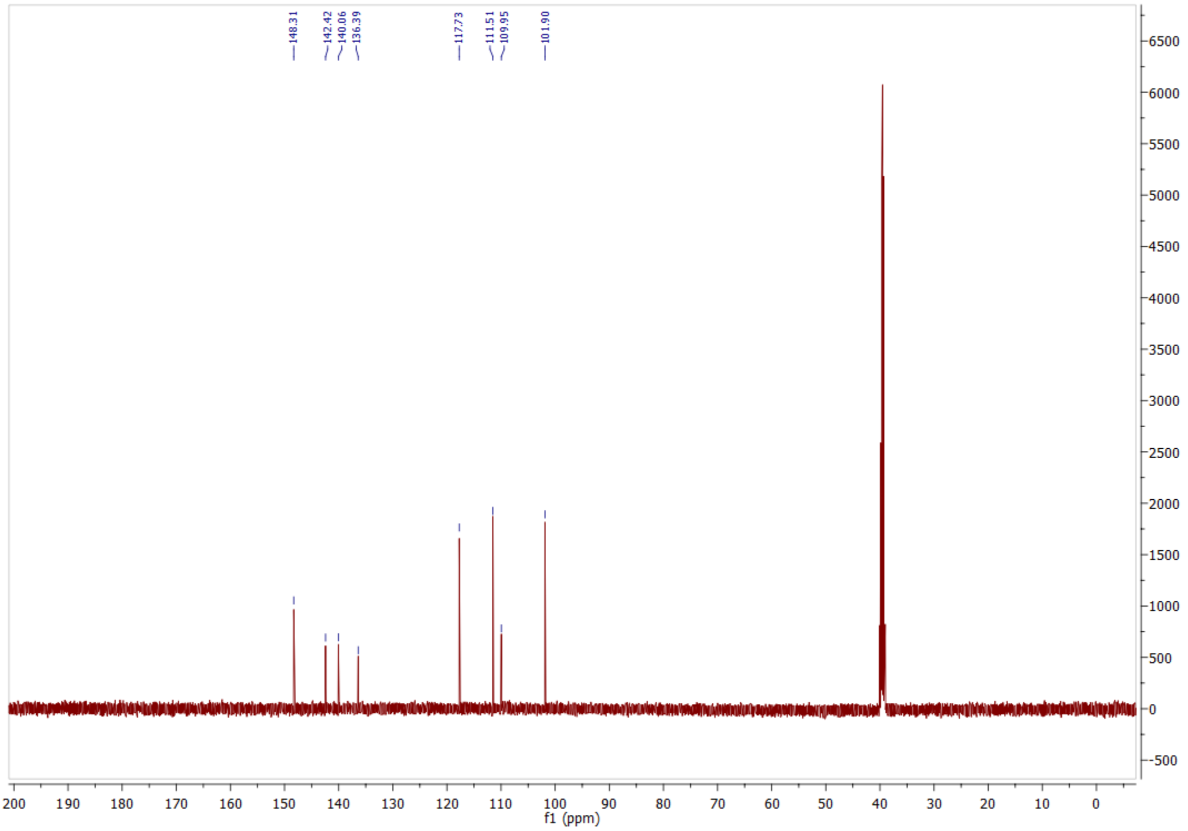

6-Amino-1,3-benzoxazole-2-carbonitrile (22)

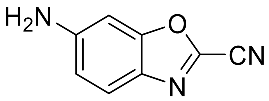

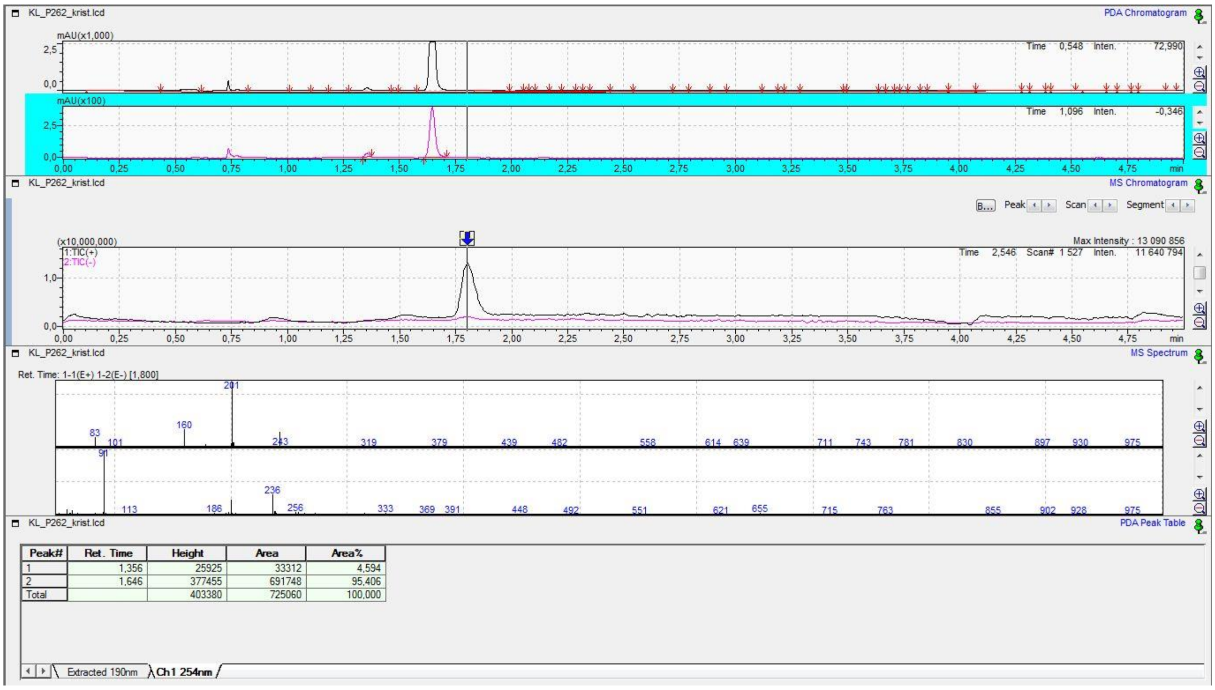

Peak#	Ret. Time	Height	Area	Area%
1	1,356	25925	33312	4,594
2	1,646	377455	691748	95,406
Total		403380	725060	100,000

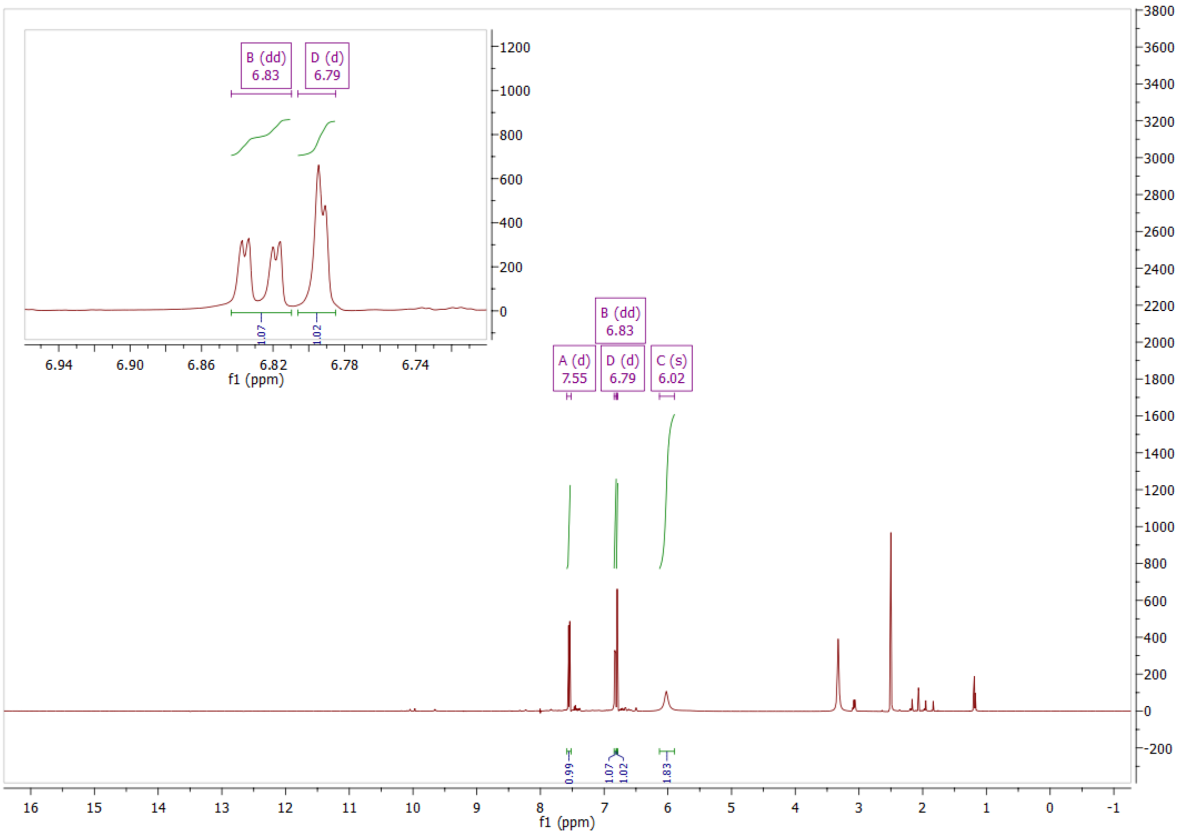
B (dd)
6.83
D (d)
6.79
1.07
1.02
6.94
6.90
6.86
6.82
6.78
6.74
f1 (ppm)
A (d)
7.55
B (dd)
6.83
D (d)
6.79
C (s)
6.02
0.99
1.07
1.02
1.83
f1 (ppm)

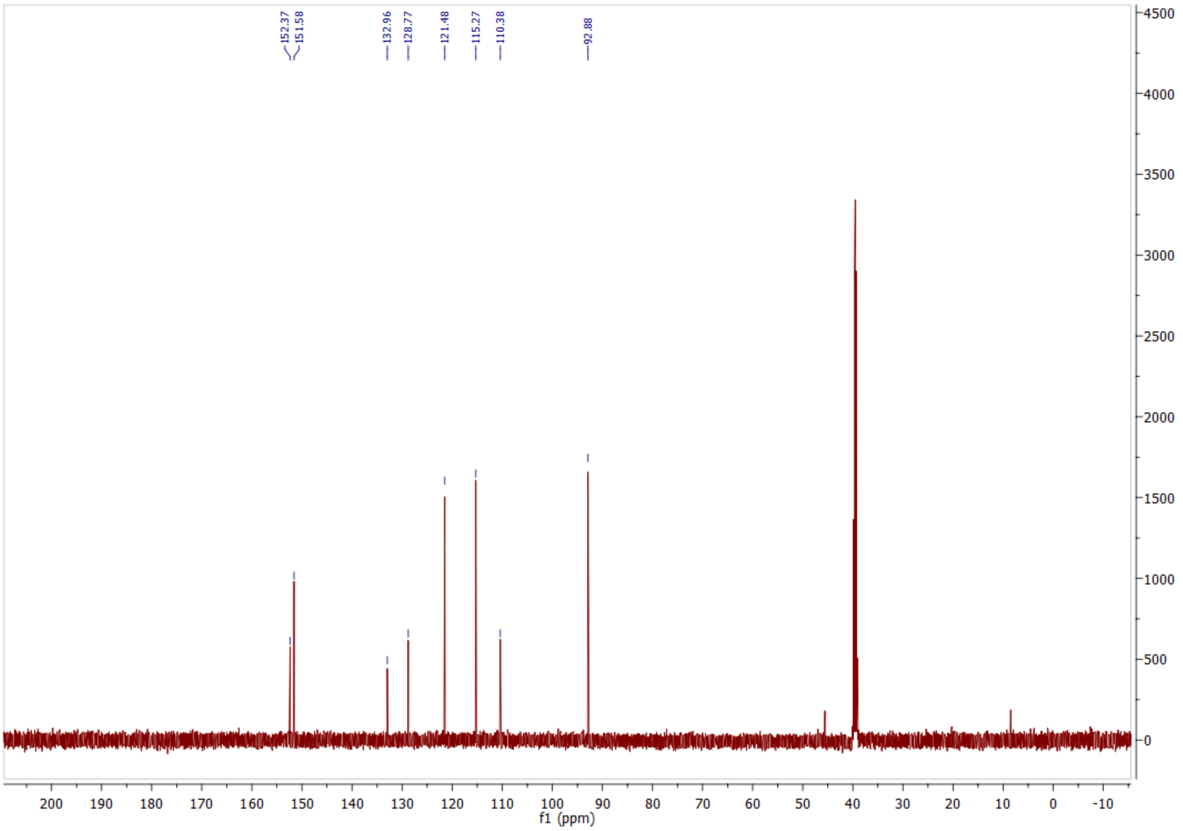
152.37
151.58
132.96
128.77
121.48
115.27
110.38
92.88
f1 (ppm)

7-Amino-1,3-benzoxazole-2-carbonitrile (23)

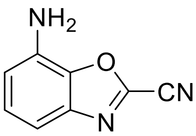

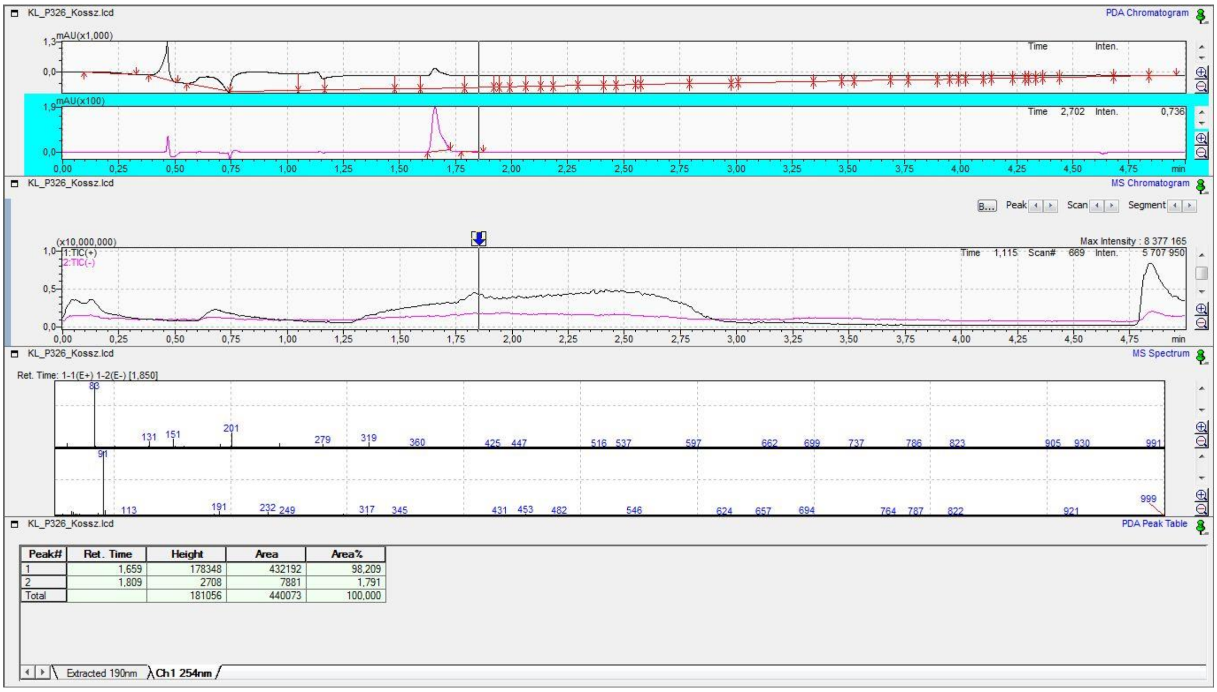

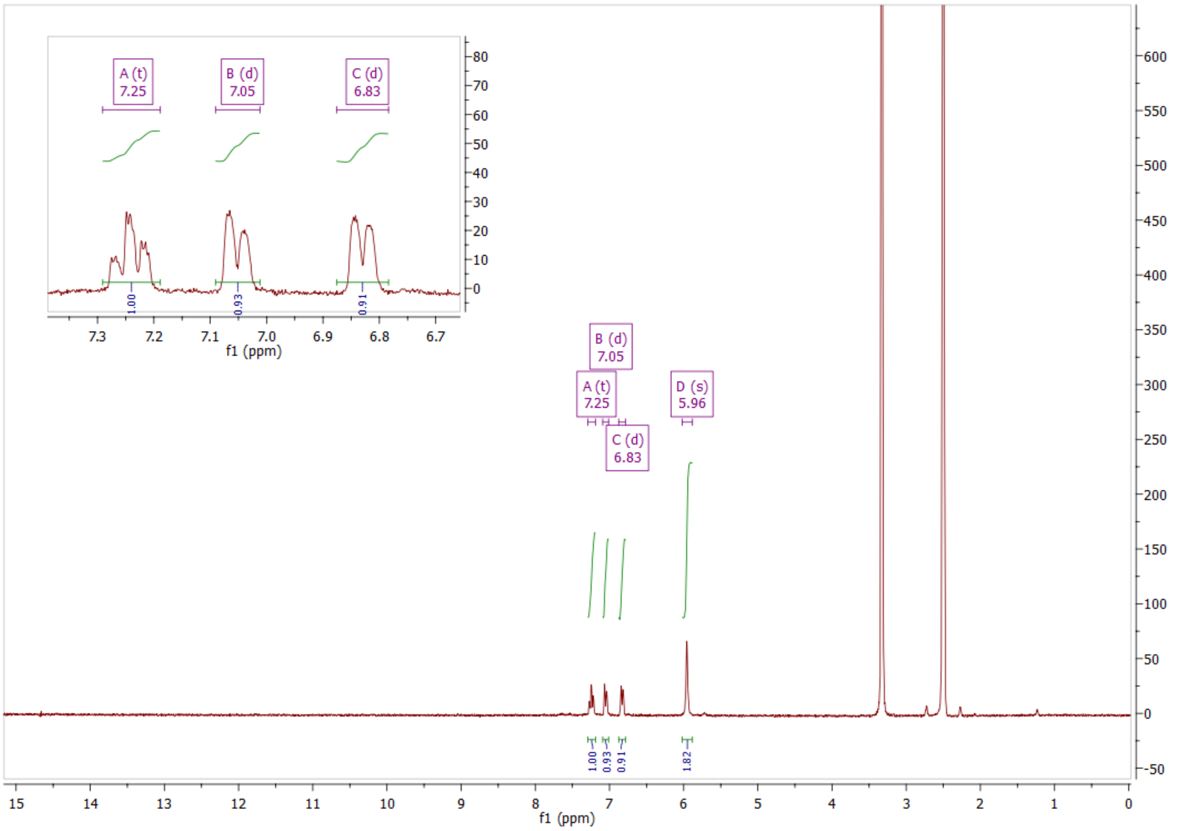

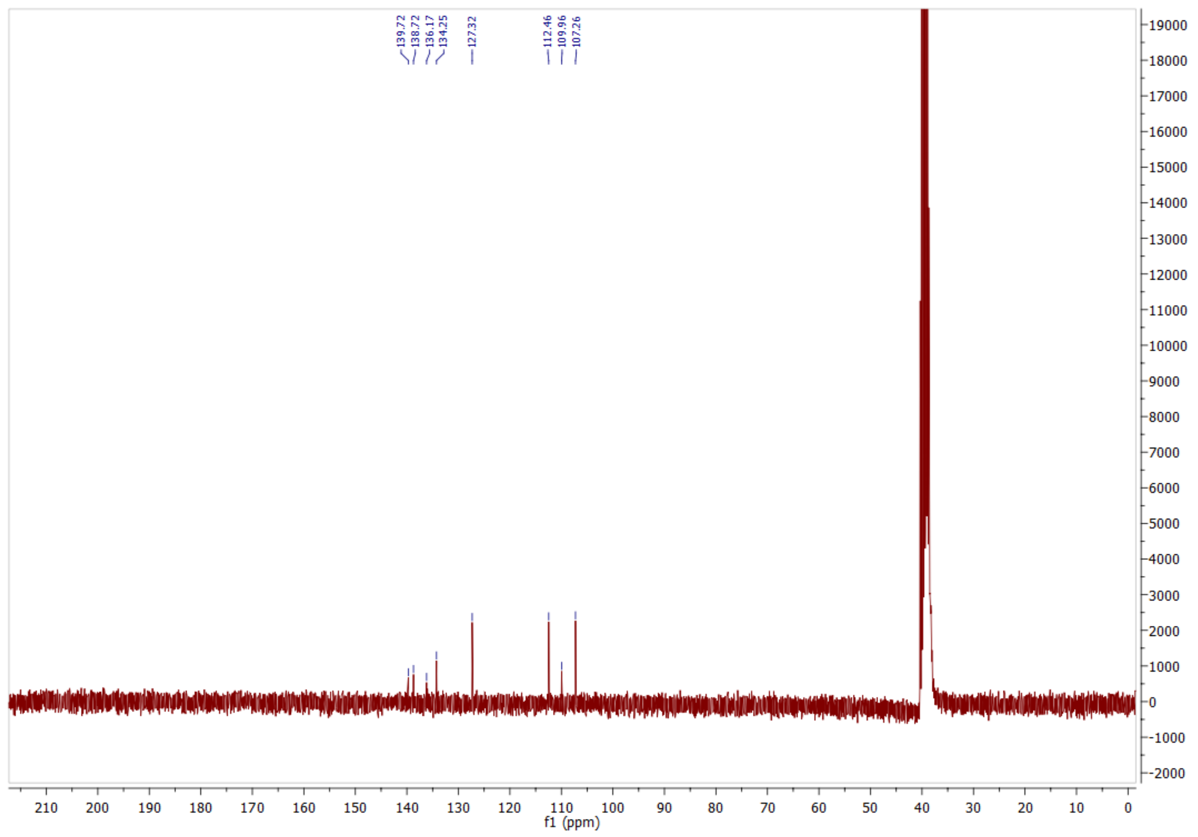

***N*-(2-cyano-1,3-benzoxazol-4-yl)benzamide (24)**

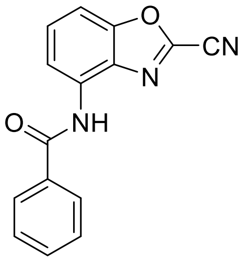

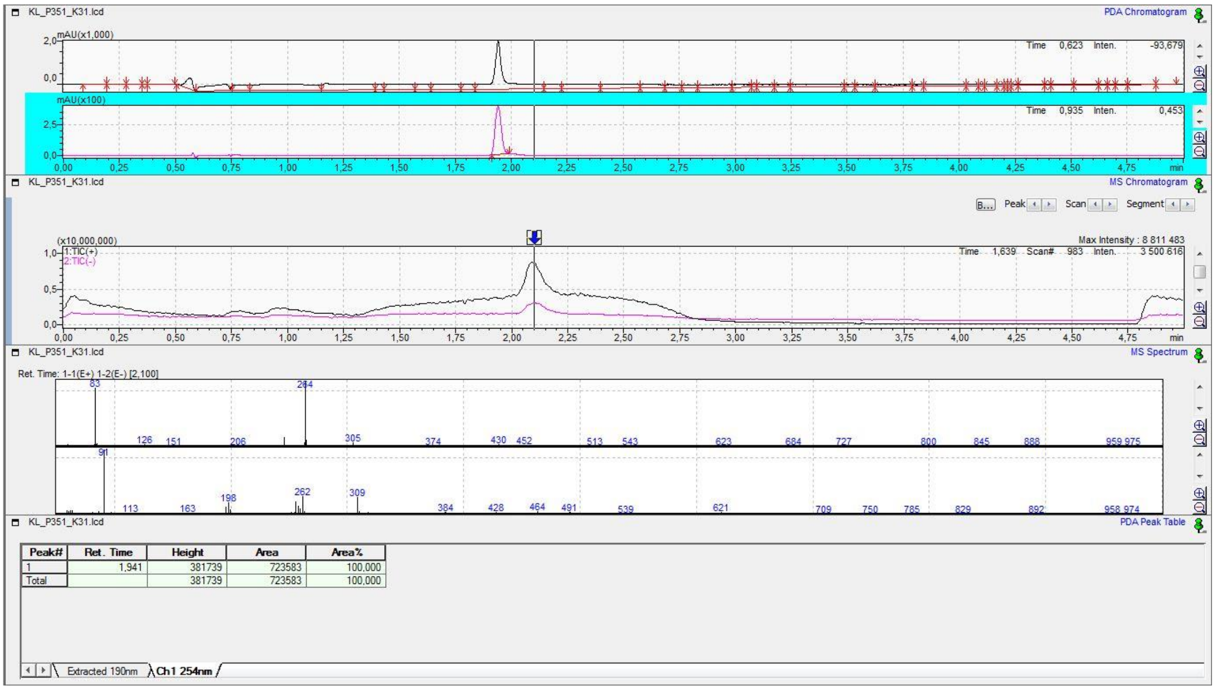
KL_P351_K31.lcd
PDA Chromatogram
MS Chromatogram
MS Spectrum
PDA Peak Table
Max Intensity : 8 811 483
Ret. Time: 1-1(E+) 1-2(E-) [2,100]
Peak# | Ret. Time | Height | Area | Area%
1 | 1,941 | 381739 | 723583 | 100,000
Total | | 381739 | 723583 | 100,000
Extracted 190nm
Ch1 254nm

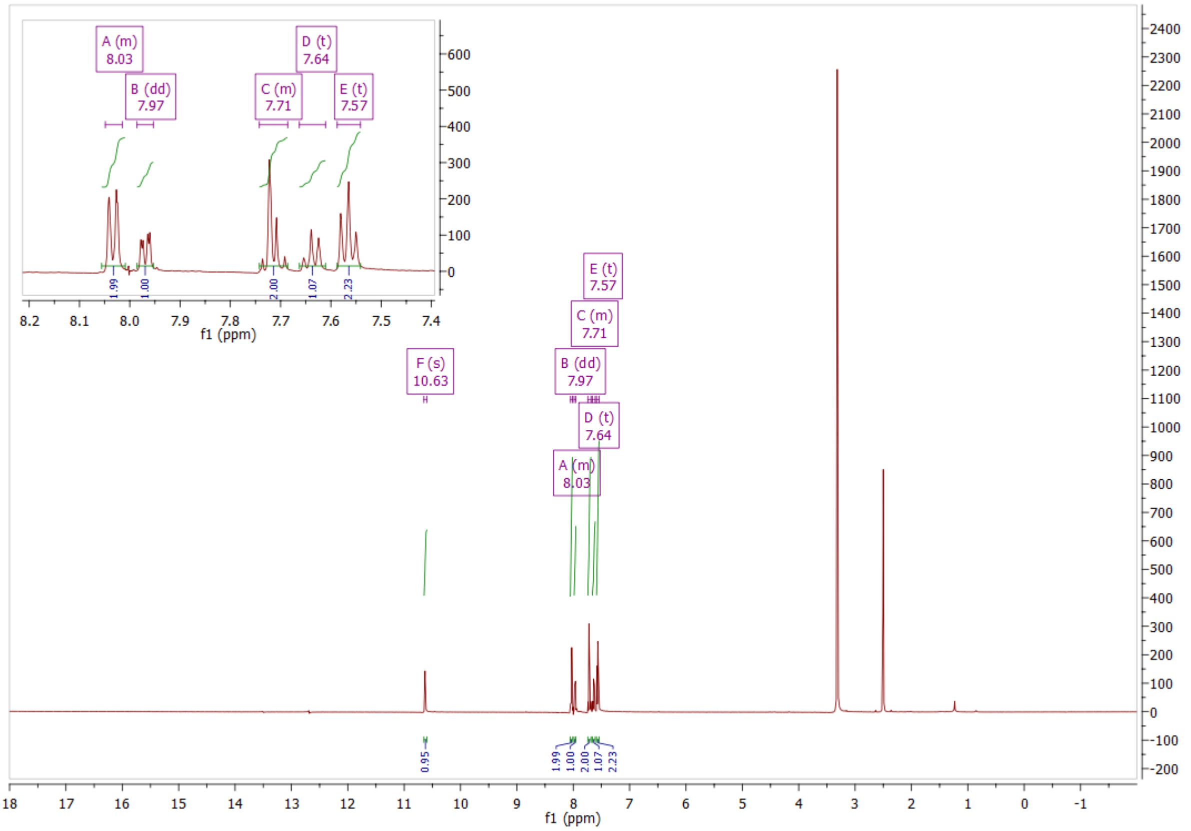
A (m) 8.03
B (dd) 7.97
C (m) 7.71
D (t) 7.64
E (t) 7.57
F (s) 10.63
f1 (ppm)

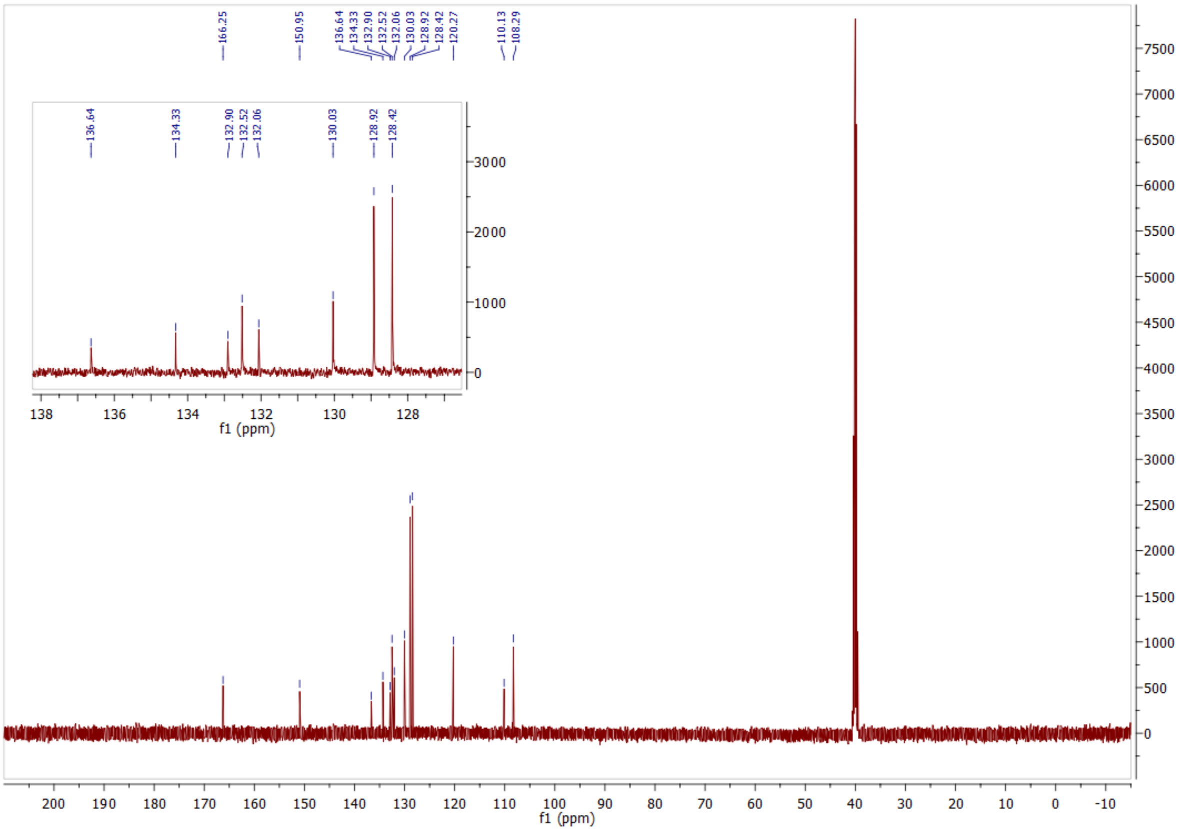

N-(2-cyano-1,3-benzoxazol-5-yl)benzamide (25)

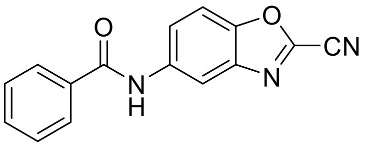

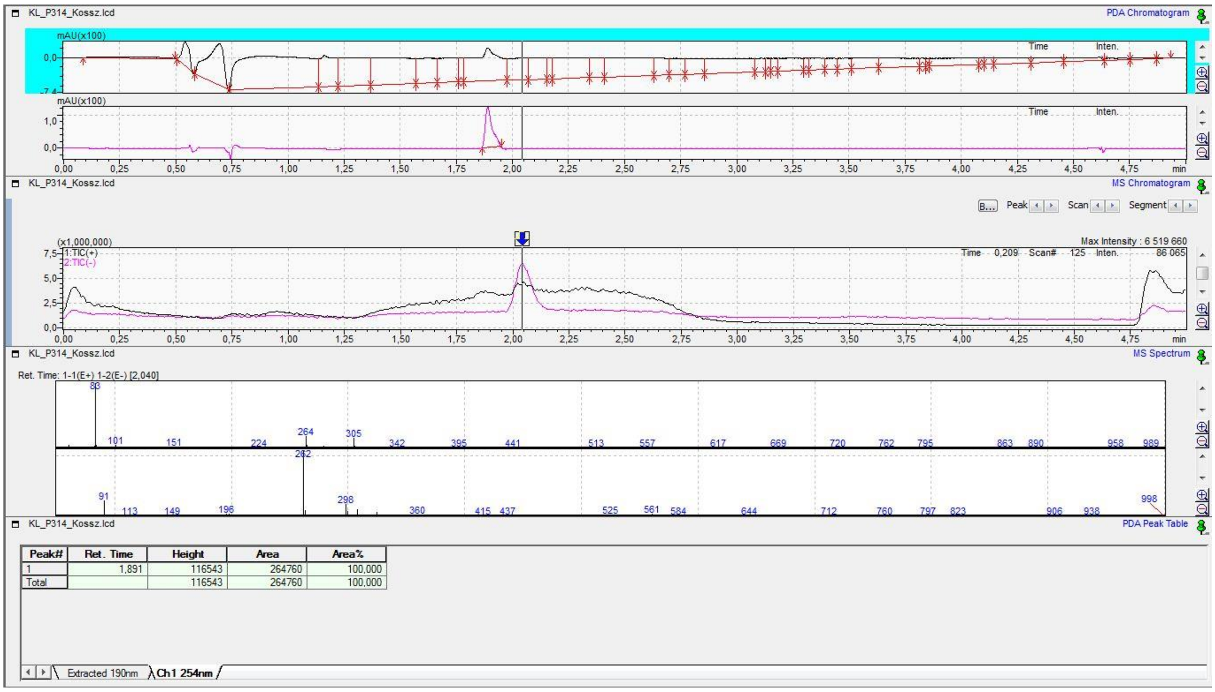

Peak#	Ret. Time	Height	Area	Area%
1	1,891	116543	264760	100,000
Total		116543	264760	100,000

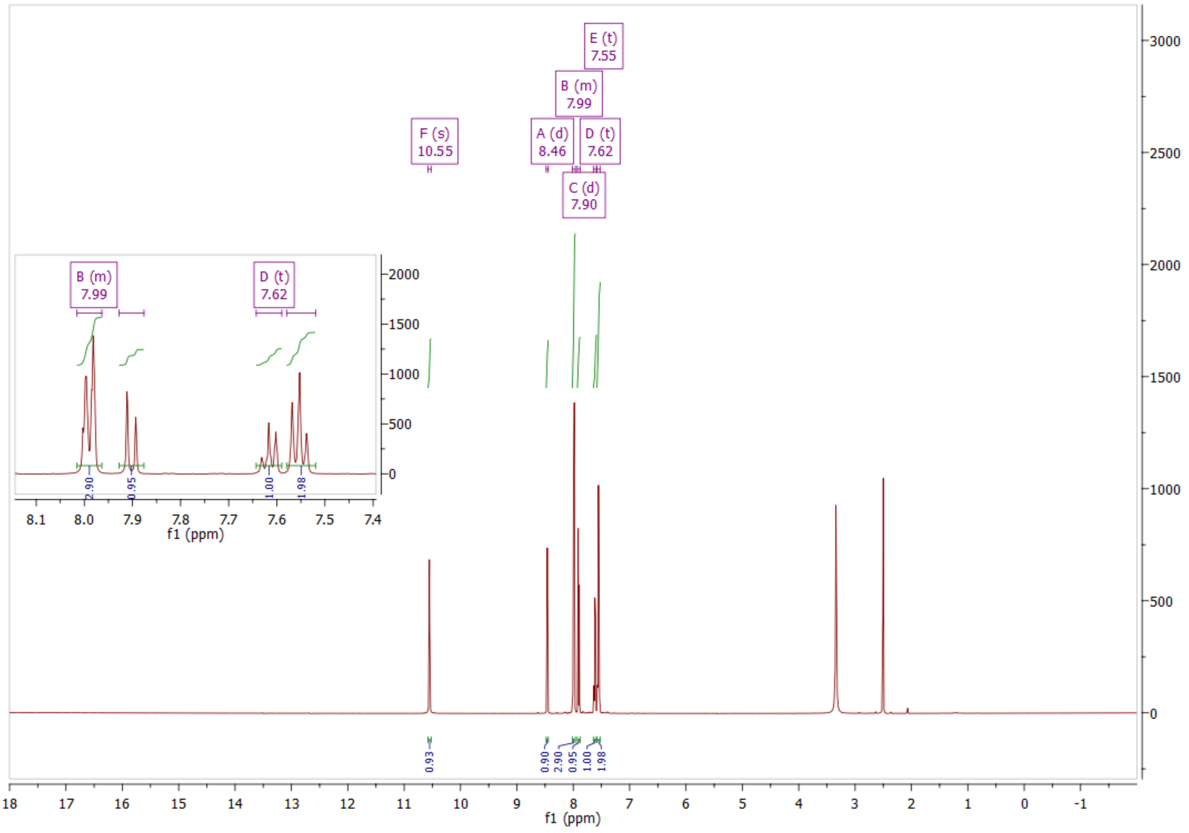
F (s)
10.55
A (d)
8.46
B (m)
7.99
E (t)
7.55
D (t)
7.62
C (d)
7.90
0.93
0.90
2.90
0.95
1.00
1.98
f1 (ppm)

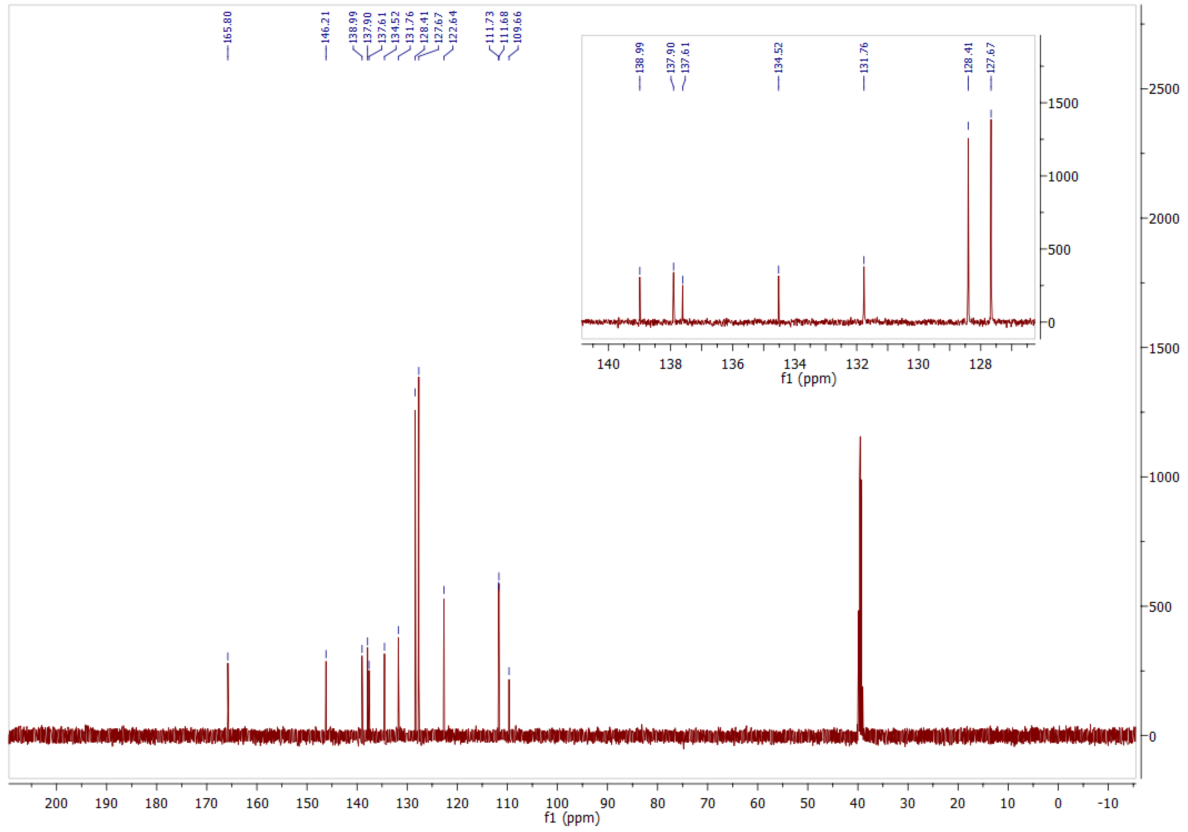
165.80
146.21
138.99
137.90
137.61
134.52
131.76
128.41
127.67
122.64
111.73
111.68
109.66
f1 (ppm)

N-(2-cyano-1,3-benzoxazol-6-yl)benzamide (26)

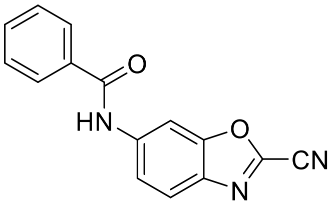

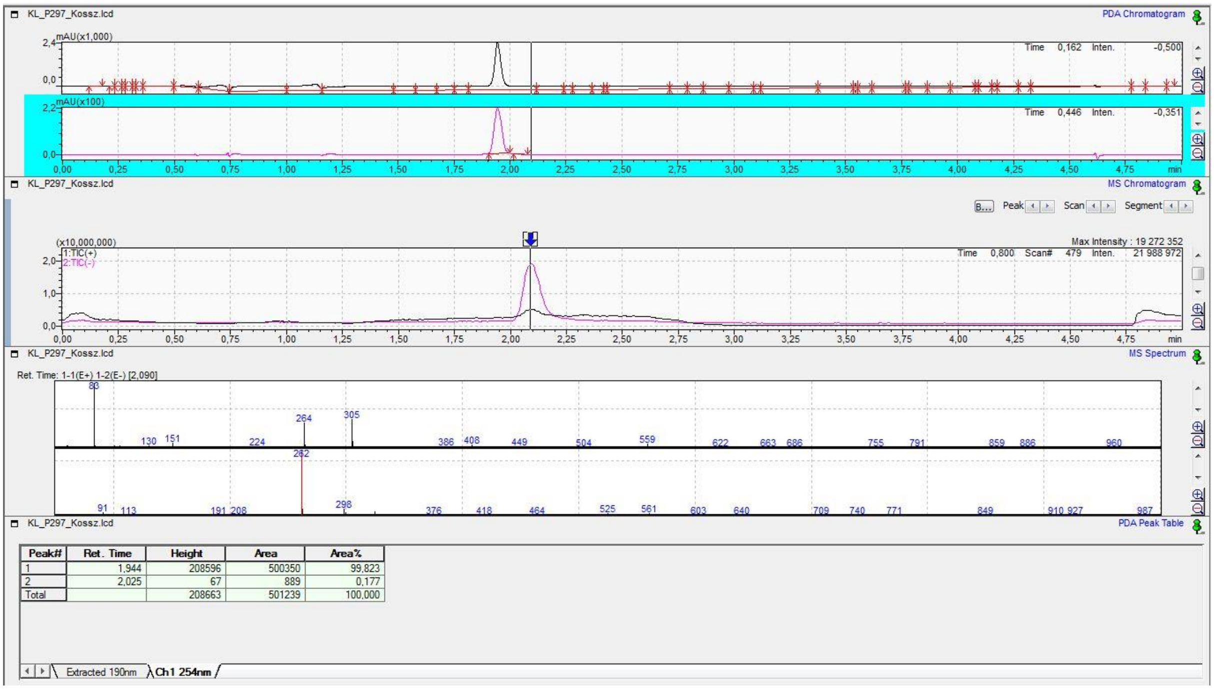

Peak#	Ret. Time	Height	Area	Area%
1	1,944	208596	500350	99,823
2	2,025	67	889	0,177
Total		208663	501239	100,000

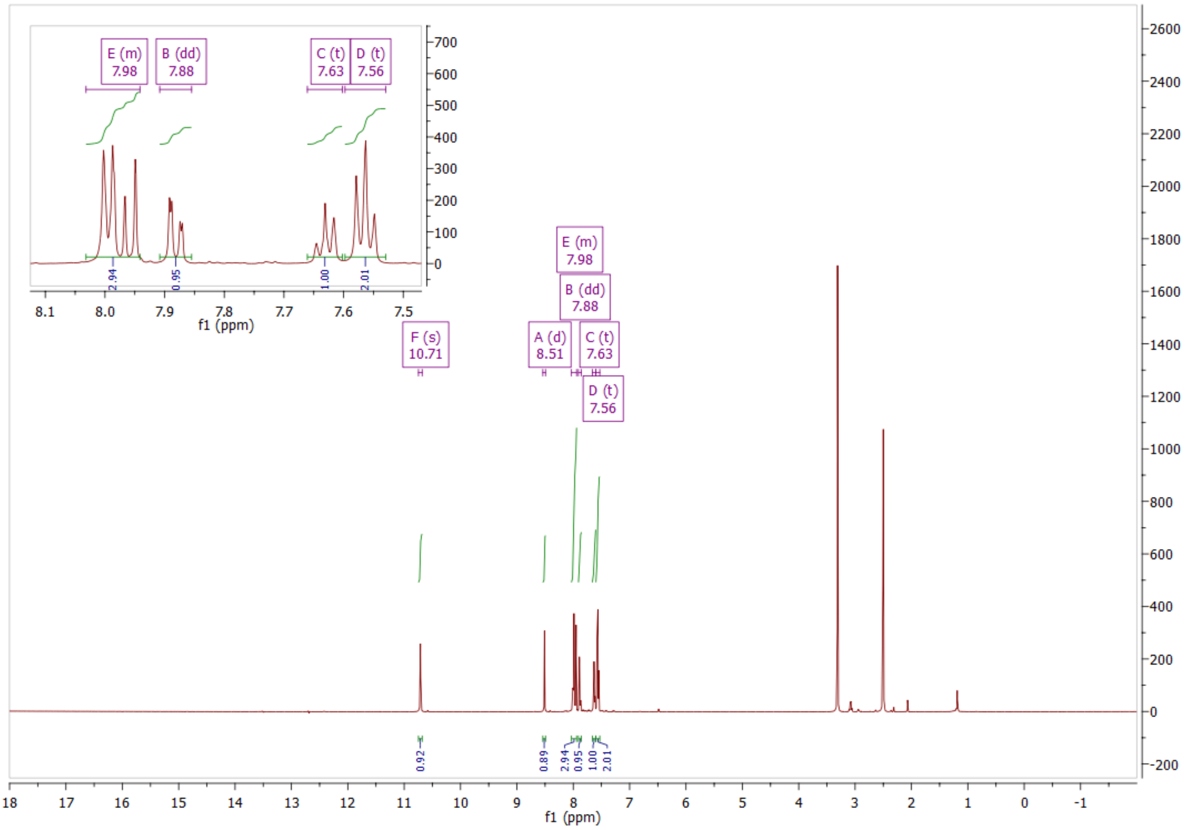

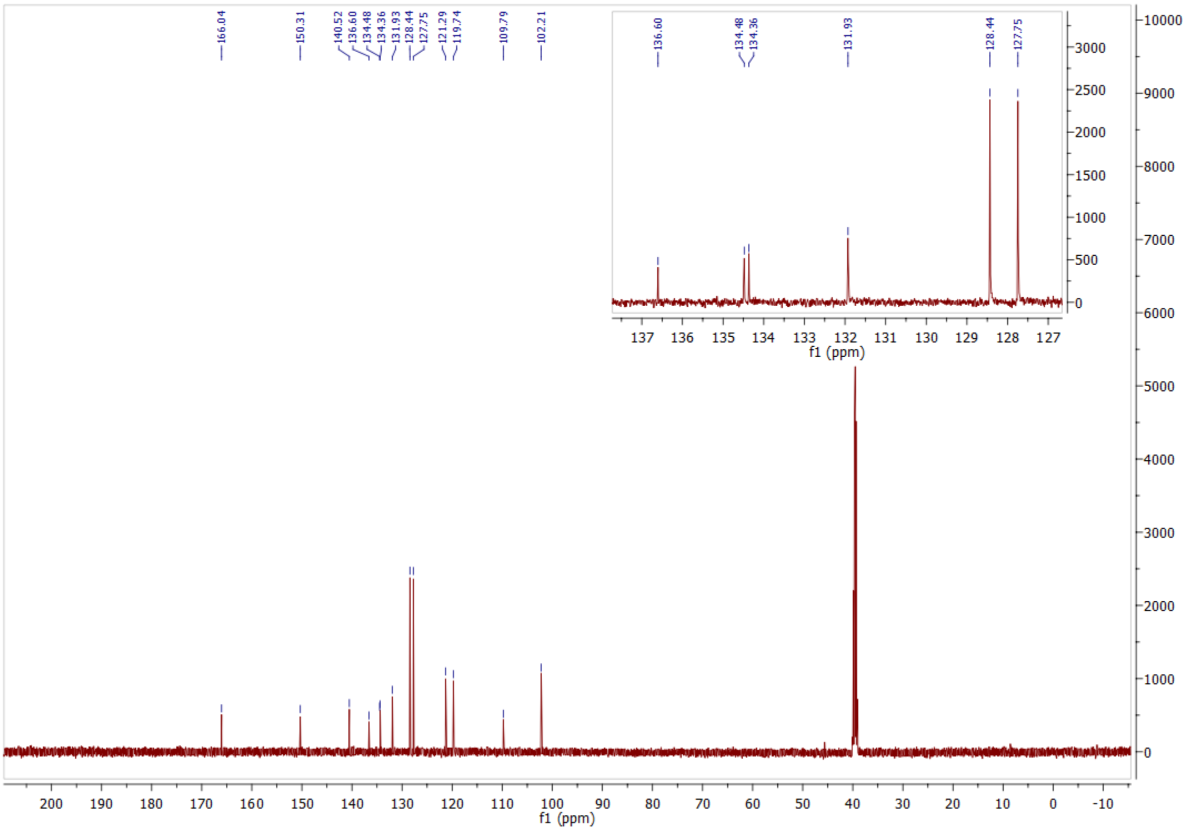

N-(2-cyano-1,3-benzoxazol-7-yl)benzamide (27)

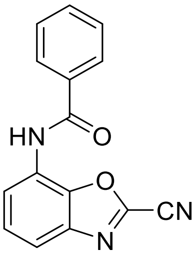

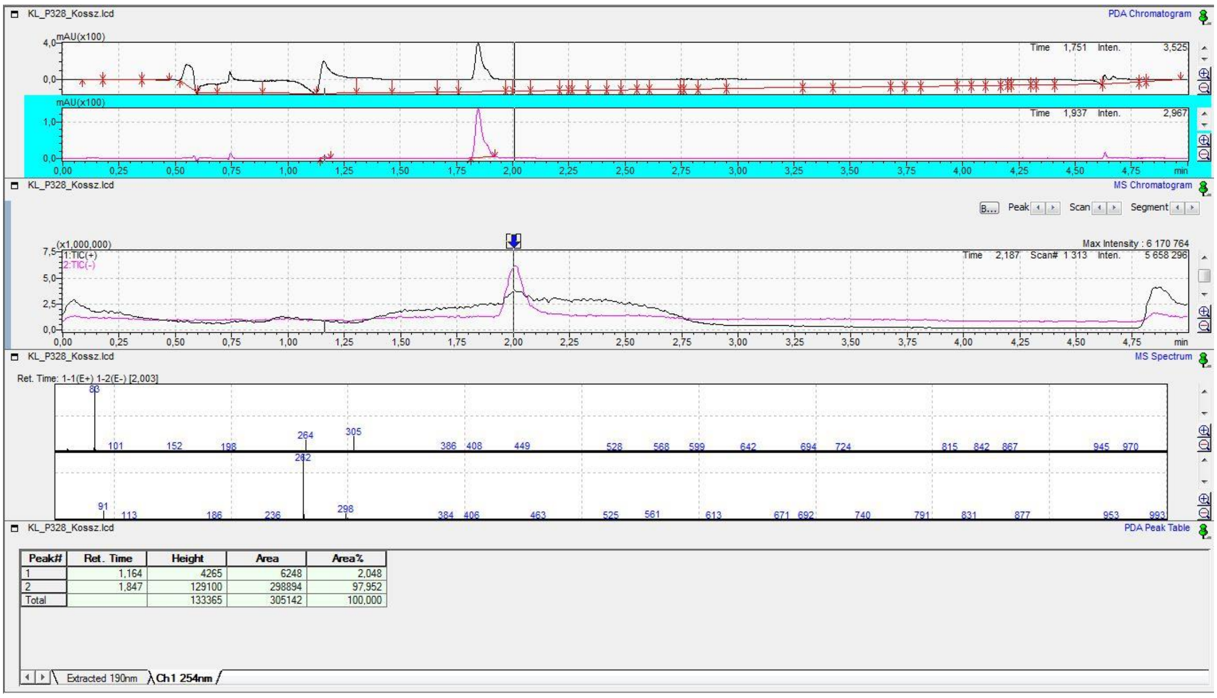

Peak#	Ret. Time	Height	Area	Area%
1	1,164	4265	6248	2,048
2	1,847	129100	298894	97,952
Total		133365	305142	100,000

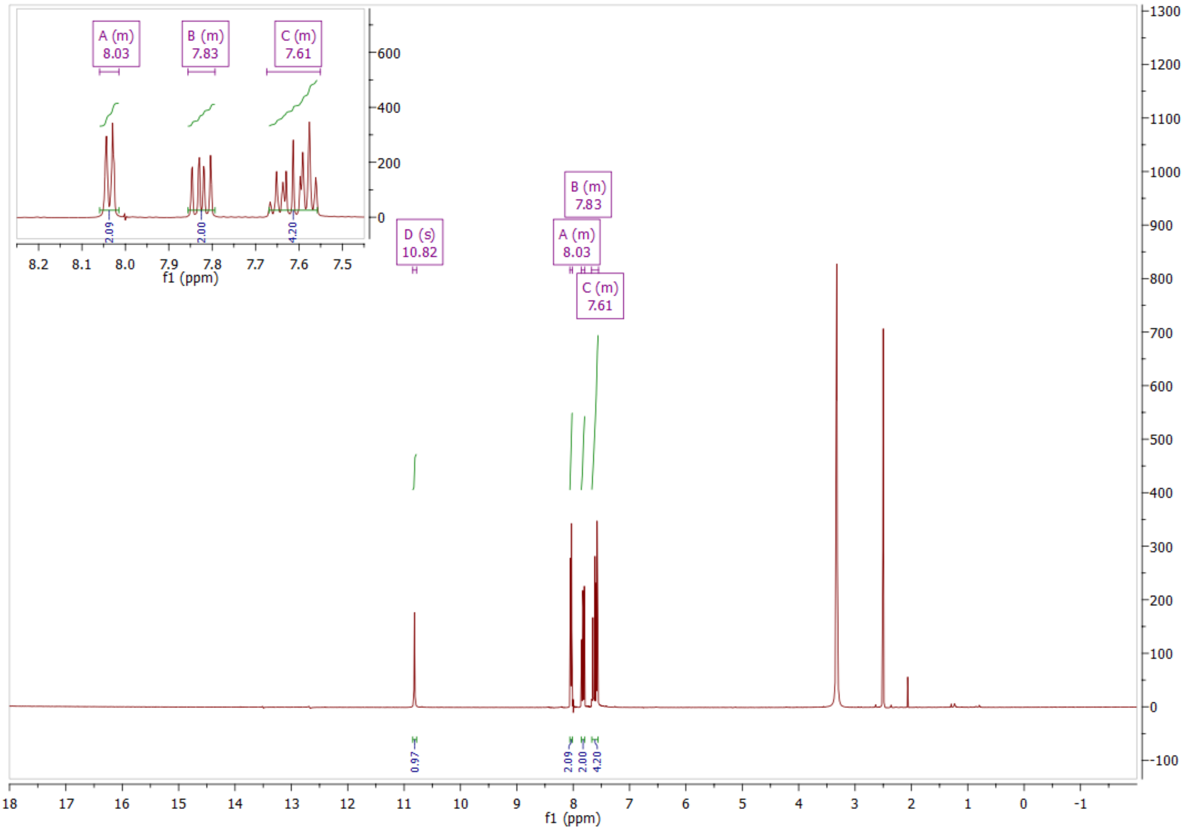

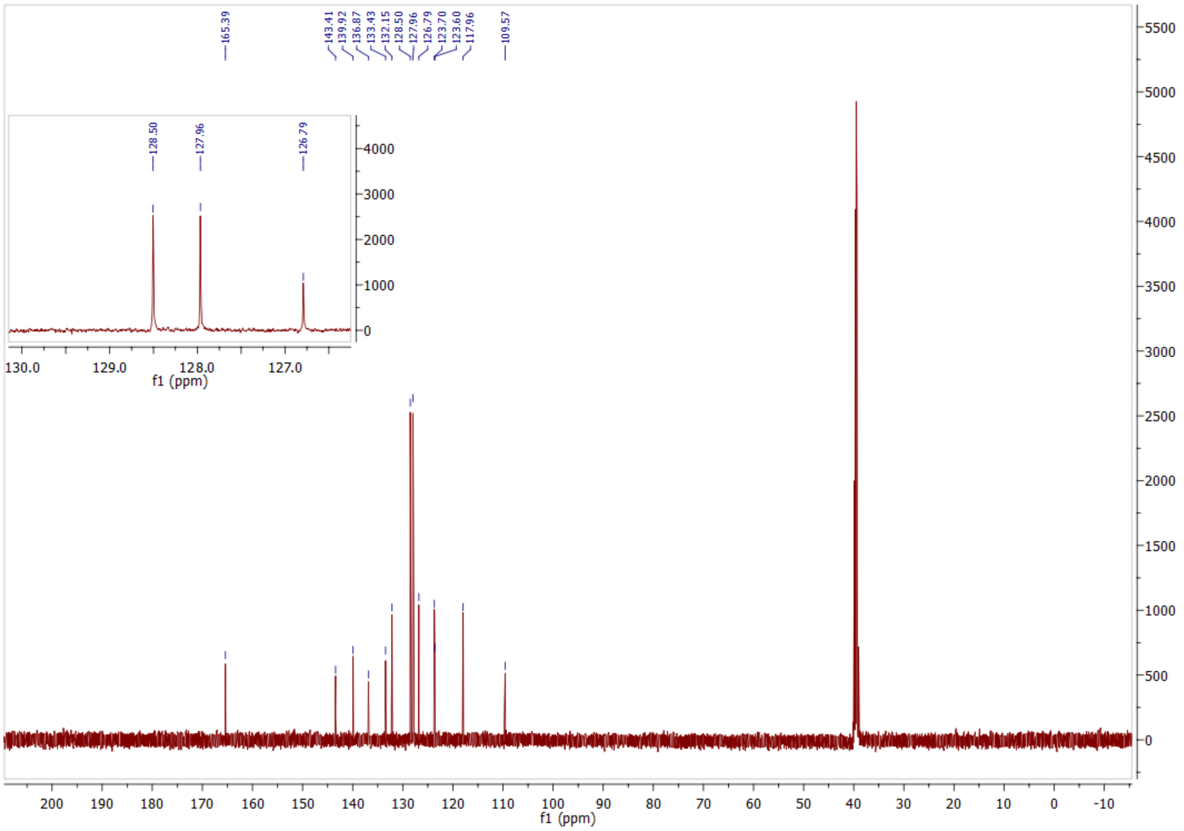

Methyl 2-(2-bromo-1,3-thiazol-4-yl)acetate (XXIV)

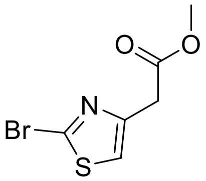

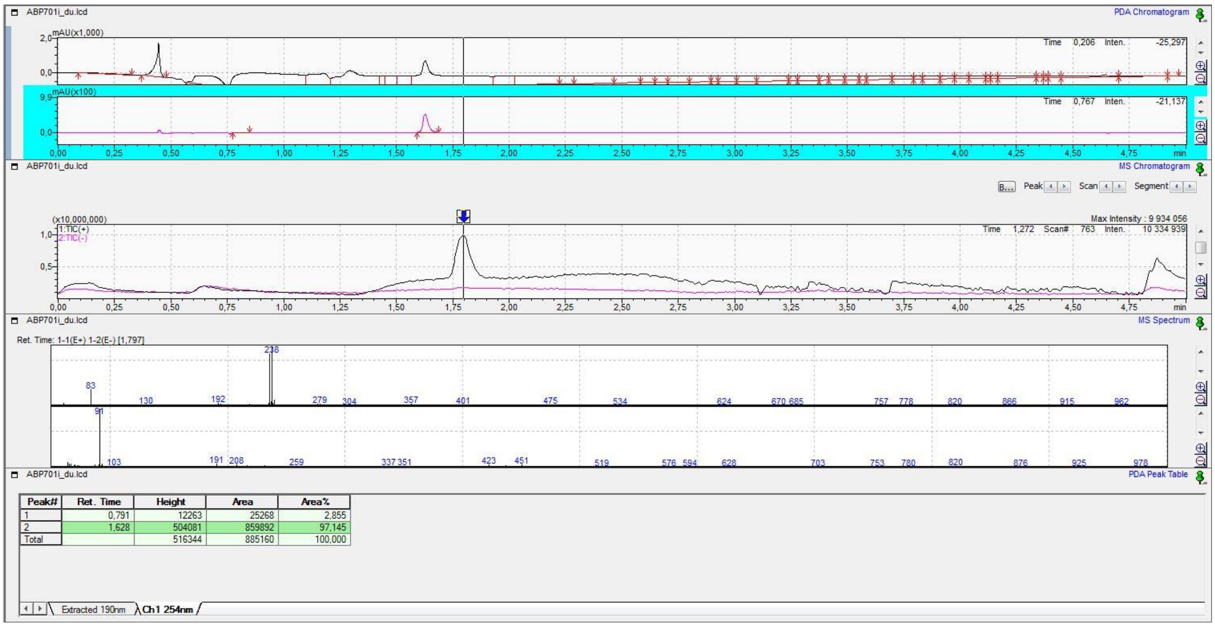

Peak#	Ret. Time	Height	Area	Area%
1	0,791	12263	25268	2,855
2	1,628	504081	859892	97,145
Total		516344	885160	100,000

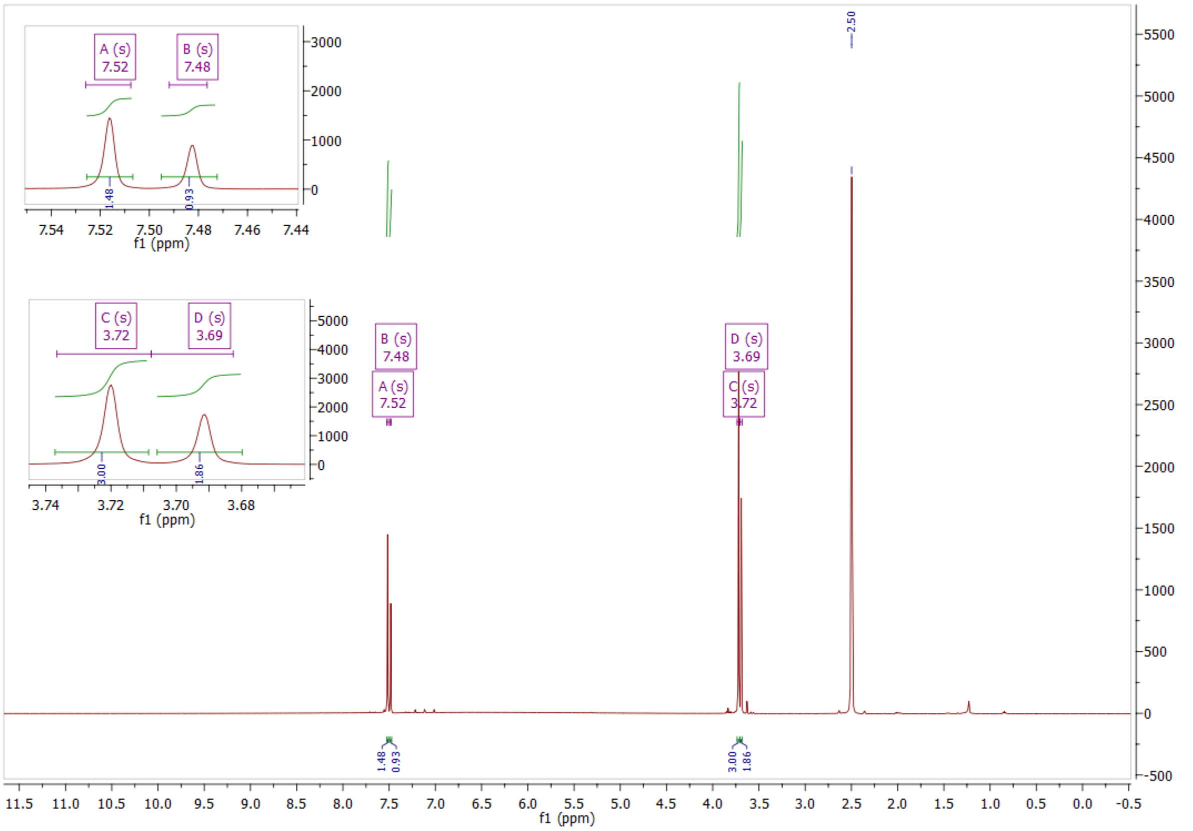
2.50
A (s)
7.52
B (s)
7.48
1.48
0.93
C (s)
3.72
D (s)
3.69
3.00
1.86
f1 (ppm)

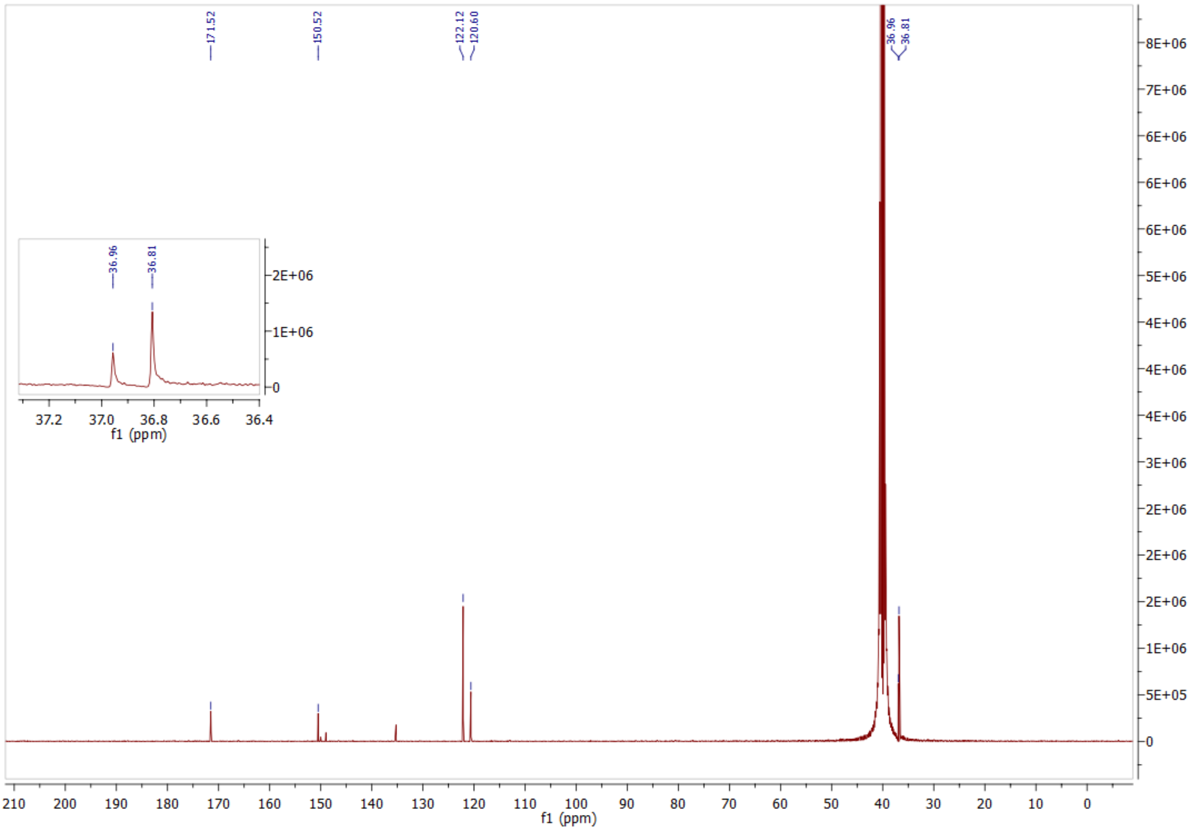
171.52
150.52
122.12
120.60
36.96
36.81
f1 (ppm)

Methyl 2-(2-ethenyl-1,3-thiazol-4-yl)acetate (XXV)

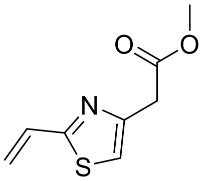

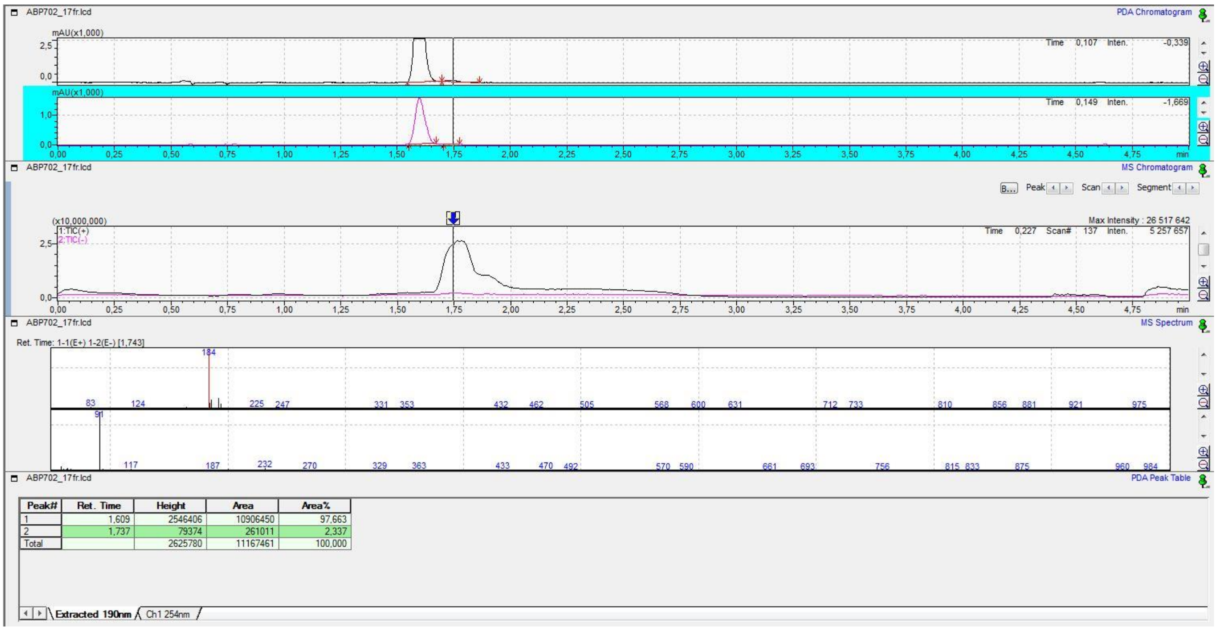

Peak#	Ret. Time	Height	Area	Area%
1	1,609	2546406	10906450	97,663
2	1,737	79374	261011	2,337
Total		2625780	11167461	100,000

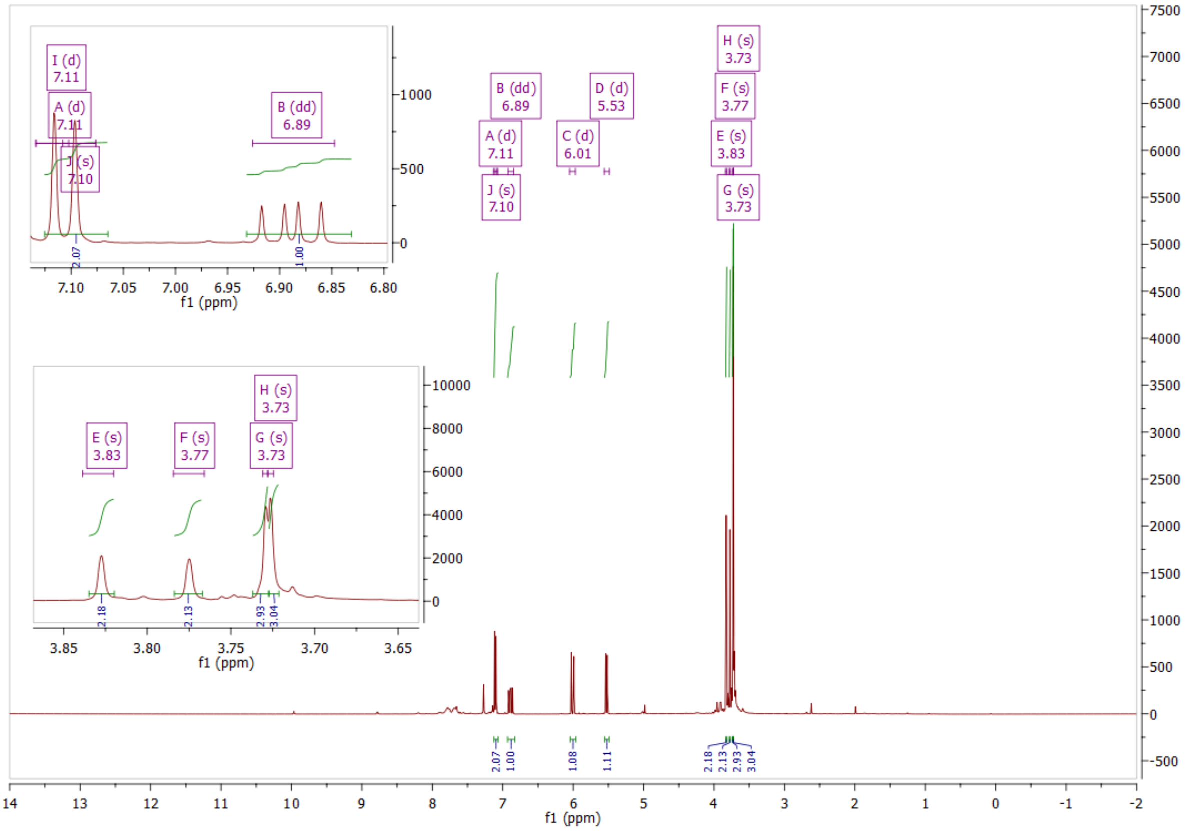

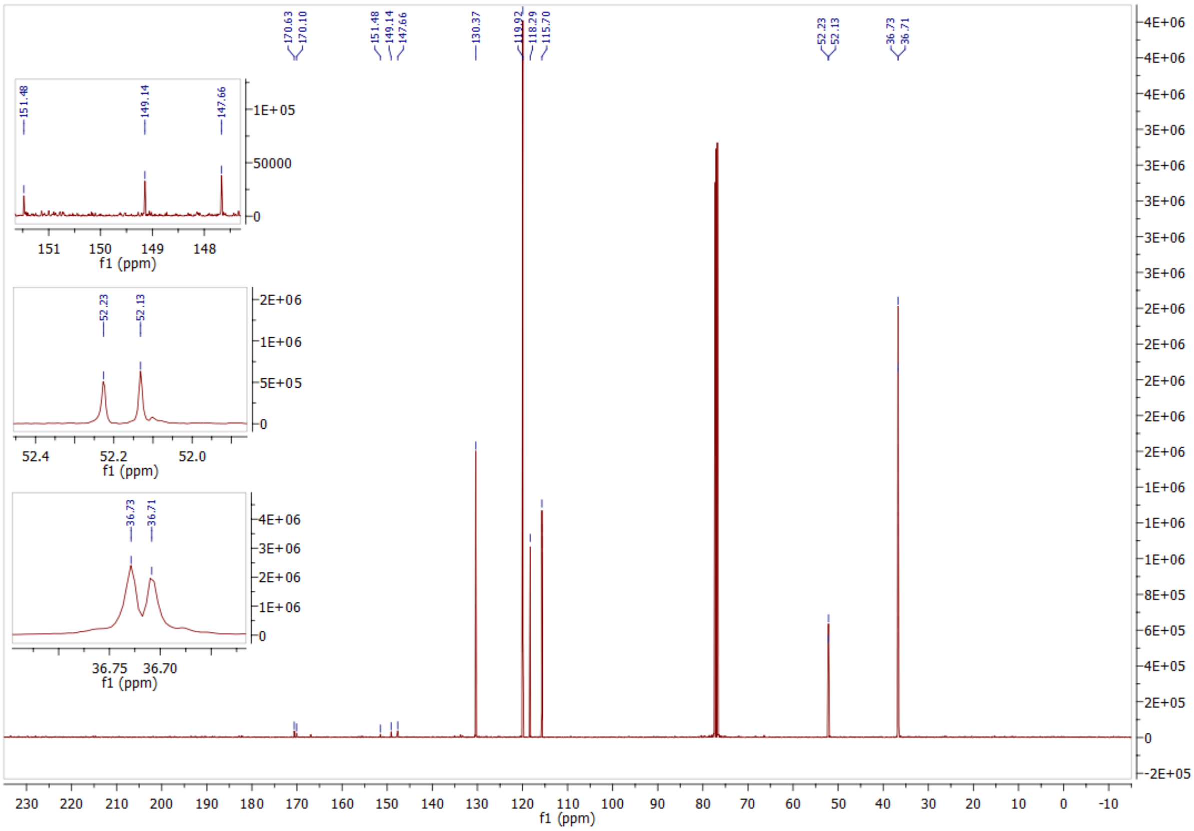

2-(2-Ethenyl-1,3-thiazol-4-yl)acetic acid (XXVI)

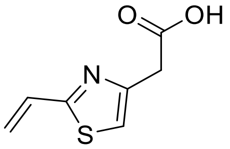

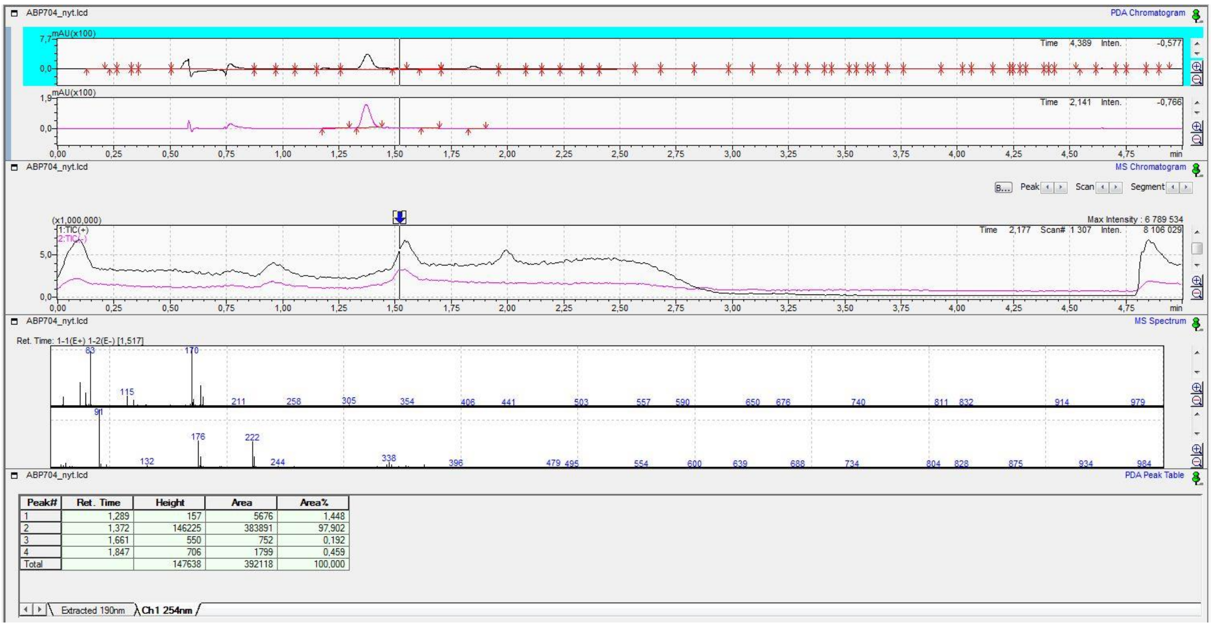

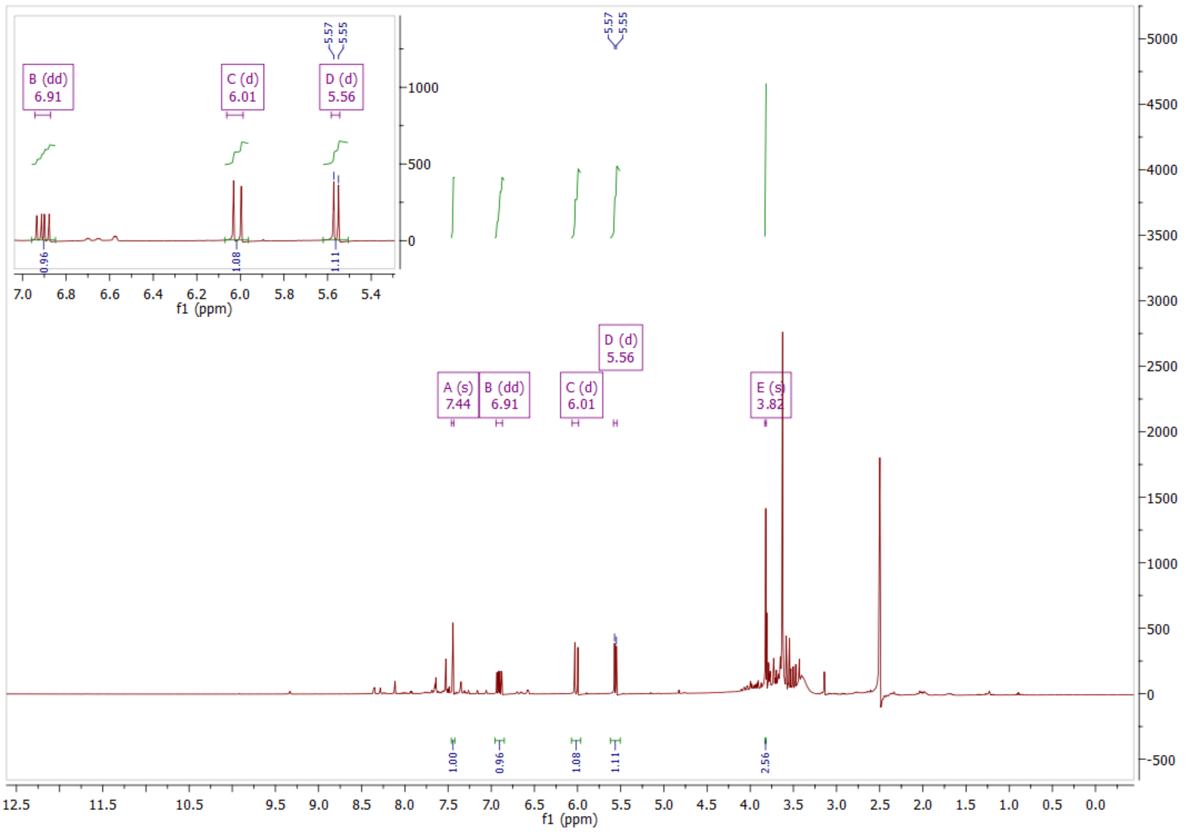

2-(2-Ethenyl-1,3-thiazol-4-yl)-*N*-[(1*R*)-3-methyl-1-[(1*S*,2*S*,6*R*,8*S*)-2,9,9-trimethyl-3,5-dioxa-4-boratricyclo[6.1.1.0^{2,6}]decan-4-yl]butyl]acetamide (28)

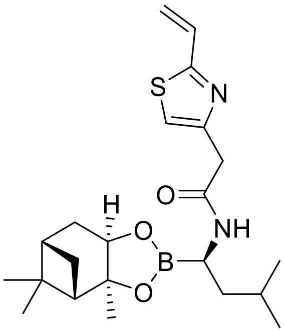

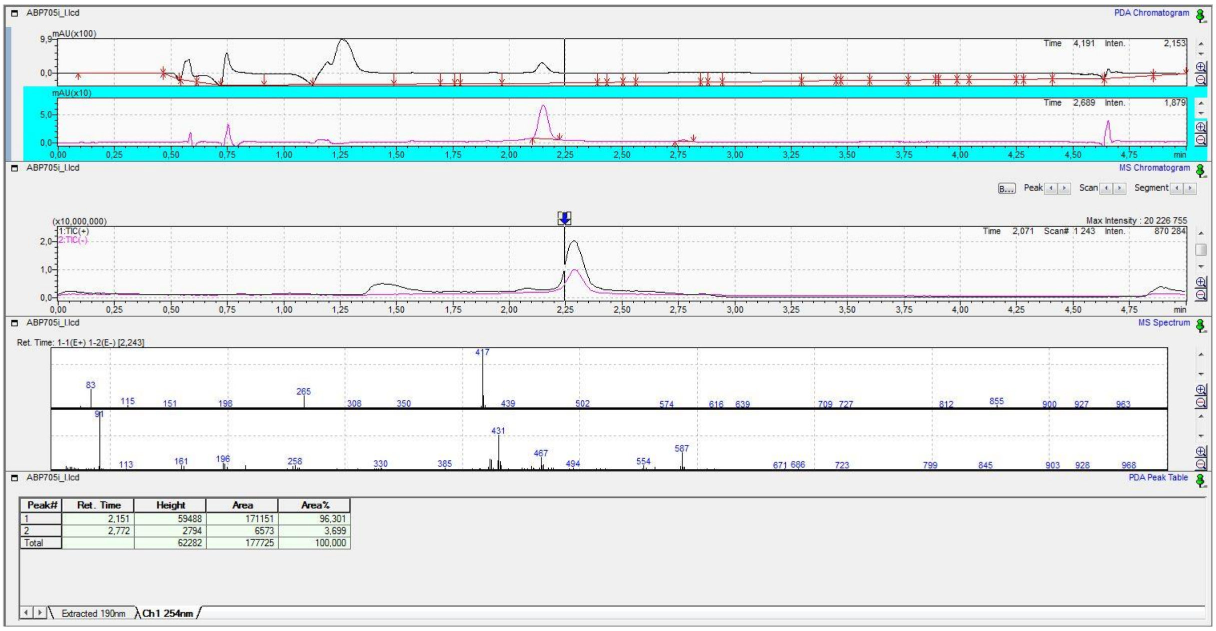

Peak#	Ret. Time	Height	Area	Area%
1	2,151	59488	171151	96,301
2	2,772	2794	6573	3,699
Total		62282	177725	100,000

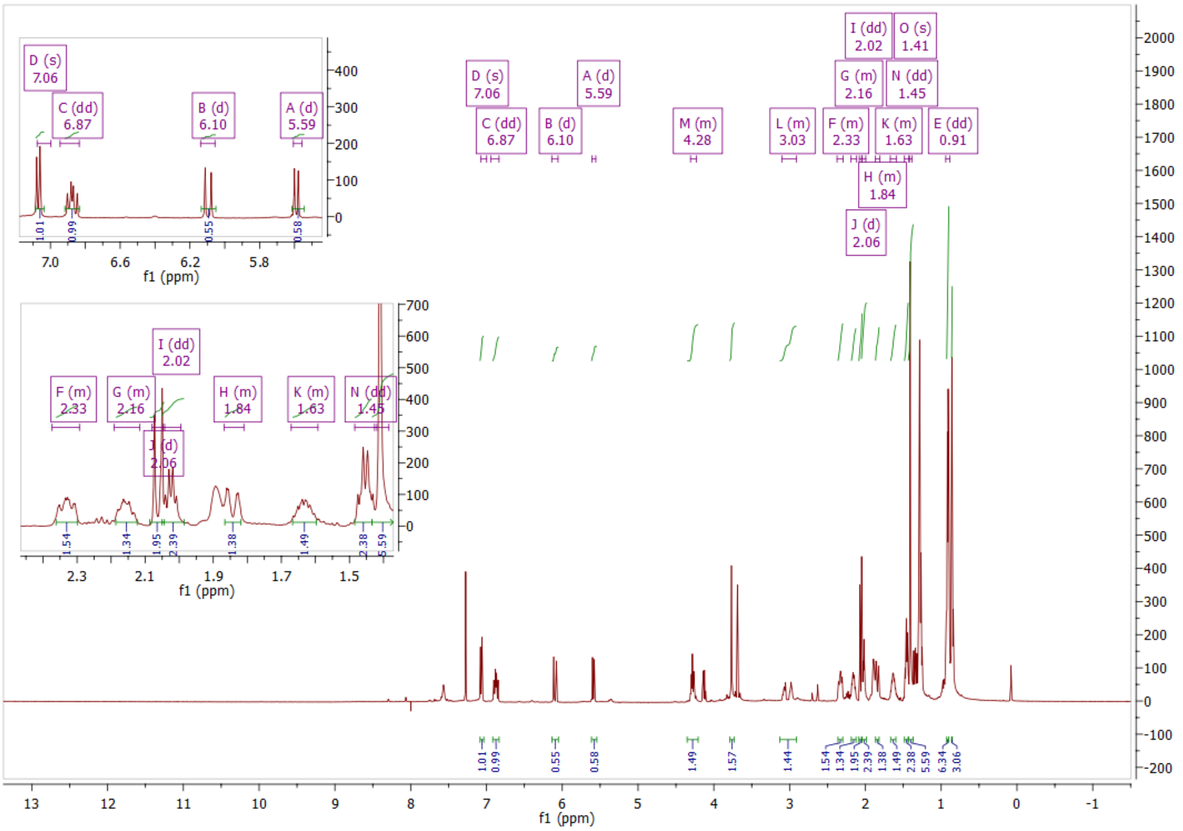

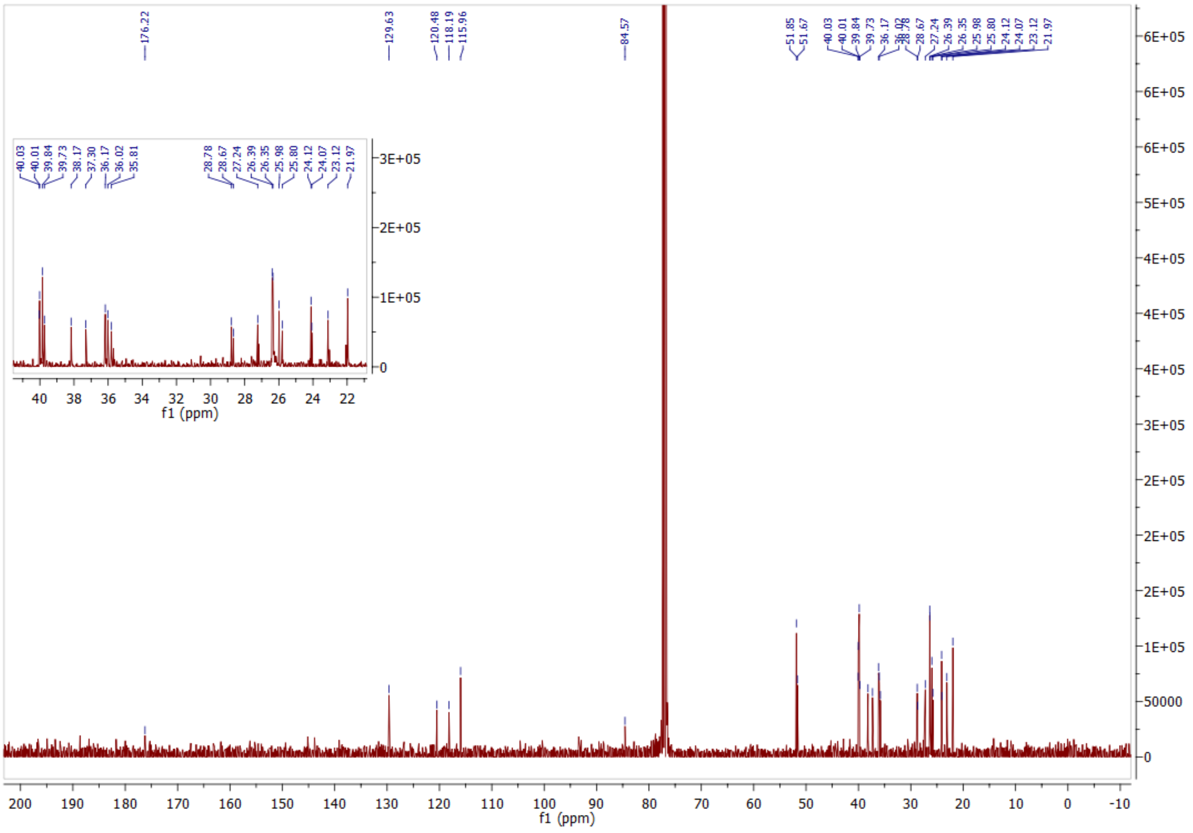

2-(2-Cyano-1*H*-1,3-benzodiazol-1-yl)acetic acid (XXX)

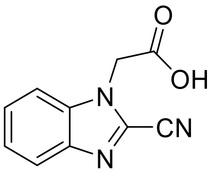

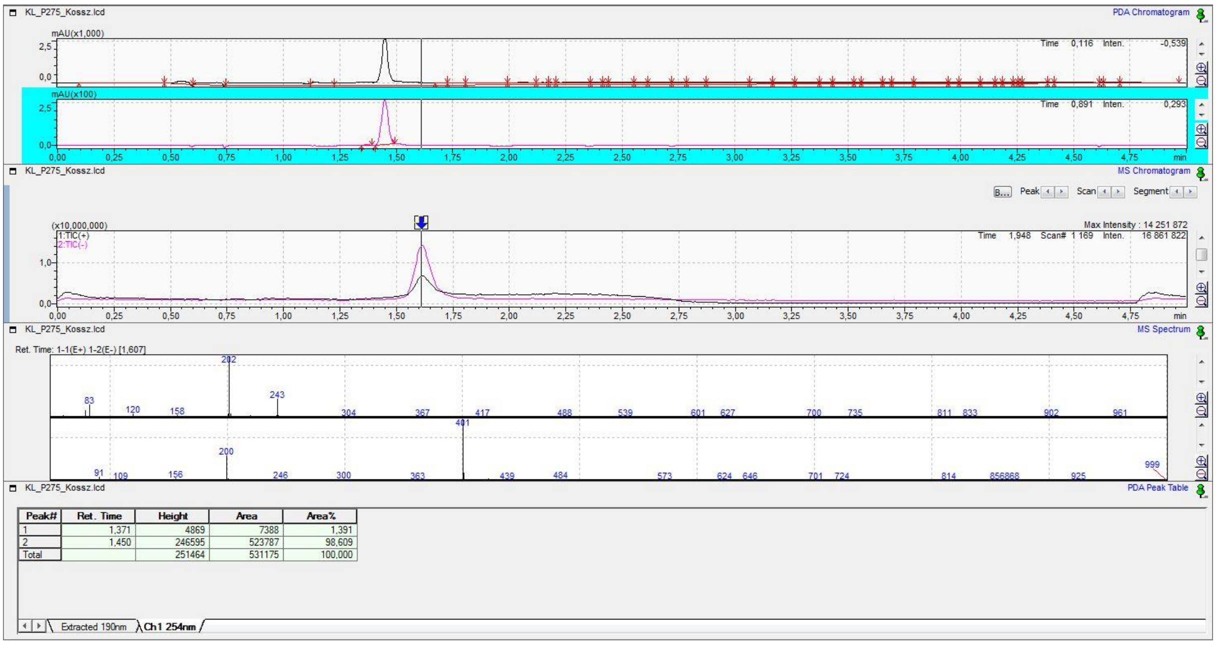

Peak#	Ret. Time	Height	Area	Area%
1	1,371	4869	7388	1,391
2	1,450	246595	523787	98,609
Total		251464	531175	100,000

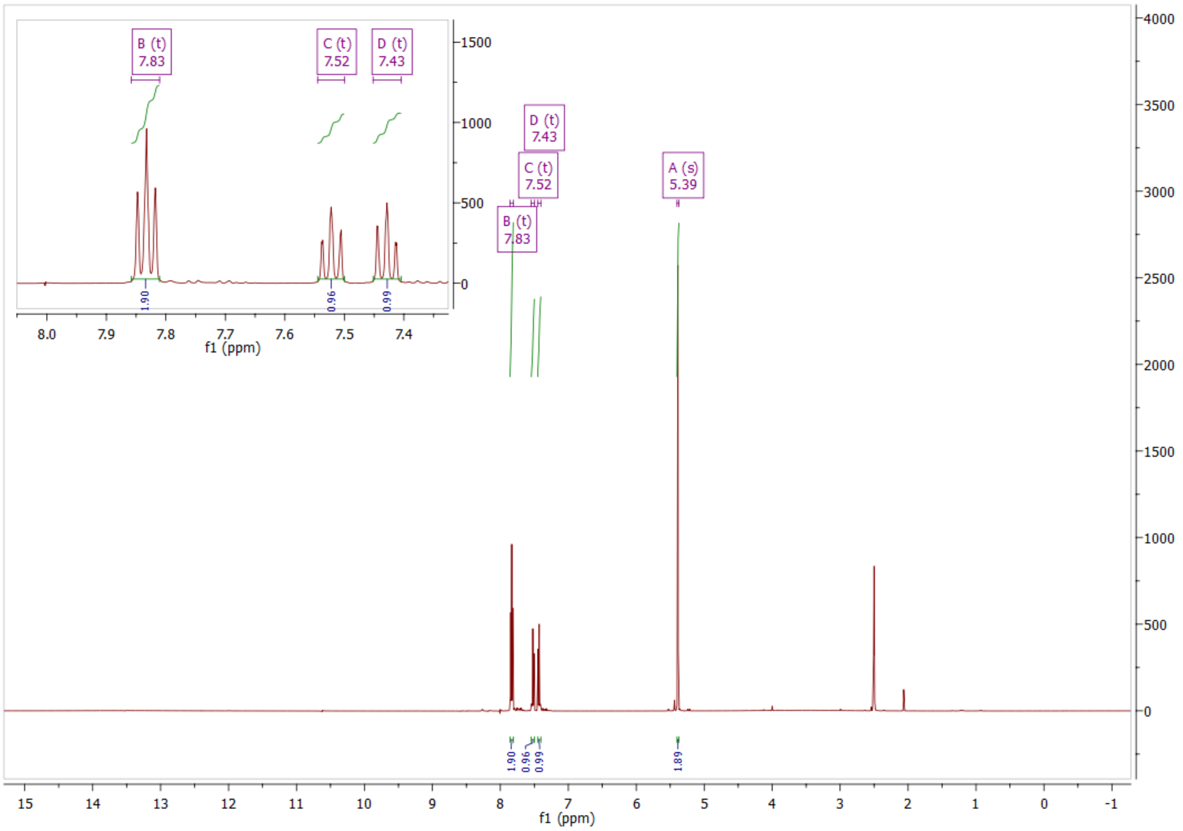
B (t)
7.83
C (t)
7.52
D (t)
7.43
A (s)
5.39
1.90
0.96
0.99
1.89
f1 (ppm)

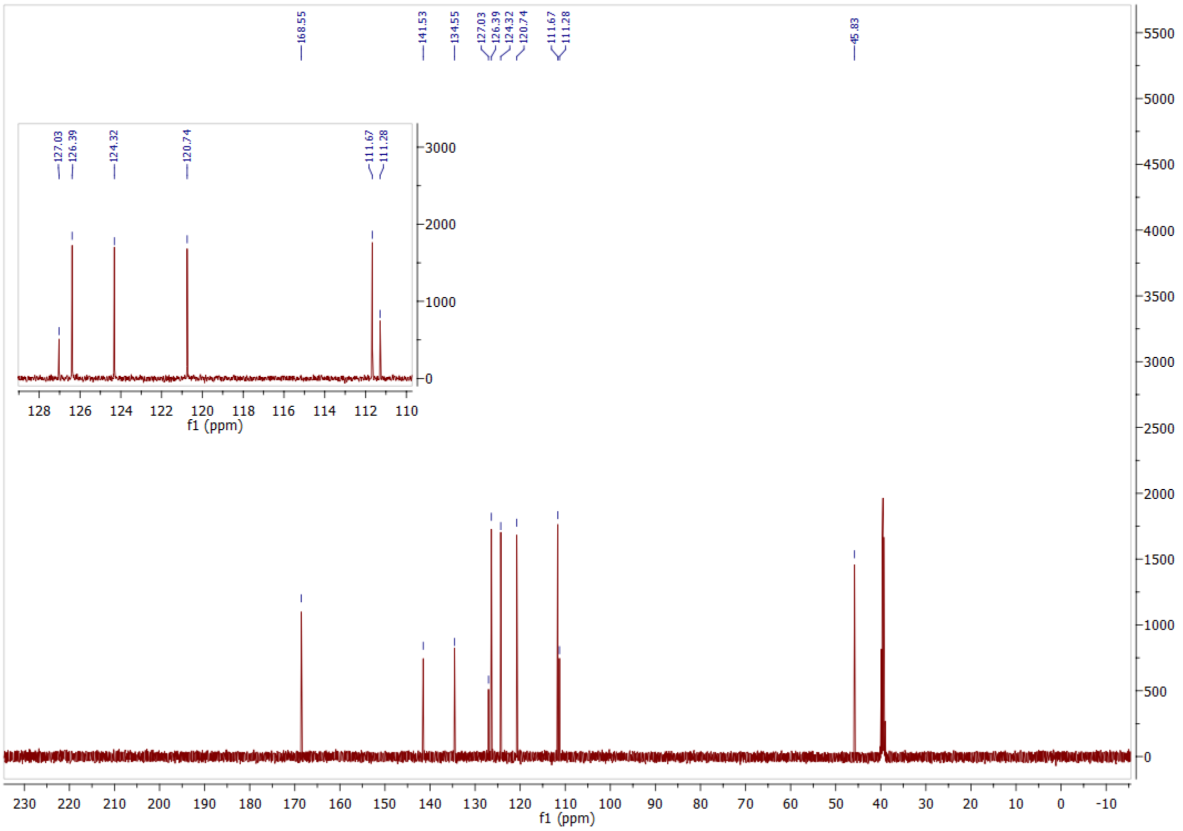
168.55
141.53
134.55
127.03
126.39
124.32
120.74
111.67
111.28
45.83
f1 (ppm)

2-(2-Cyano-1*H*-1,3-benzodiazol-1-yl)-*N*-[(1*R*)-3-methyl-1-[(1*S*,2*S*,6*R*,8*S*)-2,9,9-trimethyl-3,5-dioxa-4-boratricyclo[6.1.1.0^{2,6}]decan-4-yl]butyl]acetamide (29)

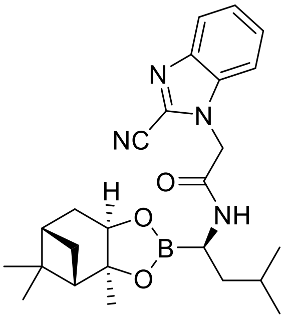

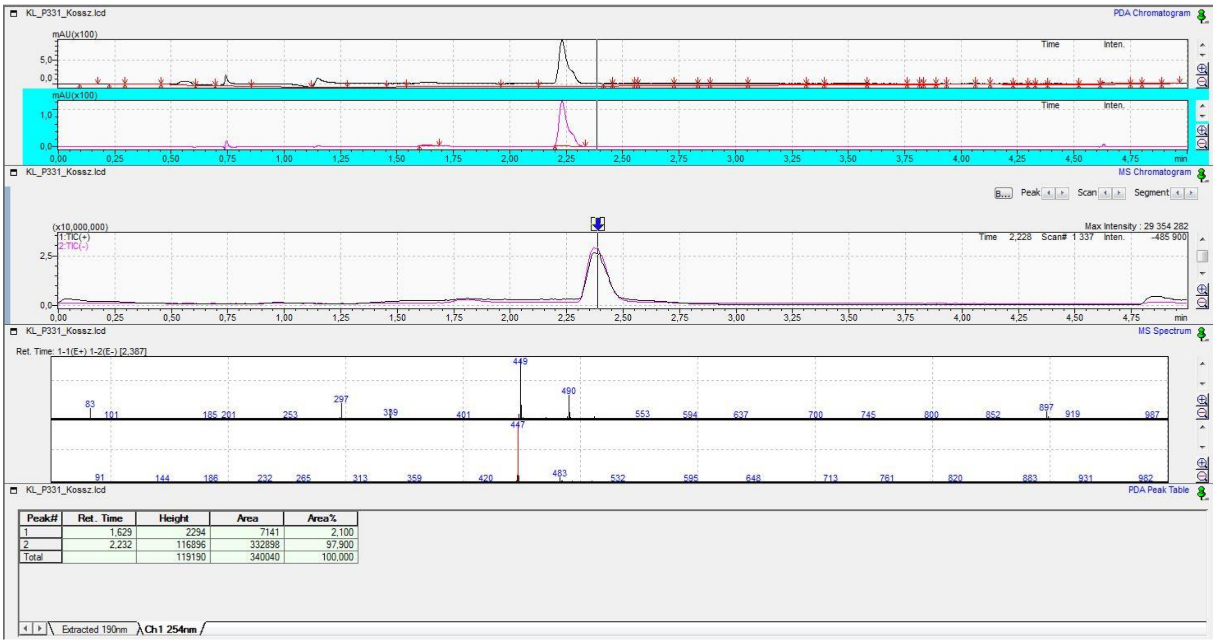

Peak#	Ret. Time	Height	Area	Area%
1	1,629	2294	7141	2,100
2	2,232	116896	332898	97,900
Total		119190	340040	100,000

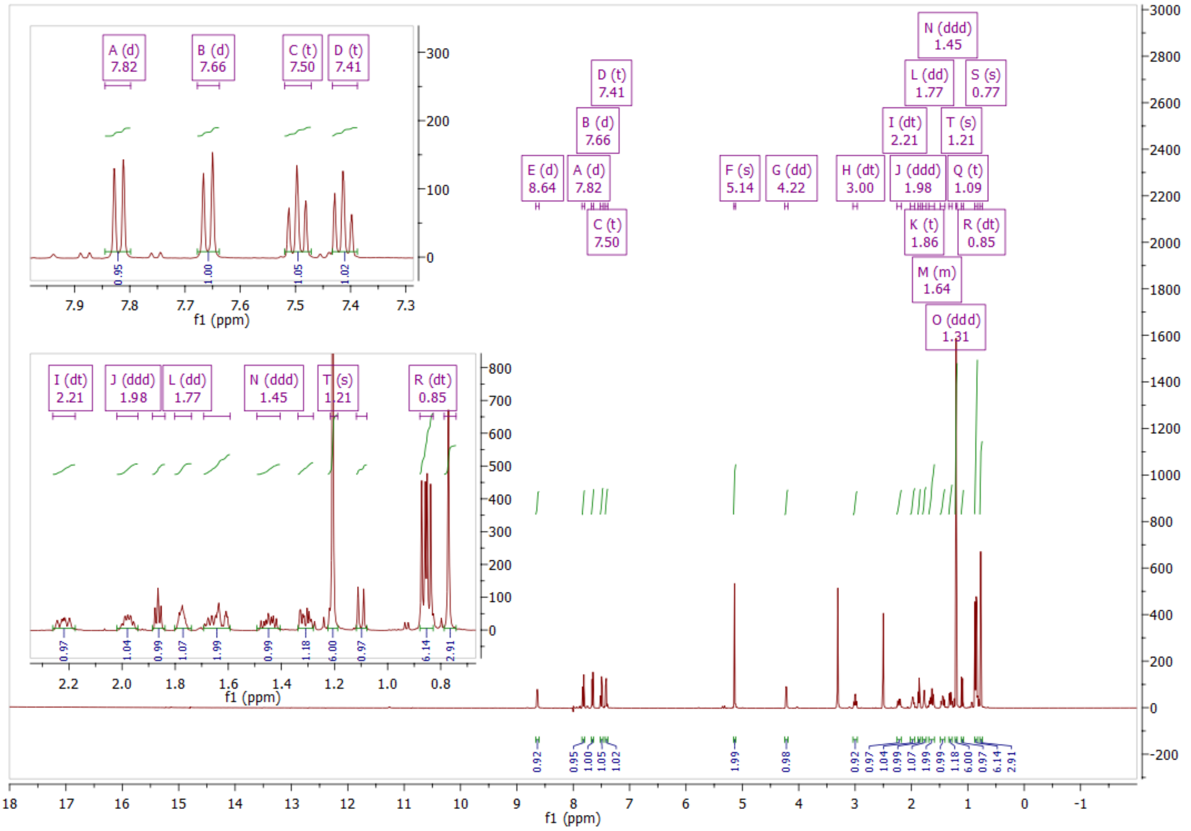
A (d) 7.82
B (d) 7.66
C (t) 7.50
D (t) 7.41
E (d) 8.64
F (s) 5.14
G (dd) 4.22
H (dt) 3.00
I (dt) 2.21
J (ddd) 1.98
K (t) 1.86
L (dd) 1.77
M (m) 1.64
N (ddd) 1.45
O (ddd) 1.31
Q (t) 1.09
R (dt) 0.85
S (s) 0.77
T (s) 1.21
f1 (ppm)

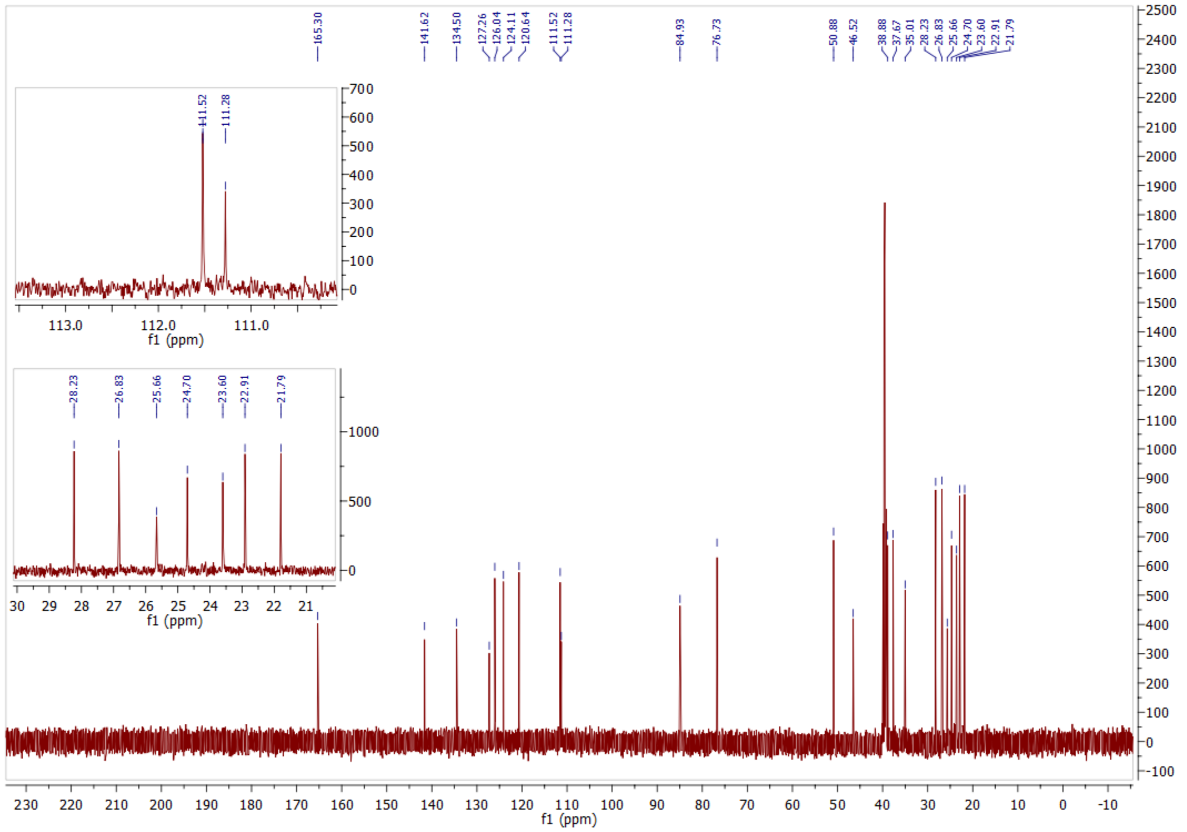
165.30
141.62
134.50
127.26
126.04
124.11
120.64
111.52
111.28
84.93
76.73
50.88
46.52
38.88
37.67
35.01
28.23
26.83
25.66
24.70
23.60
22.91
21.79
f1 (ppm)

[(1*R*)-1-[2-(2-Cyano-1*H*-1,3-benzodiazol-1-yl)acetamido]-3-methylbutyl]boronic acid (33)

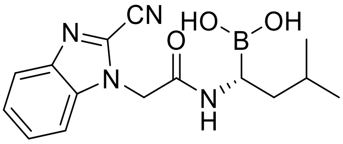

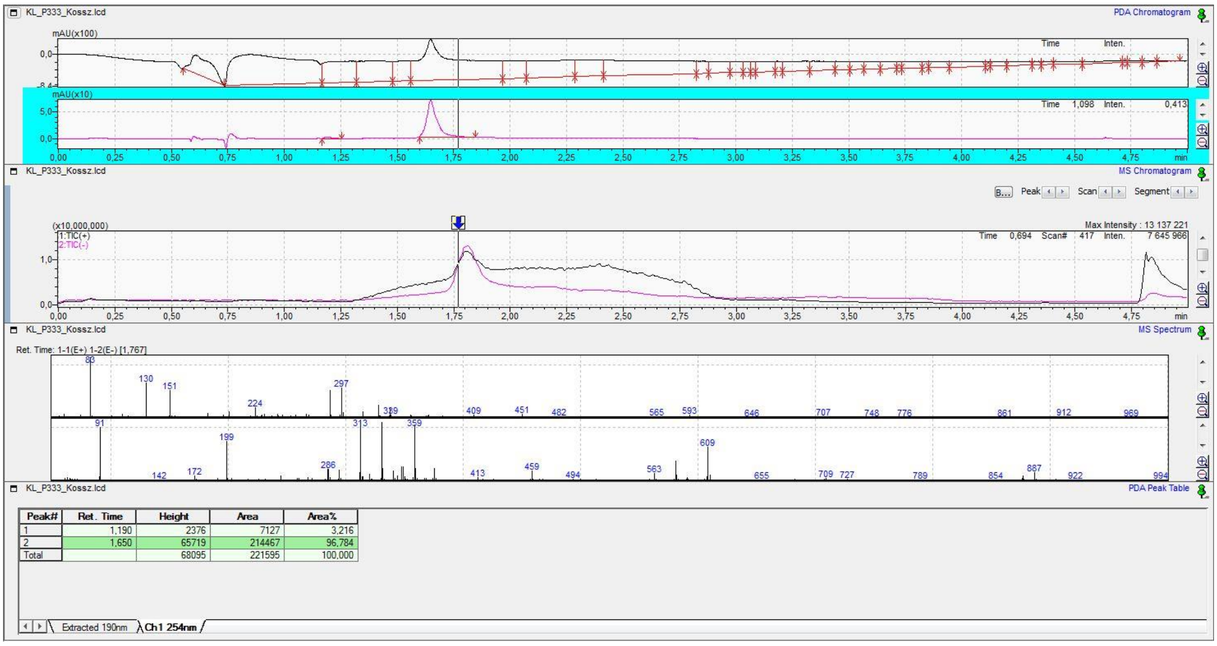

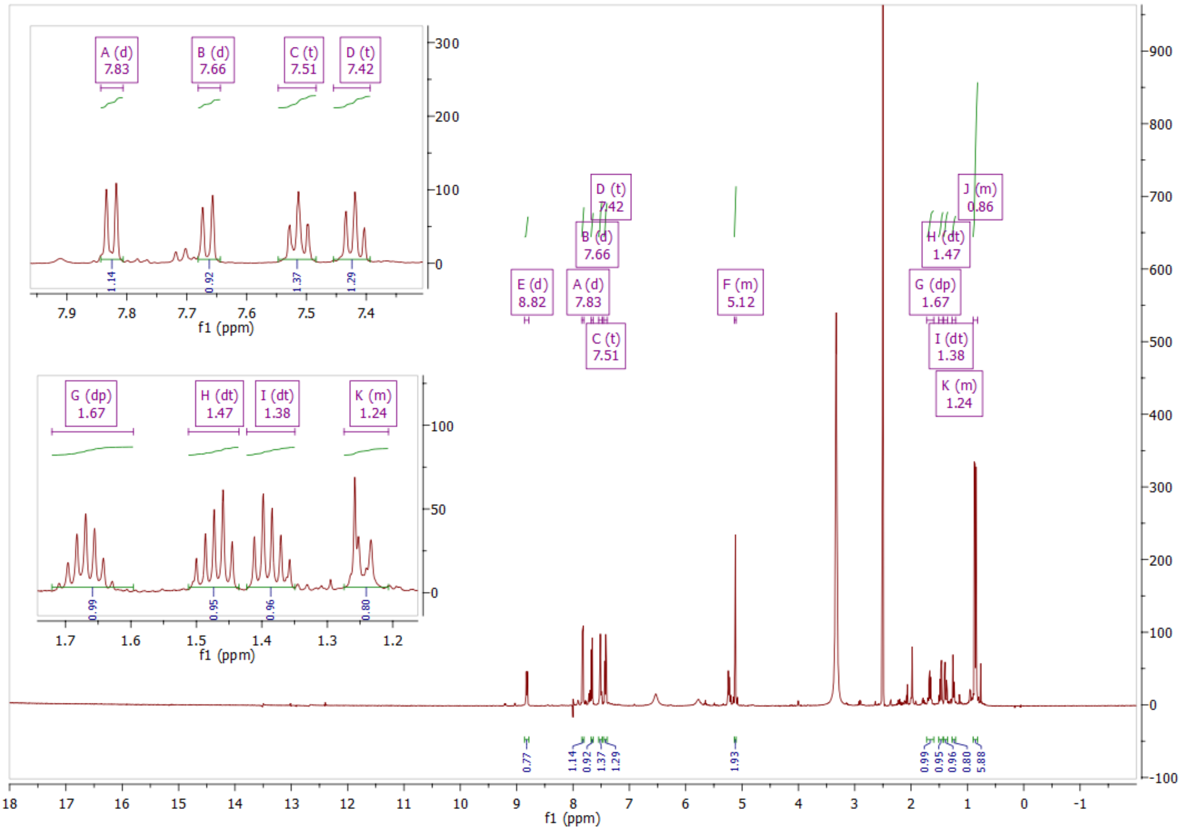

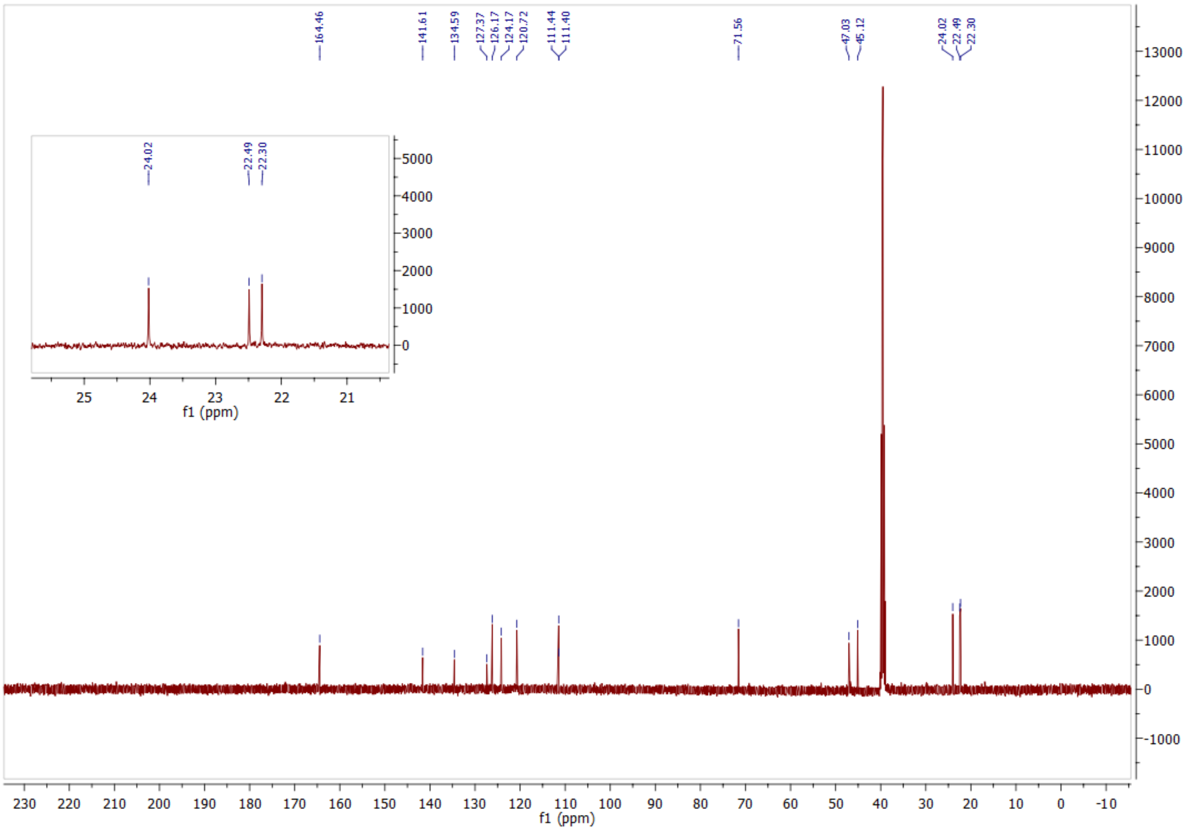

3-(2-Cyano-1*H*-1,3-benzodiazol-1-yl)propanoic acid (XXXI)

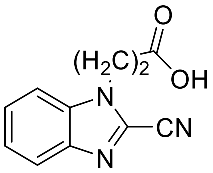

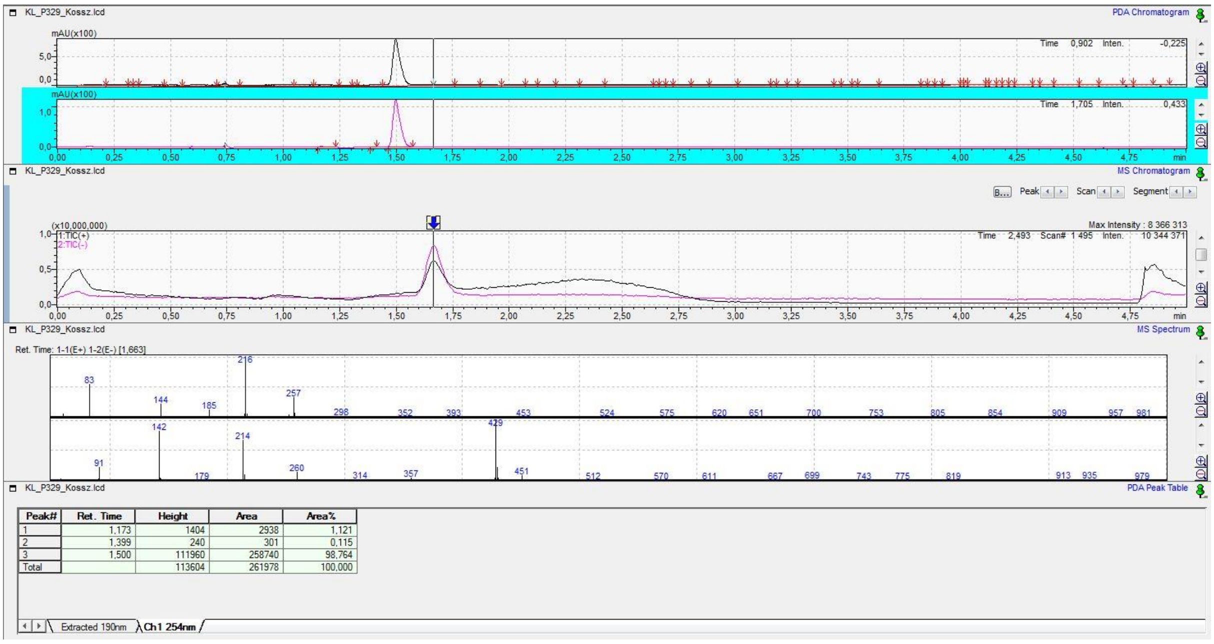

Peak#	Ret. Time	Height	Area	Area%
1	1,173	1404	2938	1,121
2	1,399	240	301	0,115
3	1,500	111960	258740	98,764
Total		113604	261978	100,000

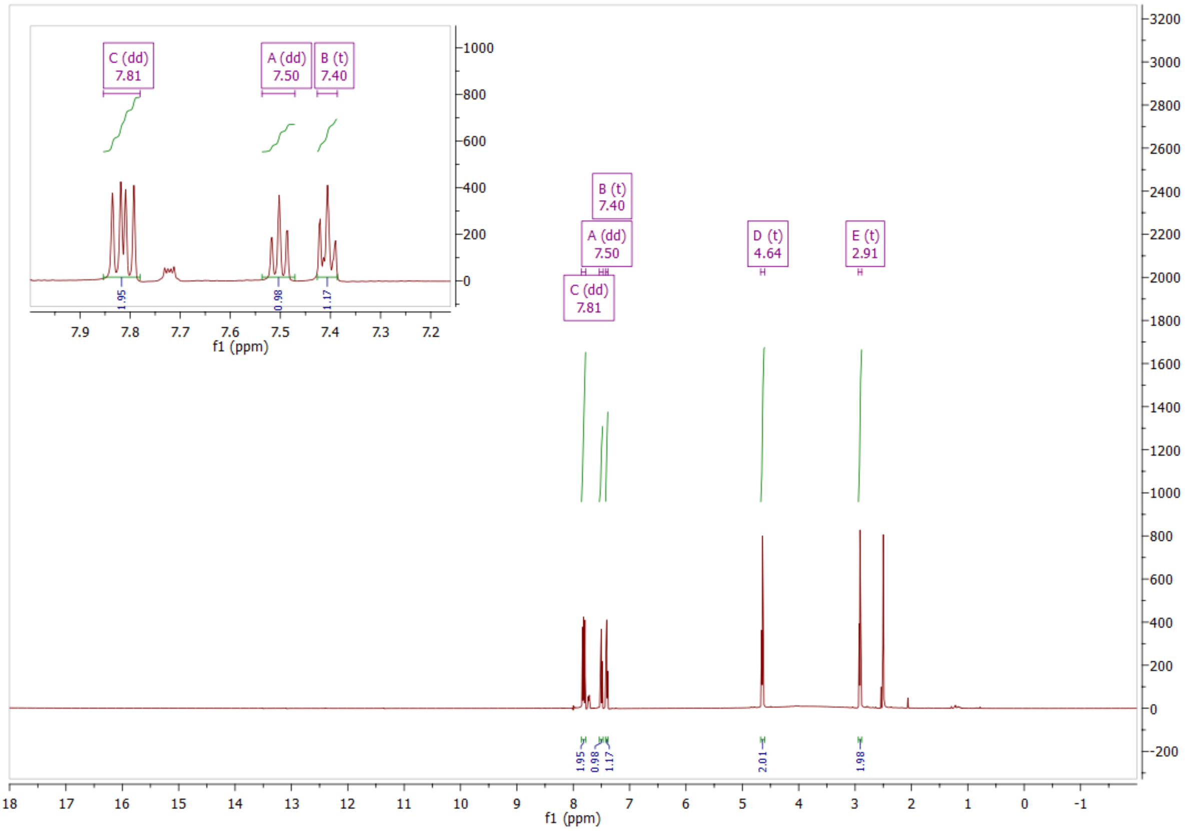

C (dd)
7.81
A (dd)
7.50
B (t)
7.40
D (t)
4.64
E (t)
2.91
1.95
0.98
1.17
2.01
1.98
f1 (ppm)

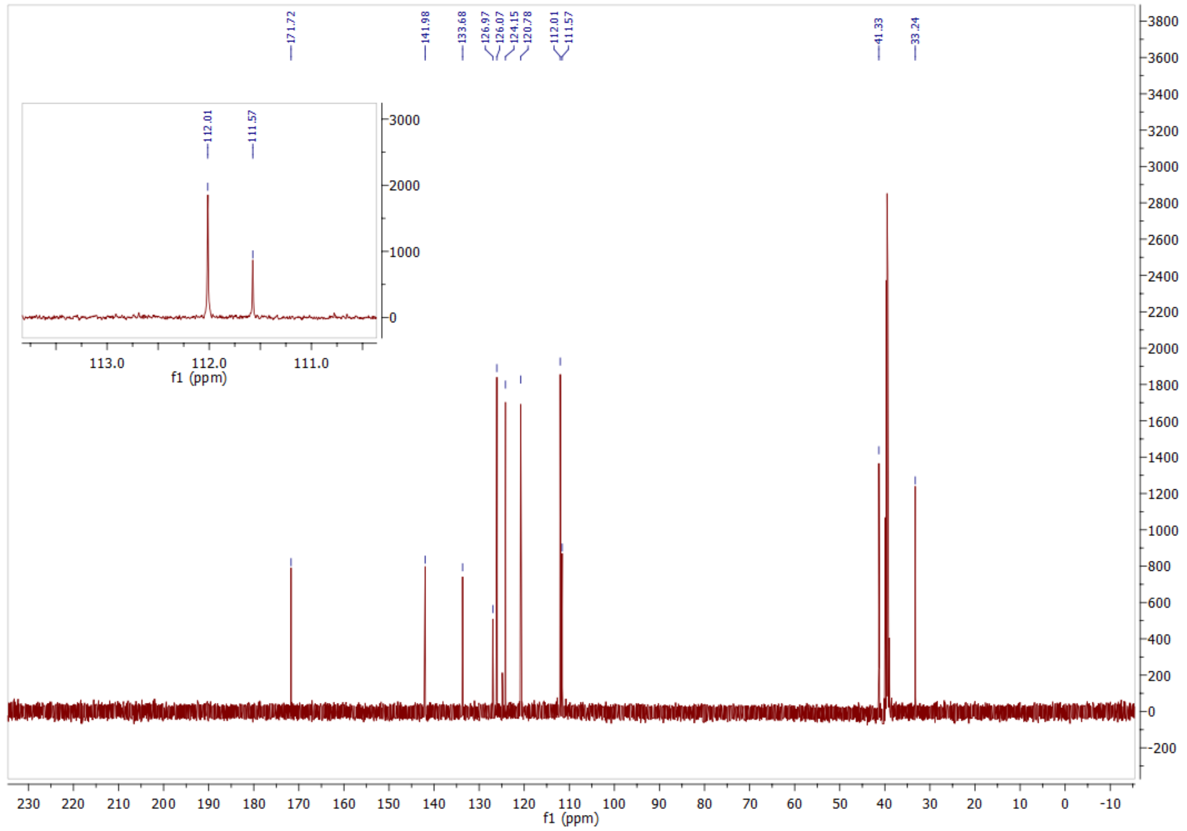

171.72
141.98
133.68
126.97
126.07
124.15
120.78
112.01
111.57
41.33
33.24
f1 (ppm)

3-(2-Cyano-1*H*-1,3-benzodiazol-1-yl)-*N*-[(1*R*)-3-methyl-1-[(1*S*,2*S*,6*R*,8*S*)-2,9,9-trimethyl-3,5-dioxa-4-boratricyclo[6.1.1.0^2,6^]decan-4-yl]butyl]propanamide (30)

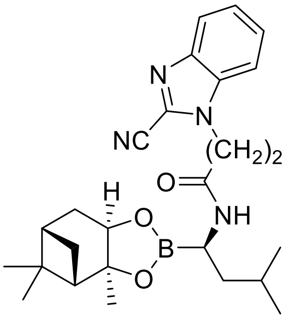

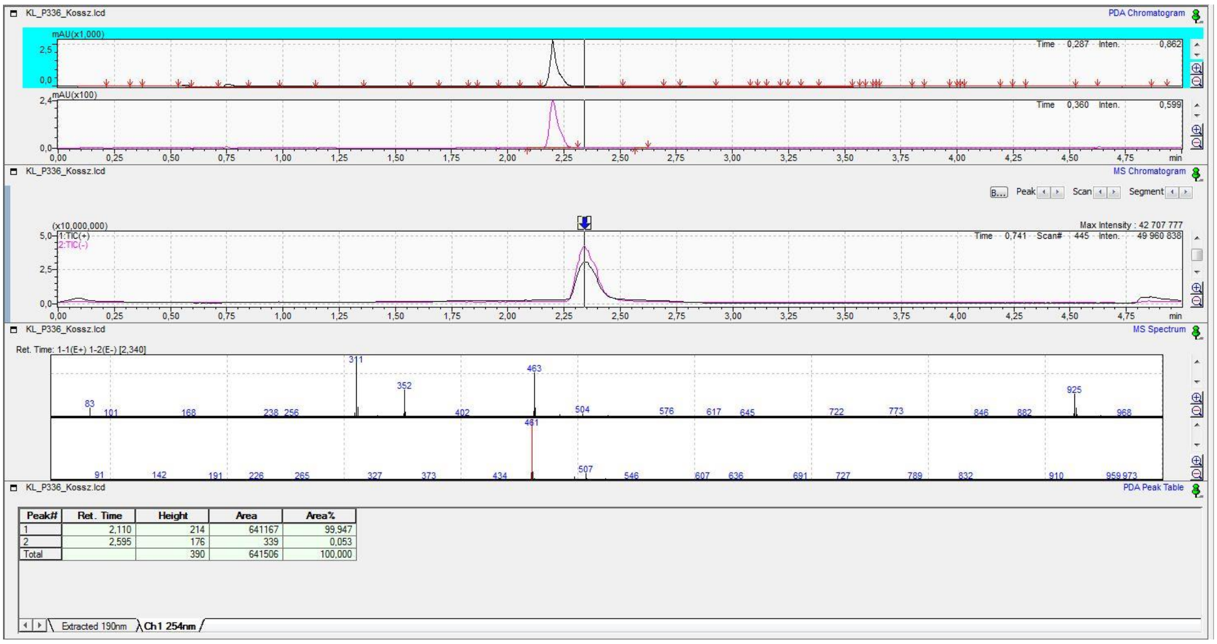

Peak#	Ret. Time	Height	Area	Area%
1	2,110	214	641167	99,947
2	2,595	176	339	0,053
Total		390	641506	100,000

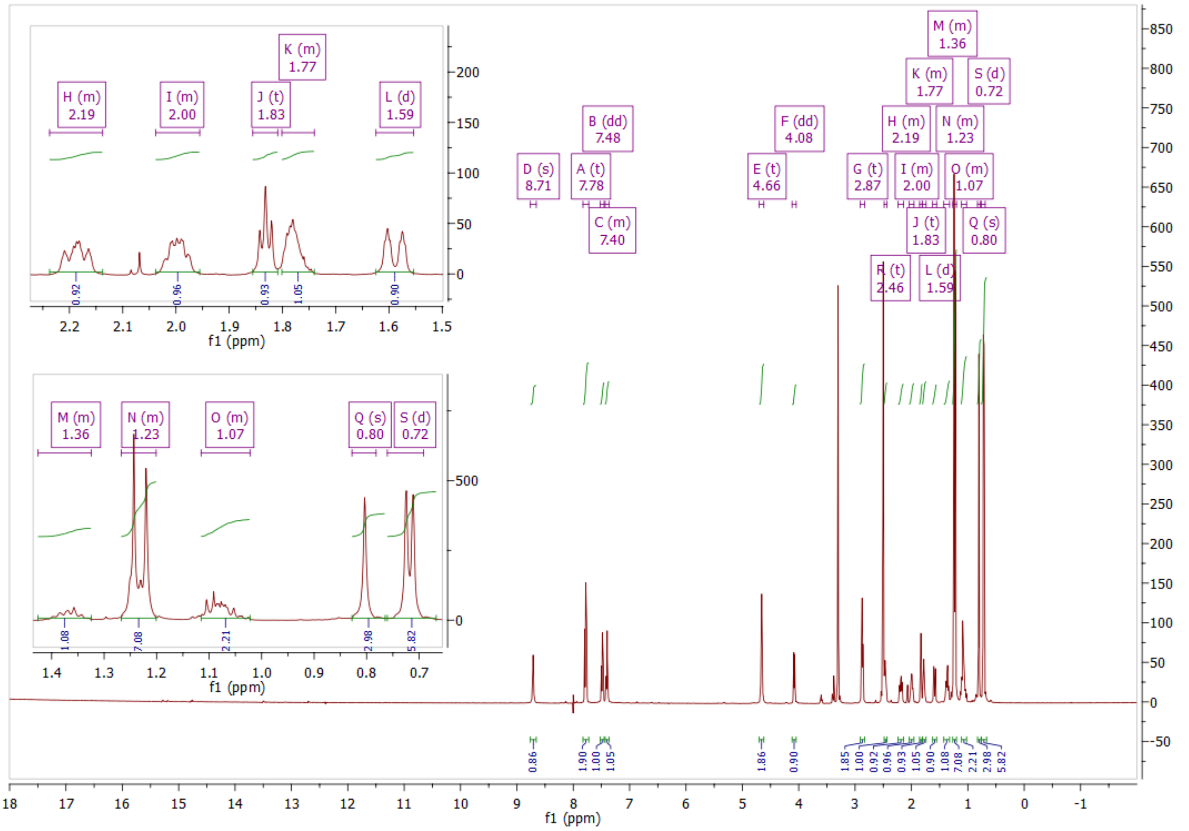

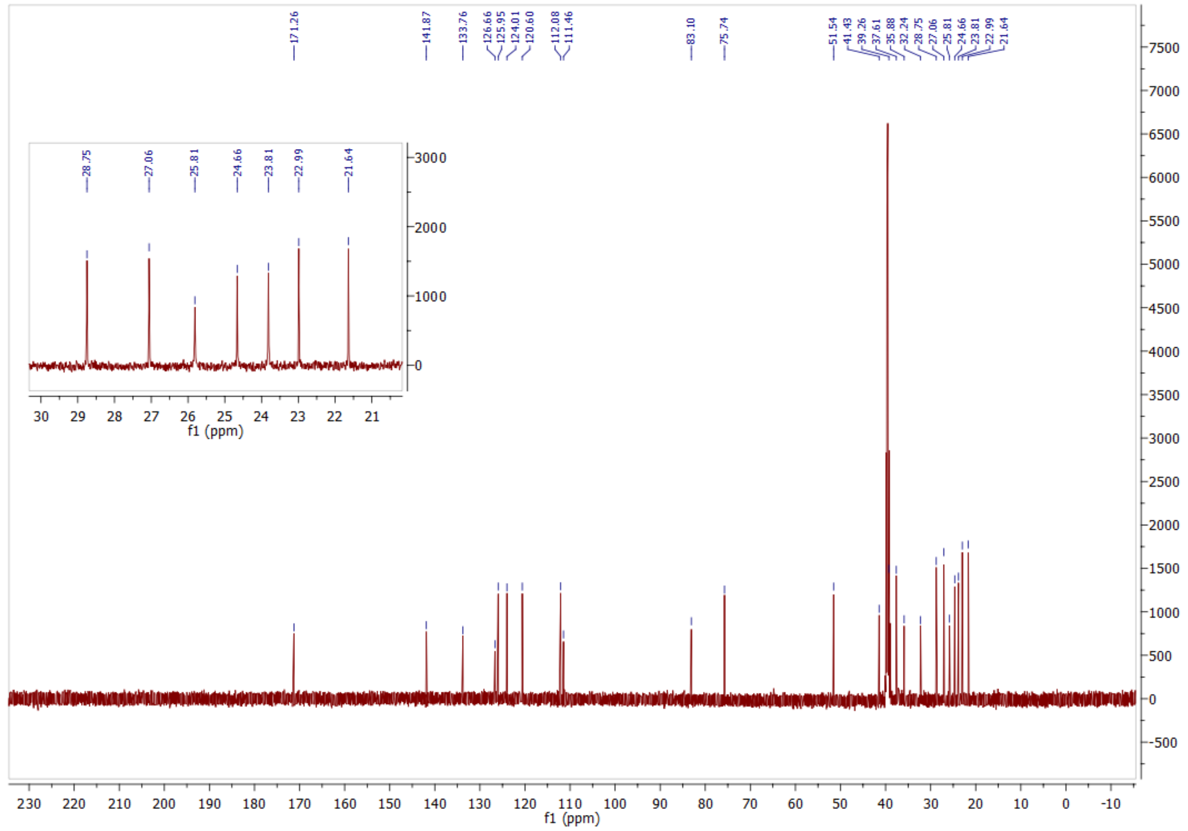

[(1*R*)-1-[3-(2-Cyano-1*H*-1,3-benzodiazol-1-yl)propanamido]-3-methylbutyl]boronic acid (34)

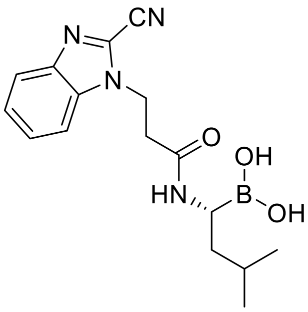

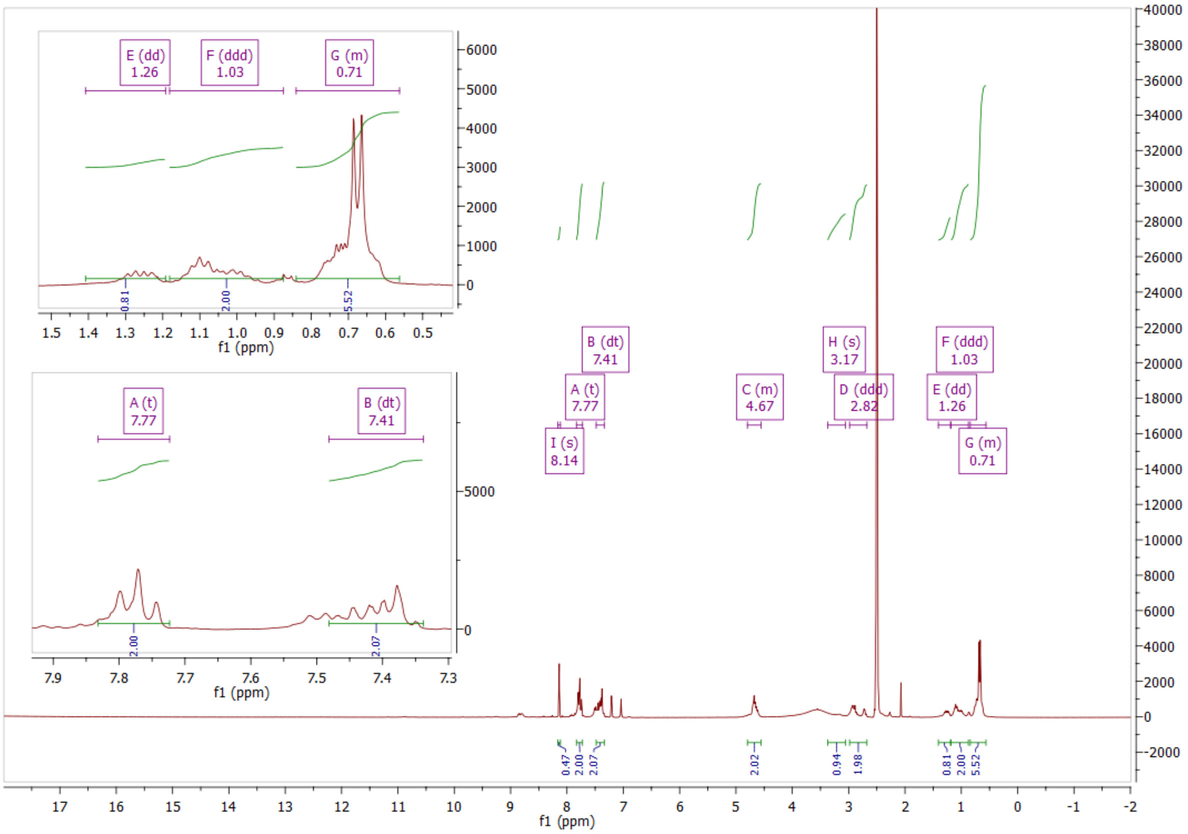

E (dd)
1.26
F (ddd)
1.03
G (m)
0.71
A (t)
7.77
B (dt)
7.41
I (s)
8.14
C (m)
4.67
H (s)
3.17
D (ddd)
2.82
f1 (ppm)

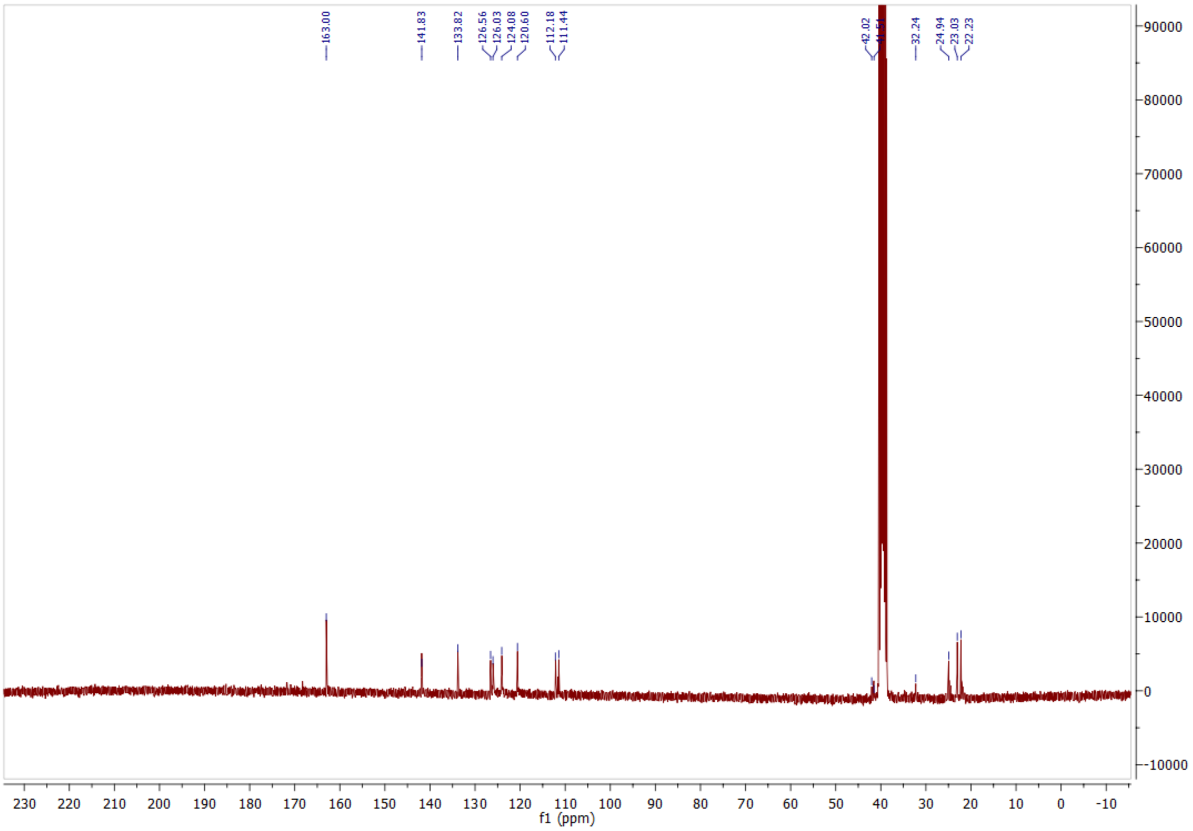

4-(2-Cyano-1*H*-1,3-benzodiazol-1-yl)butanoic acid (XXXII)

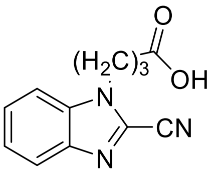

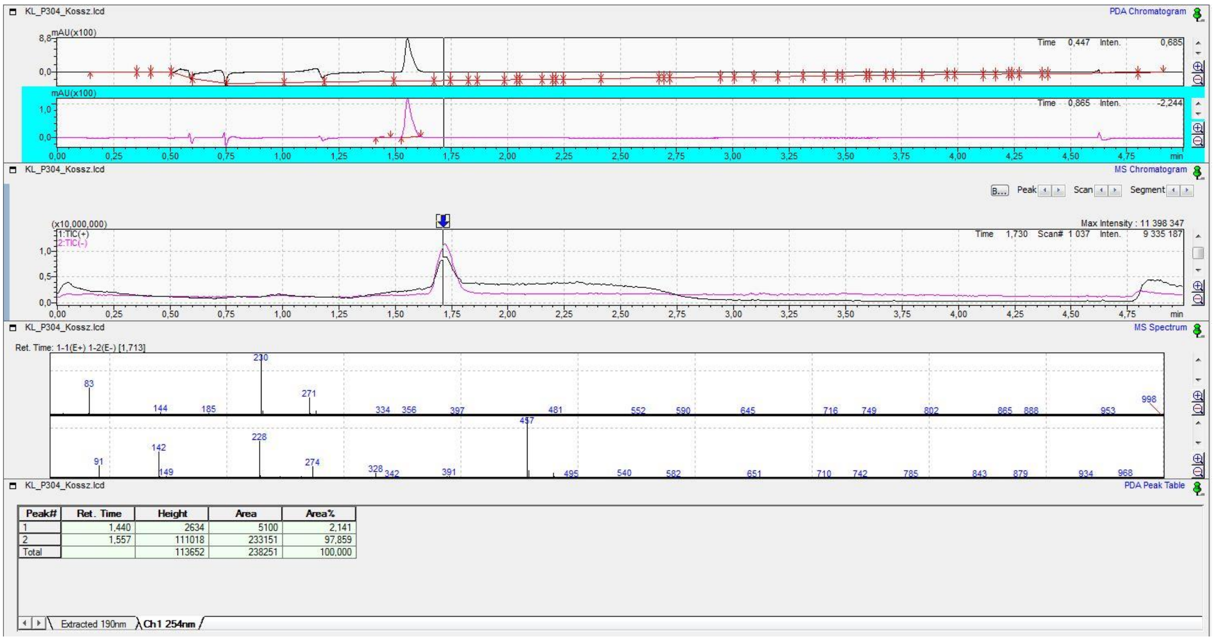

Peak#	Ret. Time	Height	Area	Area%
1	1,440	2634	5100	2,141
2	1,557	111018	233151	97,859
Total		113652	238251	100,000

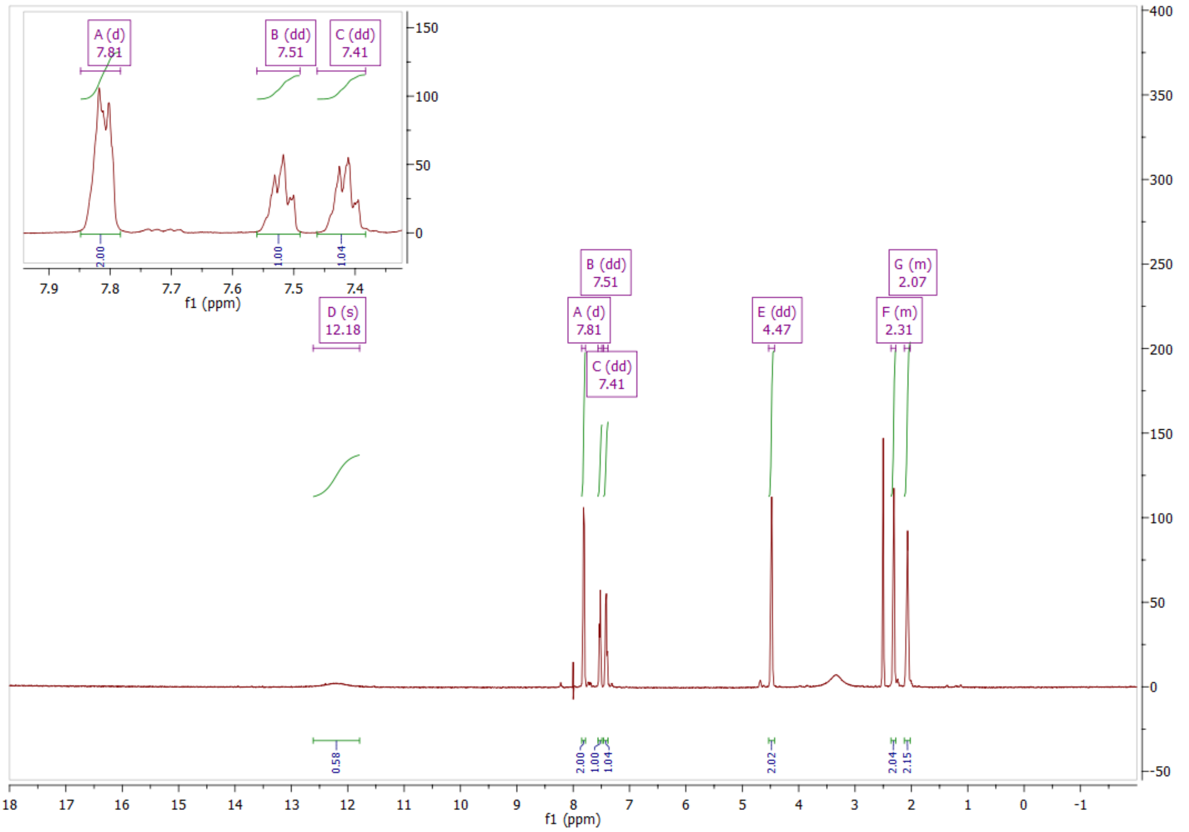

A (d)
7.81
B (dd)
7.51
C (dd)
7.41
D (s)
12.18
E (dd)
4.47
F (m)
2.31
G (m)
2.07
f1 (ppm)

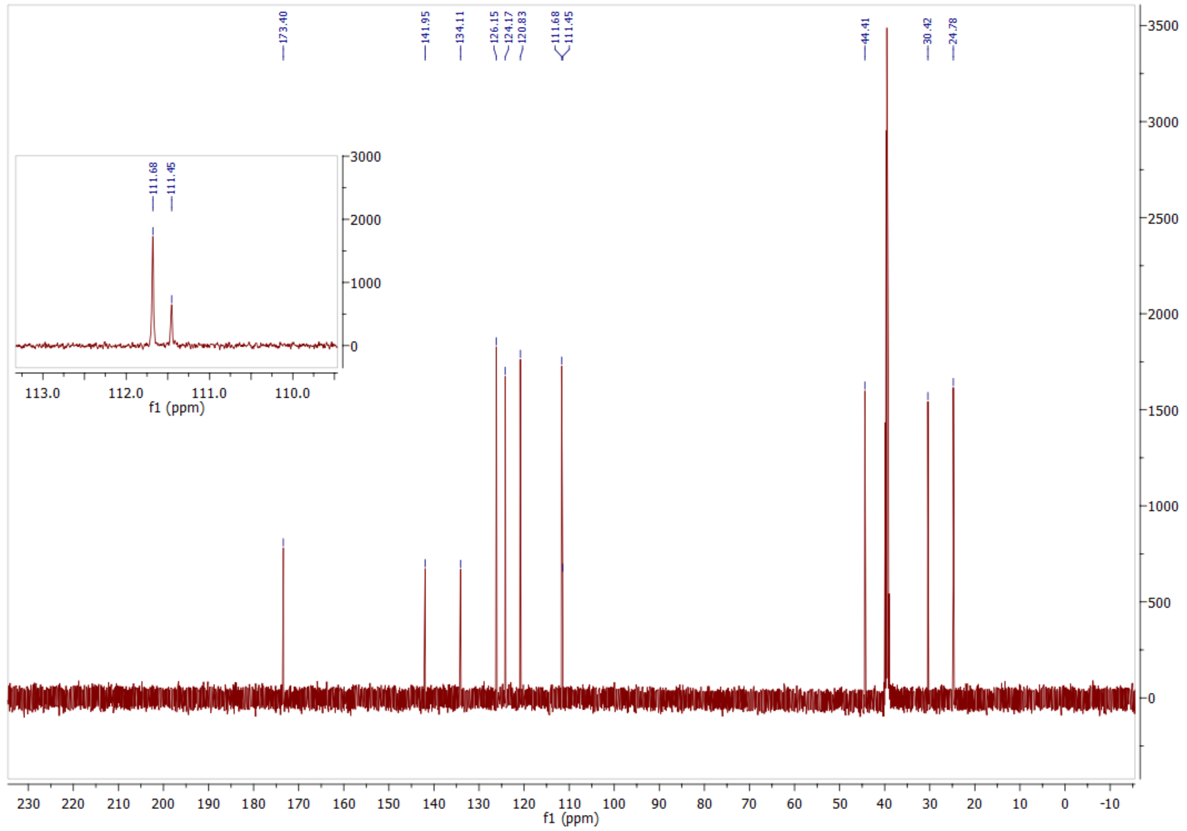

173.40
141.95
134.11
126.15
124.17
120.83
111.68
111.45
44.41
30.42
24.78
f1 (ppm)

4-(2-Cyano-1*H*-1,3-benzodiazol-1-yl)-*N*-[(1*R*)-3-methyl-1-[(1*S*,2*S*,6*R*,8*S*)-2,9,9-trimethyl-3,5-dioxa-4-boratricyclo[6.1.1.0^{2,6}]decan-4-yl]butyl]butanamide (31)

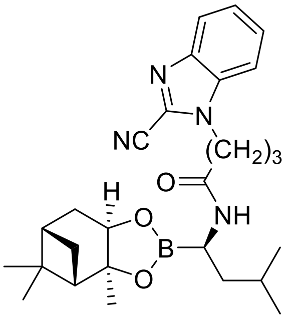

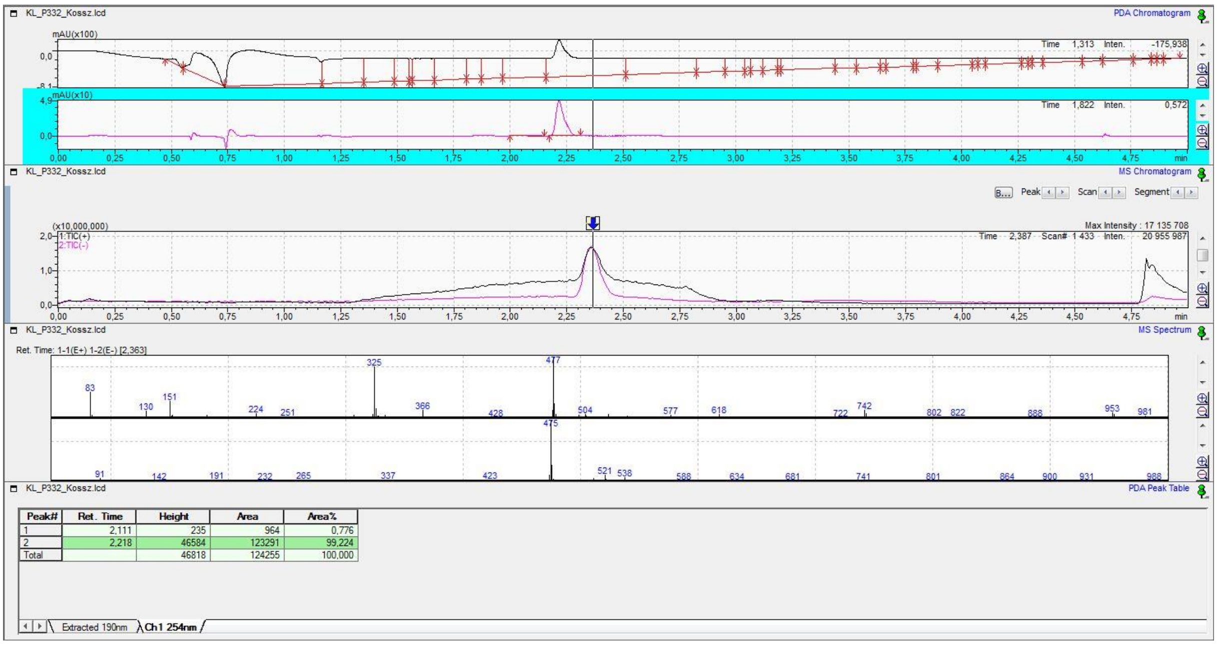

Peak#	Ret. Time	Height	Area	Area%
1	2,111	235	964	0,776
2	2,218	46584	123291	99,224
Total		46818	124255	100,000

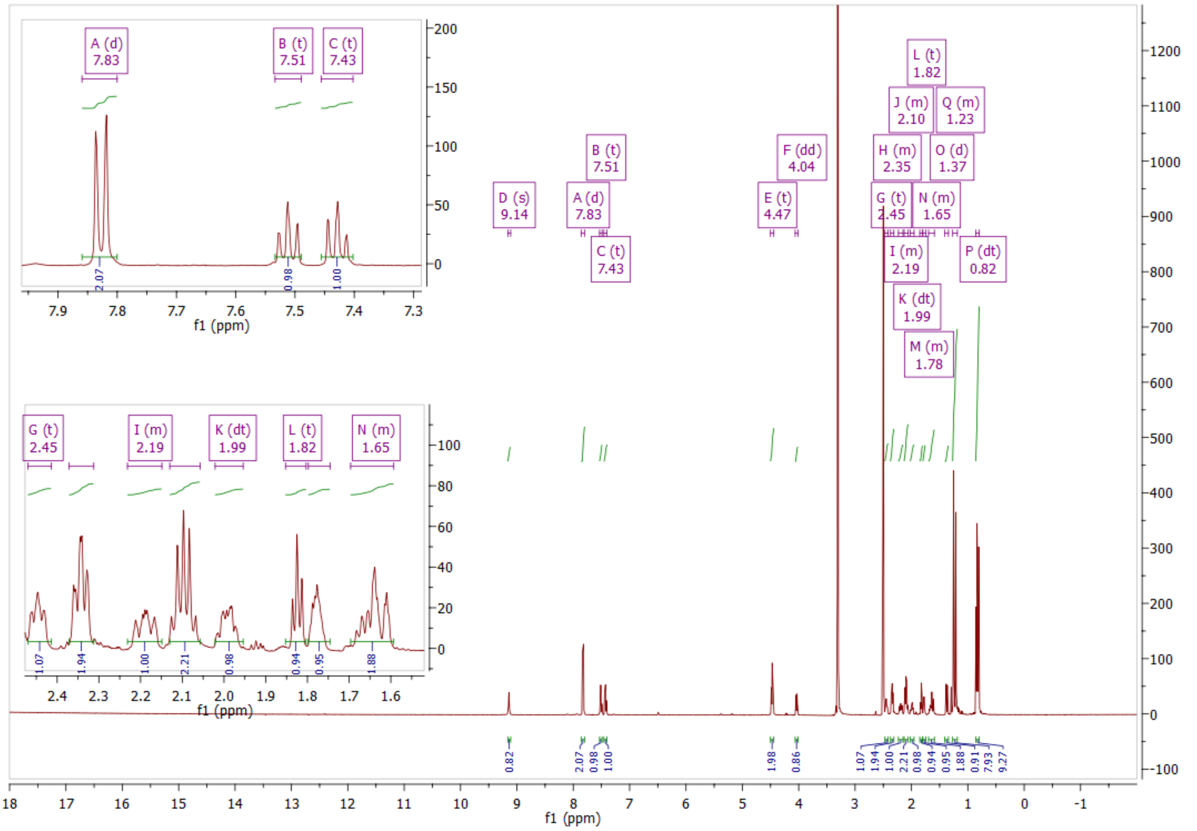

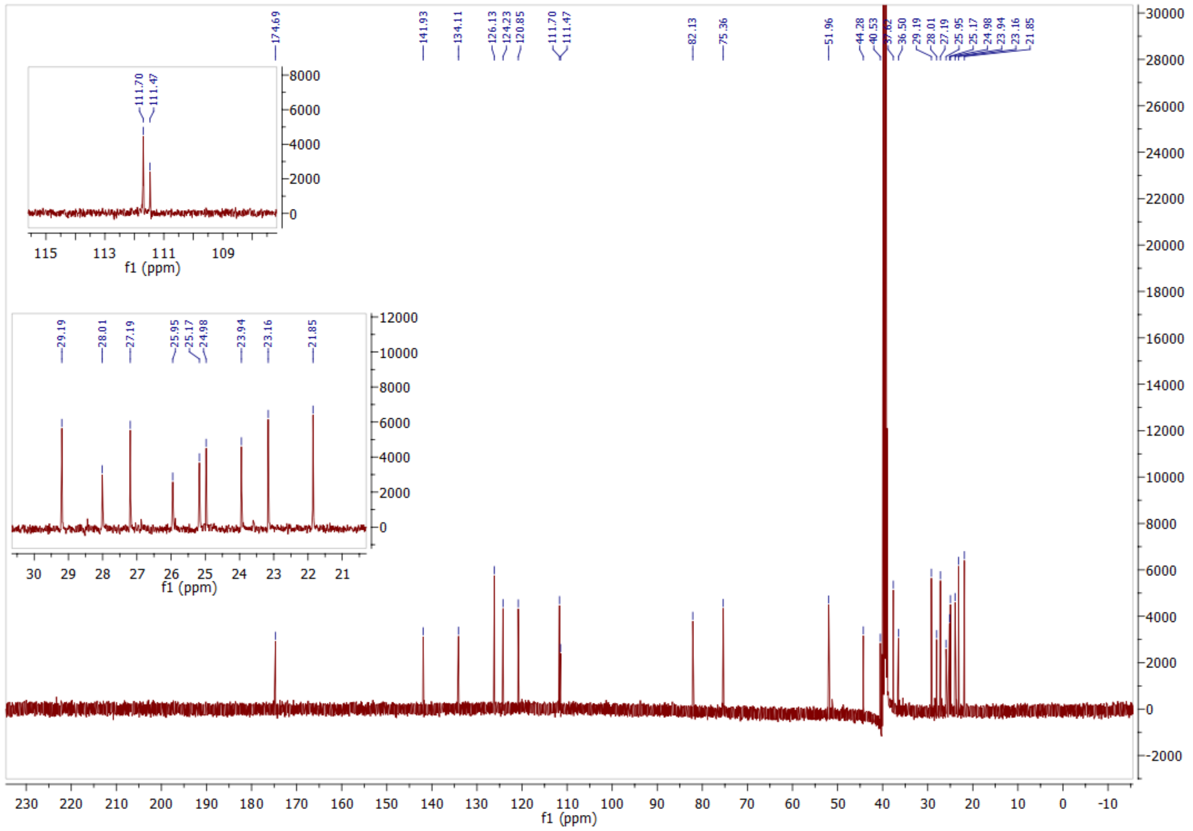

[(1*R*)-1-[4-(2-Cyano-1*H*-1,3-benzodiazol-1-yl)butanamido]-3-methylbutyl]boronic acid (35)

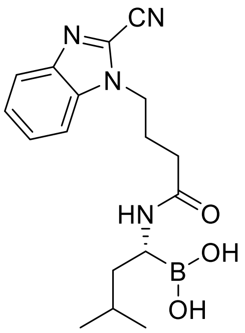

CN
N
N
HN
O
B
OH
OH

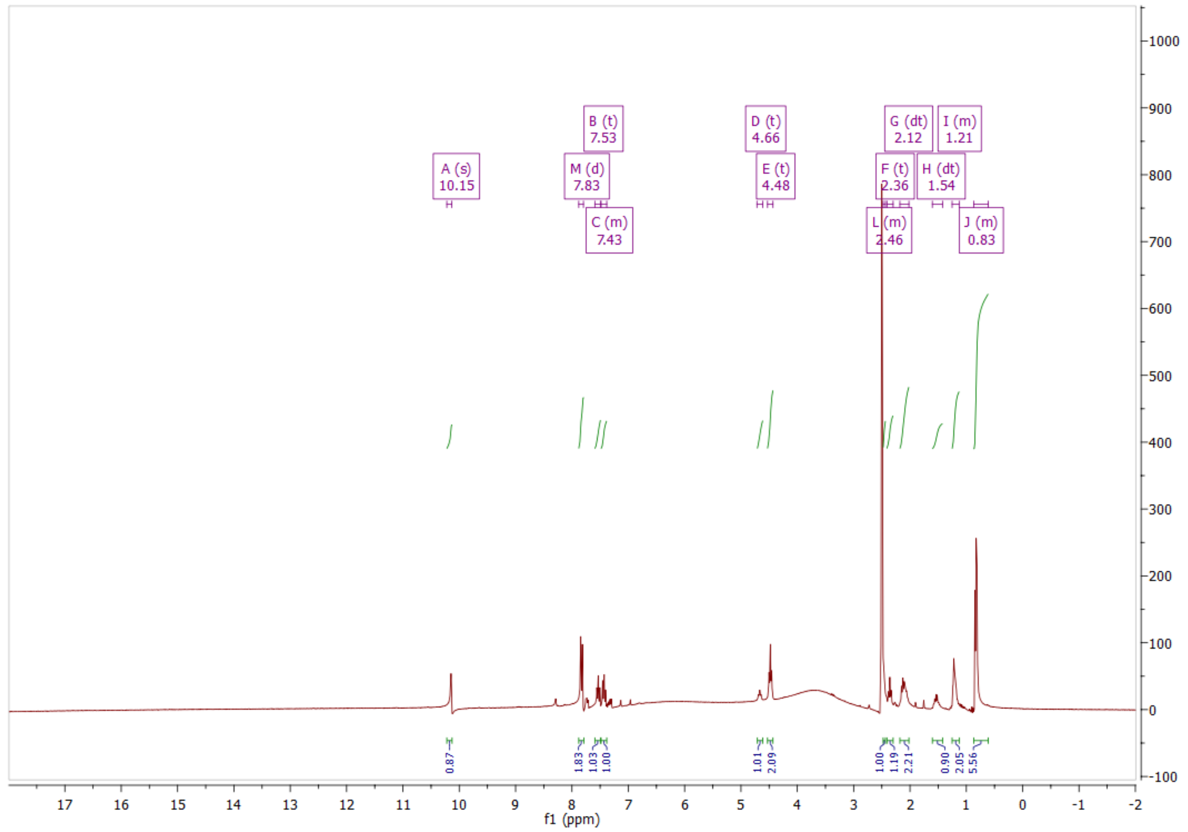

A (s)
10.15
M (d)
7.83
B (t)
7.53
C (m)
7.43
D (t)
4.66
E (t)
4.48
G (dt)
2.12
I (m)
1.21
F (t)
2.36
H (dt)
1.54
L (m)
2.46
J (m)
0.83
0.87
1.83
1.03
1.00
1.01
2.09
1.00
1.19
2.21
0.90
2.05
5.56
f1 (ppm)

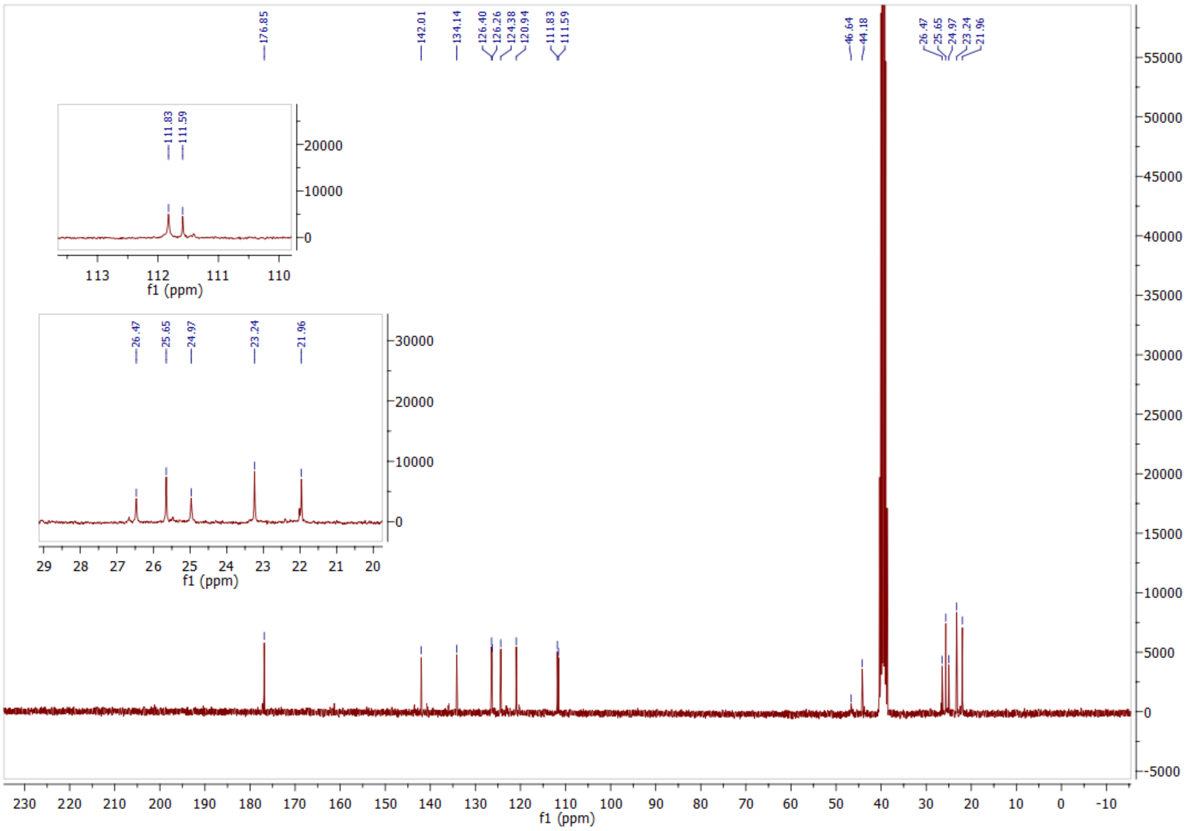

5-(2-Cyano-1*H*-1,3-benzodiazol-1-yl)pentanoic acid (XXXIII)

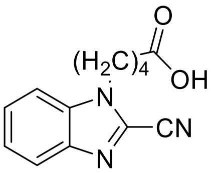

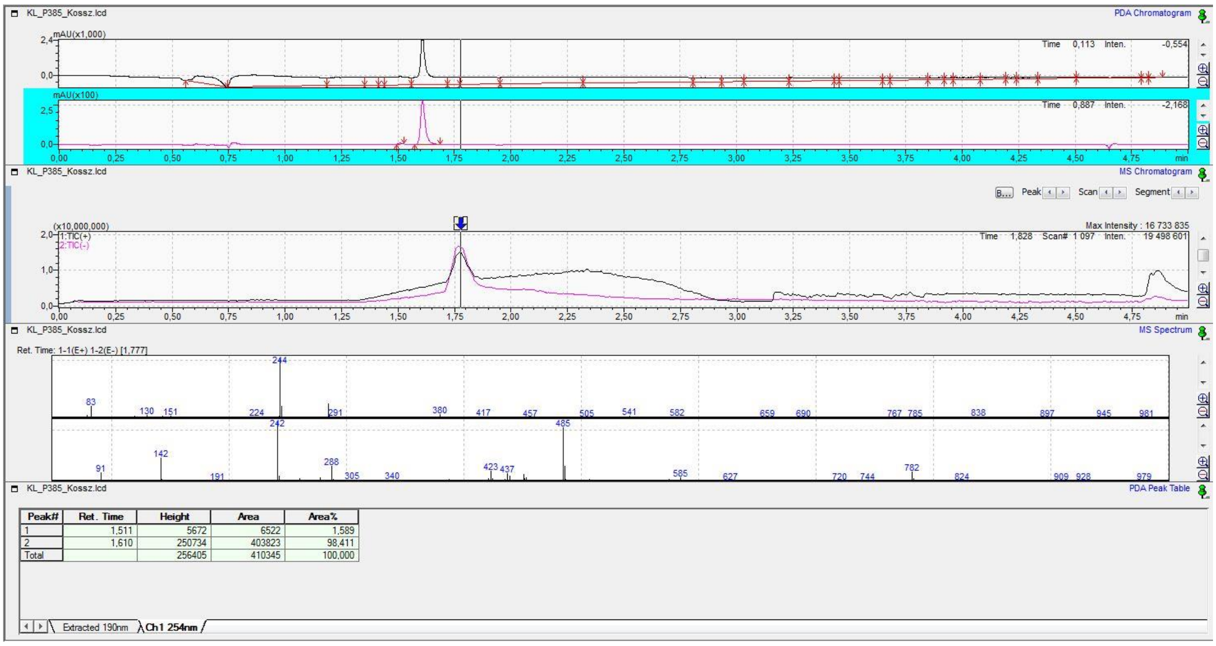

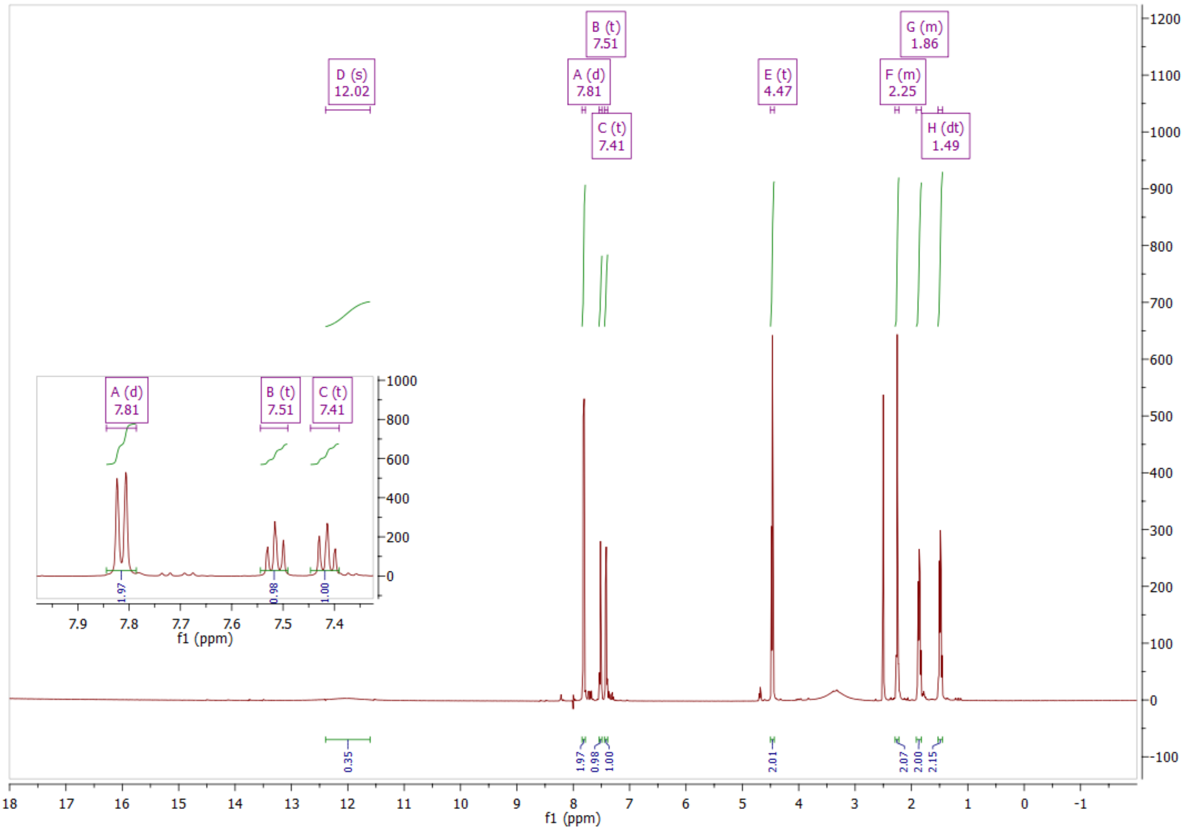

D (s)
12.02
A (d)
7.81
B (t)
7.51
C (t)
7.41
E (t)
4.47
F (m)
2.25
G (m)
1.86
H (dt)
1.49
0.35
1.97
0.98
1.00
2.01
2.07
2.00
2.15
f1 (ppm)

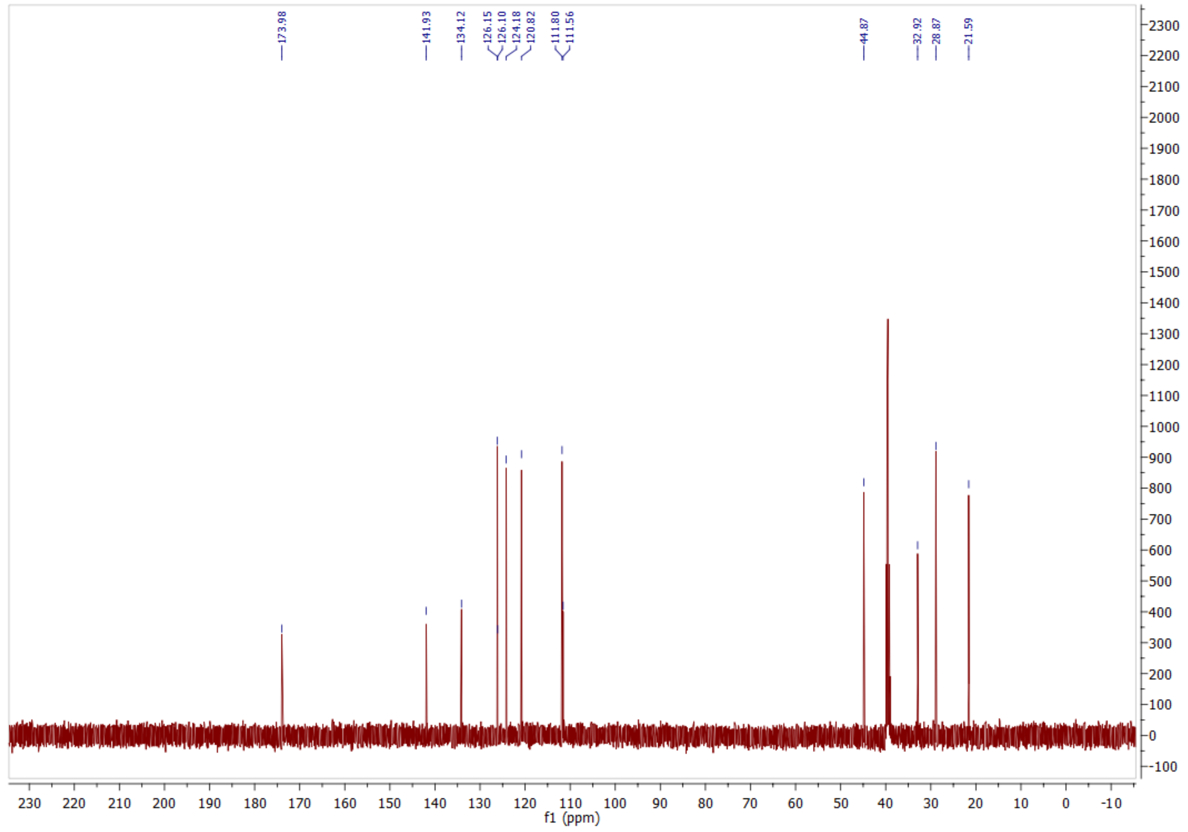

173.98
141.93
134.12
126.15
126.10
124.18
120.82
111.80
111.56
44.87
32.92
28.87
21.59
f1 (ppm)

5-(2-Cyano-1*H*-1,3-benzodiazol-1-yl)-*N*-[(1*R*)-3-methyl-1-[(1*S*,2*S*,6*R*,8*S*)-2,9,9-trimethyl-3,5-dioxa-4-boratricyclo[6.1.1.0^{2,6}]decan-4-yl]butyl]pentanamide (32)

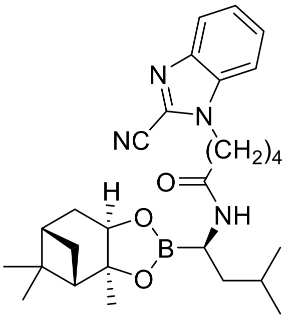

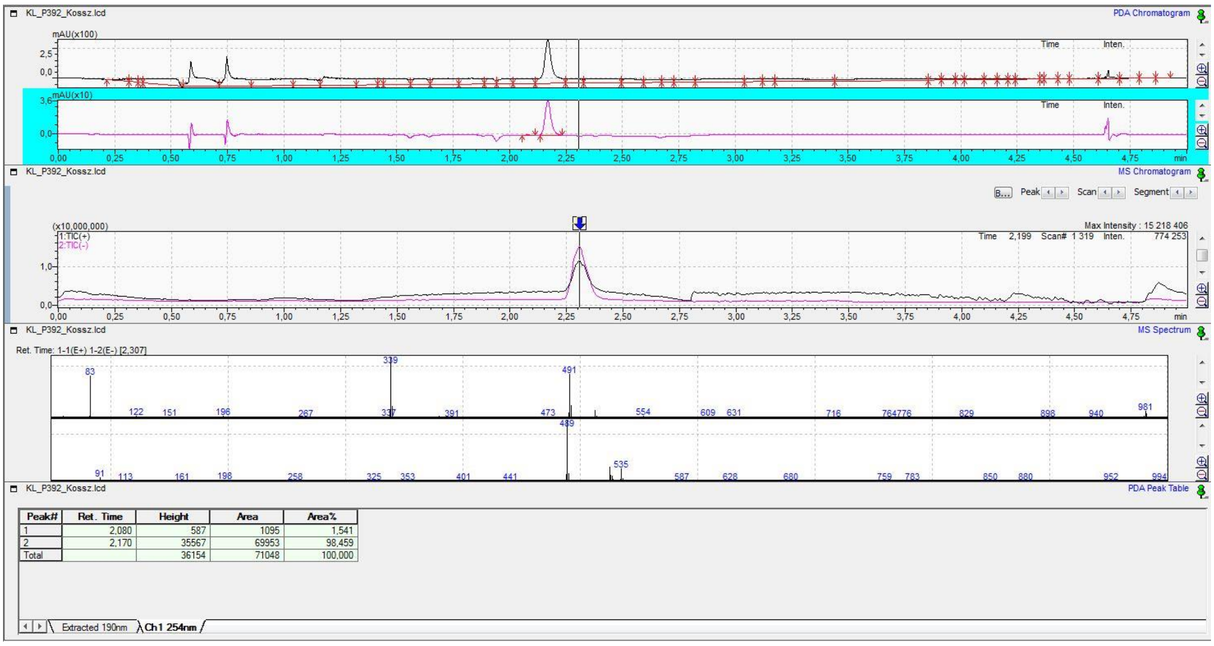

Peak#	Ret. Time	Height	Area	Area%
1	2,080	587	1095	1,541
2	2,170	35567	69953	98,459
Total		36154	71048	100,000

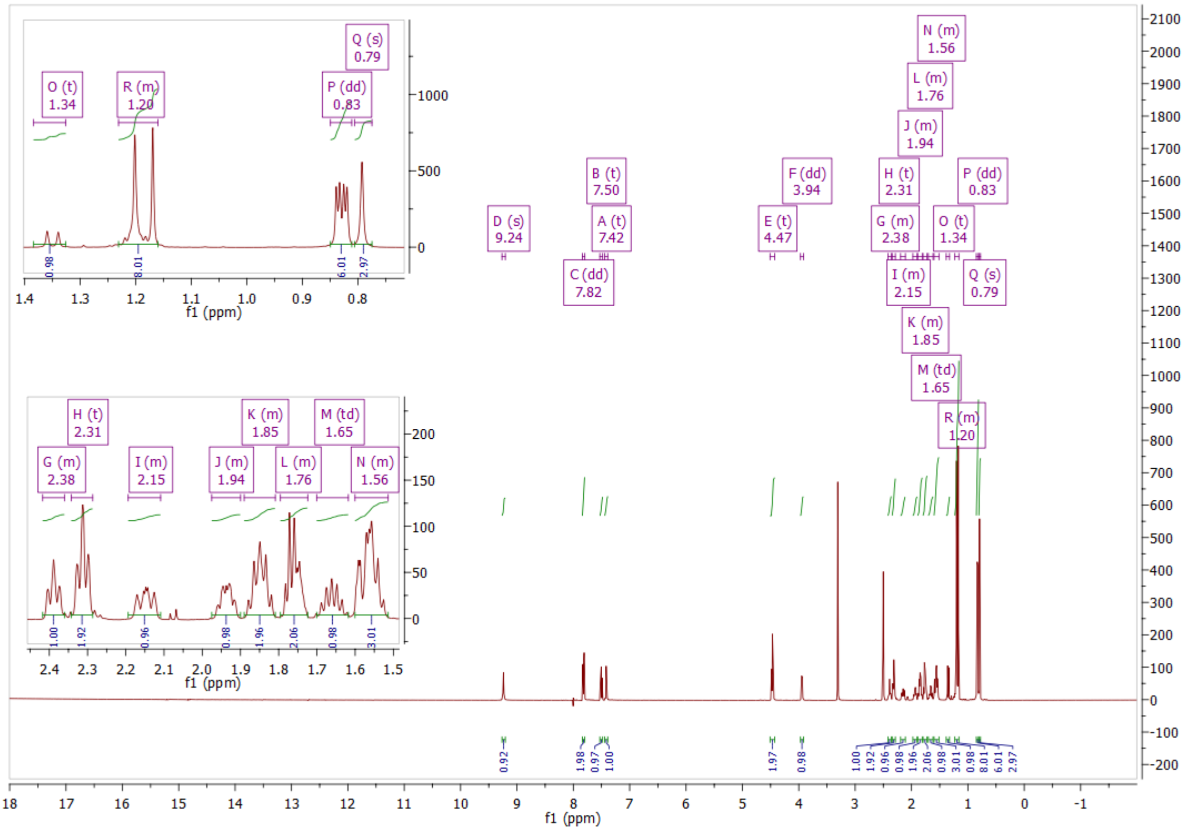

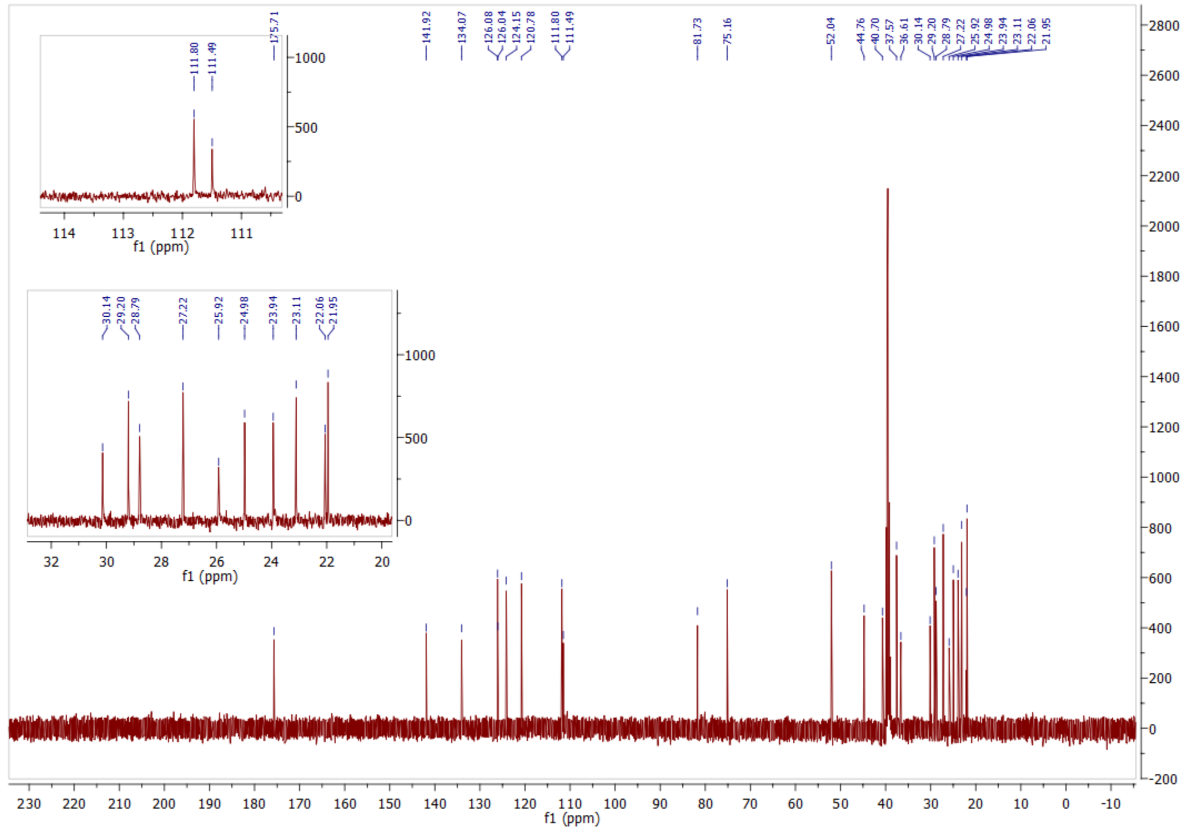

[(1*R*)-1-[5-(2-Cyano-1*H*-1,3-benzodiazol-1-yl)pentanamido]-3-methylbutyl]boronic acid (36)

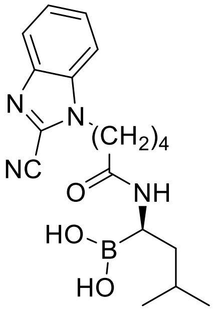
NC
N
N
(CH2)4
O
NH
HO
B
HO

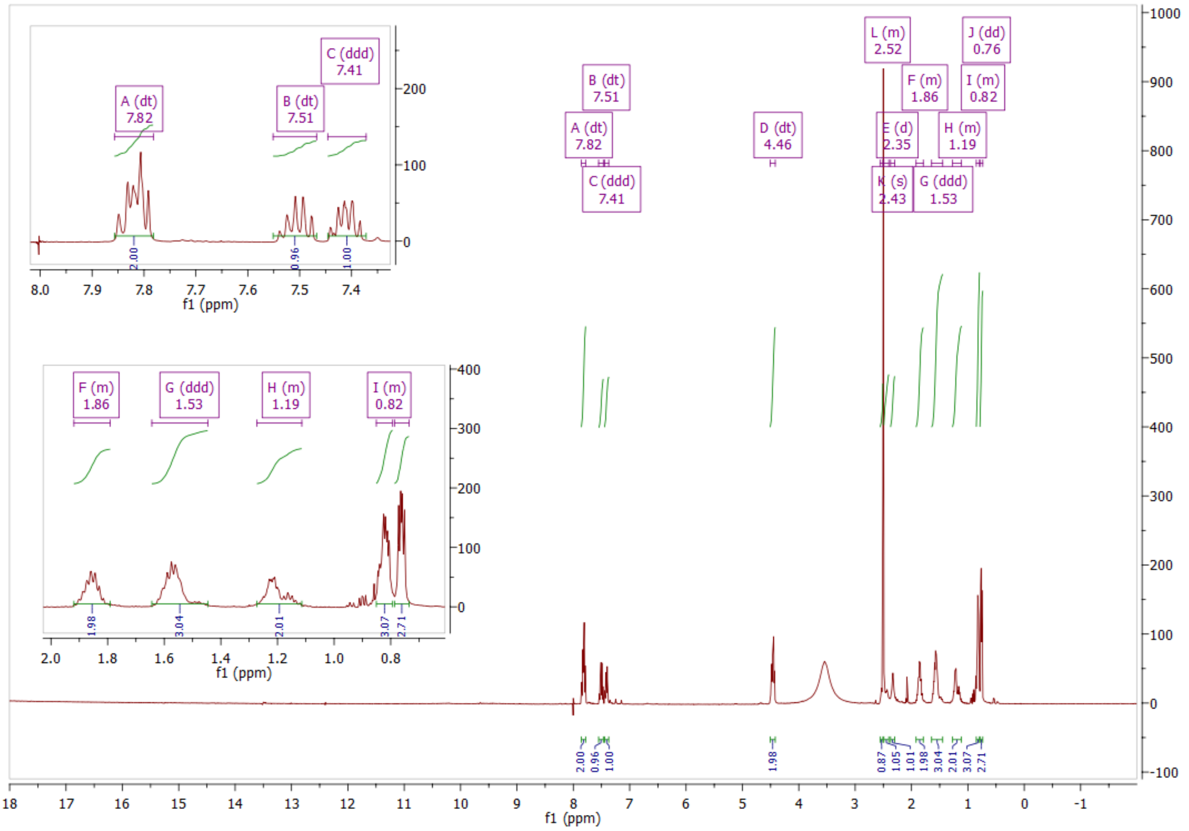
A (dt) 7.82
B (dt) 7.51
C (ddd) 7.41
D (dt) 4.46
E (d) 2.35
F (m) 1.86
G (ddd) 1.53
H (m) 1.19
I (m) 0.82
J (dd) 0.76
K (s) 2.43
L (m) 2.52
f1 (ppm)

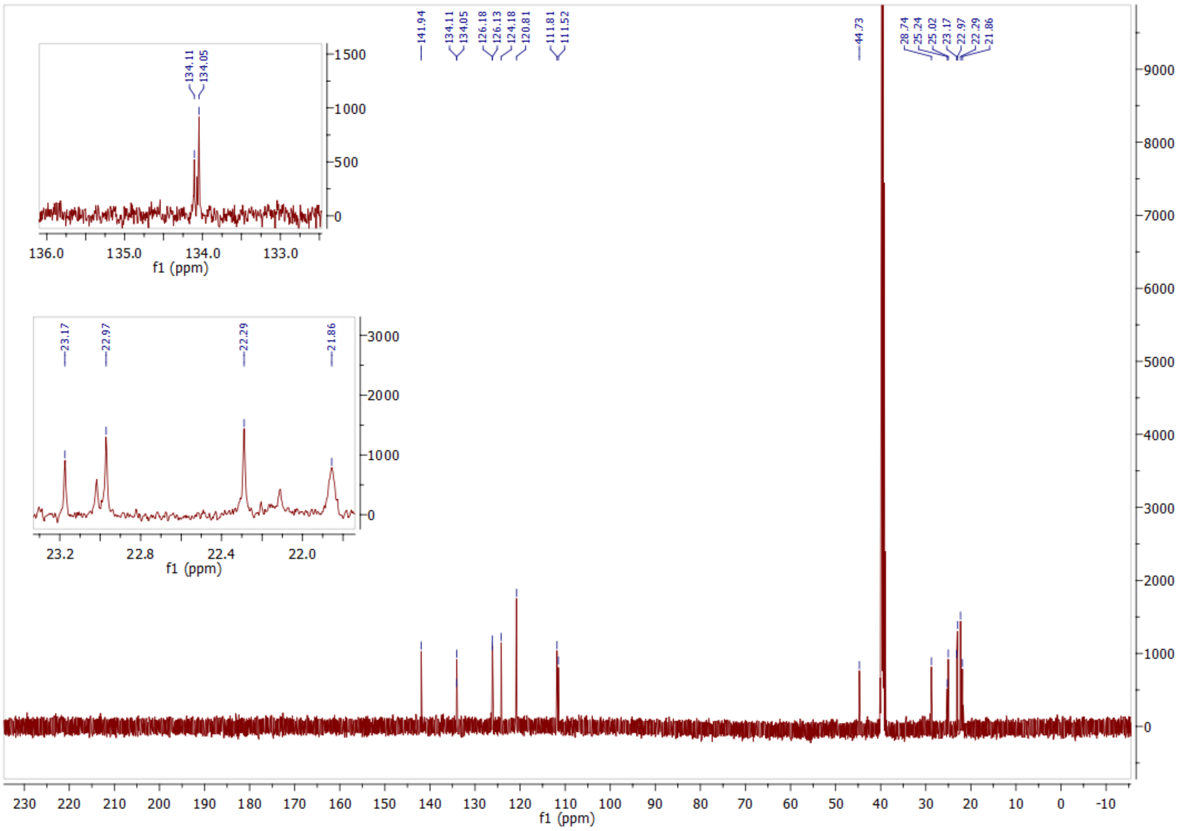

3-[(5-Chloro-2-nitrophenyl)amino]propanoic acid (XXXVI)

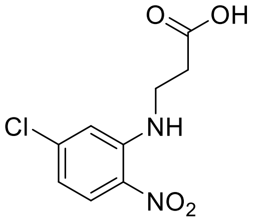

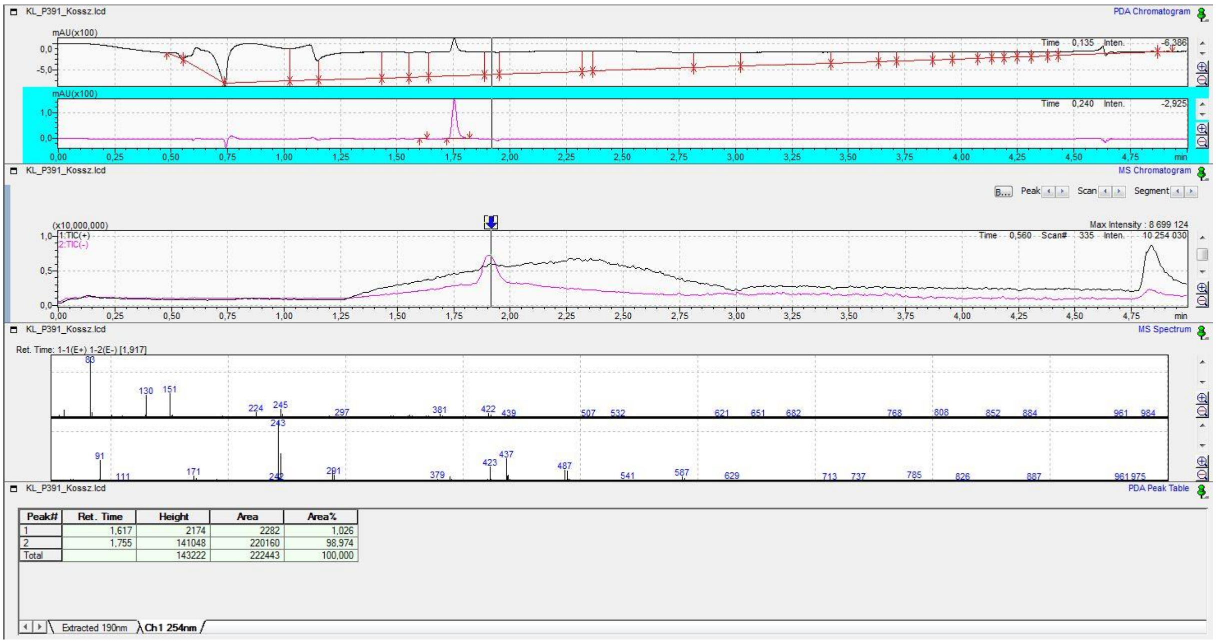

Peak#	Ret. Time	Height	Area	Area%
1	1,617	2174	2282	1,026
2	1,755	141048	220160	98,974
Total		143222	222443	100,000

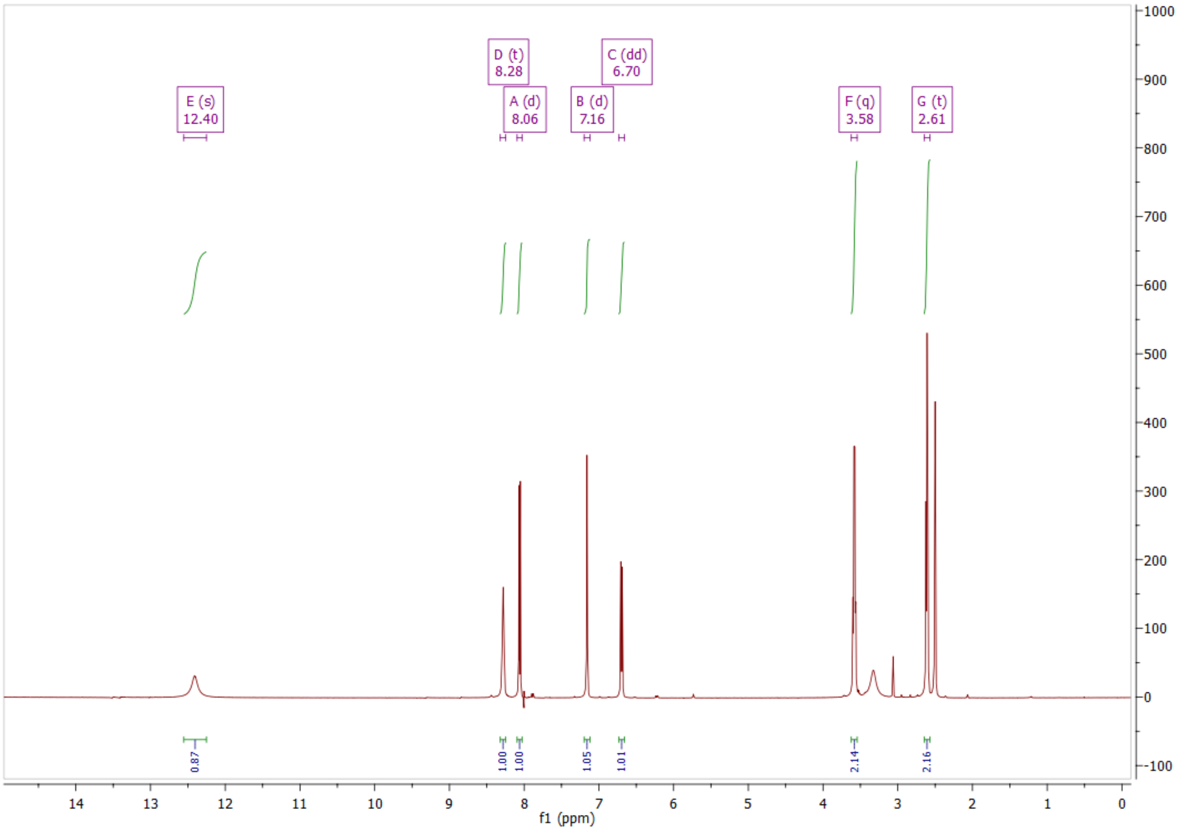

E (s)
12.40
D (t)
8.28
A (d)
8.06
B (d)
7.16
C (dd)
6.70
F (q)
3.58
G (t)
2.61
0.87
1.00
1.00
1.05
1.01
2.14
2.16
f1 (ppm)

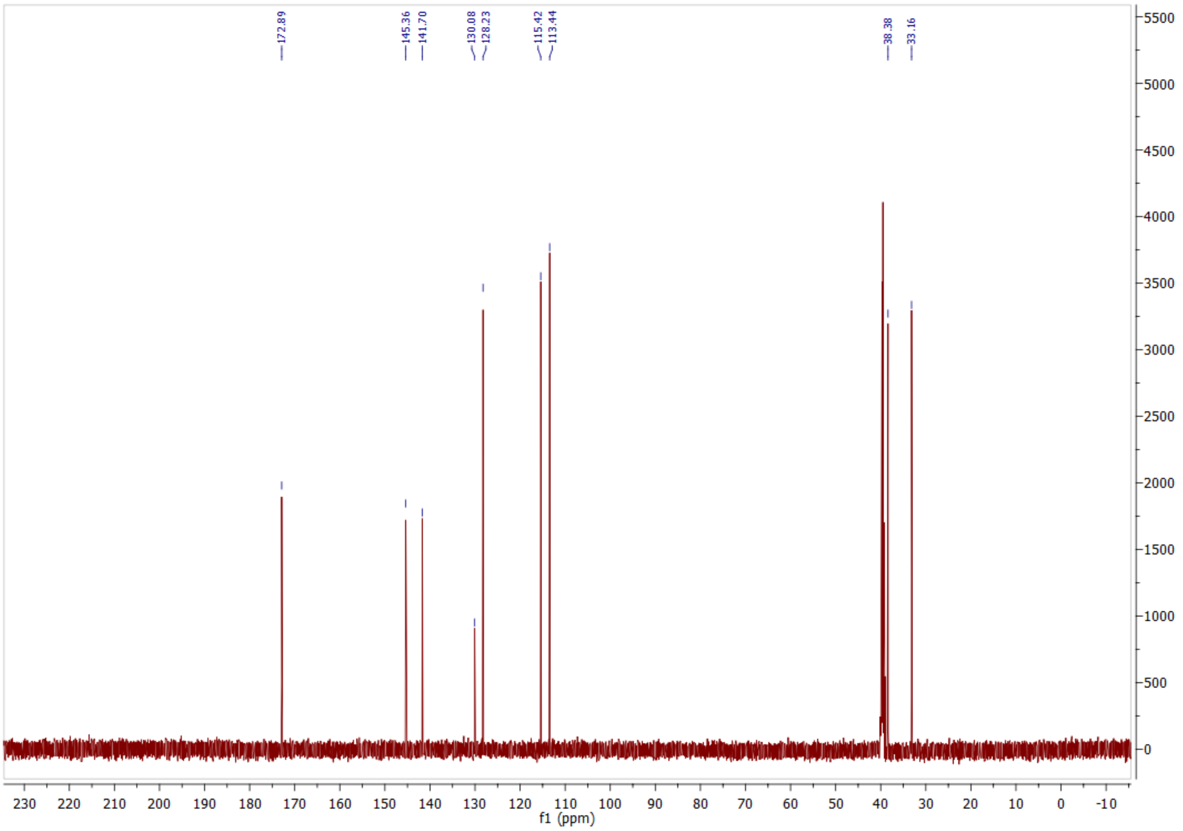

172.89
145.36
141.70
130.08
128.23
115.42
113.44
38.38
33.16
f1 (ppm)

3-[(5-Chloro-2-nitrophenyl)amino]-*N*-[(1*R*)-3-methyl-1-[(1*S*,2*S*,6*R*,8*S*)-2,9,9-trimethyl-3,5-dioxa-4-boratricyclo[6.1.1.0^{2,6}]decan-4-yl]butyl]propanamide (XXXVIII)

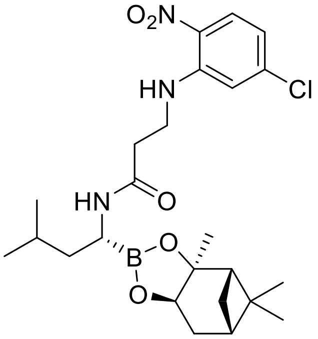

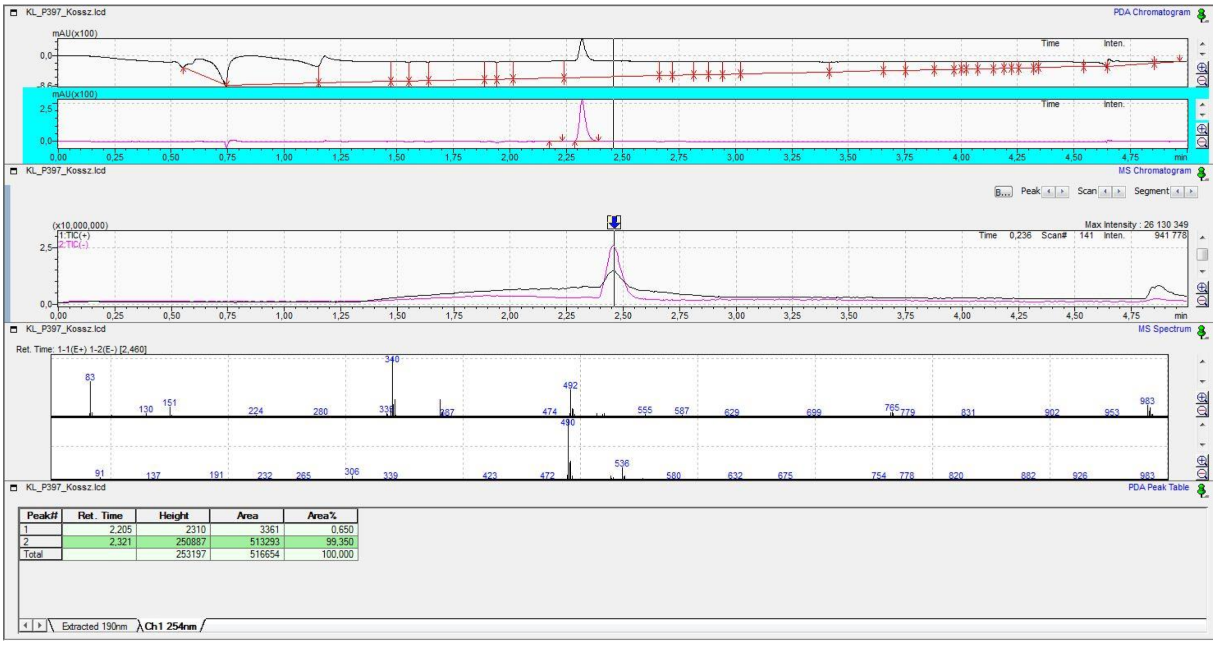

Peak#	Ret. Time	Height	Area	Area%
1	2,205	2310	3361	0,650
2	2,321	250887	513293	99,350
Total		253197	516654	100,000

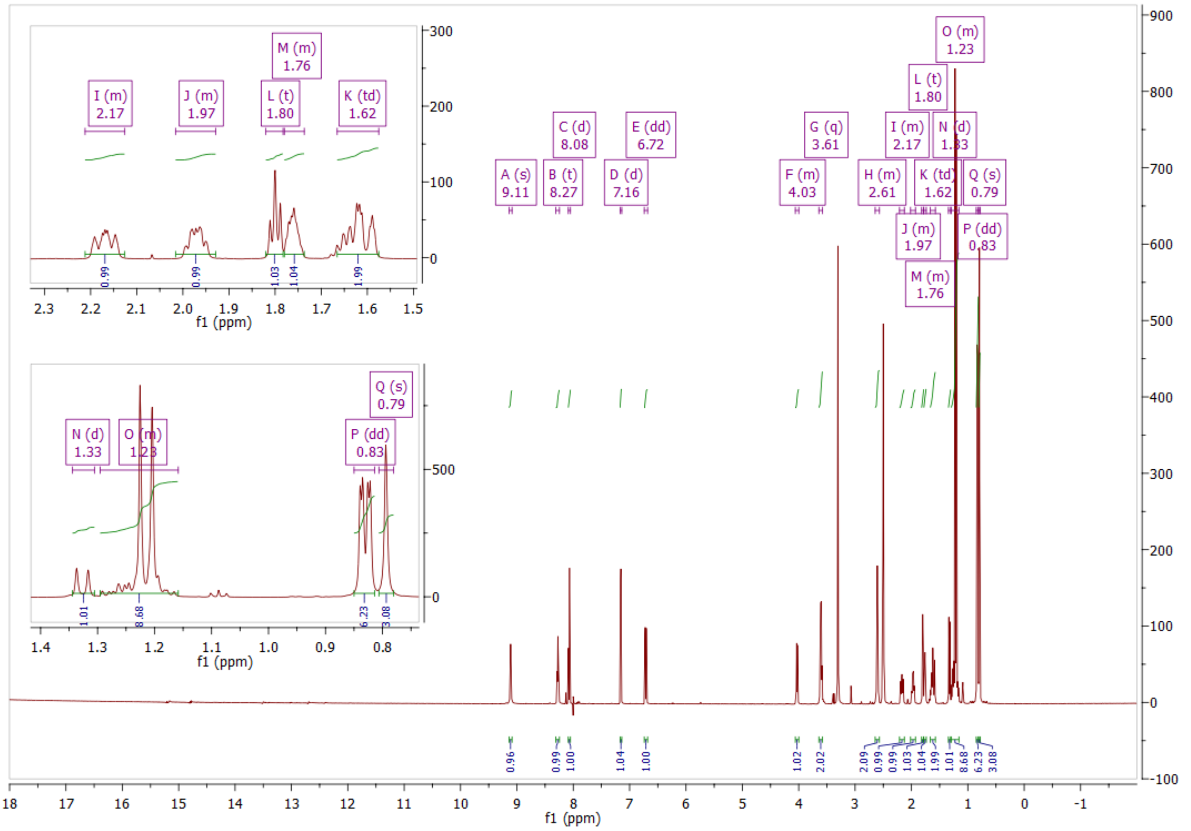
A (s) 9.11
B (t) 8.27
C (d) 8.08
D (d) 7.16
E (dd) 6.72
F (m) 4.03
G (q) 3.61
H (m) 2.61
I (m) 2.17
J (m) 1.97
K (td) 1.62
L (t) 1.80
M (m) 1.76
N (d) 1.33
O (m) 1.23
P (dd) 0.83
Q (s) 0.79
f1 (ppm)

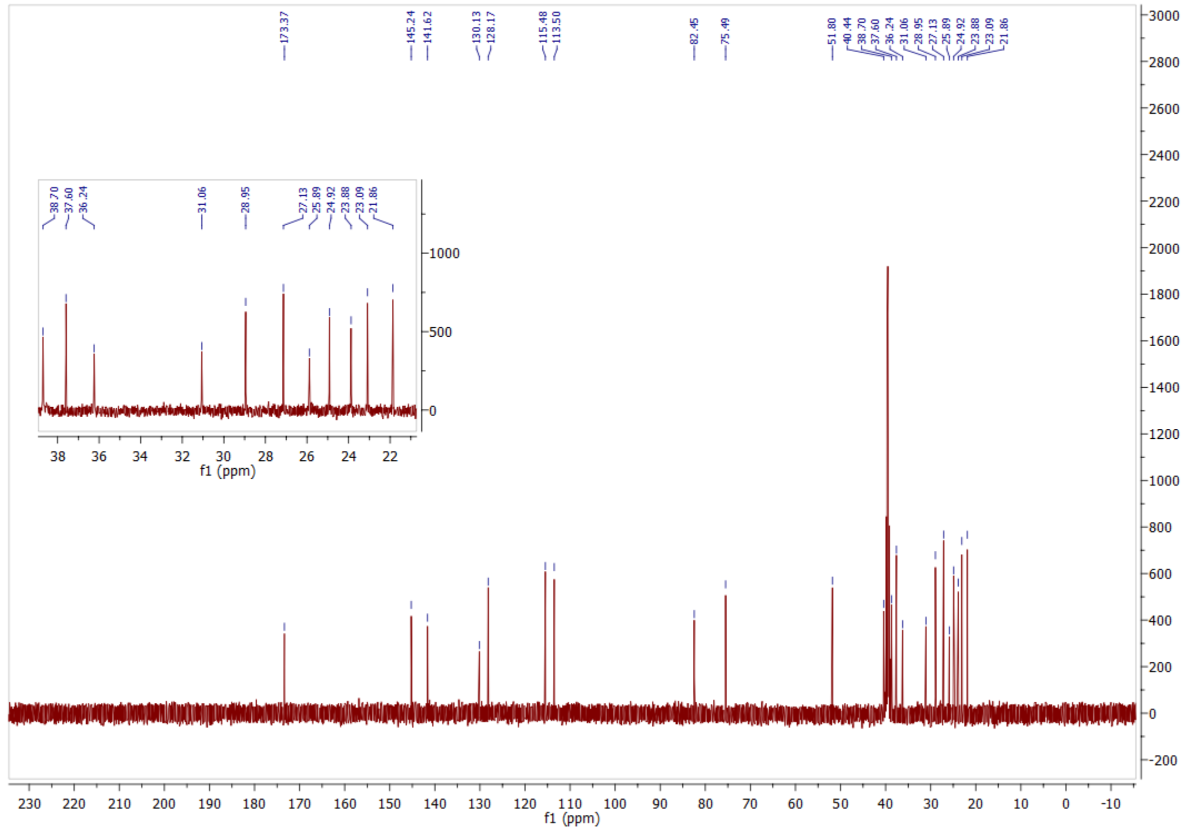
173.37
145.24
141.62
130.13
128.17
115.48
113.50
82.45
75.49
51.80
40.44
38.70
37.60
36.24
31.06
28.95
27.13
25.89
24.92
23.88
23.09
21.86
f1 (ppm)

3-[(2-Amino-5-chlorophenyl)amino]-*N*-[(1*R*)-3-methyl-1-[(1*S*,2*S*,6*R*,8*S*)-2,9,9-trimethyl-3,5-dioxa-4-boratricyclo[6.1.1.0^{2,6}]decan-4-yl]butyl]propanamide (XL)

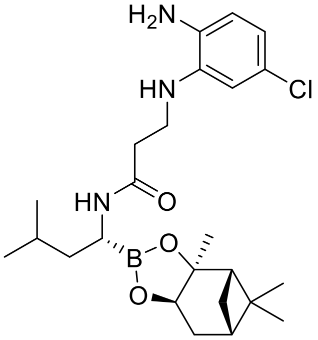

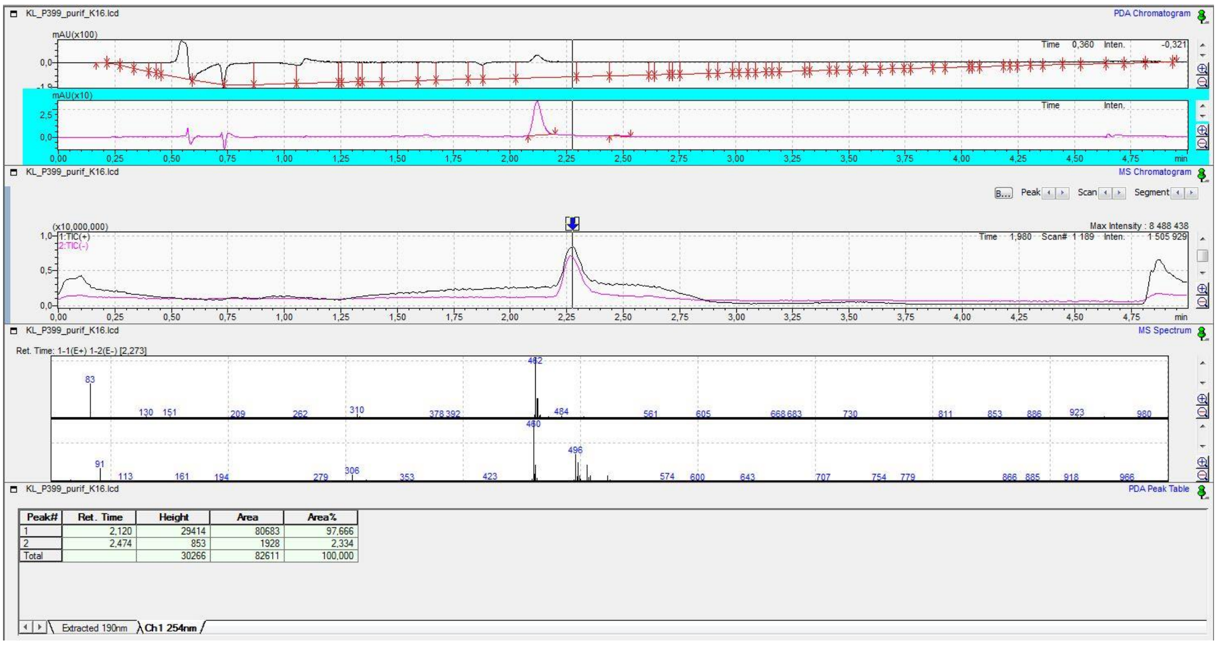

Peak#	Ret. Time	Height	Area	Area%
1	2,120	29414	80683	97,666
2	2,474	853	1928	2,334
Total		30266	82611	100,000

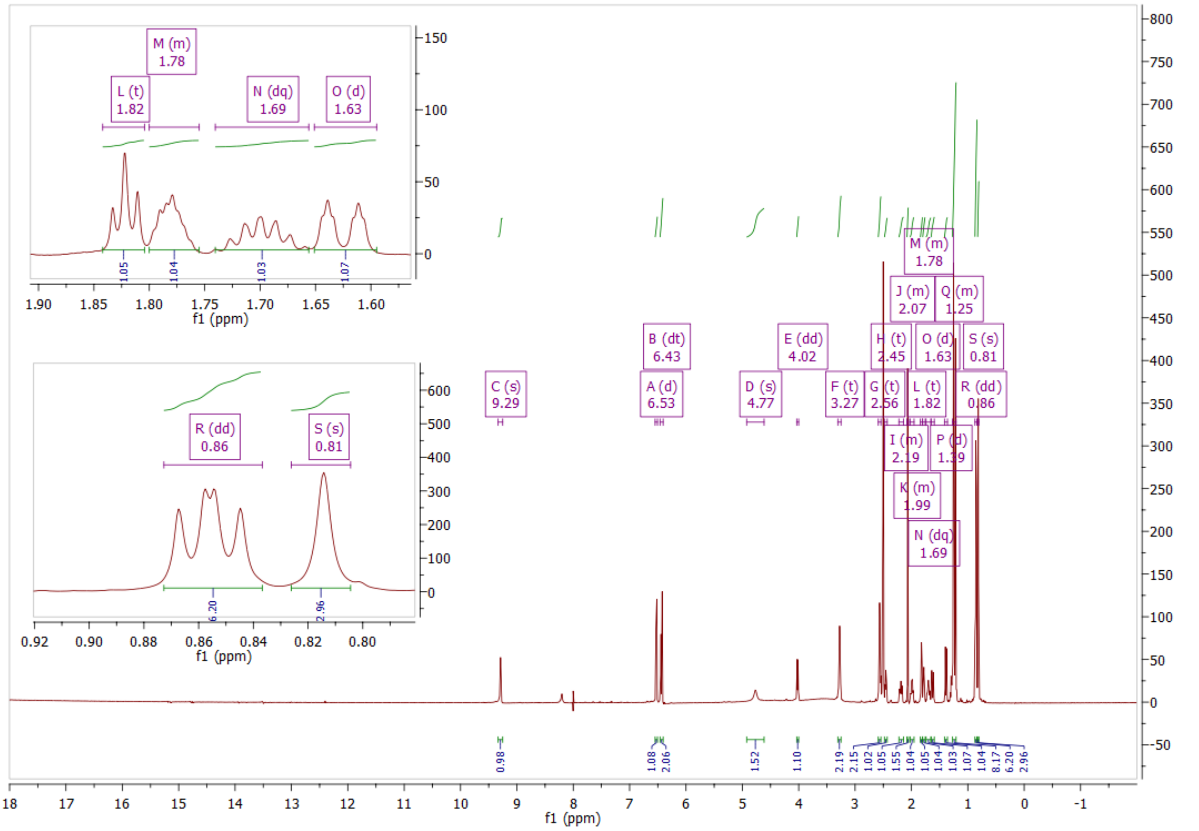
C (s) 9.29
B (dt) 6.43
A (d) 6.53
D (s) 4.77
E (dd) 4.02
F (t) 3.27
G (t) 2.56
H (t) 2.45
I (m) 2.19
J (m) 2.07
K (m) 1.99
L (t) 1.82
M (m) 1.78
N (dq) 1.69
O (d) 1.63
P (d) 1.39
Q (m) 1.25
R (dd) 0.86
S (s) 0.81
f1 (ppm)

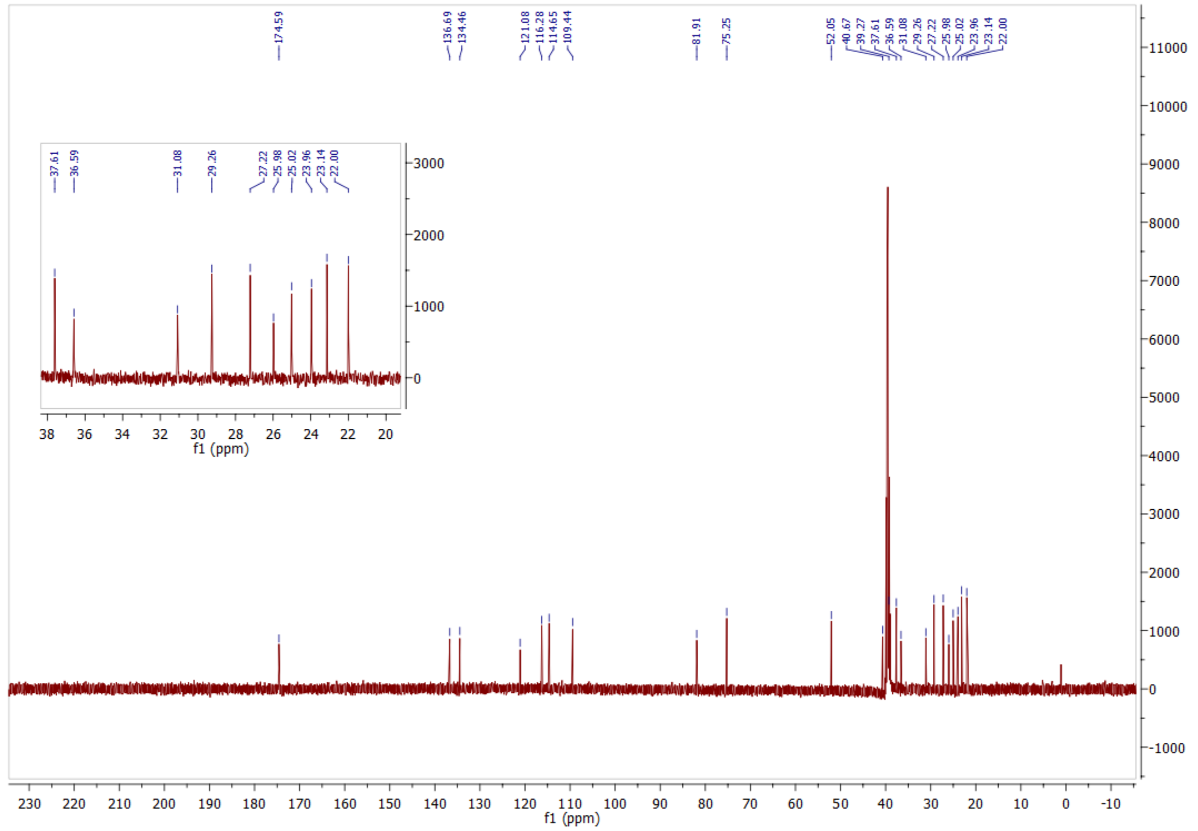
174.59
136.69
134.46
121.08
116.28
114.65
109.44
81.91
75.25
52.05
40.67
39.27
37.61
36.59
31.08
29.26
27.22
25.98
25.02
23.96
23.14
22.00
f1 (ppm)

3-(6-Chloro-2-cyano-1*H*-1,3-benzodiazol-1-yl)-*N*-[(1*R*)-3-methyl-1-[(1*S*,2*S*,6*R*,8*S*)-2,9,9-trimethyl-3,5-dioxa-4-boratricyclo[6.1.1.0^{2,6}]decan-4-yl]butyl]propanamide (37)

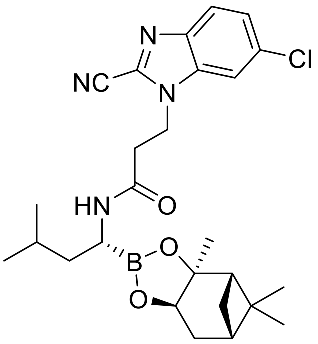

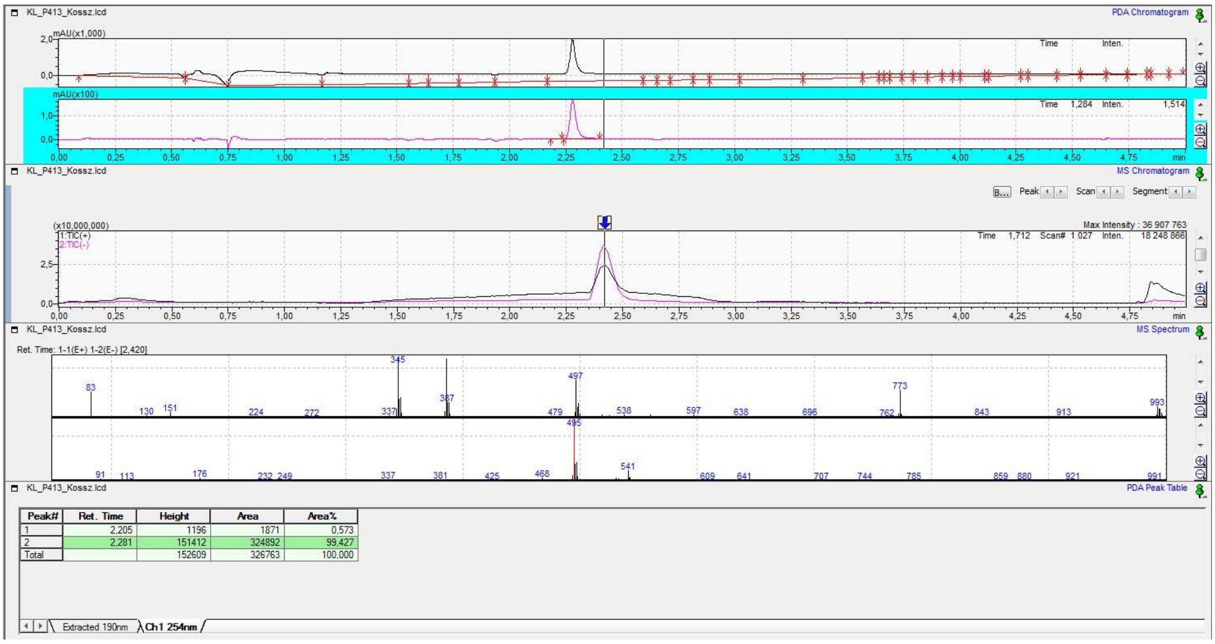

Peak#	Ret. Time	Height	Area	Area%
1	2,205	1196	1871	0,573
2	2,281	151412	324892	99,427
Total		152609	326763	100,000

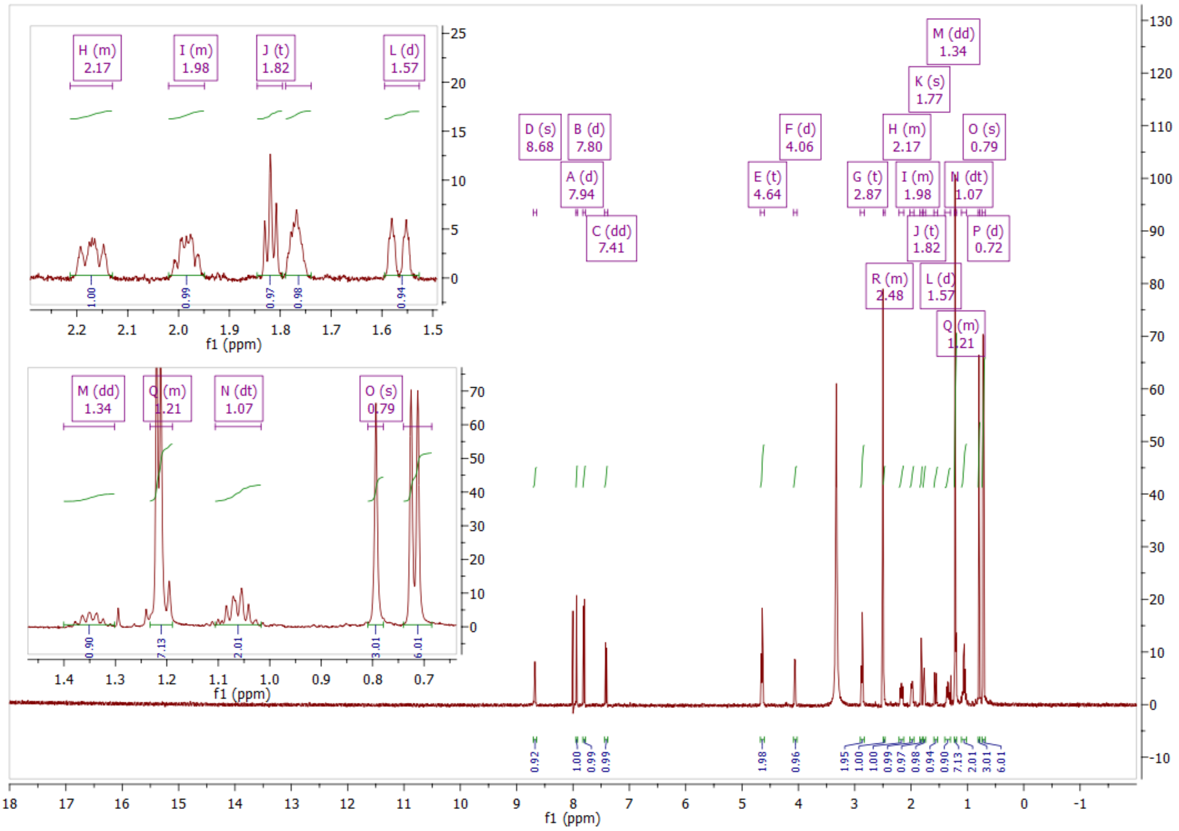
D (s) 8.68
B (d) 7.80
A (d) 7.94
C (dd) 7.41
E (t) 4.64
F (d) 4.06
G (t) 2.87
H (m) 2.17
I (m) 1.98
J (t) 1.82
K (s) 1.77
L (d) 1.57
M (dd) 1.34
N (dt) 1.07
O (s) 0.79
P (d) 0.72
Q (m) 1.21
R (m) 2.48
f1 (ppm)

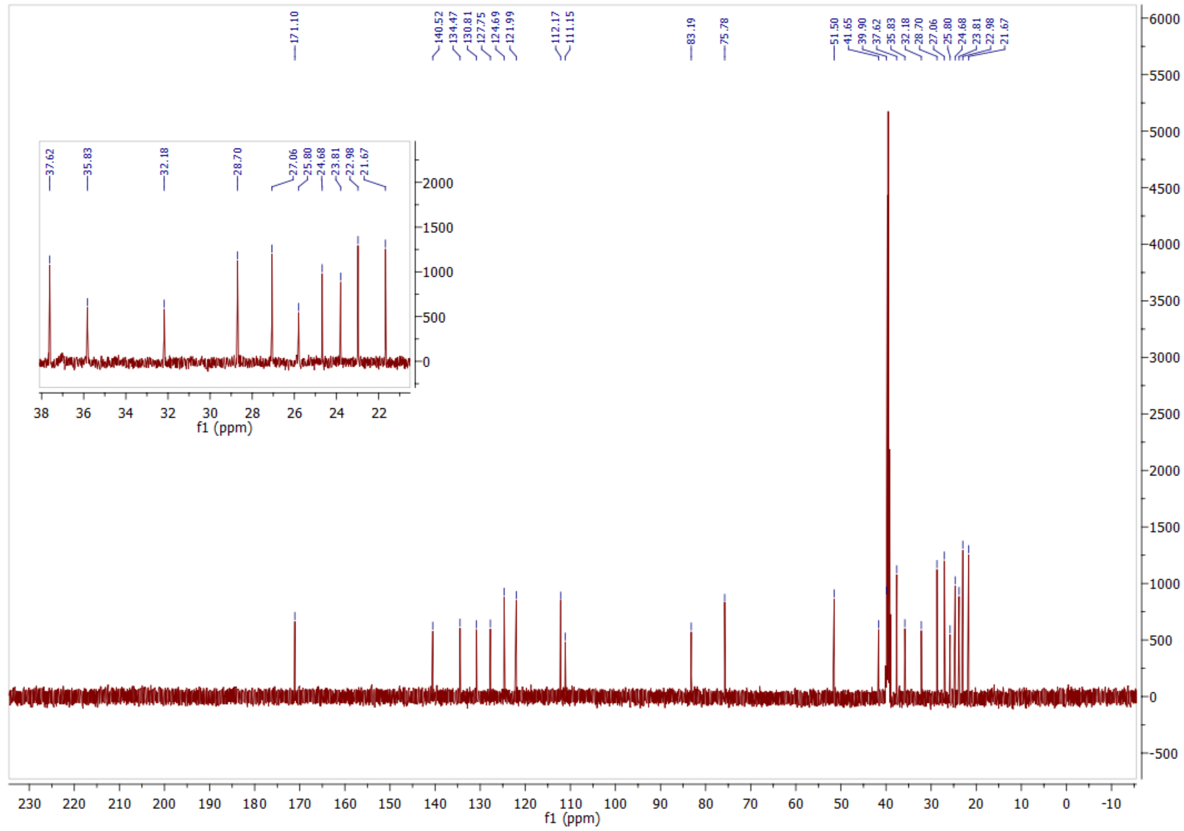
171.10
140.52
134.47
130.81
127.75
124.69
121.99
112.17
111.15
83.19
75.78
51.50
41.65
39.90
37.62
35.83
32.18
28.70
27.06
25.80
24.68
23.81
22.98
21.67
f1 (ppm)

3-[(2-Chloro-6-nitrophenyl)amino]propanoic acid (XXXVII)

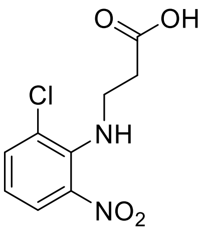

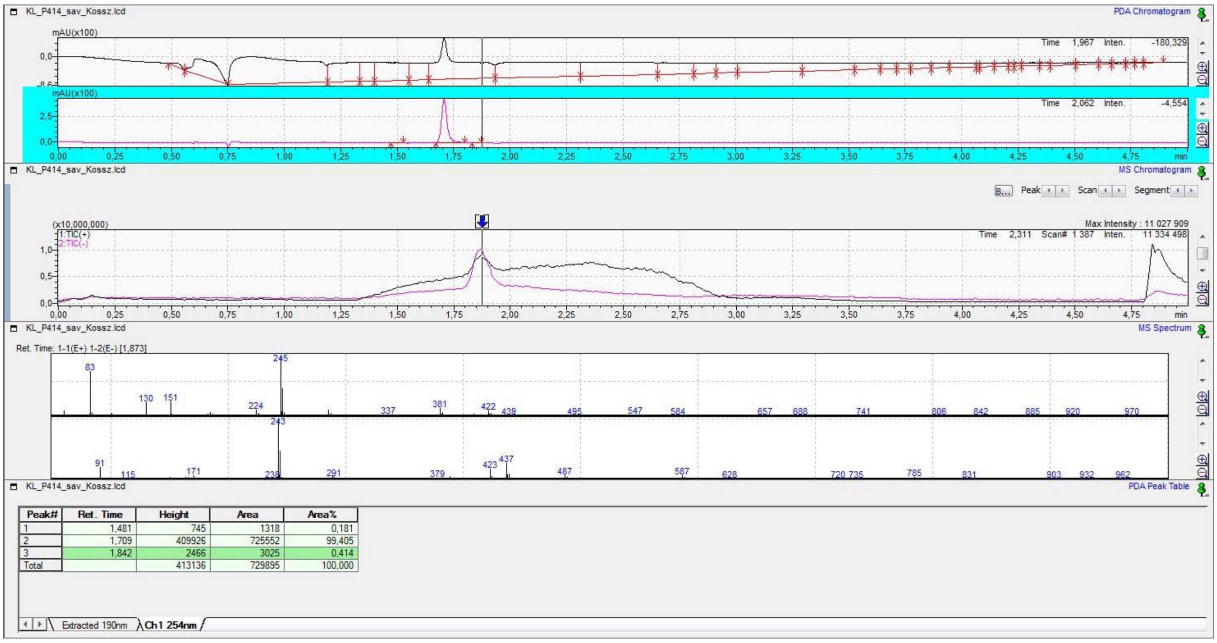

Peak#	Ret. Time	Height	Area	Area%
1	1,481	745	1318	0,181
2	1,709	409926	725552	99,405
3	1,842	2466	3025	0,414
Total		413136	729895	100,000

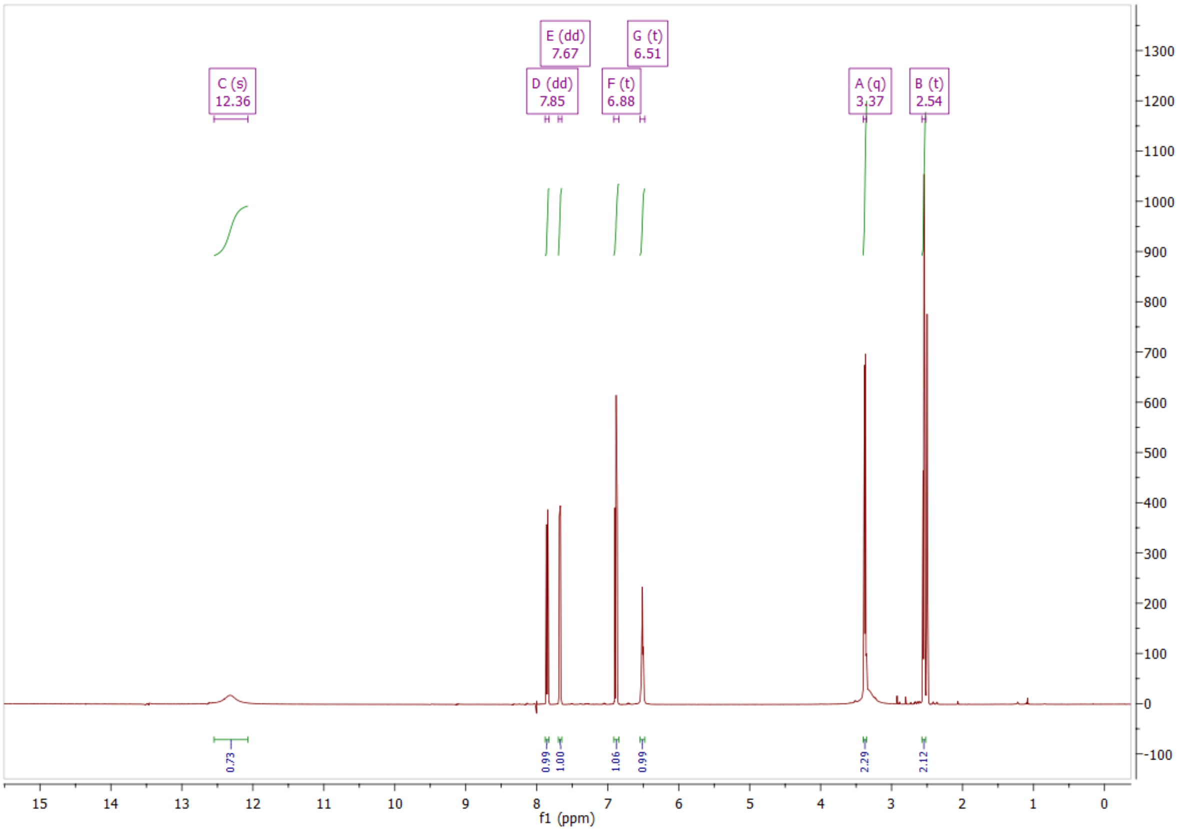

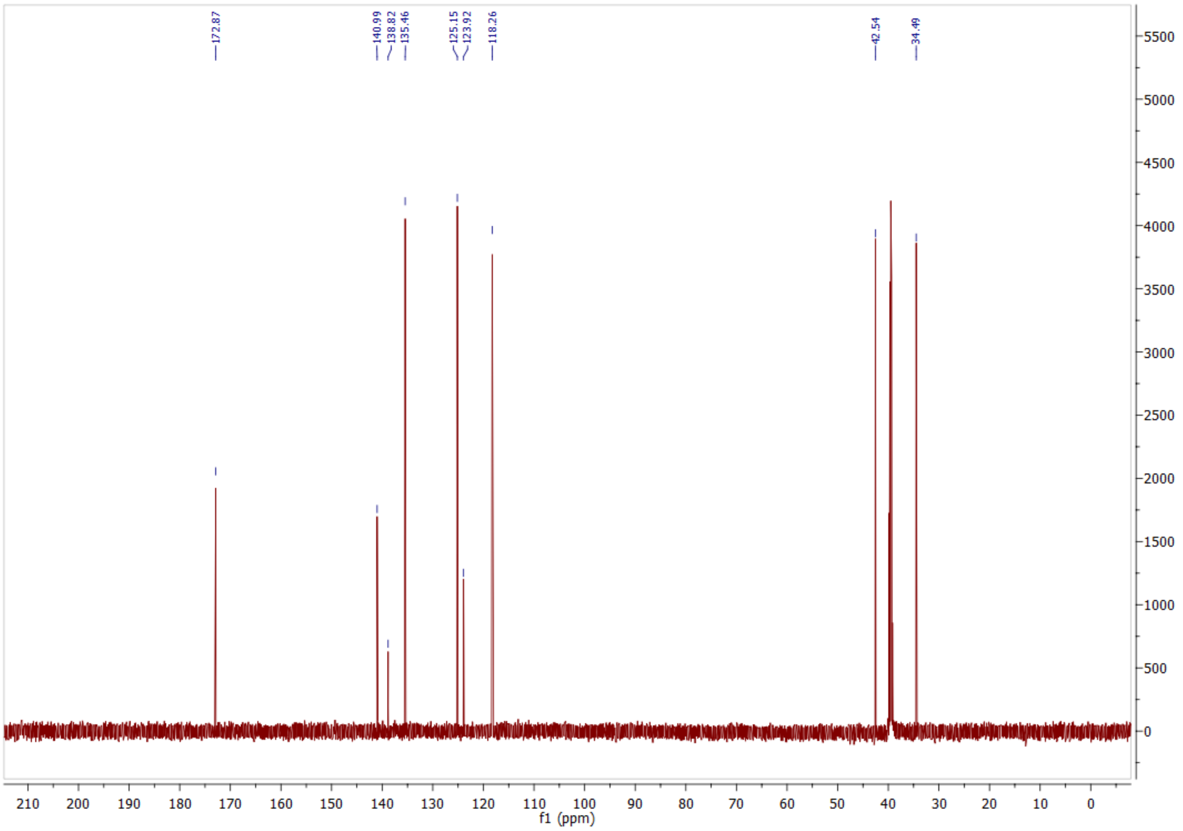

3-[(2-Chloro-6-nitrophenyl)amino]-*N*-[(1*R*)-3-methyl-1-[(1*S*,2*S*,6*R*,8*S*)-2,9,9-trimethyl-3,5-dioxa-4-boratricyclo[6.1.1.0^{2,6}]decan-4-yl]butyl]propanamide (XXXIX)

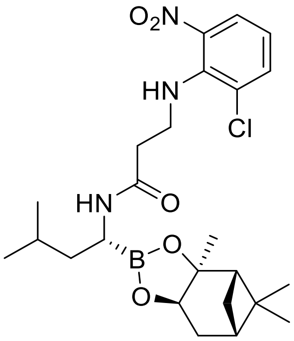

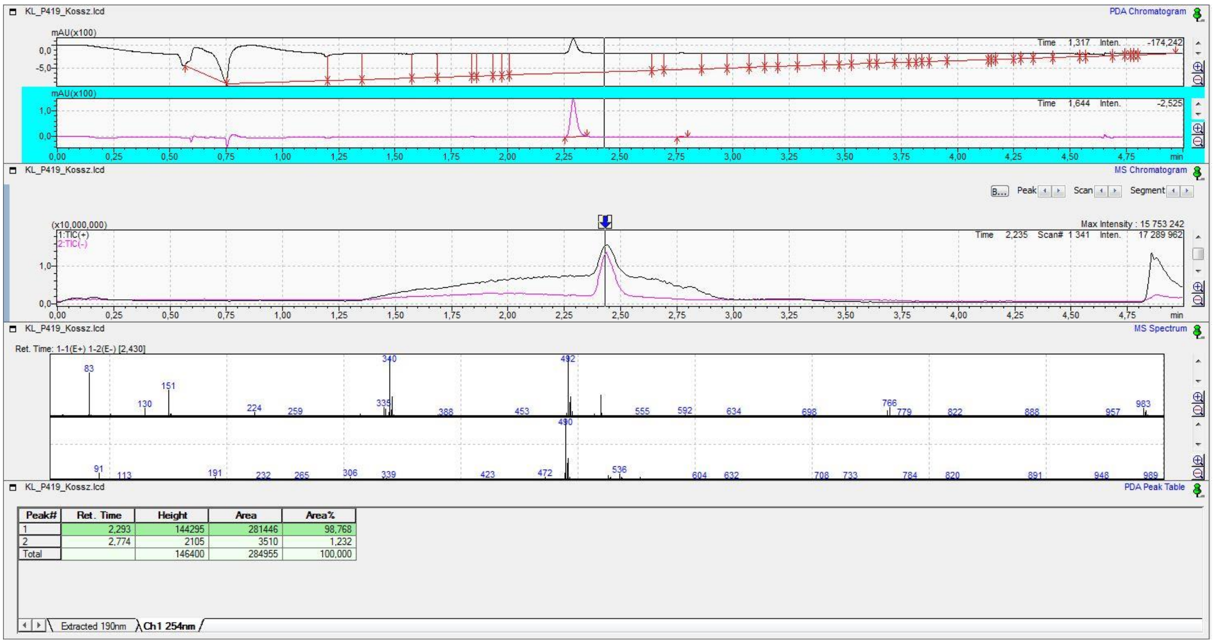
KL_P419_Kossz.lcd
PDA Chromatogram
MS Chromatogram
MS Spectrum
PDA Peak Table
Ret. Time: 1-1(E+) 1-2(E-) [2,430]
Max Intensity : 15 753 242
Peak# | Ret. Time | Height | Area | Area%
1 | 2,293 | 144295 | 281446 | 98,768
2 | 2,774 | 2105 | 3510 | 1,232
Total | | 146400 | 284955 | 100,000
Extracted 190nm
Ch1 254nm

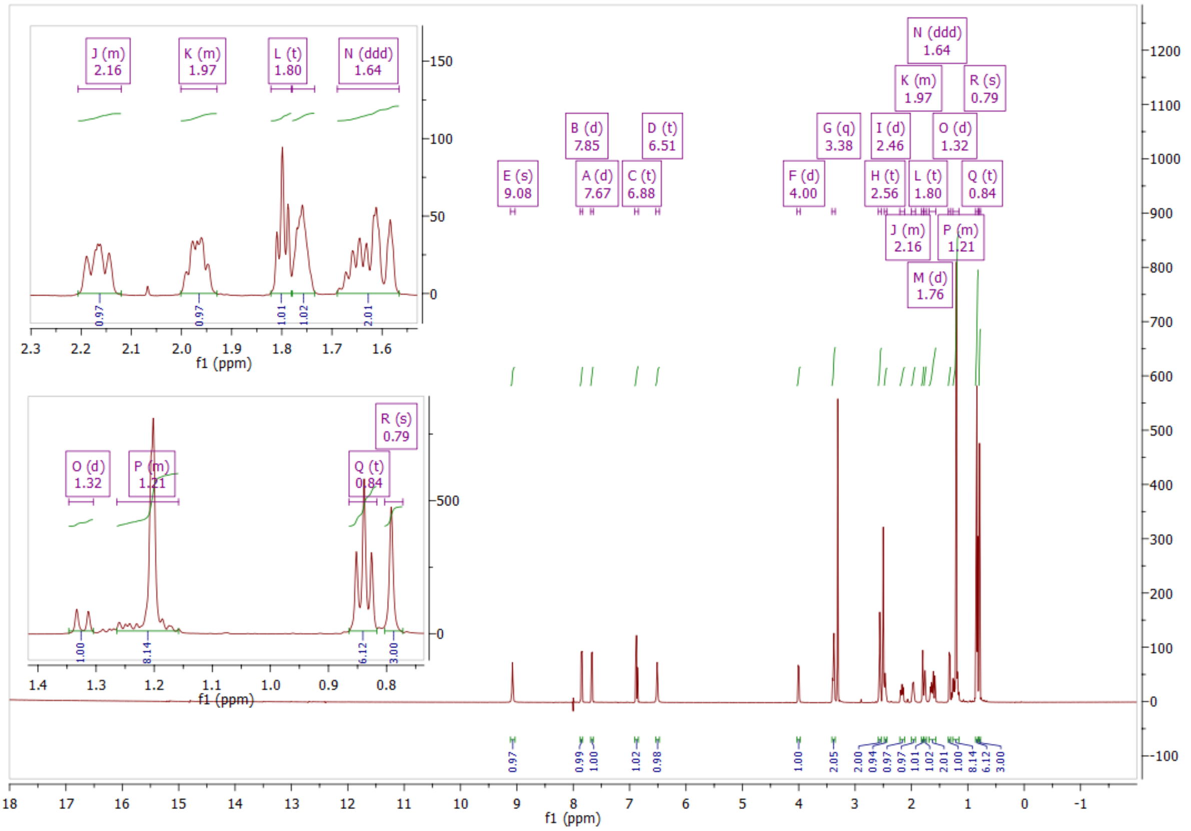
A (d) 7.67
B (d) 7.85
C (t) 6.88
D (t) 6.51
E (s) 9.08
F (d) 4.00
G (q) 3.38
H (t) 2.56
I (d) 2.46
J (m) 2.16
K (m) 1.97
L (t) 1.80
M (d) 1.76
N (ddd) 1.64
O (d) 1.32
P (m) 1.21
Q (t) 0.84
R (s) 0.79
f1 (ppm)

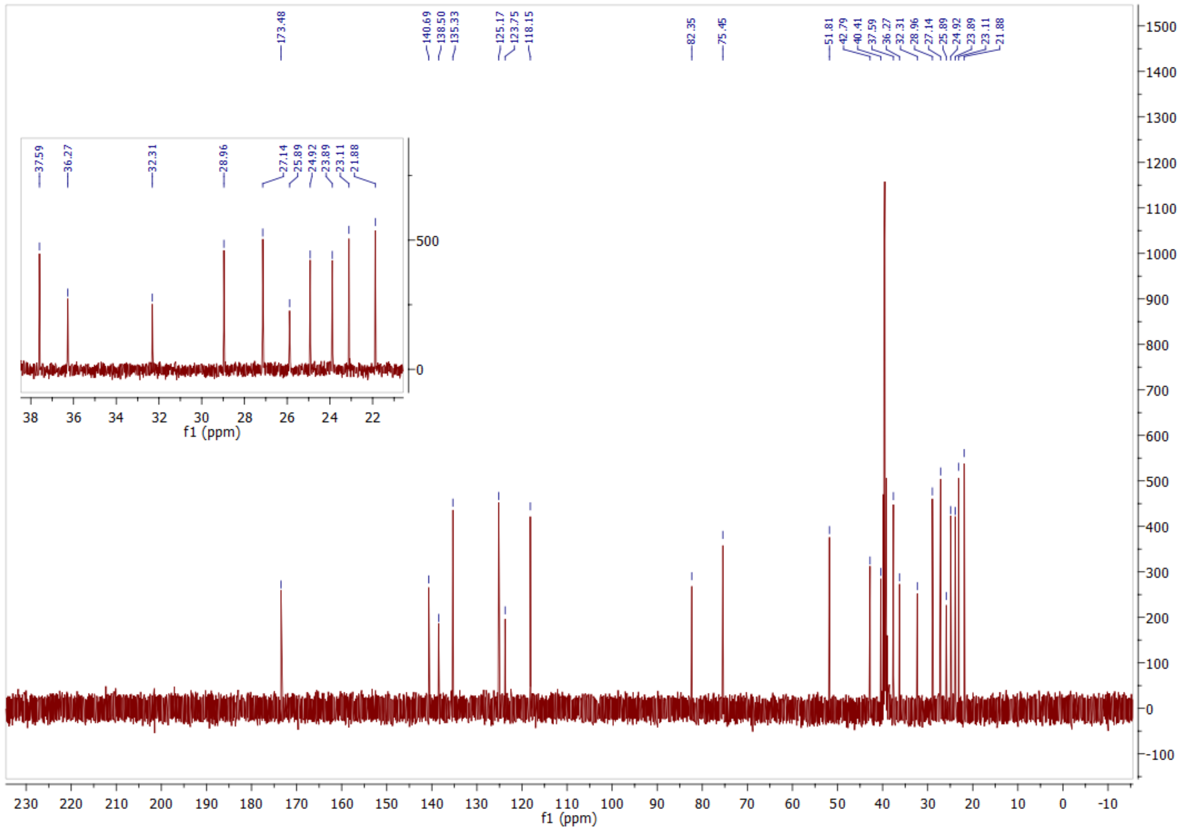

3-[(2-Amino-6-chlorophenyl)amino]-*N*-[(1*R*)-3-methyl-1-[(1*S*,2*S*,6*R*,8*S*)-2,9,9-trimethyl-3,5-dioxa-4-boratricyclo[6.1.1.$0^{2,6}$]decan-4-yl]butyl]propanamide (XLI)

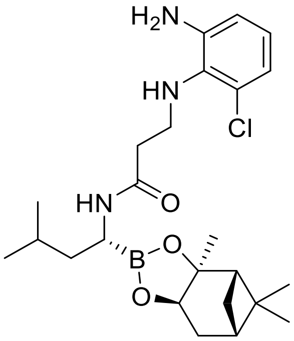

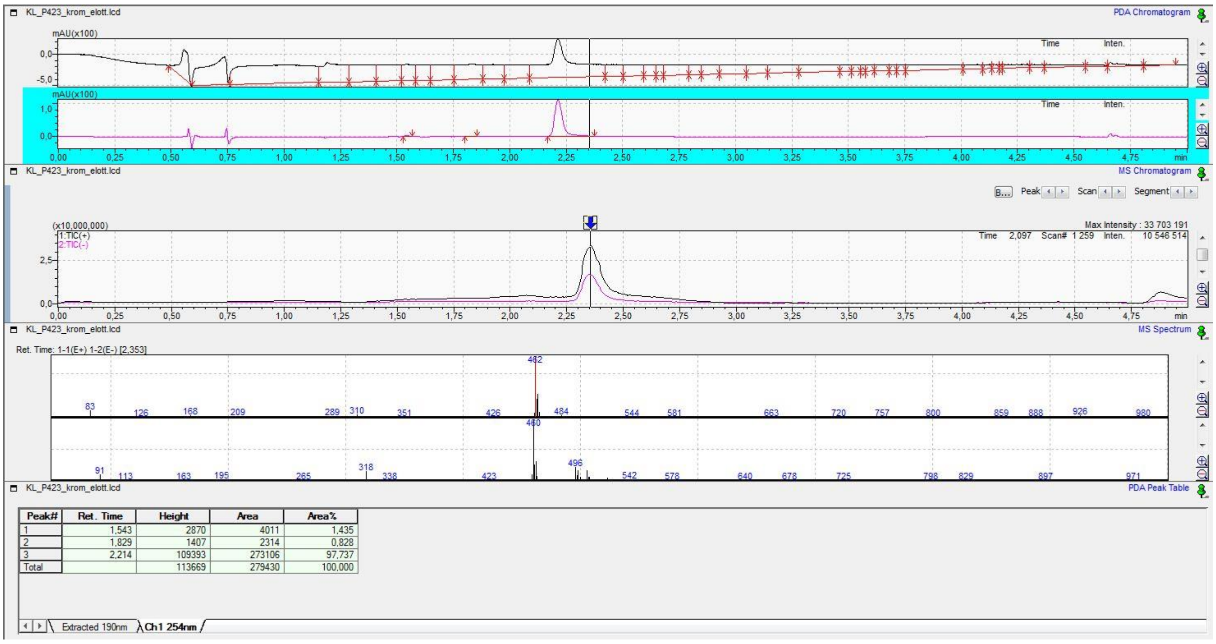

Peak#	Ret. Time	Height	Area	Area%
1	1,543	2870	4011	1,435
2	1,829	1407	2314	0,828
3	2,214	109393	273106	97,737
Total		113669	279430	100,000

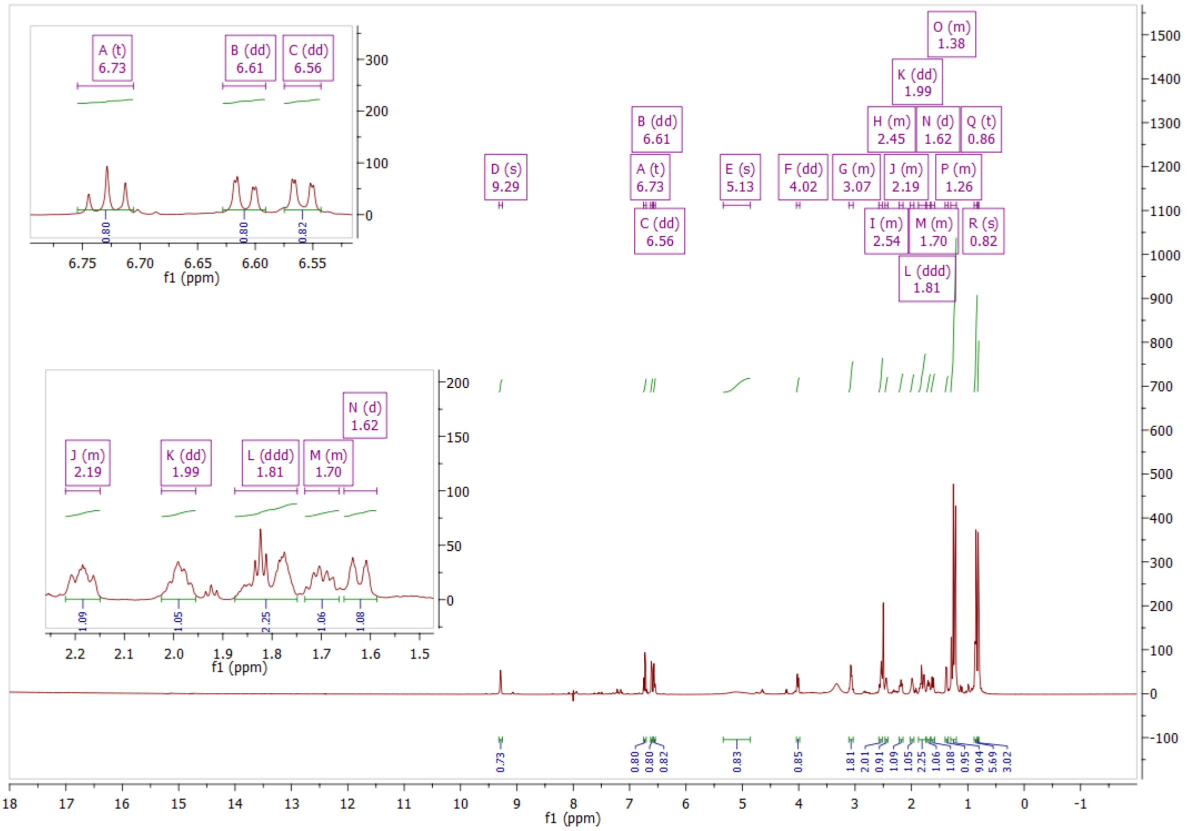

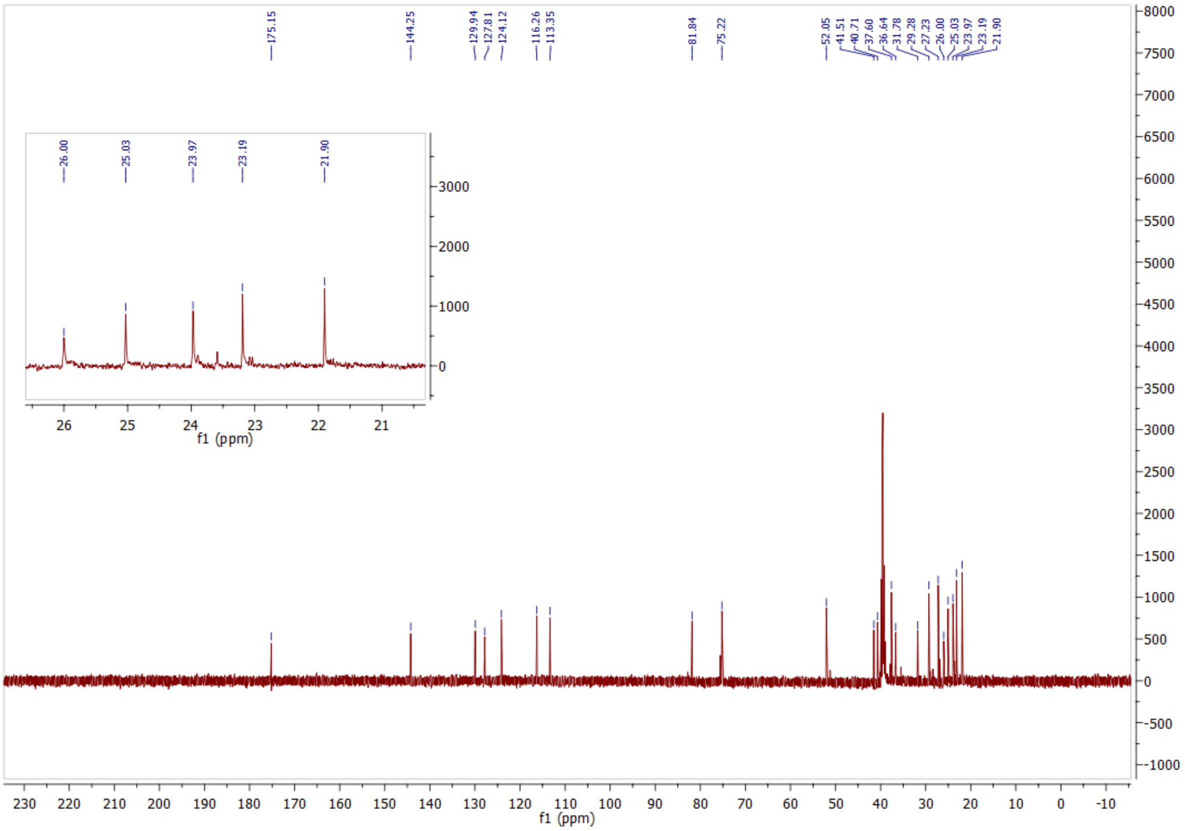

3-(7-Chloro-2-cyano-1*H*-1,3-benzodiazol-1-yl)-*N*-[(1*R*)-3-methyl-1-[(1*S*,2*S*,6*R*,8*S*)-2,9,9-trimethyl-3,5-dioxa-4-boratricyclo[6.1.1.0^{2,6}]decan-4-yl]butyl]propanamide (38)

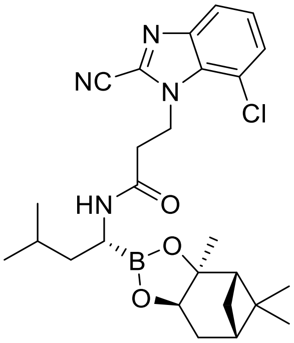

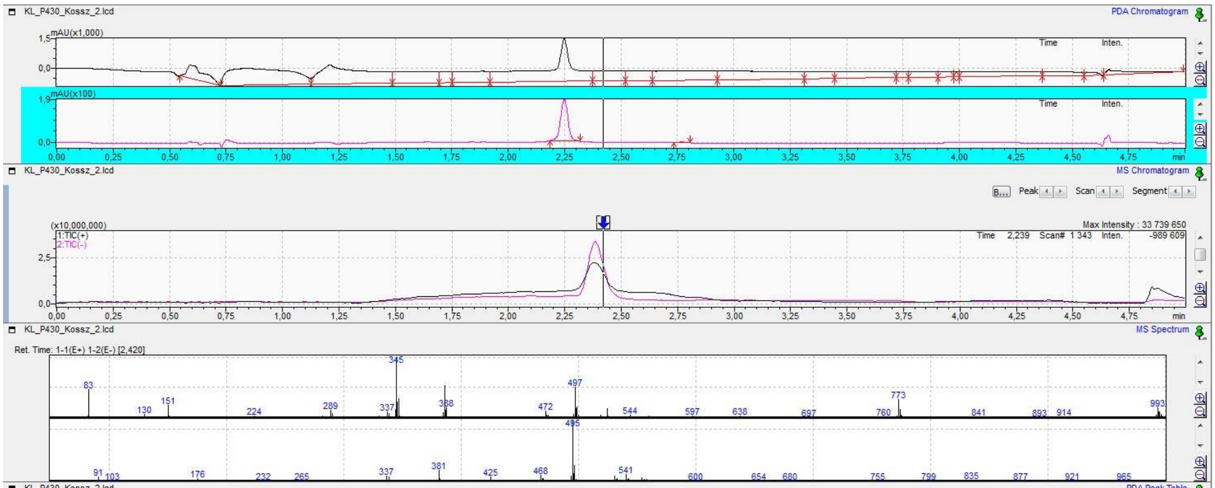

KL_P430_Kossz_2.lcd

PDA Peak Table

Peak#	Ret. Time	Height	Area	Area%
1	2,250	181514	433418	98,154
2	2,772	3624	8149	1,846
Total		185138	441567	100,000

Extracted 190nm | Ch1 254nm

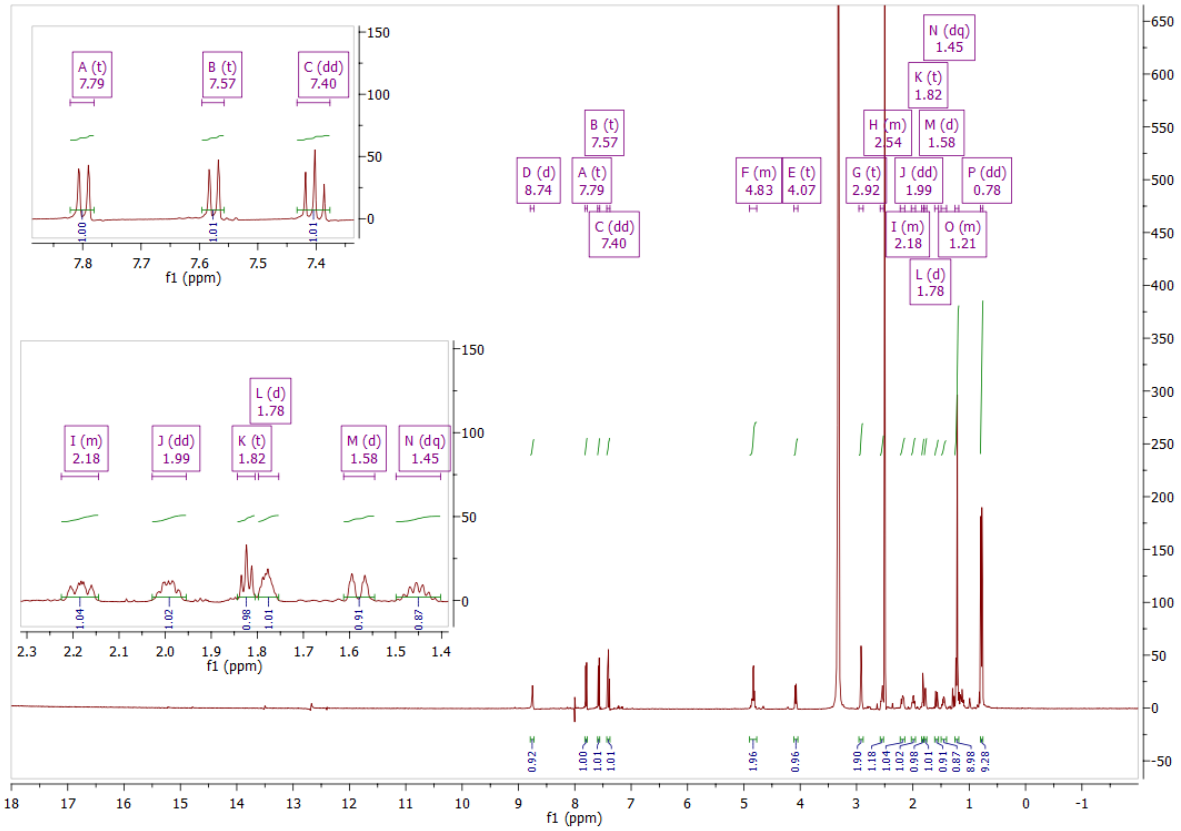

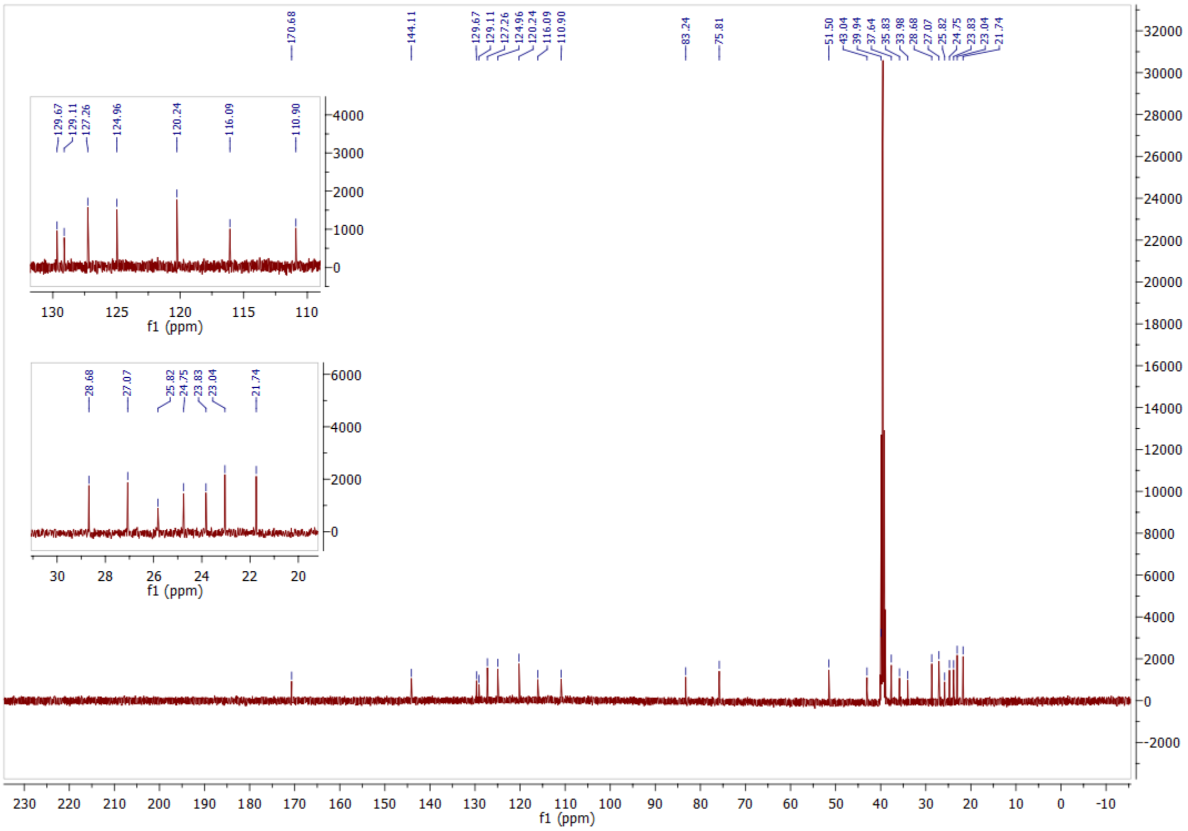

2-(3-Amino-4-hydroxyphenyl)acetic acid hydrochloride (XLIII)

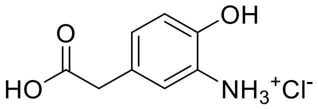

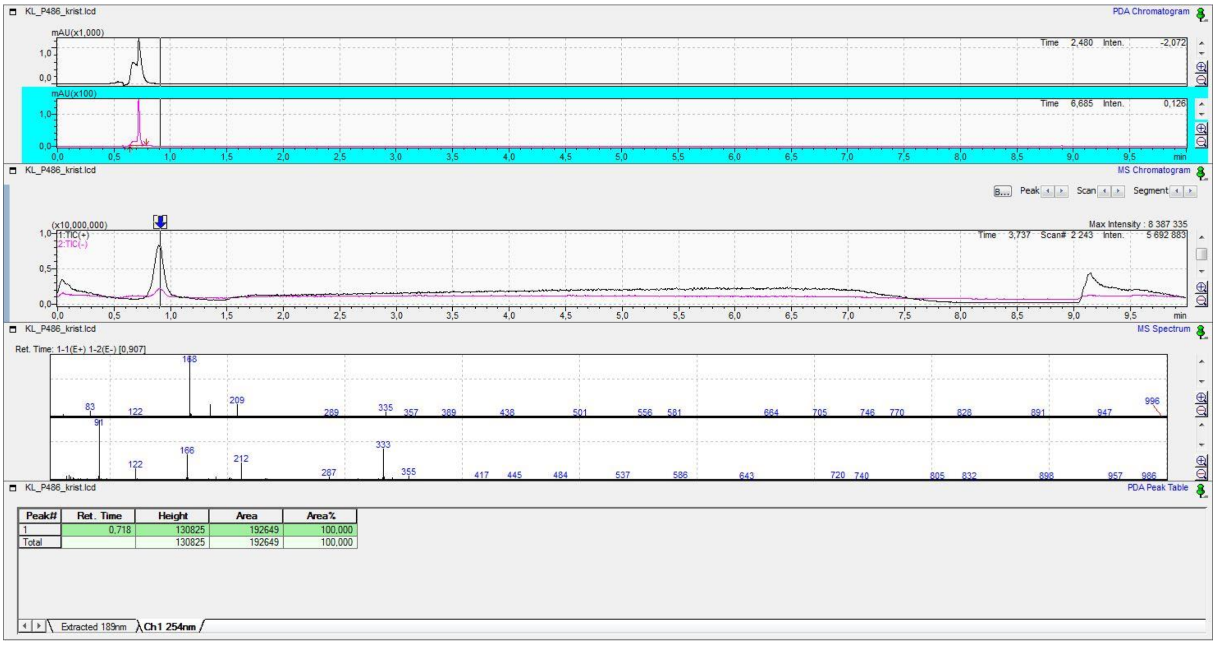

Peak#	Ret. Time	Height	Area	Area%
1	0,718	130825	192649	100,000
Total		130825	192649	100,000

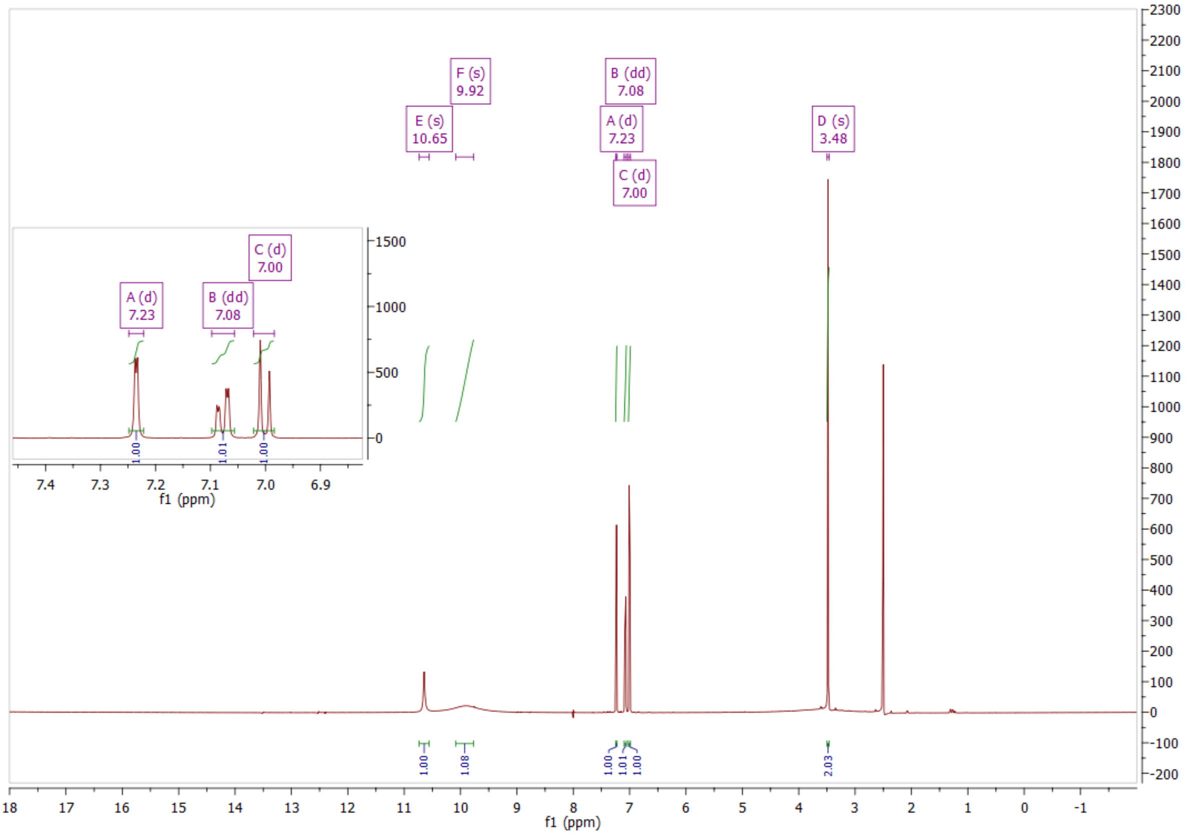
F (s)
9.92
E (s)
10.65
B (dd)
7.08
A (d)
7.23
C (d)
7.00
D (s)
3.48
1.00
1.08
1.00
1.01
1.00
2.03
f1 (ppm)

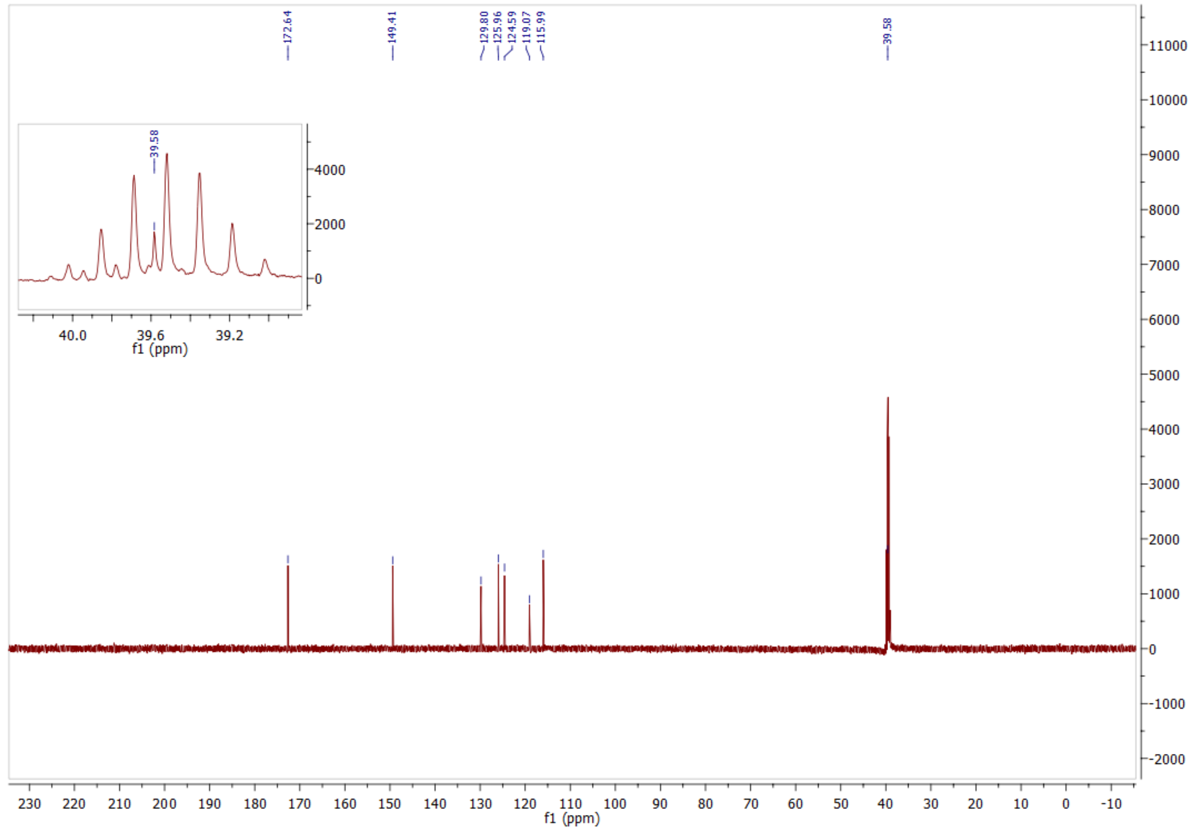
172.64
149.41
129.80
125.96
124.59
119.07
115.99
39.58
f1 (ppm)

2-(3-Amino-4-hydroxyphenyl)-*N*-[(1*R*)-3-methyl-1-[(1*S*,2*S*,6*R*,8*S*)-2,9,9-trimethyl-3,5-dioxa-4-boratricyclo[6.1.1.0^{2,6}]decan-4-yl]butyl]acetamide (XLIV)

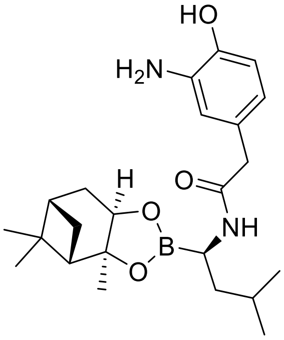

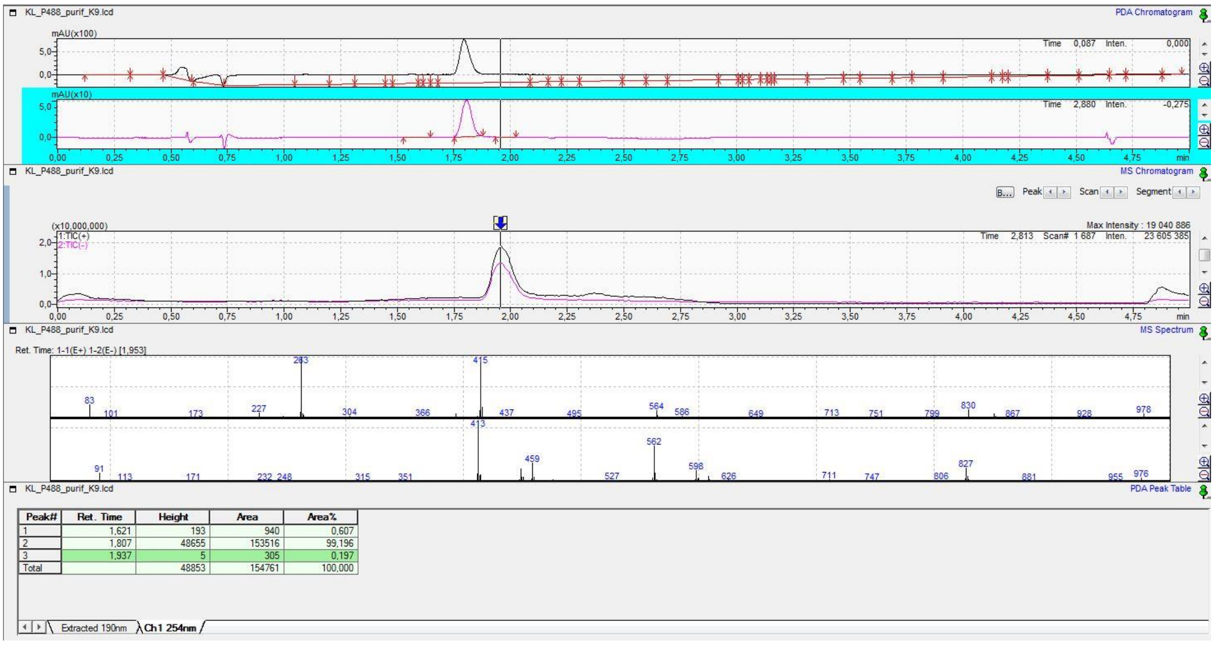

Peak#	Ret. Time	Height	Area	Area%
1	1,621	193	940	0,607
2	1,807	48655	153516	99,196
3	1,937	5	305	0,197
Total		48853	154761	100,000

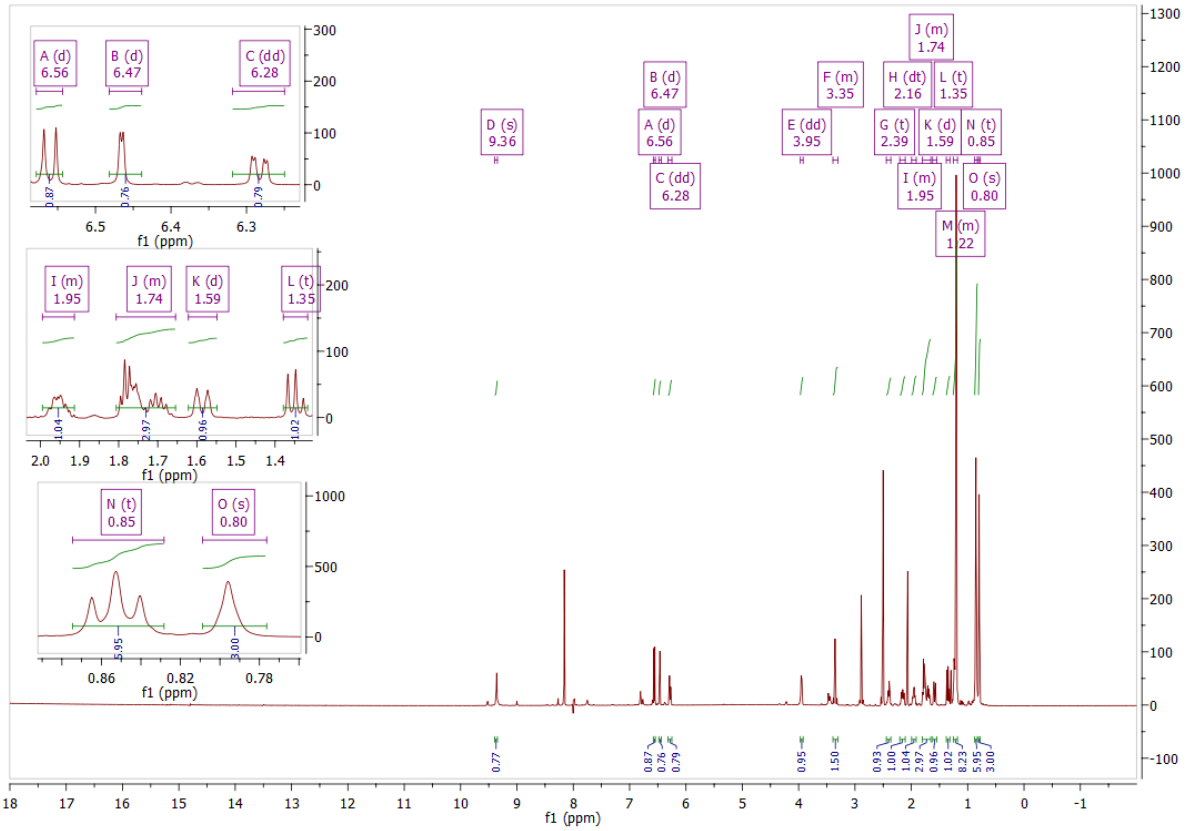
A (d) 6.56
B (d) 6.47
C (dd) 6.28
D (s) 9.36
E (dd) 3.95
F (m) 3.35
G (t) 2.39
H (dt) 2.16
I (m) 1.95
J (m) 1.74
K (d) 1.59
L (t) 1.35
M (m) 1.22
N (t) 0.85
O (s) 0.80
f1 (ppm)

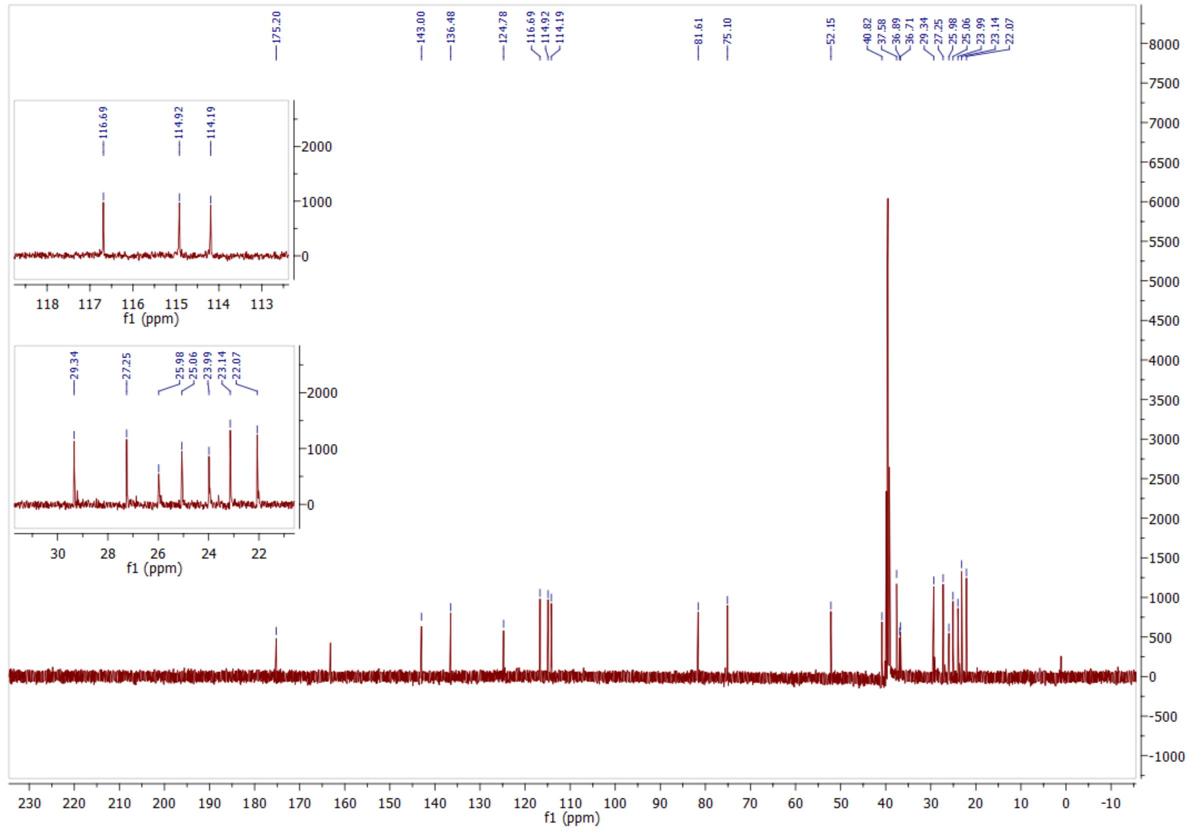
175.20
143.00
136.48
124.78
116.69
114.92
114.19
81.61
75.10
52.15
40.82
37.58
36.89
36.71
29.34
27.25
25.98
25.06
23.99
23.14
22.07
f1 (ppm)

2-(2-Cyano-1,3-benzoxazol-5-yl)-*N*-[(1*R*)-3-methyl-1-[(1*S*,2*S*,6*R*,8*S*)-2,9,9-trimethyl-3,5-dioxa-4-boratricyclo[6.1.1.0^2,6^]decan-4-yl]butyl]acetamide (39)

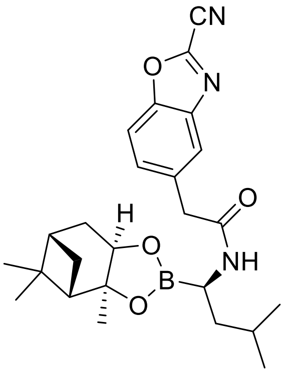

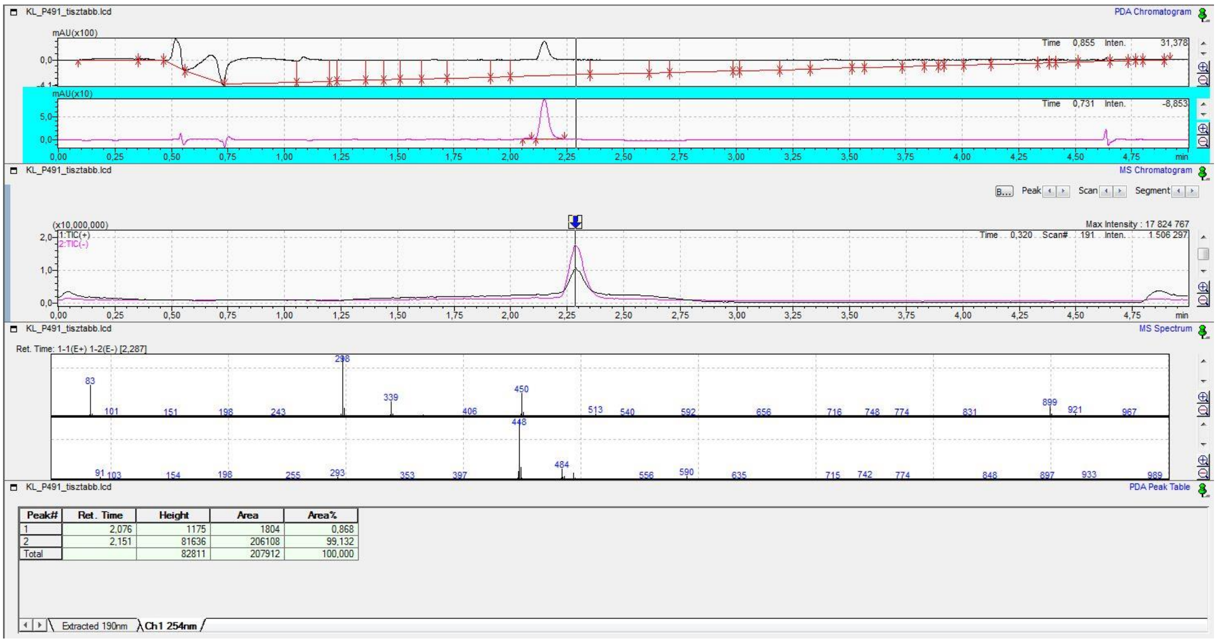

Peak#	Ret. Time	Height	Area	Area%
1	2,076	1175	1804	0,868
2	2,151	81636	206108	99,132
Total		82811	207912	100,000

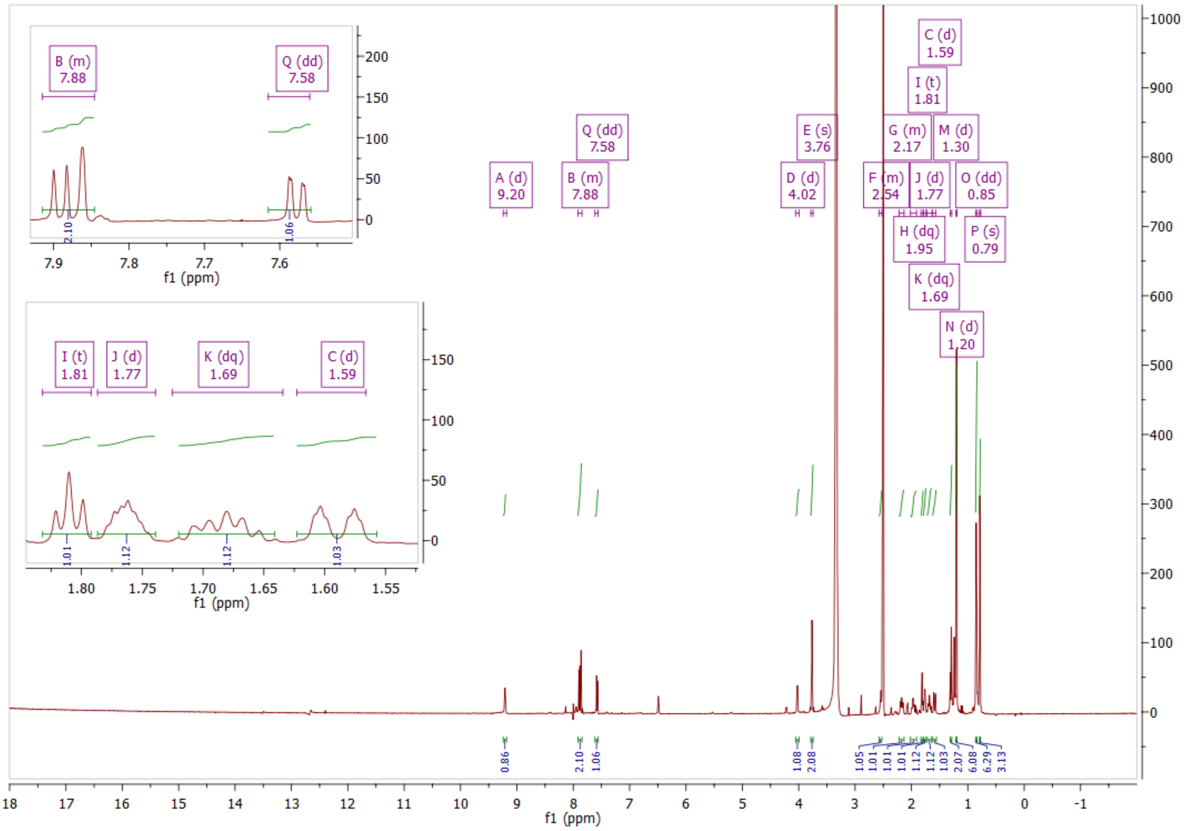

A (d) 9.20
B (m) 7.88
Q (dd) 7.58
D (d) 4.02
E (s) 3.76
F (m) 2.54
G (m) 2.17
H (dq) 1.95
I (t) 1.81
J (d) 1.77
K (dq) 1.69
C (d) 1.59
M (d) 1.30
N (d) 1.20
O (dd) 0.85
P (s) 0.79
f1 (ppm)

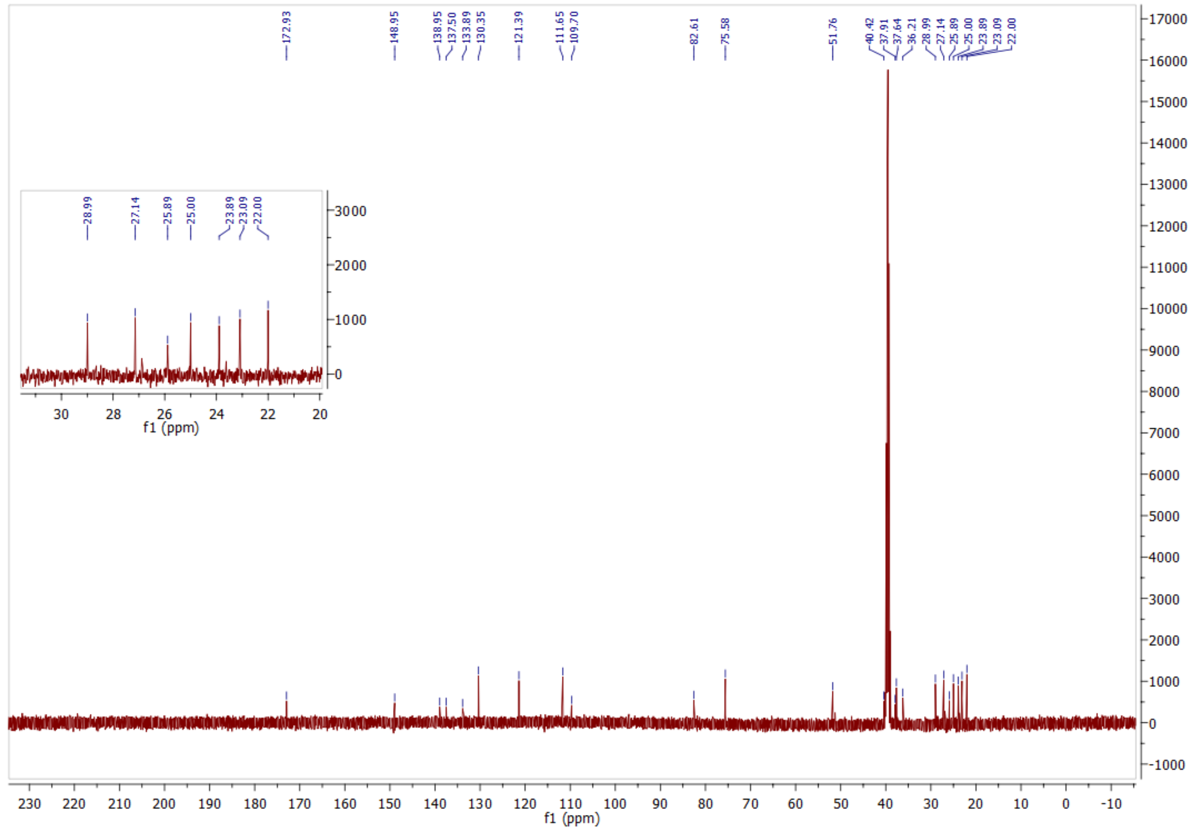

172.93
148.95
138.95
137.50
133.89
130.35
121.39
111.65
109.70
82.61
75.58
51.76
40.42
37.91
37.64
36.21
28.99
27.14
25.89
25.00
23.89
23.09
22.00
f1 (ppm)

[(1*R*)-1-[2-(2-Cyano-1,3-benzoxazol-5-yl)acetamido]-3-methylbutyl]boronic acid (40)

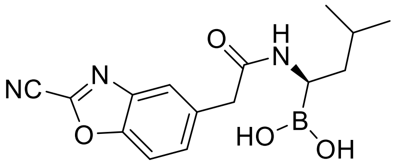

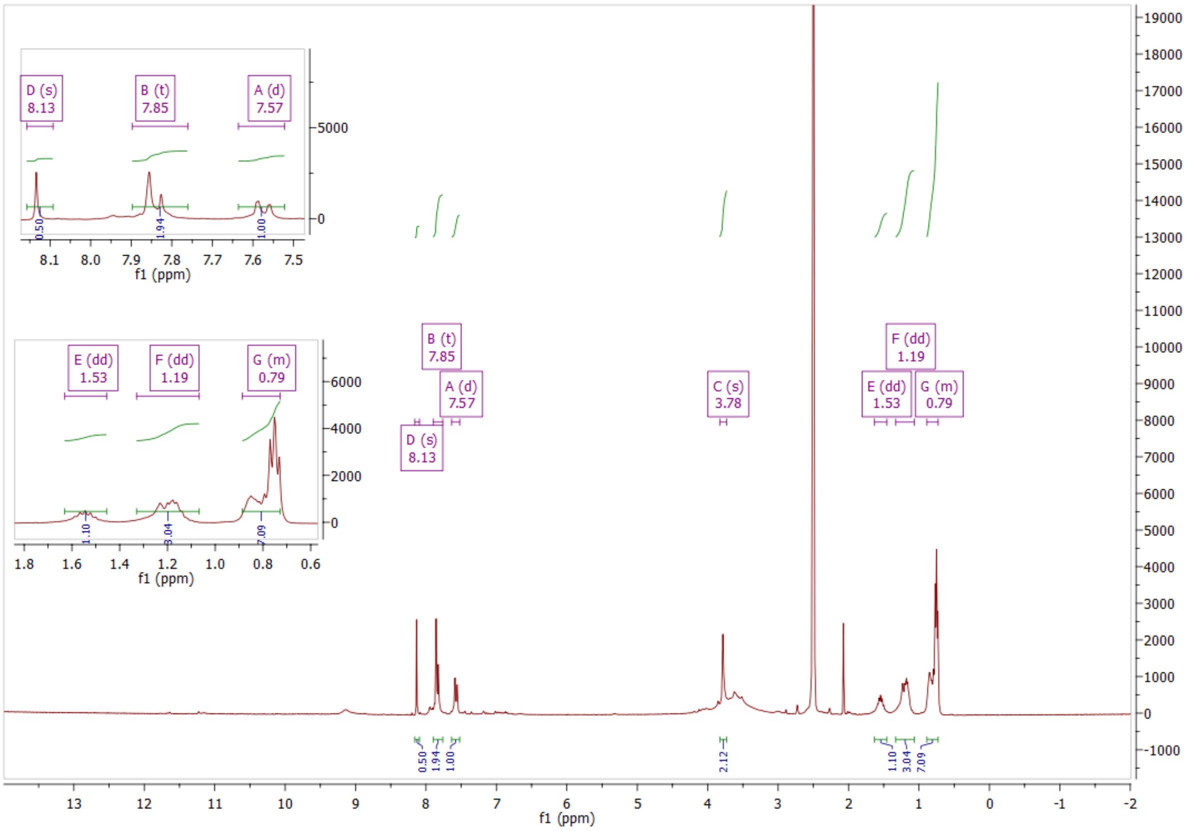

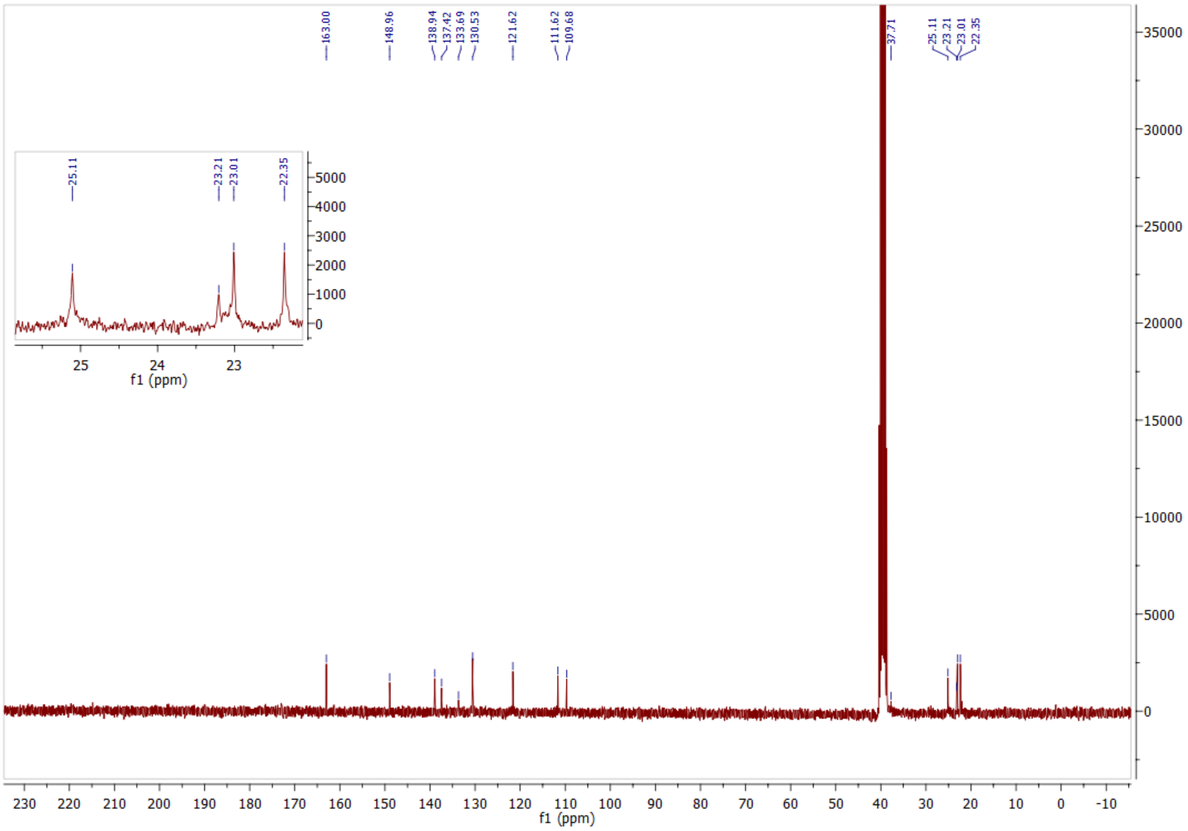

4. Supplementary Tables and Figures

Table S1. Reactivity of benzoxazole-2-carbonitrile derivatives. Compounds were marked as hyperreactive when the reaction with *N*-acetyl cysteine was too fast to be monitored. Chemical shift of the CN peak in ^{13}C NMR, and the energy barrier of the reaction between the fragment and cysteamine are represented in the last two columns.

Substituent	Position	Label	Reactivity with *N*-acetyl cysteine (UV-Vis-based assay)	Reactivity with GSH (HPLC-based assay)	^{13}C NMR chemical shift of the CN peak in DMSO-d_6 [ppm]	Calculated barrier [kcal/mol]
-Me	6	**5**	low absorbance	$t_{1/2} < 3$ min	109.78	
-OMe	4	**6**	hyperreactive	$t_{1/2} < 3$ min	109.68	6.8
	5	**7**	hyperreactive	$t_{1/2} < 3$ min	109.67	7.4
	6	**8**	hyperreactive		109.85	8.1
	7	**9**	hyperreactive	$t_{1/2} < 3$ min	109.52	7.2
-OH	4	**10**	hyperreactive		109.83	7.5
	5	**11**	hyperreactive		109.83	
	6	**12**	hyperreactive		109.98	
	7	**13**	hyperreactive		109.78	

-OCOMe	4	**14**	hyperreactive		109.81	
	5	**15**	hyperreactive		112.39	
$-NO_2$	4	**16**	hyperreactive		109.21	
	5	**17**	ND[a]		109.24	
	6	**18**	ND[b]		109.66	
	7	**19**	hyperreactive		109.16	
$-NH_2$	4	**20**	hyperreactive		109.01	7.2
	5	**21**	hyperreactive		109.95	
	6	**22**	hyperreactive	$t_{1/2}$ < 3 min	110.38	
	7	**23**	hyperreactive		109.96	
-NHCOPh	4	**24**	hyperreactive		110.13	
	5	**25**	hyperreactive	3 min < $t_{1/2}$ < 120 min	109.66	
	6	**26**	ND[b]	3 min < $t_{1/2}$ < 120 min	109.79	
	7	**27**	hyperreactive		109.57	

[a]Not determined due to insolubility in the assay buffer.

[b]Not determined due to instability in the assay buffer.

Table S2. Results of electrophilic heterocyclic library screening. Compounds with less than 50% remaining activity at 500 ◎M concentration are shown.

**Structure**	RA at 500 µM [%]
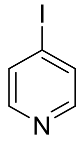	5 ± 2
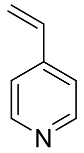	22 ± 3
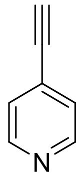	24 ± 19
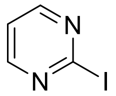	18 ± 9
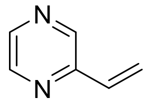	15 ± 10

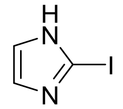	26 ± 10
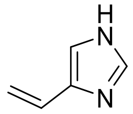	15 ± 8
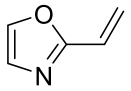	40 ± 3
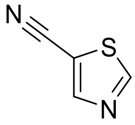	18 ± 15
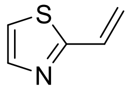	32 ± 12
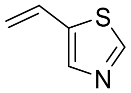	5 ± 3
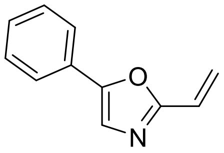	13 ± 4

Table S3. Reactivity of benzimidazole-2-carbonitrile-containing boronic acid derivatives.

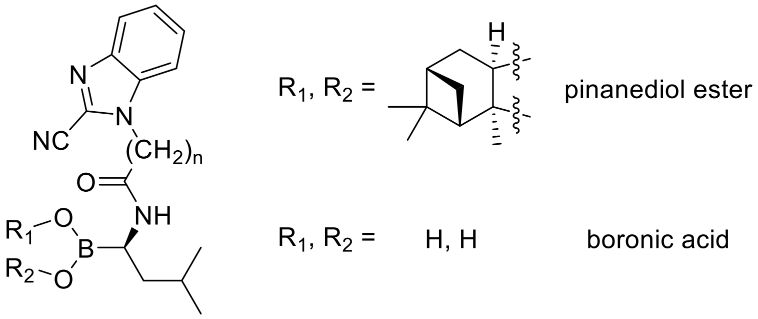

n =	X=	Y=	R_1 , R_2 =	Label	Reactivity with *N*-acetyl cysteine (UV-Vis-based assay)	Reactivity with GSH (HPLC-based assay)
n = 1	-	-	pinanediol ester	**29**	ND[a]	ND[a]
n = 1	-	-	boronic acid	**33**	ND[a]	ND[a]
n = 2	-	-	pinanediol ester	**30**	not reactive	not reactive
n = 2	-	-	boronic acid	**34**	low absorbance	low absorbance

n = 2	-Cl	-	pinanediol ester	**37**	not reactive	not reactive
n = 2	-	-Cl	pinanediol ester	**38**	not reactive	not reactive
n = 3	-	-	pinanediol ester	**31**	not reactive	not reactive
n = 3	-	-	boronic acid	**35**	not reactive	low absorbance
n = 4	-	-	pinanediol ester	**32**	not reactive	not reactive
n = 4	-	-	boronic acid	**36**	not reactive	low absorbance

[a]Not determined due to instability in the assay buffer.

Table S4. Reactivity of other boronic acid derivatives.

Structure	Label	Reactivity with *N*-acetyl cysteine (UV-Vis-based assay)	Reactivity with GSH (HPLC-based assay)
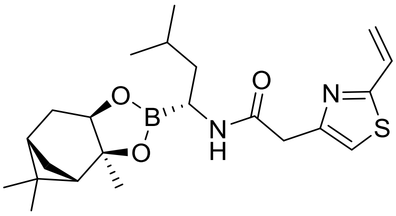	**28**	low absorbance	not reactive
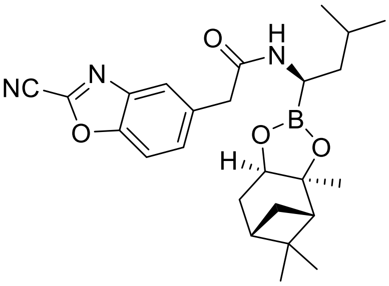	**39**	hyperreactive	3 min < $t_{1/2}$ < 60 min
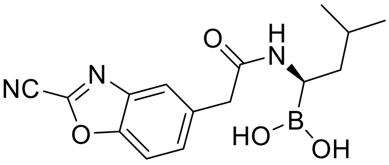	**40**	hyperreactive	low absorbance

Table S5. Determination of the IC_{50} shift for selected bidentate compounds for the inhibition of β5i activity. Compounds were assayed without pre-incubation or with 30-min pre-incubation. PR-957 (irreversible inhibitor) was used as a control.

**Pre-incubation**	**Compound IC_{50} (μM)[a]**

(min)	30	34	38	39	PR-957
0	1.5 ± 0.3	5.1 ± 0.0	6.4 ± 0.7	3.2 ± 0.4	0.102 ± 0.006
30	0.6 ± 0.0	2.2 ± 0.6	0.6 ± 0.1	3.1 ± 0.3	0.022 ± 0.009

[a]IC_{50} values are means from at least three independent determinations.

Table S6. Inhibitory potencies of selected benzoxazole-2-carbonitriles and bidentate ligands against hAChE, hBChE, MAO-A/B and caspase-1.

Cpd	Residual activity [%] ± SD at 100 µM and 30 min pre-incubation [a]				
	hMAO-A	hMAO-B	hAChE	hBChE	Caspase-1
**6**	27 ± 0%	35 ± 1%	NA	36 ± 1%	n.d.
**10**	38 ± 2%	NA	27 ± 2%	NA	9 ± 3%
**11**	26 ± 0%	29 ± 0%	27 ± 1%	NA	0 ± 1%
**13**	48 ± 1%	NA	25 ± 1%	NA	5 ± 1%
**14**	34 ± 0%	NA	11 ± 2%	NA	49 ± 5%
**15**	16 ± 0%	33 ± 0%	33 ± 2%	NA	12 ± 2%
**28**	42 ± 0%	NA	NA	27 ± 3%	NA
**30**	NA	NA	NA	NA	NA
**31**	24 ± 0%	NA	NA	NA	NA
**34**	NA	NA	NA	NA	NA
**35**	NA	NA	NA	NA	NA
**38**	24 ± 1%	42 ± 0%	NA	NA	NA
**39**	42 ± 0%	47 ± 1%	NA	45 ± 1%	NA
**40**	36 ± 0%	44 ± 0%	46 ± 1%	33 ± 1%	NA

[a]RAs are means from at least two independent determinations. NA, Not active (RA above 50%). n.d., not determined.

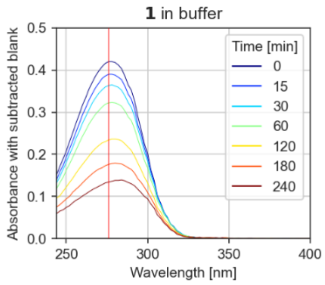

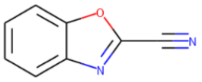

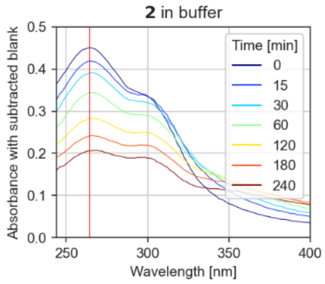

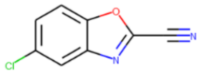

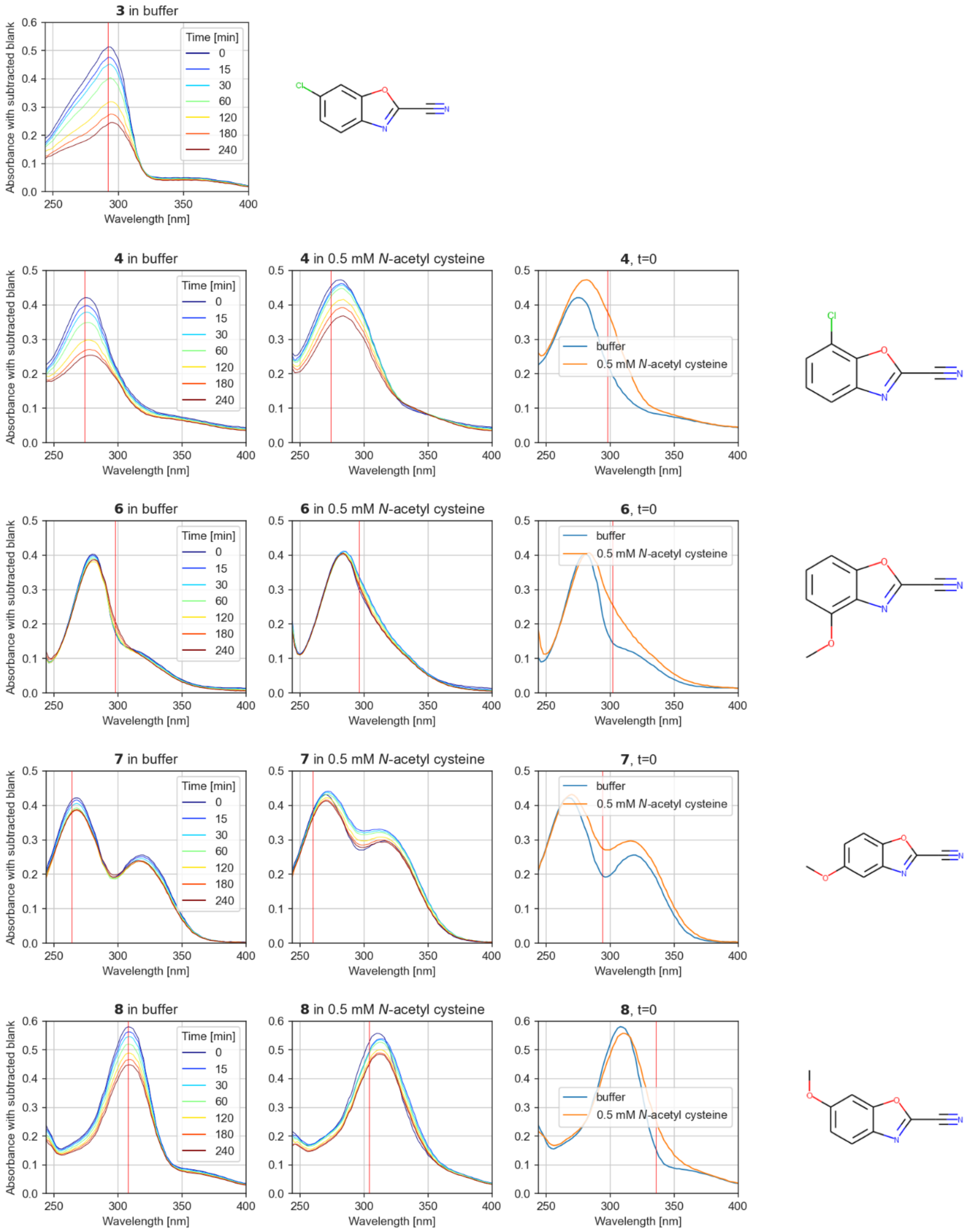

3 in buffer
4 in buffer
4 in 0.5 mM N-acetyl cysteine
4, t=0
6 in buffer
6 in 0.5 mM N-acetyl cysteine
6, t=0
7 in buffer
7 in 0.5 mM N-acetyl cysteine
7, t=0
8 in buffer
8 in 0.5 mM N-acetyl cysteine
8, t=0
Absorbance with subtracted blank
Wavelength [nm]
Time [min]
0
15
30
60
120
180
240
buffer
0.5 mM N-acetyl cysteine
Cl
O
N

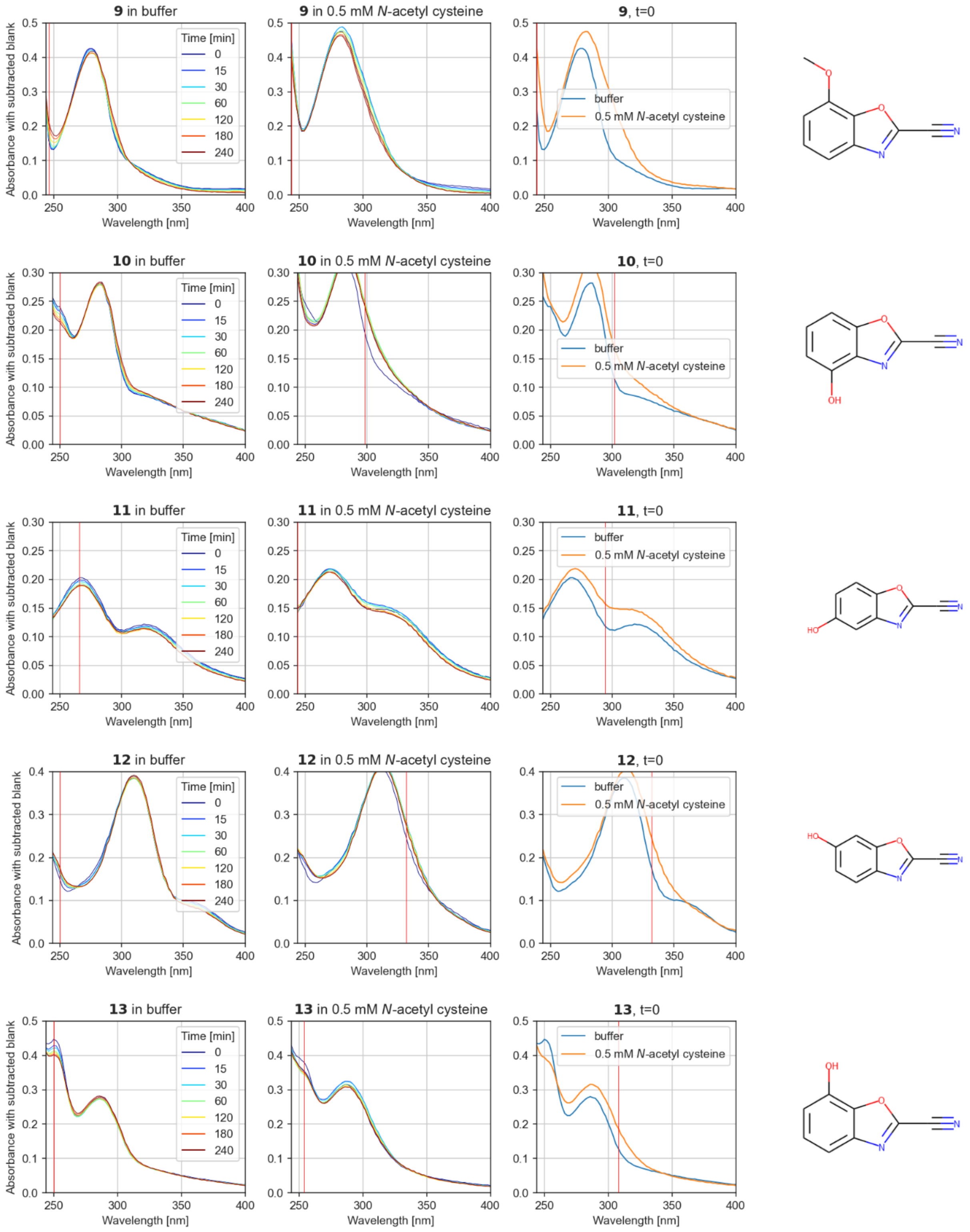

9 in buffer
9 in 0.5 mM *N*-acetyl cysteine
9, t=0
10 in buffer
10 in 0.5 mM *N*-acetyl cysteine
10, t=0
11 in buffer
11 in 0.5 mM *N*-acetyl cysteine
11, t=0
12 in buffer
12 in 0.5 mM *N*-acetyl cysteine
12, t=0
13 in buffer
13 in 0.5 mM *N*-acetyl cysteine
13, t=0
Absorbance with subtracted blank
Wavelength [nm]
Time [min]
0
15
30
60
120
180
240
buffer
0.5 mM *N*-acetyl cysteine
O
N
OH
HO

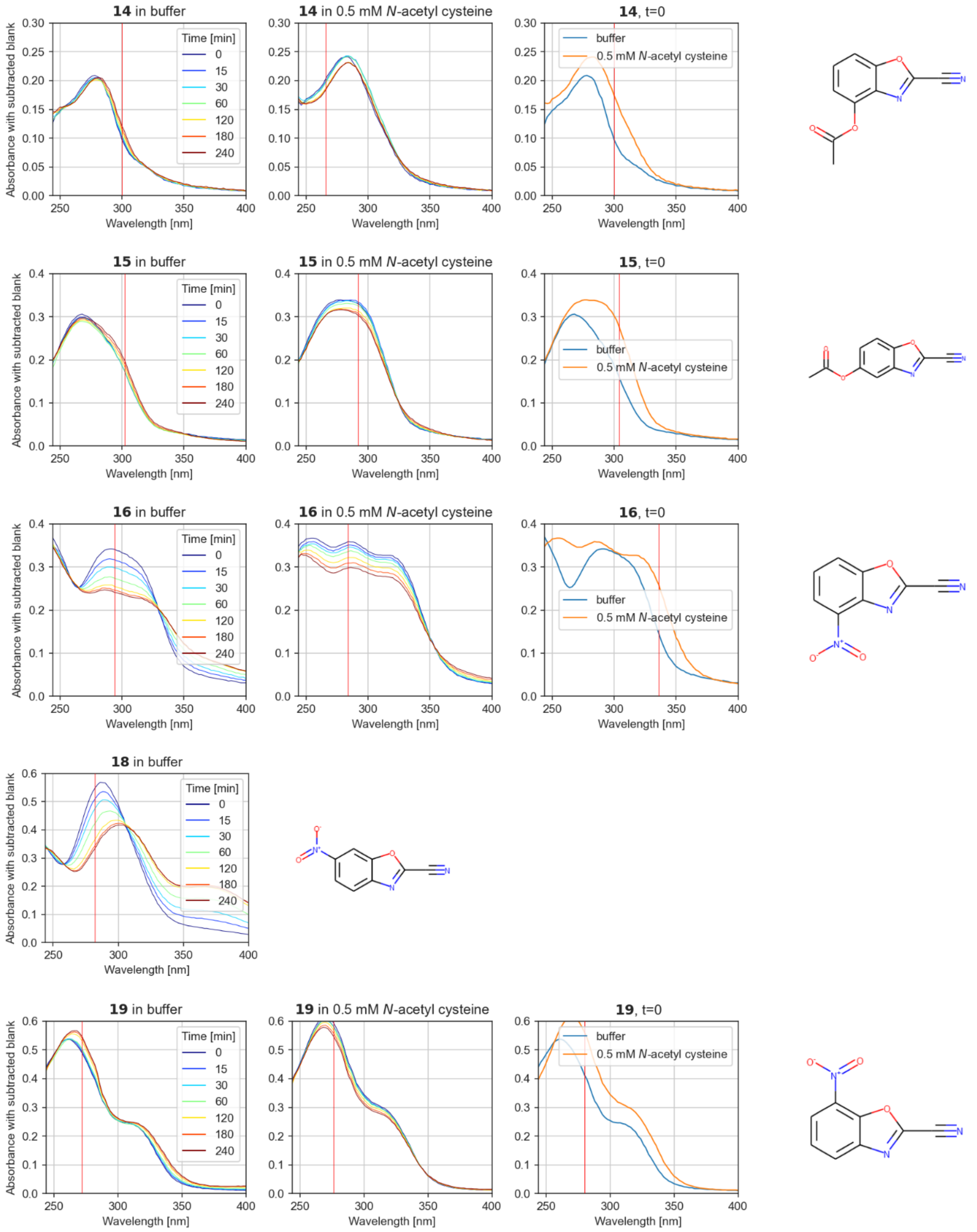

14 in buffer
14 in 0.5 mM N-acetyl cysteine
14, t=0
15 in buffer
15 in 0.5 mM N-acetyl cysteine
15, t=0
16 in buffer
16 in 0.5 mM N-acetyl cysteine
16, t=0
18 in buffer
19 in buffer
19 in 0.5 mM N-acetyl cysteine
19, t=0
Absorbance with subtracted blank
Wavelength [nm]
Time [min]
0
15
30
60
120
180
240
buffer
0.5 mM N-acetyl cysteine

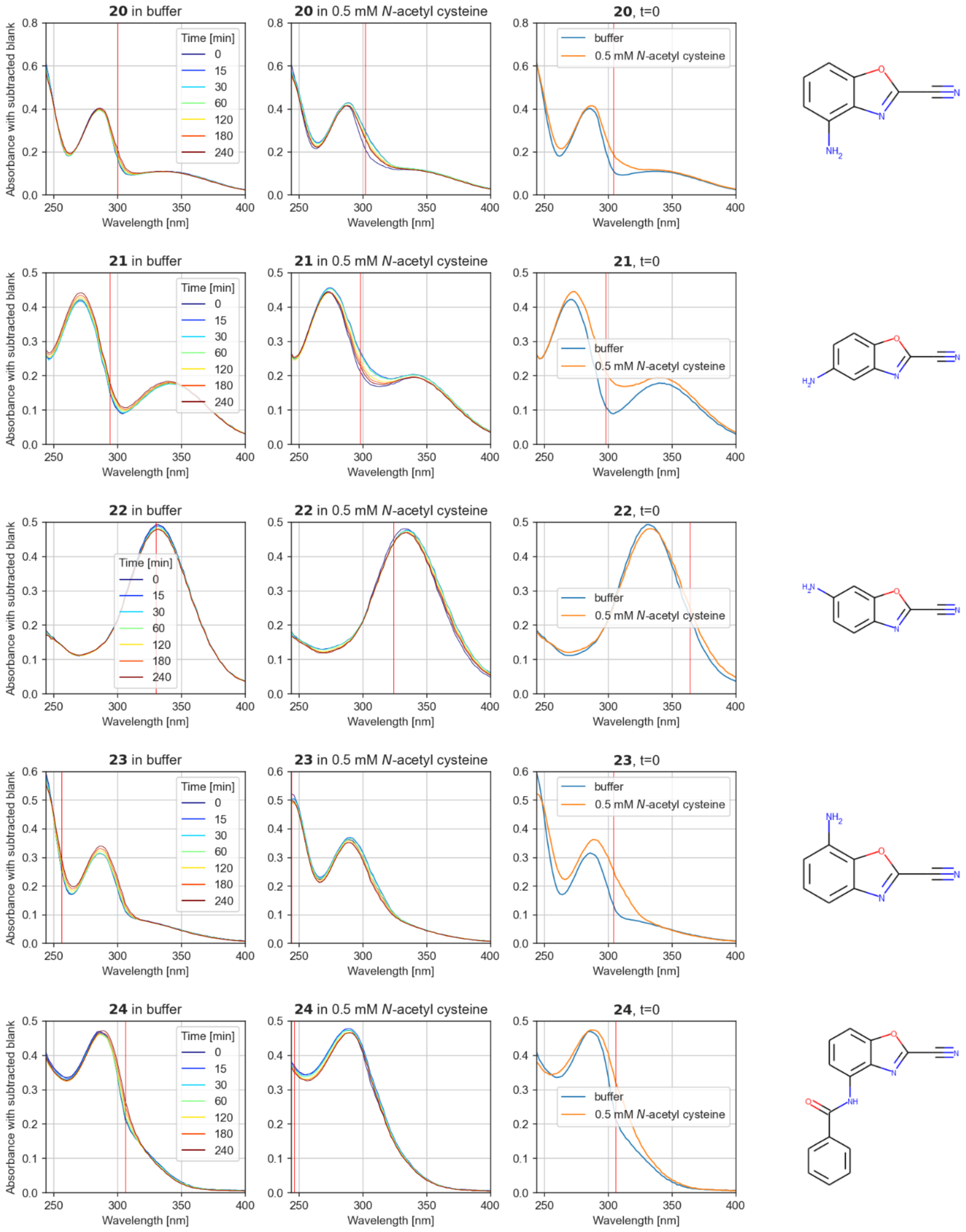

20 in buffer
20 in 0.5 mM N-acetyl cysteine
20, t=0
21 in buffer
21 in 0.5 mM N-acetyl cysteine
21, t=0
22 in buffer
22 in 0.5 mM N-acetyl cysteine
22, t=0
23 in buffer
23 in 0.5 mM N-acetyl cysteine
23, t=0
24 in buffer
24 in 0.5 mM N-acetyl cysteine
24, t=0
Absorbance with subtracted blank
Wavelength [nm]
Time [min]
0
15
30
60
120
180
240
buffer
0.5 mM N-acetyl cysteine
NH2
H2N
NH
O
N

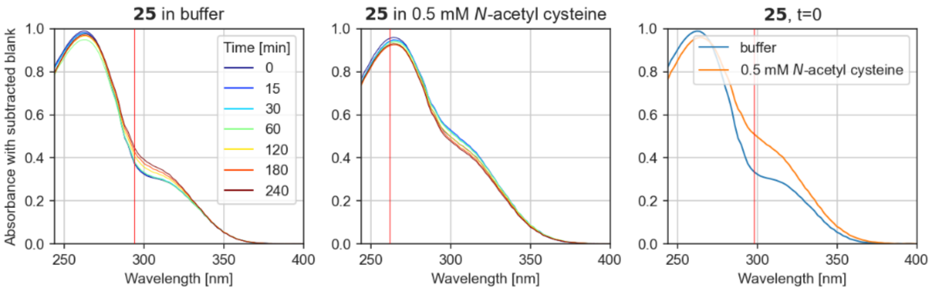
25 in buffer
25 in 0.5 mM N-acetyl cysteine
25, t=0
Absorbance with subtracted blank
Wavelength [nm]
Time [min]
0
15
30
60
120
180
240
buffer
0.5 mM N-acetyl cysteine

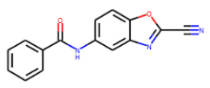

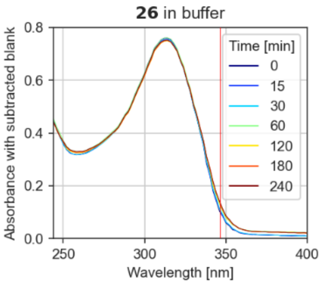
26 in buffer
Absorbance with subtracted blank
Wavelength [nm]
Time [min]
0
15
30
60
120
180
240

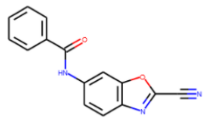

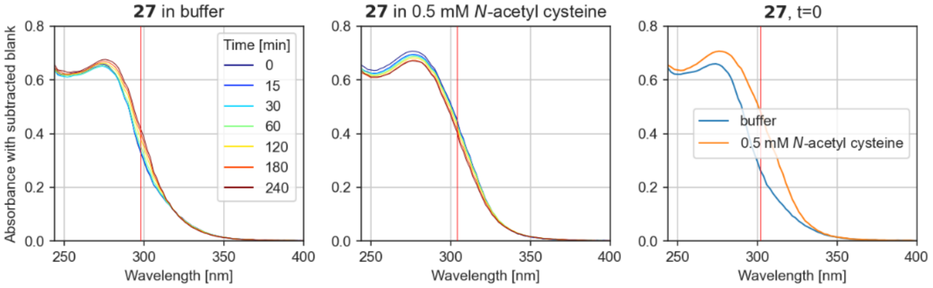
27 in buffer
27 in 0.5 mM N-acetyl cysteine
27, t=0
Absorbance with subtracted blank
Wavelength [nm]
Time [min]
0
15
30
60
120
180
240
buffer
0.5 mM N-acetyl cysteine

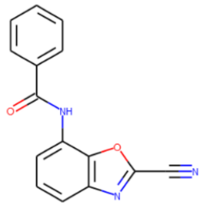

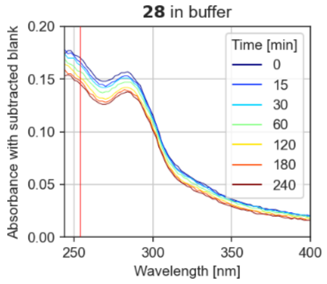
28 in buffer
Absorbance with subtracted blank
Wavelength [nm]
Time [min]
0
15
30
60
120
180
240

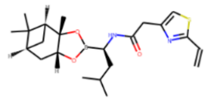

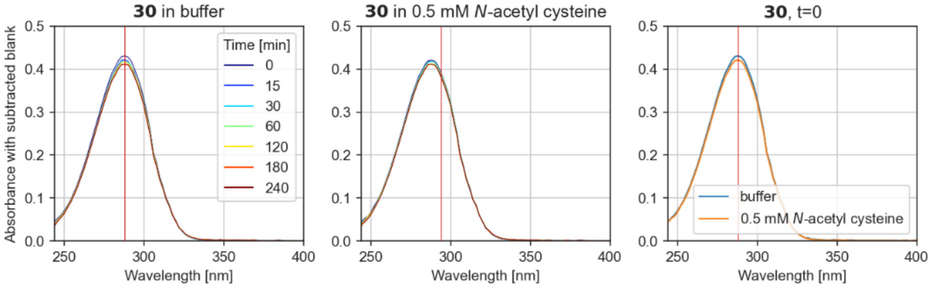
30 in buffer
30 in 0.5 mM N-acetyl cysteine
30, t=0
Absorbance with subtracted blank
Wavelength [nm]
Time [min]
0
15
30
60
120
180
240
buffer
0.5 mM N-acetyl cysteine

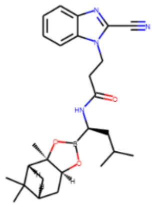

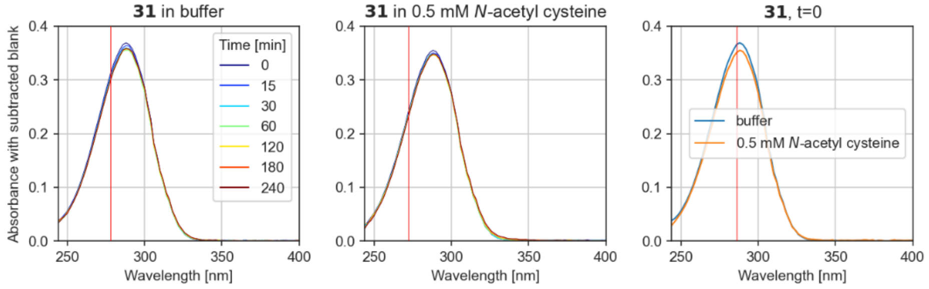
31 in buffer
31 in 0.5 mM N-acetyl cysteine
31, t=0
Absorbance with subtracted blank
Wavelength [nm]
Time [min]
0
15
30
60
120
180
240
buffer
0.5 mM N-acetyl cysteine

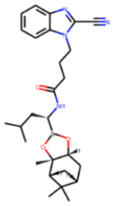

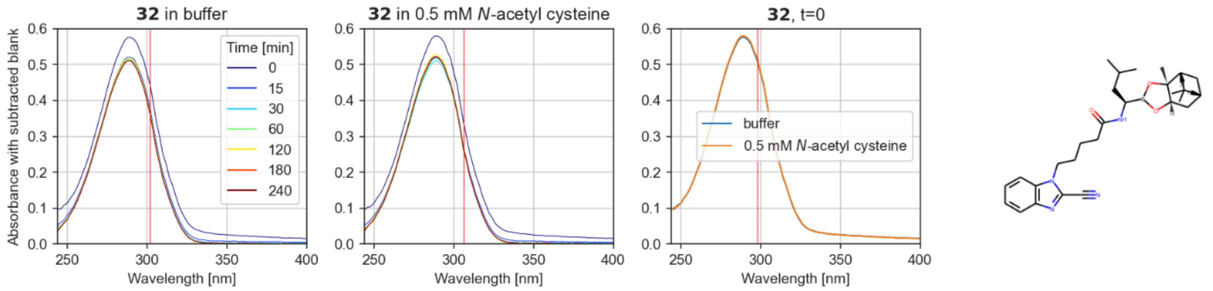
32 in buffer
32 in 0.5 mM N-acetyl cysteine
32, t=0
Absorbance with subtracted blank
Wavelength [nm]
Time [min]
0
15
30
60
120
180
240
buffer
0.5 mM N-acetyl cysteine
N
O
NH

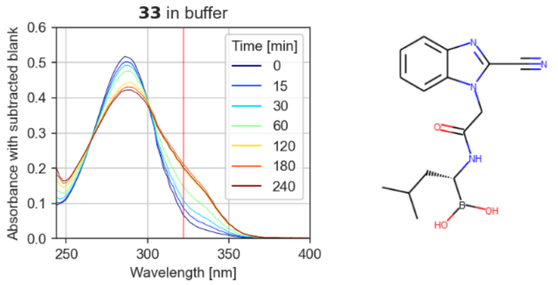
33 in buffer
Absorbance with subtracted blank
Wavelength [nm]
Time [min]
0
15
30
60
120
180
240
N
O
NH
B
OH
HO

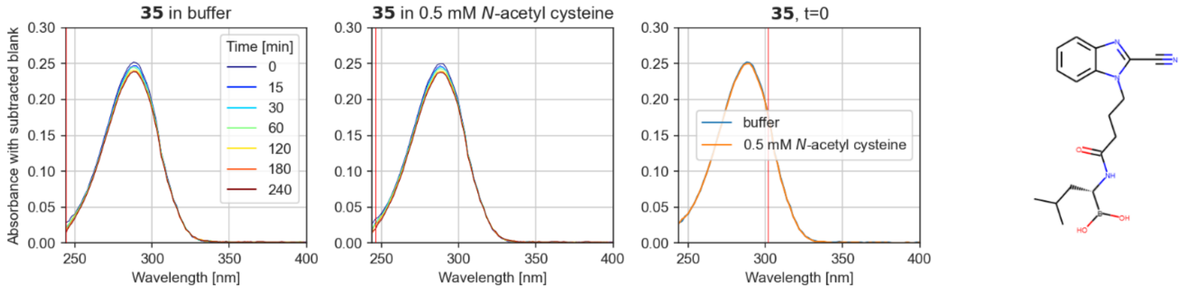
35 in buffer
35 in 0.5 mM N-acetyl cysteine
35, t=0
Absorbance with subtracted blank
Wavelength [nm]
Time [min]
0
15
30
60
120
180
240
buffer
0.5 mM N-acetyl cysteine

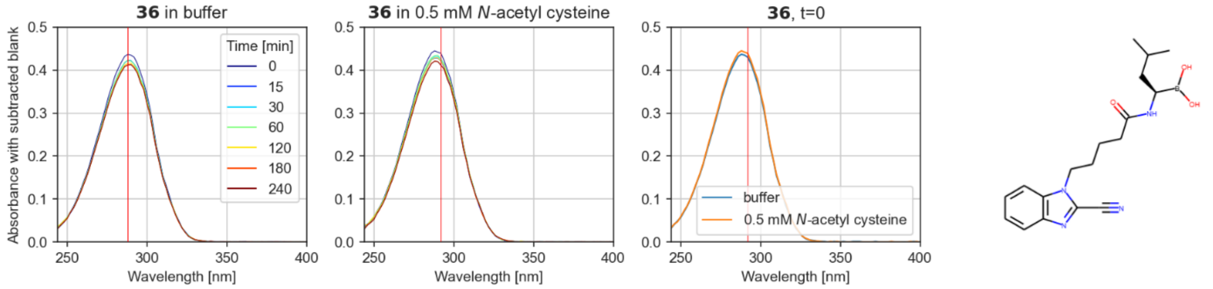
36 in buffer
36 in 0.5 mM N-acetyl cysteine
36, t=0
Absorbance with subtracted blank
Wavelength [nm]
Time [min]
0
15
30
60
120
180
240
buffer
0.5 mM N-acetyl cysteine

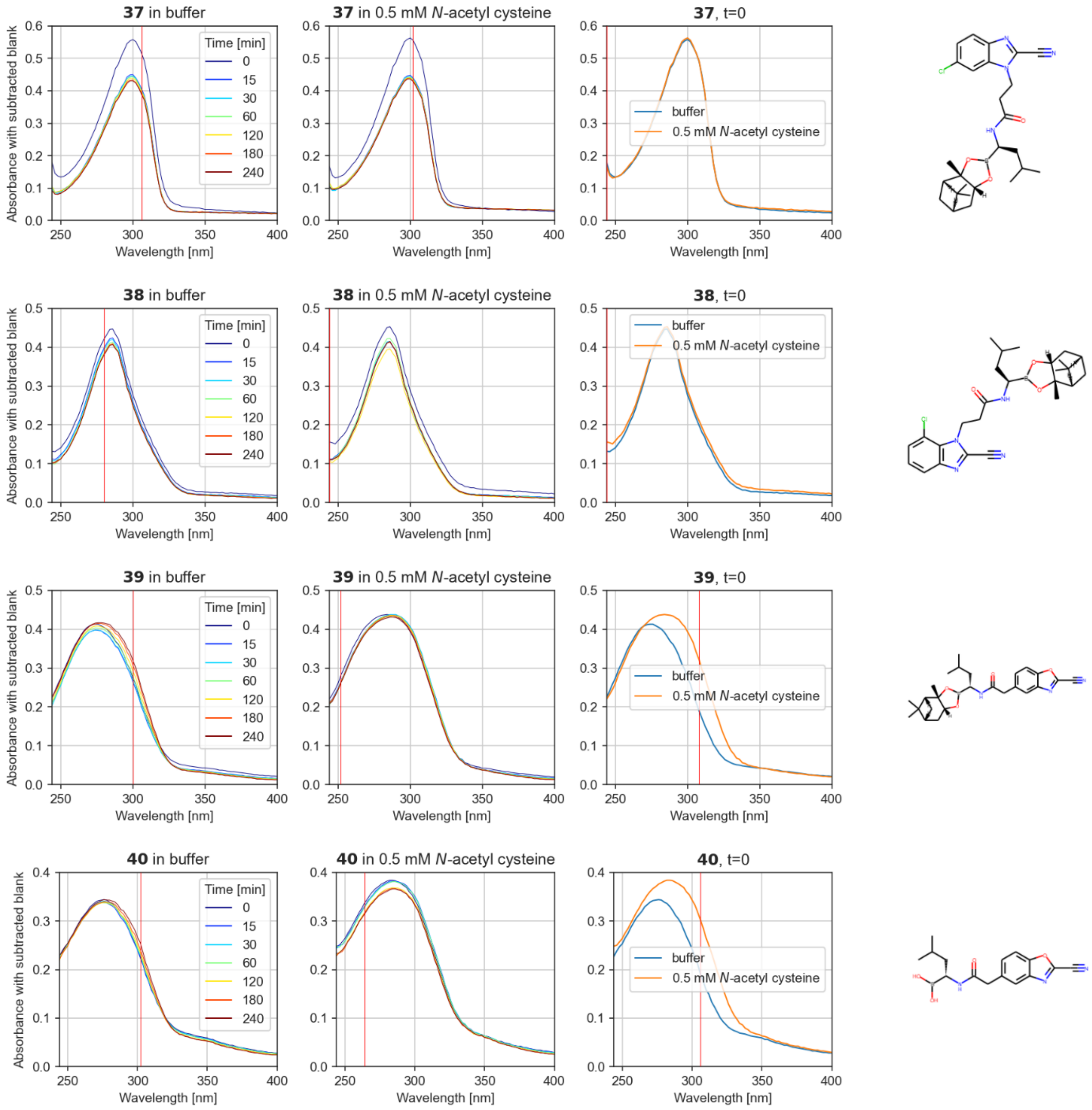

Figure S1. Absorption spectra from the UV-Vis-based stability and reactivity assays. The first plot shows stability in aqueous solution, with significant changes indicating instability. The second plot shows reactivity with *N*-acetyl cysteine, which was determined only for compounds that are stable or intermediately stable in buffer. The third graph was used to identify hyperreactive compounds, where the reaction with *N*-acetyl cysteine is complete before the first time point can be acquired. Significant differences in the spectra of the compound in the buffer solution with or without 0.5 mM *N*-acetyl cysteine at the first time point (t = 0 min) indicate hyperreactivity. Stability and reactivity were not determined by this assay for compounds **5**, **28** and **34** due to their low absorbance and for compound **17** due to its poor solubility.

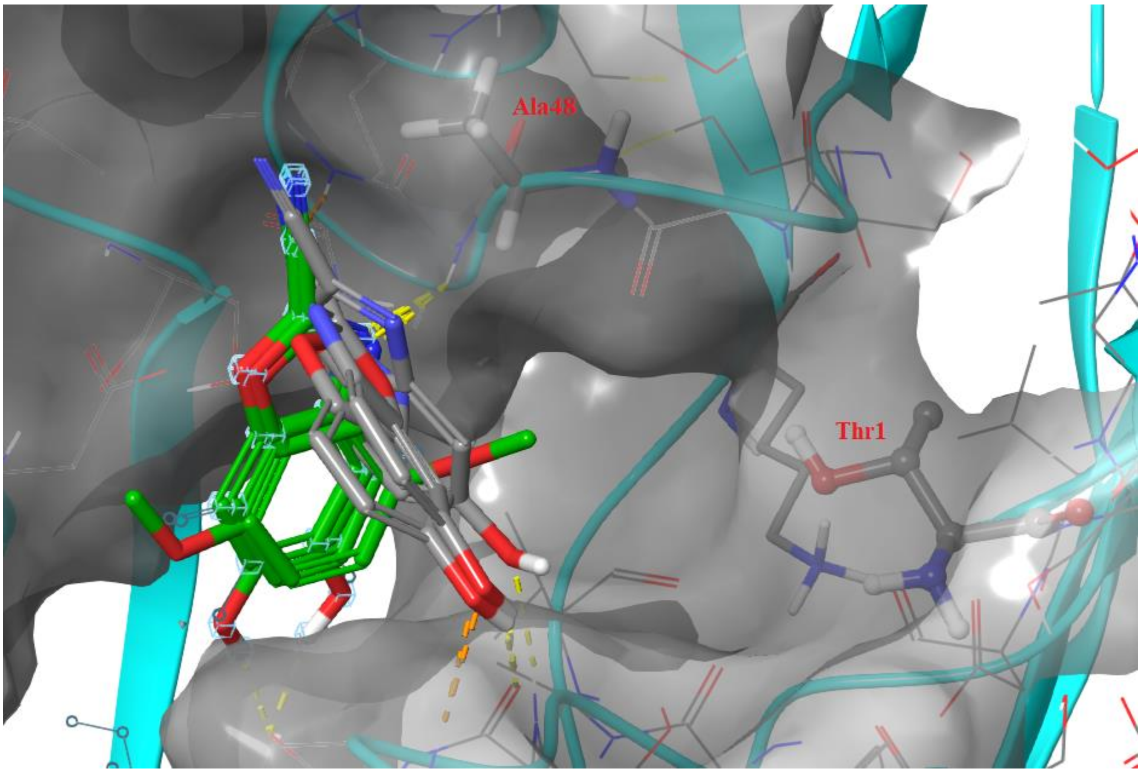

Figure S2. Binding poses of OH- and OMe-substituted benoxazole-2-carbonitriles form two clusters both separated by 8–9 Å from the catalytic Thr1.

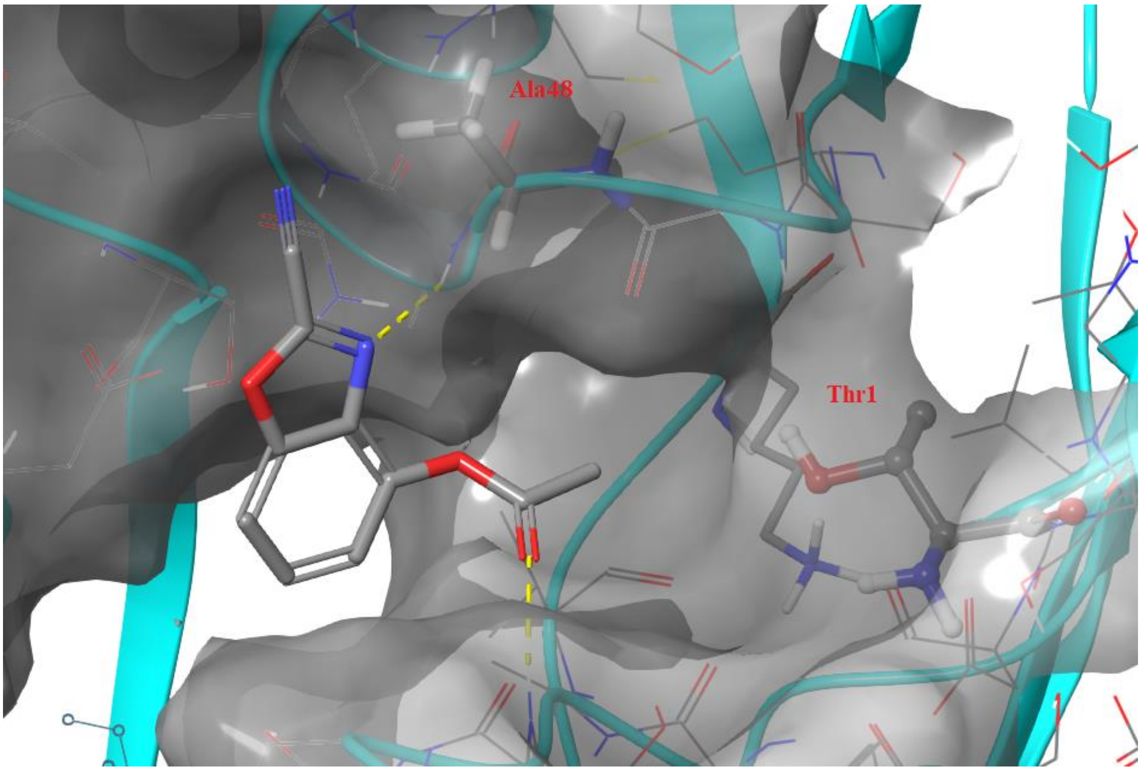

Figure S3. Putative binding pose of 4-OCOMe-substituted benzoxazole-2-carbonitrile **14**. The substituent at position 4 extends toward the catalytic Thr1 residue.

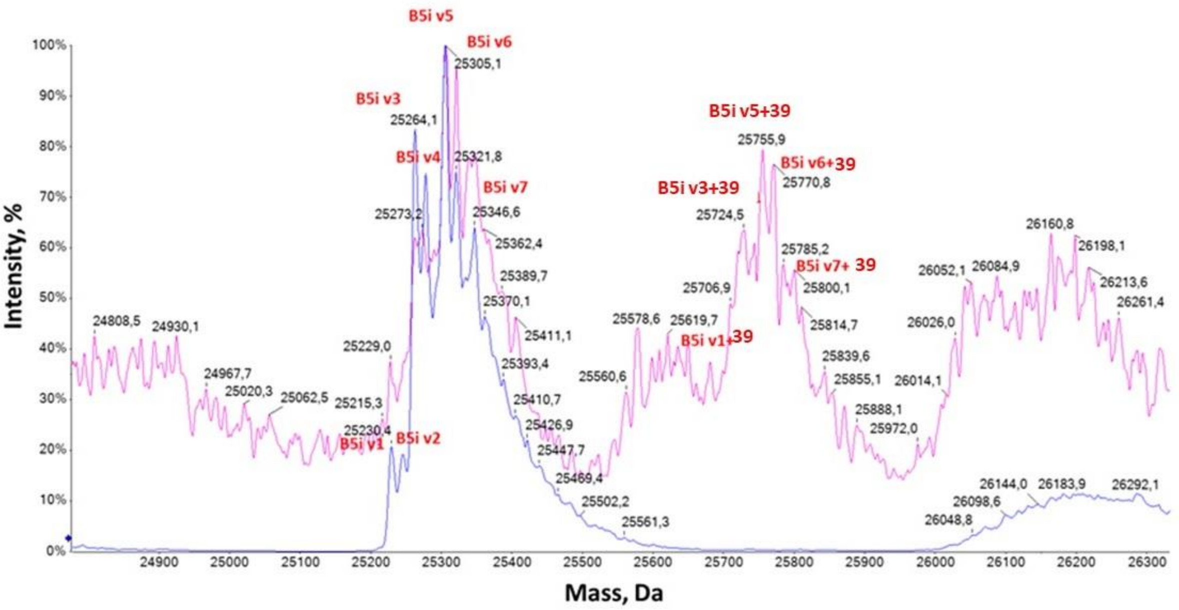

Figure S4. Deconvoluted mass spectrum of reference β5i (PSMB8 Human) (marked with blue) and β5i modified by **39** (449 Da) (marked with magenta). Multiple peaks appear to be due to the presence of several mutant variants in both the reference and the modified β5i.

References

1. Cozza, G.; Gianoncelli, A.; Bonvini, P.; Zorzi, E.; Pasquale, R.; Rosolen, A.; Pinna, L.A.; Meggio, F.; Zagotto, G.; Moro, S. Urolithin as a Converging Scaffold Linking Ellagic acid and Coumarin Analogues: Design of Potent Protein Kinase CK2 Inhibitors. *ChemMedChem* **2011**, *6*, 2273–2286, doi:10.1002/cmdc.201100338.
2. Kollár, L.; Gobec, M.; Szilágyi, B.; Proj, M.; Knez, D.; Ábrányi-Balogh, P.; Petri, L.; Imre, T.; Bajusz, D.; Ferenczy, G.G.; et al. Discovery of selective fragment-sized immunoproteasome inhibitors. *Eur. J. Med. Chem.* **2021**, *219*, doi:10.1016/j.ejmech.2021.113455.
3. Woodroofe, C.C.; Meisenheimer, P.L.; Klaubert, D.H.; Kovic, Y.; Rosenberg, J.C.; Behney, C.E.; Southworth, T.L.; Branchini, B.R. Novel heterocyclic analogues of firefly luciferin. *Biochemistry* **2012**, *51*, 9807–9813, doi:10.1021/bi301411d.
4. Möller, H. Zur Chemie 2-substituierter Benzoxazole. *Justus Liebigs Ann. Chem.* **1971**, *749*, 1–11, doi:10.1002/jlac.19717490102.
5. Kobayashi, R.; Hanaya, K.; Shoji, M.; Umezawa, K.; Sugai, T. A Chemo-Enzymatic Expeditious Route to Racemic Dihexanoyl (2R*, 3R*, 4R*)-Dehydroxymethylepoxyquinomycin (DHMEQ), the Precursor for Lipase-Catalyzed Synthesis of the Potent Nuclear Factor-κB Inhibitor, (2S, 3S, 4S)-DHMEQ. *Chem. Pharm. Bull.* **2012**, *60*, 1220–1223, doi:10.1248/cpb.c12-00417.
6. Rubino, M.T.; Maggi, D.; Laghezza, A.; Loiodice, F.; Tortorella, P. Identification of Novel Matrix Metalloproteinase Inhibitors by Screening of Phenol Fragments Library. *Arch. Phram.*

Chem. Life Sci. **2011**, *344*, 557–563, doi:10.1002/ardp.201000350.

7. Reddy, G.L.; Sarma, R.; Liu, S.; Huang, W.; Lei, J.; Fu, J.; Hu, W. Design, synthesis and biological evaluation of novel scaffold benzo[4,5]imidazo [1,2-a]pyrazin-1-amine: Towards adenosine A2A receptor (A2A AR) antagonist. *Eur. J. Med. Chem.* **2021**, *210*, 113040, doi:10.1016/j.ejmech.2020.113040.
